# Rapid Dereplication of Trunk Bark Constituents of *Croton sylvaticus* and Molecular Docking of Terpenoids from Three Congolese *Croton* Species

**DOI:** 10.3390/ijms26094305

**Published:** 2025-05-01

**Authors:** Bienvenu Kamalandua Mvingu, Tienabe Nsiama, Obed Nsemi Kanga, Kalulu Muzele Taba, Jason Thambwe Kilembe, Jean-Noël Kanyinda Mputu, Sarah Garifo, Céline Henoumont, Dya Fita Dibwe, Blaise Mavinga Mbala, Sophie Laurent

**Affiliations:** 1 Mention Chimie et Industrie, Faculté des Sciences et Technologies, Université de Kinshasa, Kin XI, Kinshasa P.O. Box 190, Democratic Republic of the Congo; bienvenu.mvingu@unikin.ac.cd (B.K.M.); tienabe@gmail.com (T.N.); obedkangansemi@gmail.com (O.N.K.); jason.kilembe@unikin.ac.cd (J.T.K.); jean-noel.mputukanyinda@umons.ac.be (J.-N.K.M.); blaise.mbala@unikin.ac.cd (B.M.M.); 2 NMR and Molecular Imaging Laboratory, General, Organic and Biomedical Chemistry Unit, University of Mons, 19 Avenue Maistriau, 7000 Mons, Belgium; sarah.garifo@umons.ac.be (S.G.); celine.henoumont@umons.ac.be (C.H.); sophie.laurent@umons.ac.be (S.L.); 3 Faculty of Health Sciences, Hokkaido University, Kita-12, Nishi-5, Kita-Ku, Sapporo 060-0812, Japan

**Keywords:** ^1^H- and ^13^C-NMR metabolomics, aleuritolic acid, phytol, caryophyllene oxide, metabolomic analysis

## Abstract

Phytochemical investigation and bioactivity evaluation of terpenoids from the *Croton* species were conducted. The chemical composition of *C. sylvaticus* was explored using chemical phytochemical screening techniques and dereplication of ^13^C NMR data using MixONat software (v. 1.0.1). Natural products with diverse structural features were identified in the dichloromethane extract of trunk bark. These include monoterpenoids, sesquiterpenoids, diterpenoids, triterpenoids, along with other minor metabolites, such as steroids, saponins, and fatty acids. Further purification of this extract led to the isolation of three major secondary metabolites, acetyl aleuritolic acid, caryophyllene oxide, and phytol. These secondary metabolites were reported for the first time in *C. sylvaticus*. The isolated compounds were structurally compared to known anticancer terpenoids previously identified in two other Congolese *Croton* species. Through molecular docking studies, the predicted binding affinities of the identified compounds were assessed, and possible structure–activity relationships (SAR) were proposed. Two structurally characterized receptors—the human androgen receptor (HAR, PDB ID: 1E3G) and hypoxia-inducible factor 1-alpha (HIF-1α, PDB ID: 3KCX), known for their involvement in cancer-related pathways, were used for molecular docking investigations. Among the tested compounds, **1**, **2**, **3**, and **12** were identified as having strong-to-moderate predicted binding affinities to both protein targets, along with favorable drug-like properties according to the ADMET analysis. This investigation could justify the use of *Croton* plants in traditional medicine. In addition, our study highlights the potential of the Congolese *Croton* species as sources of bioactive secondary metabolites.

## 1. Introduction

The genus *Croton* comprises more than 1300 species of trees, shrubs, and herbs prevalent in pantropical regions worldwide [1]. The genus is used in traditional medicine globally [2,3]. The African species of this genus are found in western and central Africa. Among the 50 species reported in Africa, 22 are recorded in various regions of the DR Congo [4]. The country is known for its rich plant biodiversity. This flora is an attractive and potential reservoir to be explored for the discovery of novel bioactive metabolites. Species from its flora have not been deeply explored, and *Croton sylvaticus* is a species that has historical uses in folk medicine, yet its phytochemical composition is understudied. *Croton* chemistry is dominated by diterpenoids. These secondary class metabolites are reported for their wide range of biological activities [5,6,7,8]. Previous studies on the Congolese species, *C. mubango* and *C. haumanianus*, have resulted in the identification of diterpenoids with antiproliferative activity [9,10]. These studies demonstrate the potential of the *Croton* species to be an interesting source of bioactive natural products. Despite its medicinal relevance, the phytochemistry of the Congolese *C. sylvaticus* remains underexplored. Investigations of *C. sylvaticus* from other regions resulted in the identification of several classes of secondary metabolites. These include alkaloids, anthraquinones, essential oils, flavonoids, lignans, phenolic acids, sterols, tannins, and terpenoids [11]. However, the Congolese species may differ in its metabolite profile, since a metabolome composition may vary due to environmental factors. In addition, the plant has been reported to possess various biological activities. Reported activities include antibacterial, antifungal, anti-inflammatory, antioxidant, and larvicidal effects [12,13,14,15,16].

As the second leading cause of death, cancer is a global burden [17], and natural products remain a vital source of potential anticancer agents. The diterpenoids, the major constituents of the *Croton* genus are among the promising anticancer candidates [18,19,20]. Paclitaxel is a natural product with a diterpenoid main structure that is in use in clinical oncology. Paclitaxel has shown notable cytotoxic activity against both breast and prostate cancer cell lines [21,22]. Previous research has demonstrated that diterpenoids from the *Croton* species possess anticancer properties, especially against human prostate and breast cancer cell lines PC-3 and MCF-7. Moreover, several medicinal plants, including five extracts of *Gardenia ternifolia*, were tested for cytotoxicity against PC-3 and MCF-7 cells, suggesting that Congolese medicinal and food plants are a good source of bioactive secondary metabolites [21,22].

Molecular docking studies are in silico techniques that have been proven to be useful tools in drug discovery. These techniques enable the evaluation of the interaction of phytochemicals with cancer-related molecular targets such as HIF-1α and the human androgen receptor. HIF-1α is recognized as a key regulator in tumor hypoxia responses. Its overexpression in many solid tumors plays a key role in the activation of genes under hypoxic conditions [23,24]. Its relevance in cancer progression has made it a target of therapeutic inhibitors, including echinomycin, which has shown efficacy in preclinical studies [23,24]. Similarly, HAR is involved in androgen-dependent cancer progression, including in prostate and breast cancers. It mediates pathways for tumor growth and survival [25,26,27]. Both HIF-1α and HAR were selected for their reported high-resolution crystal structures, which enable precise docking predictions to support drug discovery efforts [28]. More importantly, HAR is the target of enzalutamide, an approved therapy for prostate cancer that effectively blocks androgen receptor signaling [26,29]. Using these in silico techniques, secondary metabolites from *C. sylvaticus* could be investigated to determine their anticancer potential measuring their interactions with specific cancer-related targets.

Our ongoing research focuses on investigating the potential bioactive phytochemical constituents of the Congolese *Croton* species extracts, with particular emphasis on evaluating the predicted binding affinity of isolated terpenoids to two structurally characterized receptors—the human androgen receptor (HAR, PDB ID: 1E3G) and hypoxia-inducible factor 1-alpha (HIF-1α, PDB ID: 3KCX), associated with PC-3 and MCF-7 cancer cell lines, respectively, through molecular docking studies. This investigation is a comprehensive phytochemical analysis of Congolese *C. sylvaticus*. An examination of its metabolome was conducted by 1D NMR-based analysis, facilitated by MixONat software. tour aims are as follows: (i) to characterize the metabolomic profile of the apolar extract from the trunk bark and isolate and characterize major metabolites from this extract; (ii) to conduct in silico studies to determine their anticancer potential along with well-established anticancer diterpenoids from *C. mubango* and *C. haumanianus* through structure–activity relationship (SAR) and molecular docking studies; and (iii) to acquire useful knowledge of their anticancer potential to guide future in vitro and in vivo investigations.

Our research program aims to characterize the metabolome of the *Croton* species using recent techniques and determine their therapeutic potential. In this approach, metribolone, clioquinol, and paclitaxel will serve as references. Predictions against benchmarks are employed prior to in vitro testing to guide future screening and bioguided isolation, thereby expediting the discovery of potentially active compounds. This investigation aims to contribute to the body of knowledge that supports the therapeutic potential of the Congolese *Croton* species.

## 2. Results and Discussion

### 2.1. Chemical Screening

**Figure 1:**
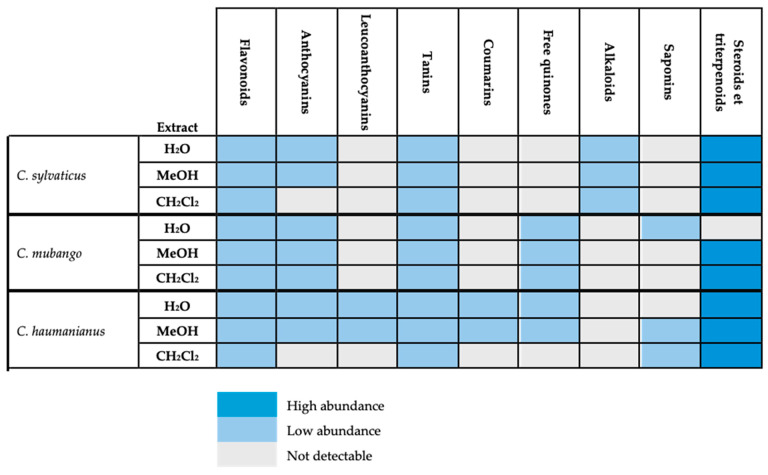
Heat map screening of *C. mubango*, *C. haumanianus*, and *C. sylvaticus*.

Phytochemical screening of *C. sylvaticus* was carried out and compared with those of *C. haumanianus* and *C. mubango*, which are two species with well-established metabolite profiles (Figure 1). The apolar and polar extracts of the three species had similar compositions. Steroids and triterpenoids were found to be abundant across all species except for the aqueous extract of *C. mubango*, where they were absent. The presence of steroids and terpenoids in less polar solvents indicates their lipophilic nature, which is often associated with anti-inflammatory, antimicrobial, antimalarial, analgesic, and antitumor properties [15,16].

### 2.2. Rapid Dereplication of EDCM from C. sylvaticus

**Figure 2:**
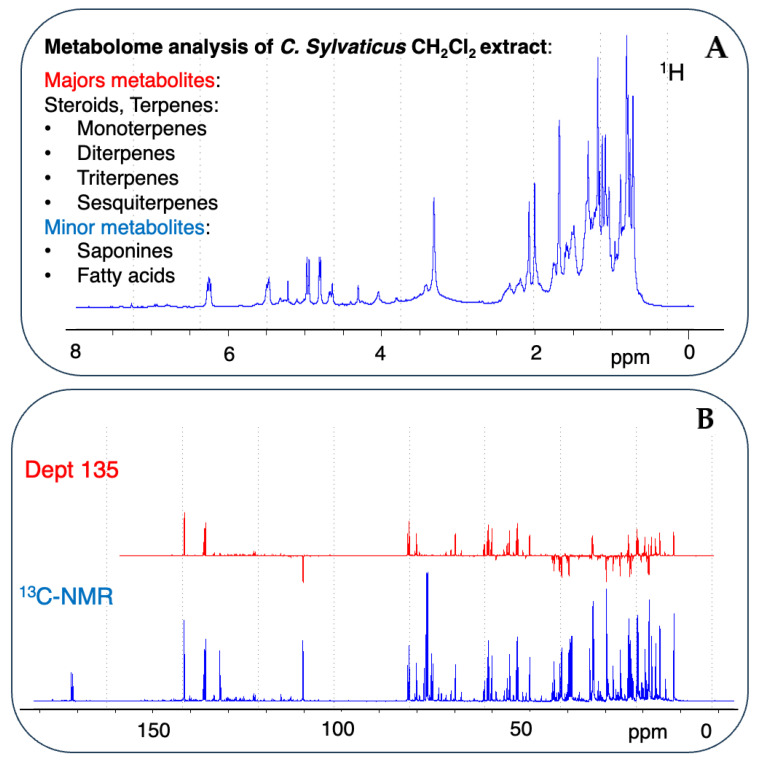
Fingerprinting and signature of potential metabolites from a mixture of natural products in CH_2_Cl_2_ extract of *C. sylvaticus* using 1D-NMR spectra. (**A**) The ^1^H-NMR profile of CH_2_Cl_2_ extract of *C. sylvaticus* with *δ*_H_ ranging from 0 to 8.0 ppm. (**B**) The ^13^C-NMR and Dept 135 spectra of CH_2_Cl_2_ extract of *C. sylvaticus* in CDCl_3_ with *δ*_C_ ranging from 0 to 180 ppm.

A metabolomic analysis by dereplication was performed by comparing the experimentally acquired ^13^C NMR data with the predicted chemical shifts recorded in the database [30] (Figure 2). The number of matching carbons *δ*_C_ (displacement tolerance range of 0.4 ppm) was calculated and presented as a “score”, with higher scores indicating better matches. Based on the score obtained, an analysis of EDCM suggested five main types of NPs with diverse structural features. The majority of dereplicated compounds in DB-1 and DB-2 were produced by the terpenoid NP pathway, and other NP pathways, such as fatty acids, amino acids and peptides, carbohydrates, shikimates, and phenylpropanoids, were responsible for several minor dereplicated compounds (Supporting Information: SM-1 and SM-2). The most abundant compounds identified in the Lamiaceae-DB-1 were terpenoids, with a total of 376 compounds dereplicated, including 10 triterpenoids, 34 diterpenoids, 181 sesquiterpenoids, and 151 monoterpenoids. Additionally, several minor compounds were identified 3 steroids, 2 saponins, 22 fatty acids, and 70 other compounds. All compounds were unequivocally identified in the mixture, with scores ranging from 1 to 0.87, as per the output of MixONat. In this study, the analysis was expanded to a more specific DB for *Croton*, a newly in-house Euphorbiaceae-DB2 was built from molecules extracted in LOTUS DB. The Euphorbiaceae-DB2 was used for dereplication using MixONat. Significant differences were noted between the types of compounds identified and their skeletal structures in the Lamiaceae-DB1 and the Euphorbiaceae-DB2 (Tables S1 and S2). Both the DB1 and DB2 contain the top 500 dereplicated compounds and, regarding chemical diversity, their distribution varies (Table S1). The DB2 has a higher count of triterpenes (222 in DB2 compared to 10 in DB1), steroids (58 in DB2 compared to 5 in DB1), and diterpenes (105 in DB2 compared to 34 in DB1); whereas the DB1 features more sesquiterpenes (181 in DB1 compared to 44 in DB2) and monoterpenes (151 in DB1 compared to 20 in DB2). This suggests that the DB2 may be more oriented towards detecting complex structures, like triterpenes and steroids, while the DB1 emphasizes a broader range of volatile terpenes. Analyzing the skeletal structures in Table S2 supports this observation. The DB2 presents a diverse array of diterpene skeletons, such as lathyrane, atisane, beyerane, and cleistanthane, which are absent in the DB1. Additionally, the DB2 has a greater number of labdane (29 vs. 10) and clerodane (14 vs. 7), indicating better coverage of structurally diverse diterpenes. Table S4 shows the distribution of compound scores in the DB1 and DB2 databases, highlighting the superior compound coverage by the DB2. In the DB1, compounds with a maximum score of 1.0 are ranked between 1 and 130; whereas, in the DB2, this range extends from 1 to 249, indicating better compound coverage in the DB2. Scores between 0.97 and 0.96 identified 7 compounds (131–137) in the DB1, compared to 195 (250–445) in the DB2. Scores between 0.95 and 0.90 show the DB1 ranking compounds between 138 and 415, while the DB2 ranks them between 446 and 500. Conversely, the DB1 includes compounds with lower scores (0.89–0.87, ranked between 416 and 500), which are not present in the DB2. 

Many of the secondary metabolites dereplicated have not yet been reported in the literature. This demonstrates the capability of MixONat in rapidly identifying major NPs without the need for labor-intensive isolation procedures.

Past studies on *C. sylvaticus* have reported several compounds, and their biological activities include antibacterial, antifungal, anti-inflammatory, antioxidant, CNS, larvicidal, and mutagenic effects [11,12,13,14,15,16,31,32].

An analysis by dereplication corroborated that the *Croton* species mainly produce terpenoid-type secondary metabolites. An EDCM purification attempt yielded three major compounds, identified as acetyl aleuritolic acid (**6**), caryophyllene oxide (**7**), and phytol (**8**), by comparing their proton and carbon NMR data with those previously reported in the literature [Supporting information: Table S8]. Furthermore, phytol (**8**) was identified as a dereplicated compound in the mixture [rank 144, score: 0.95 (19/20C), DB-1] and with similarity to compounds of ranks 148 (0.95, 19/20) and 397 (0.9, 18/20) in DB1. 

**Figure 3:**
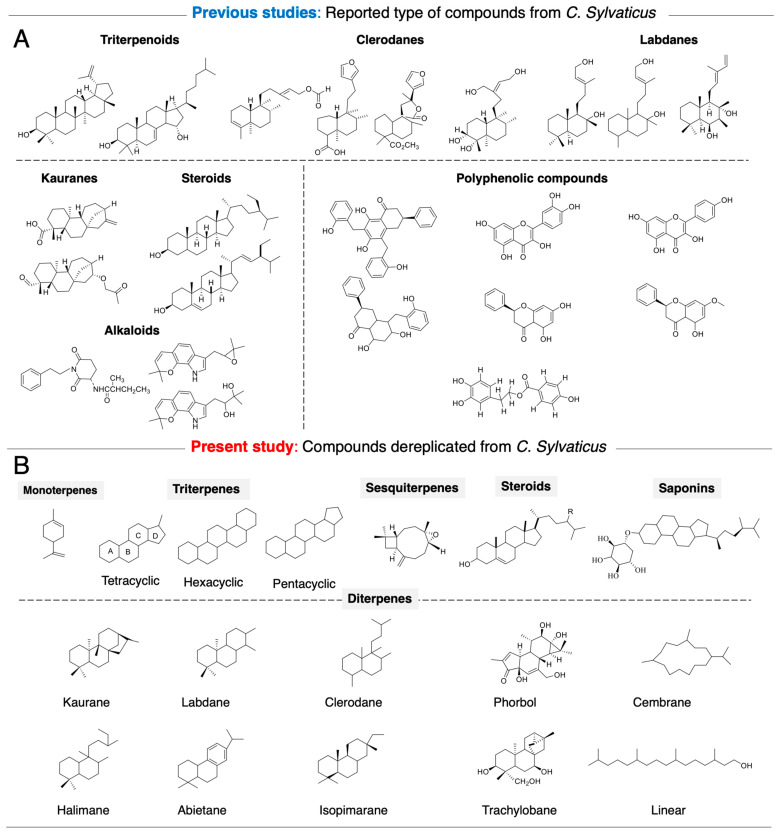
(**A**) Previous study: Main class of NPs reported from *C. sylvaticus* collected in Science Finder Scholar. (**B**) Present study: Main dereplicated type of compounds based on ^13^C-NMR and Dept with MixONat approach.

In the DB2, the compound was directly identified with several stereochemical variants having the same score of 0.95 (19/20C) at ranks 459, 466, 467, and 475, suggesting better structural coverage. However, another similar compound was detected at rank 460 (0.95, 19/20). The DB2 offers better differentiation of phytol variants, while the DB1 appears to be more restricted in the number of entries. This underlines the importance of using appropriate databases in these dereplication processes, particularly when incorporating potential metabolites to verify their existence (Figure 3A,B).

### 2.3. Phytochemical Analysis

**Figure 4:**
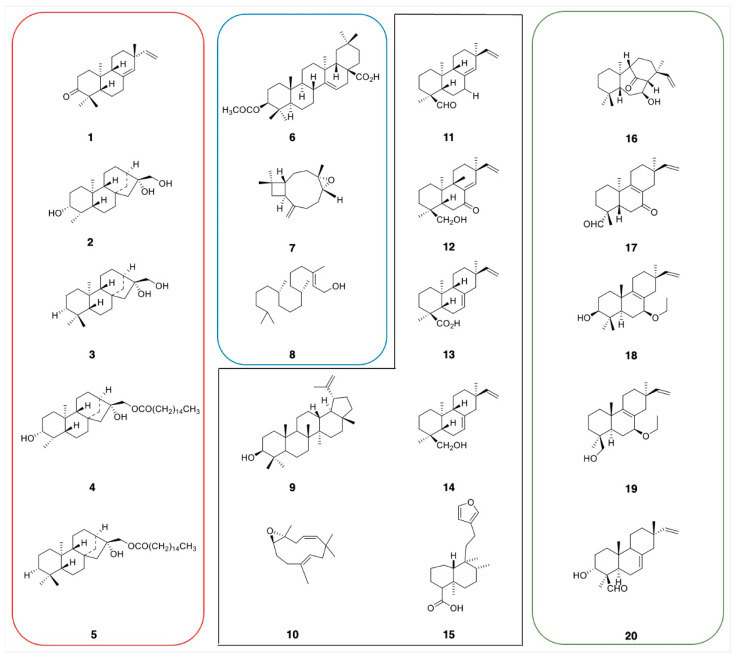
Structures of the selected compounds used in this study: In red: anti-cancer candidates from *C. mubango* and *C. haumanianus* (**1**–**5**). In blue: compounds isolated from Congolese *C. sylvaticus* (**6**–**8**). In black: selected compounds similar to **6**–**8** isolated from *C. mubango*, *haumanianus*, and *sylvaticus* (**9**–**15**). In green: compounds isolated from *C. laui*.

We initiated a phytochemical analysis of the DR Congo *Croton* species and selected three species *C. mubango*, *C. haumanianus*, and *C. sylvaticus* from the 22 species previously reported in the Congolese regions. These species were selected for their accessibility, well-established taxonomic characteristics, and historical use in folk medicine [4]. Previous studies with our collaborators on *C. mubango* and *C. haumanianus* resulted in the isolation of anticancer diterpenoids, compounds **1**–**5** (Figure 4) [9,10]. While several studies have extensively investigated bioactive compounds from *C. mubango* and *C. haumanianus* [9,10], the chemical profile of the DR Congo *C. sylvaticus* remains as yet unstudied. Given its historical medicinal use and potential as a source of novel bioactive compounds, there is a need for comprehensive phytochemical studies of this species. A dual approach was used to investigate *C. sylvaticus* metabolome. First, we applied classical chemical screening techniques to confirm the presence of major secondary metabolite classes. Second, the dereplication process using ^13^C NMR analysis facilitated by MixONat software enabled the rapid identification of key metabolite classes. Dereplication provides insights into the structural diversity of plant species. This systematic analysis provides a foundation for the isolation and characterization of bioactive compounds from *C. sylvaticus*.

Based on these structures, 25 analogous terpenoids previously isolated from two Congolese species, *C. mubango* and *C. haumanianus*, including reported anticancer compounds (**1**–**5**) along with structurally related compounds (**9**–**15**), were selected for molecular docking and SAR studies against HAR and HIF-1α receptors.

### 2.4. Docking and SAR Studies of Selected Terpenoids (***1***–***15***)

A series of terpenoids (**1**–**15**) from the Congolese *Croton* species was screened through docking studies with known anticancer agents (Metribolone, REF 1 [28]; clioquinol, REF 2 [33] and paclitaxel, REF 3 [34]) and five comparator molecules (**16**–**20**) were included for benchmarking. Compounds **16**–**20** are terpenoid derivatives recently isolated from *Croton laui* as anticancer [22]. The results were analyzed to correlate their structures and activities (Figure 4).

**Figure 5:**
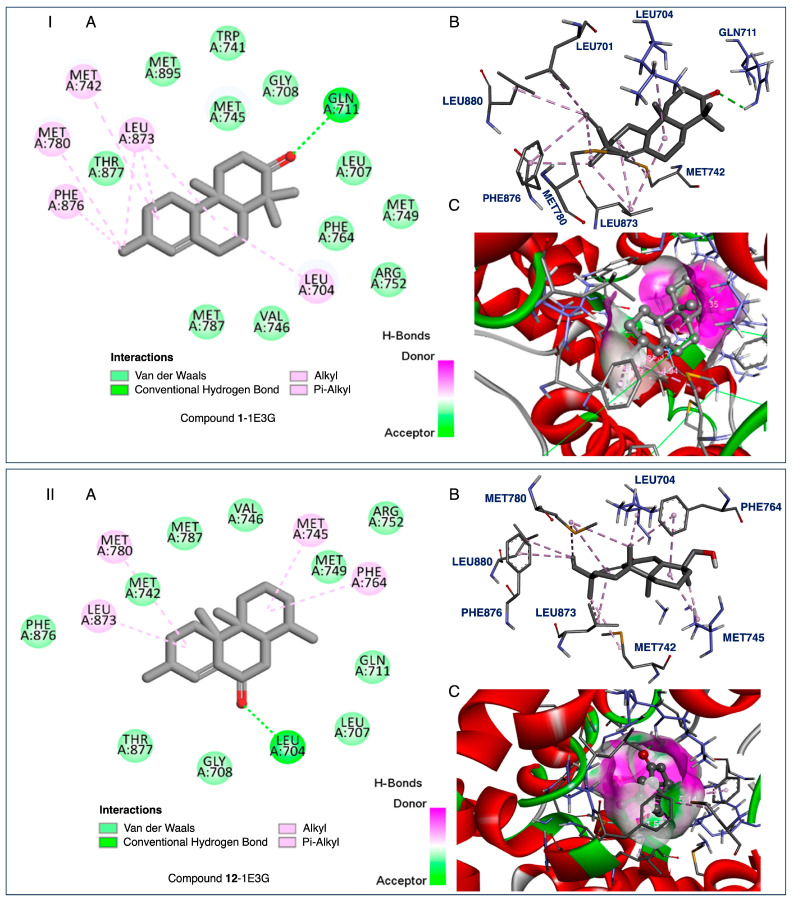
I. Compound **1**-1E3G and II. Compound **12**-1E3G. (**A**) Two-dimensional (2D) interaction diagrams showing the binding profiles of the ligands with key amino acid residues in the receptor’s active site. Interaction types are represented as follows: van der Waals interactions (light green), conventional hydrogen bonds (dark green dashed lines), and hydrophobic interactions including alkyl and π–alkyl interactions (light pink dashed lines). (**B**) Three-dimensional (3D) stick representations of the ligand–receptor complexes, displaying the orientation of the ligands within the active site and the interacting residues. (**C**) Three-dimensional (3D) surface representations of the receptor binding pocket, with the bound ligand shown in the center. The molecular surface is color-coded to indicate hydrogen bond acceptors (green) and donors (purple). These visualizations illustrate the key interactions for designing new inhibitors based on the chemical properties of the active site.

The tested compounds exhibited activities ranging from −7.7 to −14.6 kcal/mol against the HAR protein target, whereas the reference ligand showed a binding activity of −11.7 kcal/mol. Compound **1** showed the strongest binding affinity (Figure 5, −7.7 kcal/mol), and could be considered potentially robust. For the HIF-1α protein target, most of the tested terpenoids demonstrated binding affinities higher than the reference (−6.4 kcal/mol). Compound **9** exhibited the most potent affinity (−9.1 kcal/mol). Based on these results, compound **9** could be considered a good lead candidate for targeting the HIF-1α-related cancer pathways. Comparing the two protein targets, the chosen series of terpenoids generally showed stronger binding to HIF-1α than to HAR.

**Figure 6:**
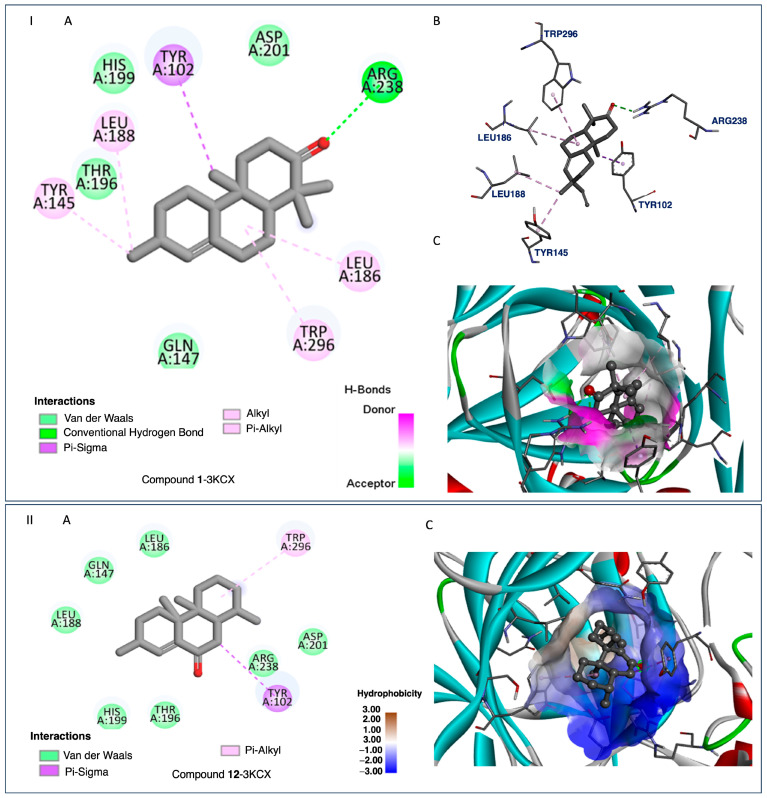
I. Compound **1**-3KCX and II. Compound **12**-3KCX. (**A**) Two-dimensional (2D) interaction diagrams depicting the molecular interactions between each ligand and key amino acid residues within the receptor’s active site. The interaction types are color-coded as follows: van der Waals interactions (light green), conventional hydrogen bonds (dark green dashed lines), π–sigma interactions (purple dashed lines), and hydrophobic contacts including alkyl and π–alkyl interactions (light pink dashed lines). (**B**) Three-dimensional (3D) stick representations of the ligand–receptor complexes, showing spatial conformations and key interacting residues. (**C**) Surface representations of the receptor binding pocket with the ligands embedded. The surface is color-coded to represent hydrogen bond donors (purple) and acceptors (green) for Compound **1**, and hydrophobicity mapping for Compound **12** (scale: brown = hydrophobic, blue = hydrophilic). These visualizations highlight the critical interactions that are useful for designing new inhibitors based on the chemical characteristics of the active site.

Considering compounds with docking score differences ≤ 1 kcal/mol across both receptors as having comparable affinities, compounds **1**, **2**, **7**, **8**, **10**, and **12** exhibited similar binding potentials for both HAR and HIF-1α. Compound **1** exhibited strong binding to both HAR (Figure 5, −7.7 kcal/mol) and HIF-1α (Figure 6, −8.4 kcal/mol), closely approaching the performance of the reference drugs. Compound **12** demonstrated consistent dual-target activity with ΔG values of −7.4 kcal/mol (HAR) and −8.4 kcal/mol (HIF-1α) (Figure 5 and Figure 6).

A structure–activity relationship analysis revealed that molecules with reduced binding affinities have greater molecular weights (e.g., compounds **4,** Mw 546.8, and **5**, Mw 544.9, Table 1) and elevated logP values of 8.7 and 10.1 (Table 1). The two compounds share an aliphatic side chain and possess more rotatable bonds (16 each). This could contribute to unfavorable interactions with the binding active site.

Cyclic structures and methyl branches likely confer increased hydrophobicity and spatial occupation, which are responsible for the strong binding activity.

Another key structural feature could be the number of hydrogen bond acceptors and donors. Compound **2** had three acceptors and three donors, demonstrating moderate to strong binding across both targets.

An analysis of the relationship between the binding affinities to the two protein targets suggests that compounds binding effectively to one target may also show some favorable affinities for the other, but the correlation is not strong enough to generalize this trend without further validation.

Compound **9**, which displayed a strong preference for HIF-1α, exhibited poor solubility. To overcome this drawback, it should undergo structural modifications to enhance its solubility while preserving potent target binding. Additionally, compounds demonstrating comparable binding affinities to the reference compound across both targets (e.g., compound **2**) could serve as potential lead compounds in the development of dual-target anticancer drugs.

Docking simulations rely on a rigid protein structure and may not fully capture the dynamic nature of protein–ligand interactions in vivo. The scoring functions used in docking are approximate and may not accurately rank the binding affinities.

These limitations should be acknowledged. Binding assays and in vitro studies are essential to confirm these findings and to establish a clear link between docking predictions and biological activity. The selected receptors are known to regulate important oncogenic pathways, and strong binding affinities suggest potential inhibitory effects. Thus, these interactions warrant further validation in experimental models to confirm their anticancer potential.

The key structure features observed guide the design of optimized terpenoids: moderate molecular weight, manageable structural cycle size, and logP values between three and five. To promote favorable interactions within the protein-binding site, the presence of two to three hydrogen bond donors and acceptors is desired. Docking studies have provided valuable insights into the potential of terpenoids as anticancer agents. Experimental validation and optimization of the identified lead compounds are necessary to translate these findings into clinical applications.

**Figure 7:**
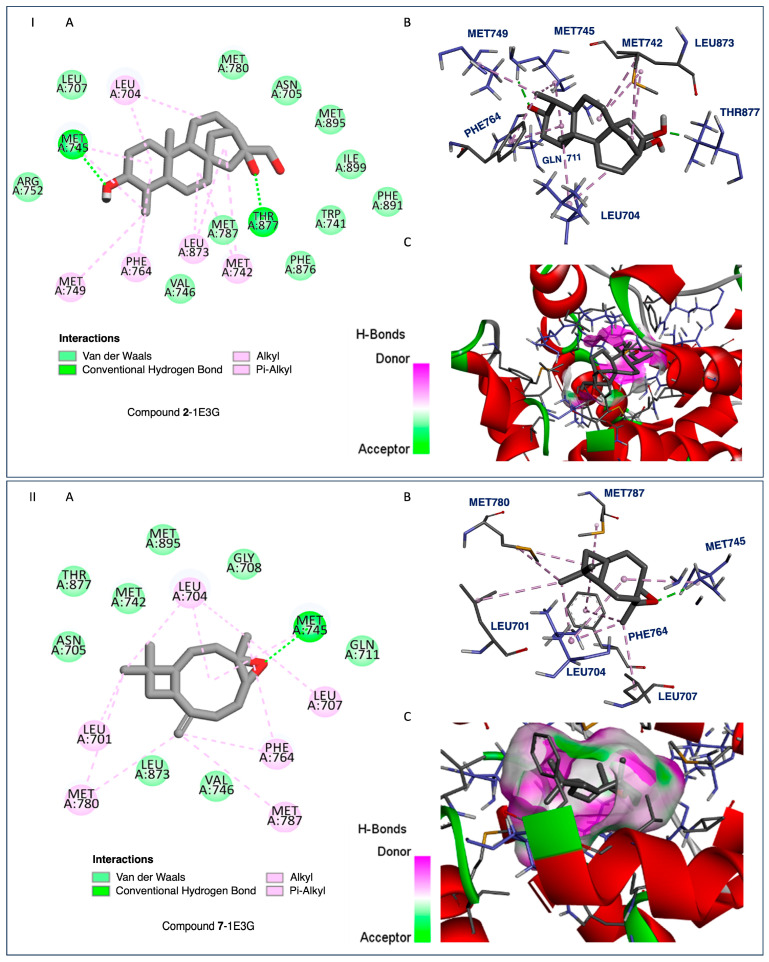
I. Compound **2**-1E3G, and II. Compound **7**-1E3G. (**A**) Two-dimensional (2D) interaction diagrams showing the molecular interactions between the ligand and key residues in the receptor binding site. Interaction types are color-coded: van der Waals interactions (light green), conventional hydrogen bonds (dark green dashed lines), and hydrophobic interactions including alkyl and π–alkyl interactions (light pink dashed lines). (**B**) Three-dimensional (3D) representations of the ligand–receptor complexes in stick format, depicting spatial orientation and interacting residues. (**C**) Surface views of the receptor binding pocket with embedded ligands. The receptor surface is color-coded to represent hydrogen bond acceptors (green) and donors (purple). These visualizations highlight key interactions, which are crucial for designing new inhibitors based on the chemical properties of the active site.

**Figure 8:**
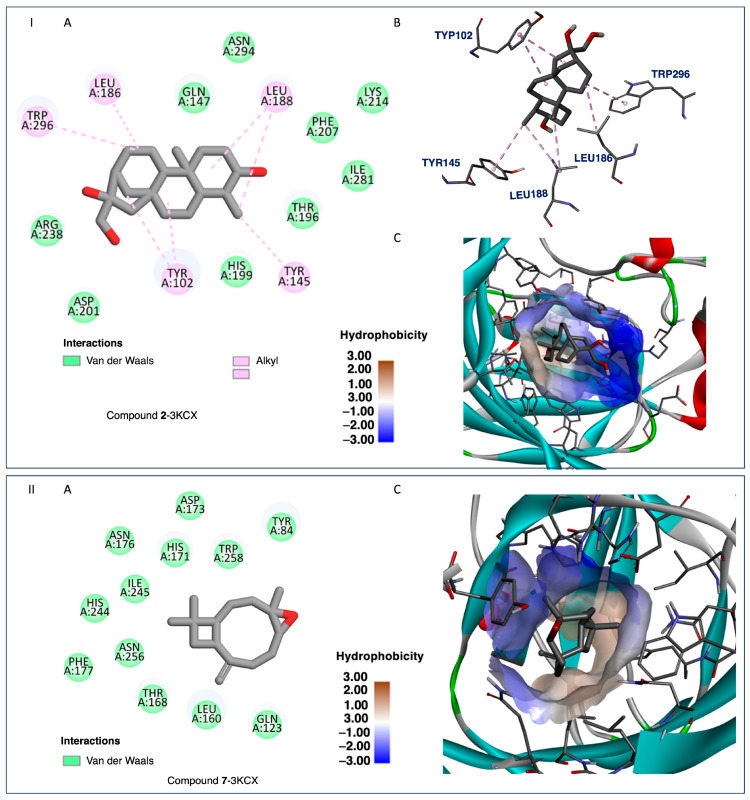
I. Compound **2**-3KCX and II. Compound **7**-3KCX. (**A**) Two-dimensional interaction diagrams showing the binding interactions between each ligand and key amino acid residues within the receptor’s active site. Van der Waals interactions are indicated in light green, and alkyl interactions are shown in light purple. (**B**) Three-dimensional representations of the docked ligands within the receptor pocket, with interacting residues labeled, and alkyl interactions represented by dashed purple lines. (**C**) Hydrophobic surface maps of the binding site, overlaid with the ligand pose. The color gradient represents hydrophobicity, from blue (hydrophilic) to brown (hydrophobic) regions. These visualizations highlight both the molecular interactions and the hydrophobicity of the receptor’s active site, providing insights into designing more efficient inhibitors.

The ADMET analysis of the tested compounds indicated that compounds **1**, **2**, **3**, and **12** had favorable drug-likeness properties, and showed their potential as dual-target anticancer agents. Compound **1** displayed considerable dual-target affinity (−7.7 and −8.4 kcal/mol) with excellent absorption (96.5%) and BBB permeability (0.645), but showed hERG II inhibition, requiring further cardiac safety evaluation. Compound **2**, with a combination of good binding (−7.3 and −8.3 kcal/mol, Figure 7 and Figure 8), best solubility (−3.77), and high absorption (93.2%), presented no predicted toxicity marking its druggable property. Compound **3** exhibited the highest Caco-2 permeability (1.68) and good intestinal absorption (96.0%), supporting passive diffusion and oral bioavailability. Although compound **3** does not exhibit strong binding activities to both proteins, it is structurally close to compound 2 suggesting that the presence of a hydroxyl group at position 3 is critical for binding affinities. Compound **12** presented strong binding affinities (−7.4 kcal/mol for HAR and −8.4 kcal/mol for HIF-1α; Figure 5 and Figure 6) and high human intestinal absorption (97.6%), and had no predicted toxicity, as well as no AMES or hERG inhibition. The completed ADMET results are included in the Supporting Information. It is worth noting that these four compounds are well balanced between their biological efficacy, favorable pharmacokinetics, and safety, justifying their selection for further in vitro testing as potential broad-spectrum anticancer agents.

It is worth noting that, despite its strong binding affinities to both targets (Figure 7 and Figure 8), compound **7**’s ADMEs analysis predicted **7** to inhibit both CYP2C19 and CYP2C9, two isoenzymes responsible for the metabolism of a variety of therapeutic agents; such inhibition could potentially lead to elevated plasma levels and toxicity of co-administered drugs. On the other hand, compounds **1**, **2**, **3**, and **12** presented minimal interaction with cytochrome P450 (CYP) enzymes. Compounds **2**, **3**, and **12** showed no inhibitory activity toward any major CYP enzymes, including CYP3A4, CYP1A2, CYP2C19, and CYP2C9, indicating a low potential for adverse metabolic interactions, and suggesting they are unlikely to interfere with the metabolism of co-administered drugs.

The isolated compounds from *C. sylvaticus* exhibited a diversity in their structure. Key structure features include a bulky structure for compound **6**, an epoxide moiety in compound **7** (which could contribute to strong bonding with active sites), and an aliphatic chain and hydroxyl group in compound **8** (which could favor interactions with membrane lipids and proteins). Their interactions with the targeted proteins are further explained in the Supporting Information, and depicted in Figure 5, Figure 6, Figure 7 and Figure 8 [Supporting Information SM-1 and SM-2].

### 2.5. Limitations, Strengths, and Future Prospects

The DR Congo is harboring a vast biodiversity of plants used as food as well as medicine, yet many of these resources remain unexplored. Approximately 22 species of *Croton* have been previously reported from the DR Congo. These plant species have pharmacological potential that could be attributed to diterpenoids, which are the predominant reported secondary metabolites for the *Croton* species.

In our study, we investigated a rapid approach that enabled the identification of a broad range of chemical components; however, it did not provide the comprehensive collection of isolated compounds for further structural and bioactivity studies. Compounds dereplicated with scores between 1 and 0.83, which have not been previously reported in *C. sylvaticus*, require targeted isolation efforts to assess their structure–activity relationships more effectively.

The technique used to analyze the metabolome permitted rapid dereplication. However, further refinement is required to provide a detailed view of the metabolites present in these species. Additionally, improving the sensitivity and resolution of NMR data could enable the identification of low-abundance metabolites.

Notably, the scope of our study was limited to only three *Croton* species. This restricts the generalization of our findings across the genus. It will be interesting to expand these studies to include a broader range of species from the DR Congo. This step was necessary to fully capture the chemical diversity within the genus *Croton*. An advantage of this approach could be the identification of species with particularly high concentrations of bioactive compounds.

Finally, dereplication techniques have been proven to be powerful tools for expanding the number of identified compounds. However, more advanced bioactivity-guided fractionation to link specific metabolites to their therapeutic effects is still required. This will help to identify and prioritize lead compounds for further in vitro and in vivo pharmacological testing.

## 3. Material and Methods

### 3.1. Material and Chemicals

Nuclear magnetic resonance (NMR) spectra were acquired using a Bruker Avance III 400 MHz spectrometer equipped with a broadband probe. The samples were dissolved in CDCl_3_ (30 mg of extract was dissolved in 600 μL CDCl_3_) (99.8%, Cambridge Isotope Laboratories, Inc., Tewksbury, MA, USA) containing tetramethylsilane (TMS) as an internal reference. All proton chemical shifts are reported in parts per million (ppm) relative to the TMS peak at δ_H_ 0 ppm. Carbon chemical shifts are also reported in parts per million (ppm) relative to the solvent peaks at δ_C_ 77.16 ppm. Phase and baseline corrections of the spectra were performed automatically using TopSpin software (version 4.1.4, Bruker BioSpin, Rheinstetten, Germany). A minimum intensity threshold was then used to collect positive ^13^C-NMR signals. DEPT experiments were aligned with the ^13^C spectra using a given δ_C_. For the ^13^C-NMR (100 MHz) spectra, a WALTZ-16 decoupling sequence was employed with an acquisition time of 1.29 s (32,768 complex data points), relaxation delay of 2 s, and a total of 10,000 scans. A 1 Hz exponential line-broadening filter was applied to each free induction decay (FID) before Fourier transformation. Manual phasing and baseline correction were also performed. The DEPT experiments were aligned with the ^13^C spectra using the δ_C_ reference values from the sample.

Analytical thin-layer chromatography (TLC) was performed on silica gel 60 F254 plates (0.20 mm thickness) from Macherey-Nagel GmbH & Co. KG, Neumann-Neander-Unk. 6-8, 52355 Dueren, Germany. Column chromatography (CC) separations were carried out using silica gel (Merck 773429, particle size < 0.06 mm, column diameter 1–3 cm).

Chemicals: Analytical grade solvents were purchased and distilled prior to use. Methanol was obtained from Saarchem, Gauteng, South Africa, and dichloromethane was obtained from Protea Chemicals, Gauteng, South Africa.

Plant Material: The trunk bark of *C. sylvaticus* was collected from the Luki reserve Kongo Central, Democratic Republic of Congo, in May 2019. The plant material was authenticated in comparison with herbarium specimens (Voucher numbers P. Compère 1647 and R. Devred 392) housed in the Herbarium of the Institut National pour l’Etude et la Recherche Agronomiques (INERA), Faculty of Sciences and Technologies, University of Kinshasa. The bark was air-dried at room temperature in the Laboratory of Organic Analysis and Synthesis of Kinshasa (LASORG-K) for a month under conditions to prevent contamination and degradation. The dried bark was then ground into a fine powder using an electric grinder (Blender B-592, Butterfly). The resulting powder was used for subsequent phytochemical analysis.

### 3.2. Methods

#### 3.2.1. Extraction

Bark powder (300 g) was macerated in 3 L of dichloromethane (DCM) for 48 h at room temperature. The mixture was then filtered using Whatman No. 1 filter paper, and the solvent was evaporated under reduced pressure at 40 °C using a Büchi RE 120 rotary evaporator, yielding 5.2 g of dichloromethane extract (EDCM, extract of dichloromethane maceration).

Under similar conditions, the residual marc from the first extraction was macerated in 2 L methanol (MeOH) for 48 h. After filtration, the solvent was evaporated under reduced pressure, yielding 10.3 g of methanolic extract (EME). Residual marc was used to produce the aqueous extract under similar conditions (8.3 g) [Supporting Information: Figure S1].

#### 3.2.2. Phytochemical Screening Test

Phytochemical screening of the plant extracts was conducted using standard qualitative tests to identify major classes of secondary metabolites, as described in the literature. Alkaloids, flavonoids, steroids, tannins, phenols, saponins, triterpenoids, and anthocyanins were detected through specific reactions [35,36]. Observations include characteristic color changes or precipitate formation to confirm the presence of these compounds. All tests were performed in triplicates to ensure accuracy and reproducibility.

#### 3.2.3. Metabolome Profiling

For this study, two databases: the Lamiaceae-DB1 and Euphorbiaceae-DB2 were used. The *Croton* metabolites reported in the Euphorbiaceae are contained in those databases, and the initial dereplication search began with the Lamiaceae natural product (NP) database. This database was used for ^13^C NMR-based dereplication. This database was prepared previously, as described in [37]. The Lamiaceae DB-1 contains 958 NPs and Euphorbiaceae-DB2 contains 6286 NPs, readily available in the appropriate format to predict the δ_C_ of methyl, methylene, methine, or quaternary carbons using MixONat software. The database could be adapted and used to investigate NPs from the *Croton* species since it contains NPs with similar basic skeletons (terpenoids).

MixONat, a freely available software at https://sourceforge.net/projects/mixonat/ (accessed on 23 November 2024) developed by the SONAS lab at Université Angers (Angers, France), enables dereplication of natural product mixtures using ^13^C NMR. The experimental carbon data (^13^C and DEPT-135) and intensity values were exported to a reference .csv file using Microsoft Excel (Microsoft Office, Redmond, WA, USA), and used as input for MixONat. The putative natural products (NPs) in the extracts were ranked by the software with a score ranging from 0 to 1 based on the number of matching experimental carbon signals obtained from the extract compared to the predicted carbon values reported in the literature. A score above 0.70 was considered acceptable for putative identification. The NPs with the highest score were compared with the literature data of the reported *Croton* NPs [Supporting Information: Table S1–S4, Figure S2–S51].

#### 3.2.4. Purification and Structure Elucidation of Major Constituents from *C. sylvaticus*

The compounds described in this study were isolated using a silica gel chromatographic column (Merck 773429; particle size < 0.06 mm). For separation, a gradient elution system was used, starting with n-hexane/DCM mixtures (1:1 and 1:3) to gradually increase the polarity, followed by pure dichloromethane (DCM) under isocratic conditions. The purification process was monitored using thin-layer chromatography (TLC) on silica gel F254 plates using an eluent system of [DCM 100%, DCM-MeOH: 99-1 (1%), DCM-MeOH: 97-3 (3%), DCM-MeOH 95-5 (5%)]. TLC spots were visualized under UV light at 254 and 366 nm, followed by staining with anisaldehyde/H_2_SO_4_/methanol (1:2:97). The plates were heated to 110 °C to develop distinct colorations, aiding compound identification based on the retention factors (Rf values) and visual characteristics.

The isolated compounds were obtained in yields of 0.19 g (compound **6**), 0.23 g (compound **7**), and 0.13 g (compound **8**), with purity levels exceeding 95%, as determined by analytical TLC and NMR spectroscopy. Spectral data of these compounds (δ_H_ and δ_C_ values) were compared with previously reported values to identify known compounds. [Supporting Information, Tables S6–S8; Figures S102–S110].

#### 3.2.5. Molecular Docking

Two structurally characterized receptors—the human androgen receptor (HAR, PDB ID: 1E3G) and hypoxia-inducible factor 1-alpha (HIF-1α, PDB ID: 3KCX)—were retrieved from the Protein Data Bank (retrieved date 20 November 2024). These structures were selected because of their high resolution and relevance to the studied binding interactions. Visualization of the binding domains and identification of key amino acid residues in the binding pocket were carried out using the Chimera software (v 1.18). The inhibitor-binding site was analyzed based on previously reported amino acids residues [38,39].

Hydrogen atoms were added to the proteins to correct for ionization and tautomeric states using amino acids residues, ensuring accurate charge and geometry optimization for docking. Water molecules that were not involved in binding site interactions and ligand–receptor complexes were removed.

For incomplete side chains, the Dunbrack rotamer library was used to restore side-chain conformations. The results were crosschecked to ensure consistency. Using the AMBER 14SB force field, the proteins were subjected to energy minimization, and the AM1-BCC model was applied to the other residues. The minimization involved 200 steps at an RMS gradient of 0.02, and the post-minimization stability was verified.

The two-dimensional (2D) chemical structures of the compounds were initially drawn using ChemDraw (professional v 19.0.0.22) and subsequently converted to three-dimensional (3D) structures using Chem3D (v 19.0.0.22). These 3D structures were then subjected to energy minimization to obtain stable conformations. The minimized structures were saved in the .mol2 format and subsequently converted to the .pdbqt format using Open Babel within the PyRx (v 0.8) docking tool, in preparation for molecular docking studies. The optimized protein structures were saved in the pdbqt format and imported into PyRx.

Docking simulations were carried out using AutoDock Vina. The grid box was centered around the identified binding sites, with the grid center coordinates set to (10, 25, 11) for 1E3G and (−22, 29, 8) for 3KCX, respectively. The grid box dimensions were defined as 20 × 20 × 20 Å in each direction (x, y, z). Docking results were evaluated based on the predicted binding affinities (expressed in kcal/mol) and the comparison of docked ligand poses with known crystallographic binding modes.

#### 3.2.6. Docking Strategies

AutoDock Vina and PyRx were used to generate the bioactive binding positions of the ligands within the active sites of both HIF-1α and HAR. The active sites were defined using the protein coordinates of the bound ligands, 3KCX and 1E3G. Docking simulations were conducted based on the Lamarckian genetic algorithm, following the standard protocol described by Morris et al. (1998) [40] and Selvaraj et al. (2019) [41]. Grid maps for docking were centered on the target proteins following the methods described by Anshika et al. (2017) [38] and Vidya et al. (2019) [39].

Accelrys Discovery Studio 2019 software was employed to model the non-bonded polar and hydrophobic interactions within the inhibitor site of both proteins. Hit molecules that interact with the key amino acids in the active site may act as potent antagonists of 3KCX and 1E3G.

To validate the docking results, reference ligands were re-docked into the appropriate protein cavities, with re-docking considered to be successful if the root mean square deviation (RMSD) was ≤2.0 Å [40,41,42,43]. In this study, we achieved an RMSD of 0.5 and 0.4 Å by superposing ligand positions within the binding sites of 1E3G and 3KCX [Figure 5, Figure 6, Figure 7 and Figure 8, Supporting Information: Docking studies of terpenoids **6**–**8**, Table S9 and **8**, **10**, **11**, and **17**, Table S11; Figures S112–S114].

#### 3.2.7. ADMET Profiling

The absorption, distribution, metabolism, excretion, and toxicity (ADMET) profiles of 15 ligands were predicted using in silico bioinformatics tools to evaluate their potential as drug candidates. Key drug-likeness properties, including molecular weight (MW), bioavailability score (BioS), number of rotatable bonds (nRB), hydrogen bond acceptors (HBA), hydrogen bond donors (HBD), molar refractivity (MR), and topological polar surface area (TPSA), were calculated using SwissADME (http://www.swissadme.ch/, accessed on 20 November 2024). Toxicity and pharmacokinetic properties, such as gastrointestinal absorption, blood–brain barrier penetration, and potential hepatotoxicity, were predicted using the pkCSM web server (https://biosig.lab.uq.edu.au/pkcsm/prediction accessed on 20 November 2024).

ADMET profiling provides essential information on all the critical factors that influence the pharmacokinetics and pharmacodynamics of a drug.

## 4. Conclusions

We carried out a phytochemical analysis of the *Croton* species from the DR Congo. This investigation constitutes a notable contribution to the comprehension of the phytochemical composition and pharmacological potential of three Congolese *Croton* species: *C. mubango*, *C. haumanianus*, and *C. sylvaticus*. Using classical phytochemical screening in combination with dereplication techniques, a large number of secondary metabolites have been successfully identified, with terpenoids as the major class. The dereplication approach proved highly effective. This method enabled rapid identification of more than 376 terpenoids in the DB1 and 391 terpenoids in the DB2. Specifically, diterpenoids, sesquiterpenoids, monoterpenoids, triterpenoids, and steroids, together with other minor secondary metabolites, such as saponins, amino acid, and fatty acids, were identified.

The findings indicate a unique chemical profile for *C. sylvaticus*. This study revealed previously unreported compounds in the species, such as acetyl aleuritolic acid, caryophyllene oxide, and phytol. The bioactivities of anti-cancer terpenoids from *C. mubango* and *C. haumanianus* were evaluated using molecular docking techniques. Among the tested compounds, **1**, **2**, **3**, and **12** were identified as having strong-to-moderate predicted binding affinities to both protein targets, along with favorable drug-like properties according to the ADMET analysis. Structure–activity relationship (SAR) analysis revealed that molecular weight, hydrophobicity (logP values), and the distribution of hydrogen bond donors and acceptors play key roles in binding affinity.

This study indicates the anti-cancer therapeutic potential of the Congolese *Croton* species. Their composition could be investigated in the context of anticancer drug discovery.

Overall, this investigation demonstrates the importance of exploring under-studied plant species belonging to the biodiverse regions of the DR Congo. Such studies could contribute to novel bioactive compounds and sustain drug discovery initiatives.

## Figures and Tables

**Table 1:** Binding Affinity Energy and Physicochemical Properties of Compounds for Protein Targets 1E3G and 3KCX.

Compounds	∆G_binding_ (kcal/mol)		(ΔG Difference kcal/mol)	Molecular Weight	logP	Rotatable Bonds	H-Bond Acceptors	H-Bond Donors	Surface Area (Å^2^)	Water Solubility
	HAR (1E3G) Prostate Cancer	HIF-1α (3KCX) Breast Cancer								
**1**	−7.7	−8.4	0.7	286.4	5.3	1	1	0	129.4	−6.1
**2**	−7.3	−8.3	1	308.4	2.7	1	3	3	133.6	−3.7
**3**	−5.9	−8.0	2.1	306.4	4.1	1	2	2	135.2	−4.7
**4**	−1.5	−7.4	5.9	546.8	8.7	16	4	2	239.9	−5.8
**5**	−1.6	−7.4	5.8	544.9	10.1	16	3	1	241.5	−5.5
**6**	−6.6	−8.1	1.5	484.7	7.4	2	3	1	212.2	−4.3
**7**	−7.2	−7.2	0	220.3	3.9	0	1	0	99.2	−4.3
**8**	−6.2	−6.6	0.4	296.5	6.3	13	1	1	133.7	−7.5
**9**	−6.1	−9.1	3	426.7	8.0	1	1	1	192.3	−6
**10**	−6.7	−7.1	0.4	220.3	4.2	0	1	0	99.5	−4.1
**11**	−6.8	−7.8	1	286.4	5.3	2	1	0	129.4	−6.1
**12**	−7.4	−8.4	1	316.4	4.6	2	2	1	140.5	−5.1
**13**	−5.8	−8.0	2.2	302.4	5.2	2	1	1	134.2	−4.3
**14**	−5.9	−8.2	2.3	288.4	5.1	2	1	1	130.0	−5.7
**15**	−6.6	−8.7	2.1	318.4	5.1	4	2	1	139.0	−4.1
**REF 1**	−11.7	−8.8	2.9	284.4	3.7	0	2	1	126.2	−4.1
**REF 2**	−6.5	−6.4	0.1	305.5	3.2	0	2	1	93.7	−3.4
**REF 3**	−6.1	−9.8	3.7	853.9	3.7	10	14	4	357.9	−3.2
**16**	−7.2	−8.3	1.1	304.5	4.4	1	2	1	134.9	−4.3
**17**	−7.6	−8.0	0.4	300.4	4.6	2	2	0	133.6	−5.5
**18**	−5.3	−8.2	2.9	332.5	5.3	3	2	1	147.9	−4.8
**19**	−5.6	−8.2	2.6	332.5	5.3	4	2	1	147.9	−5.3
**20**	−5.8	−8.2	2.4	302.5	4.3	2	2	1	134.2	−5.2

## Data Availability

Data are contained within the article and the Supplementary Files.

## Supplementary Materials

The following supporting information can be downloaded at: https://www.mdpi.com/article/10.3390/ijms26094305/s1.
