# Supplementary material for: Rapid Dereplication of Trunk Bark Constituents of Croton sylvaticus and Molecular Docking of Terpenoids from Three Congolese Croton Species
Source: Int J Mol Sci. 2025 May 1;26(9):4305. doi: 10.3390/ijms26094305 (PMC12072447; doi:10.3390/ijms26094305)
Supplement: Supplementary file 1 [file ijms-26-04305-s001.zip › SM-1-Supplementary Material-1 proofreading .pdf]

Supplementary Material 1

Rapid Dereplication of Trunk Bark Constituents of *Croton sylvaticus* and Molecular Docking of Terpenoids from Three Congolese *Croton* Species

Bienvenu Kamalandua Mvingu [1], Tienabe Nsiama [1], Obed Nsemi Kanga [1], Kalulu Muzele Taba [1], Jason Thambwe Kilembe [1], Jean-Noël Kanyinda Mputu [1,2], Sarah Garifo [2], Céline Henoumont [2], Dya Fita Dibwe [1,3,*], Blaise Mavinga Mbala [1] and Sophie Laurent [2]

[1] Université de Kinshasa, Faculté des Sciences et Technologies, Mention Chimie et Industrie, B.P. 190, Kin XI, RD Congo.
[2] NMR and Molecular Imaging Laboratory, General, Organic and Biomedical Chemistry Unit, University of Mons, 19 Avenue Maistriau, 7000 Mons, Belgium.
[3] Faculty of Health Sciences, Hokkaido University, Kita-12, Nishi-5, Kita-Ku, Sapporo 060-0812, Japan;

*Corresponding author: dibwedf@hs.hokudai.ac.jp or eddy.dibwe@unikin.ac.cd (D.F.D)

Supplementary Material 1

Table of contents:

**Materials and Methods**	P7
1. Chemicals and instruments	P7
2. Extraction	P7
3. Phytochemicals test	P7
4. Purification and isolation of the compounds	P8
**Table S1.** Types of compounds, numbers and rank in MixONat	P9
**Table S2.** Skeletons of compounds detected and their rank in MixONat	P11
**Table S3.** Scoring and ranking of isolated **6–8** dereplication compounds and their similar compounds in MixoNat	P14
**Table S4.** Scores et Rank compounds in MixONat	P15
**Figure S1.** Scheme illustrates the extraction process	P16
**Compound Data base 1**	P17
**Figure S2**. Dereplication analysis from MixONat, structure of dereplicated compounds: Rank **1-10**.	P17
**Figure S3**. Dereplication analysis from MixONat, structure of dereplicated compounds: Rank **11-20**	P18
**Figure S4**. Dereplication analysis from MixONat, structure of dereplicated compounds: Rank **21-30**.	P19
**Figure S5**. Dereplication analysis from MixONat, structure of dereplicated compounds: Rank **31-40**	P20
**Figure S6**. Dereplication analysis from MixONat, structure of dereplicated compounds: Rank **41-50**.	P21
**Figure S7.** Dereplication analysis from MixONat, structure of dereplicated compounds: Rank **51-60**.	P22
**Figure S8.** Dereplication analysis from MixONat, structure of dereplicated compounds: Rank **61-70**.	P23
**Figure S9.** Dereplication analysis from MixONat, structure of dereplicated compounds: Rank **71-80**.	P24
**Figure S10**. Dereplication analysis from MixONat, structure of dereplicated compounds: Rank **81-90**.	P25
**Figure S11**: Dereplication analysis from MixONat, structure of dereplicated compounds: Rank **91-100**.	P26
**Figure S12**: Dereplication analysis from MixONat, structure of dereplicated compounds: Rank **101-110**.	P27
**Figure S13**: Dereplication analysis from MixONat, structure of dereplicated compounds: Rank **111-120**.	P28
**Figure S14**: Dereplication analysis from MixONat, structure of dereplicated compounds: Rank **121-130**.	P29

Figure S15: Dereplication analysis from MixONat, structure of dereplicated compounds: Rank **131-140**. P30
Figure S16: Dereplication analysis from MixONat, structure of dereplicated compounds: Rank **141-150** P31
Figure S17: Dereplication analysis from MixONat, structure of dereplicated compounds: Rank **151-160**. P32
Figure S18: Dereplication analysis from MixONat, structure of dereplicated compounds: Rank **161-170** P33
Figure S19: Dereplication analysis from MixONat, structure of dereplicated compounds: Rank **171-180**. P34
Figure S20: Dereplication analysis from MixONat, structure of dereplicated compounds: Rank **181-190**. P35
Figure S21: Dereplication analysis from MixONat, structure of dereplicated compounds: Rank **191-200**. P36
Figure S22: Dereplication analysis from MixONat, structure of dereplicated compounds: Rank **201-210**. P37
Figure S23: Dereplication analysis from MixONat, structure of dereplicated compounds: Rank **211-220**. P38
Figure S24: Dereplication analysis from MixONat, structure of dereplicated compounds: Rank **221-230**. P39
Figure S25: Dereplication analysis from MixONat, structure of dereplicated compounds: Rank **231-240**. P40
Figure S26: Dereplication analysis from MixONat, structure of dereplicated compounds: Rank **241-250**. P41
Figure S27: Dereplication analysis from MixONat, structure of dereplicated compounds: Rank **251-260**. P42
Figure S28: Dereplication analysis from MixONat, structure of dereplicated compounds: Rank **261-270**. P43
Figure S29: Dereplication analysis from MixONat, structure of dereplicated compounds: Rank **271-280**. P44
Figure S30: Dereplication analysis from MixONat, structure of dereplicated compounds: Rank **281-290** P45
Figure S31: Dereplication analysis from MixONat, structure of dereplicated compounds: Rank **291-300**. P46
Figure S32: Dereplication analysis from MixONat, structure of dereplicated compounds: Rank **301-310** P47
Figure S33: Dereplication analysis from MixONat, structure of dereplicated compounds: Rank **311-320**. P48
Figure S34: Dereplication analysis from MixONat, structure of dereplicated compounds: Rank **321-330**. P49
Figure S35: Dereplication analysis from MixONat, structure of dereplicated compounds: Rank **331-340**. P50
Figure S36: Dereplication analysis from MixONat, structure of dereplicated compounds: Rank **341-350**. P51
Figure S37: Dereplication analysis from MixONat, structure of dereplicated compounds: Rank **351-360** P52
Figure S38: Dereplication analysis from MixONat, structure of dereplicated compounds: Rank **361-370**. P53
Figure S39: Dereplication analysis from MixONat, structure of dereplicated compounds: Rank **371-380**. P54
Figure S40: Dereplication analysis from MixONat, structure of dereplicated compounds: Rank **381-390**. P55
Figure S41: Dereplication analysis from MixONat, structure of dereplicated compounds: Rank **391-400** P56
Figure S42: Dereplication analysis from MixONat, structure of dereplicated compounds: Rank **401-410**. P57
Figure S43: Dereplication analysis from MixONat, structure of dereplicated compounds: Rank **411-420**. P58
Figure S44: Dereplication analysis from MixONat, structure of dereplicated compounds: Rank **421-430** P59
Figure S45: Dereplication analysis from MixONat, structure of dereplicated compounds: Rank **431-440**. P60

Figure S46: Dereplication analysis from MixONat, structure of dereplicated compounds: Rank **441-450**. P61
Figure S47: Dereplication analysis from MixONat, structure of dereplicated compounds: Rank **441-460**. P62
Figure S48: Dereplication analysis from MixONat, structure of dereplicated compounds: Rank **461-470** P63
Figure S49: Dereplication analysis from MixONat, structure of dereplicated compounds: Rank **471-480** P64
Figure S50: Dereplication analysis from MixONat, structure of dereplicated compounds: Rank **481-490**. P65
Figure S51: Dereplication analysis from MixONat, structure of dereplicated compounds: Rank **491-500**. P66
Compound Data base 2 P67
Figure S52: Dereplication analysis from MixONat, structure of dereplicated compounds: Rank **1-10**. P68
Figure S53: Dereplication analysis from MixONat, structure of dereplicated compounds: Rank **11-20**. P69
Figure S54: Dereplication analysis from MixONat, structure of dereplicated compounds: Rank **21-30**. P70
Figure S55: Dereplication analysis from MixONat, structure of dereplicated compounds: Rank **31-40**. P71
Figure S56: Dereplication analysis from MixONat, structure of dereplicated compounds: Rank **41-50**. P72
Figure S57: Dereplication analysis from MixONat, structure of dereplicated compounds: Rank **51-60**. P73
Figure S58: Dereplication analysis from MixONat, structure of dereplicated compounds: Rank **61-70**. P74
Figure S59: Dereplication analysis from MixONat, structure of dereplicated compounds: Rank **71-80**. P75
Figure S60: Dereplication analysis from MixONat, structure of dereplicated compounds: Rank **81-90**. P76
Figure S61: Dereplication analysis from MixONat, structure of dereplicated compounds: Rank **91-100**. P77
Figure S62: Dereplication analysis from MixONat, structure of dereplicated compounds: Rank **101-110**. P78
Figure S63: Dereplication analysis from MixONat, structure of dereplicated compounds: Rank **111-120**. P79
Figure S64: Dereplication analysis from MixONat, structure of dereplicated compounds: Rank **121-130**. P80
Figure S65: Dereplication analysis from MixONat, structure of dereplicated compounds: Rank **131-140**. P81
Figure S66: Dereplication analysis from MixONat, structure of dereplicated compounds: Rank **141-150**. P82
Figure S67: Dereplication analysis from MixONat, structure of dereplicated compounds: Rank **151-160**. P83
Figure S68: Dereplication analysis from MixONat, structure of dereplicated compounds: Rank **161-170**. P84
Figure S69: Dereplication analysis from MixONat, structure of dereplicated compounds: Rank **171-180**. P85
Figure S70: Dereplication analysis from MixONat, structure of dereplicated compounds: Rank **181-190**. P86
Figure S71: Dereplication analysis from MixONat, structure of dereplicated compounds: Rank **191-200**. P87
Figure S72: Dereplication analysis from MixONat, structure of dereplicated compounds: Rank **201-210**. P88
Figure S73: Dereplication analysis from MixONat, structure of dereplicated compounds: Rank **211-220**. P89
Figure S74: Dereplication analysis from MixONat, structure of dereplicated compounds: Rank **221-230**. P90
Figure S75: Dereplication analysis from MixONat, structure of dereplicated compounds: Rank **231-240**. P91
Figure S76: Dereplication analysis from MixONat, structure of dereplicated compounds: Rank **241-250**. P92
Figure S77: Dereplication analysis from MixONat, structure of dereplicated compounds: Rank **251-260**. P93

Figure S78: Dereplication analysis from MixONat, structure of dereplicated compounds: Rank **261-270**. P94
Figure S79: Dereplication analysis from MixONat, structure of dereplicated compounds: Rank **271-280**. P95
Figure S80: Dereplication analysis from MixONat, structure of dereplicated compounds: Rank **281-290**. P95
Figure S81: Dereplication analysis from MixONat, structure of dereplicated compounds: Rank **291-300**. P96
Figure S82: Dereplication analysis from MixONat, structure of dereplicated compounds: Rank **301-310**. P97
Figure S83: Dereplication analysis from MixONat, structure of dereplicated compounds: Rank **311-320**. P98
Figure S84: Dereplication analysis from MixONat, structure of dereplicated compounds: Rank **321-330**. P99
Figure S85: Dereplication analysis from MixONat, structure of dereplicated compounds: Rank **331-340**. P100
Figure S86: Dereplication analysis from MixONat, structure of dereplicated compounds: Rank **341-350**. P101
Figure S87: Dereplication analysis from MixONat, structure of dereplicated compounds: Rank **351-360**. P102
Figure S88: Dereplication analysis from MixONat, structure of dereplicated compounds: Rank **361-370**. P103
Figure S89: Dereplication analysis from MixONat, structure of dereplicated compounds: Rank **371-380**. P104
Figure S90: Dereplication analysis from MixONat, structure of dereplicated compounds: Rank **381-390**. P105
Figure S91: Dereplication analysis from MixONat, structure of dereplicated compounds: Rank **391-400**. P106
Figure S92: Dereplication analysis from MixONat, structure of dereplicated compounds: Rank **401-410**. P107
Figure S93: Dereplication analysis from MixONat, structure of dereplicated compounds: Rank **411-420**. P108
Figure S94: Dereplication analysis from MixONat, structure of dereplicated compounds: Rank **421-430**. P109
Figure S95: Dereplication analysis from MixONat, structure of dereplicated compounds: Rank **431-440**. P110
Figure S96: Dereplication analysis from MixONat, structure of dereplicated compounds: Rank **441-450**. P111
Figure S97: Dereplication analysis from MixONat, structure of dereplicated compounds: Rank **451-460**. P112
Figure S98: Dereplication analysis from MixONat, structure of dereplicated compounds: Rank **461-470**. P113
Figure S99: Dereplication analysis from MixONat, structure of dereplicated compounds: Rank **471-480**. P114
Figure S100: Dereplication analysis from MixONat, structure of dereplicated compounds: Rank **481-490**. P115
Figure S101: Dereplication analysis from MixONat, structure of dereplicated compounds: Rank **491-500**. P116
Figure S102. Scheme describes the fractionation and purification of the dichloromethane extract of *Croton sylvaticus*. P117
Figure S103. ^{1}H-NMR for compound **6** (DCM/CS) in $CDCl_3$ P118
Figure S104. ^{13}C-NMR for compound **6** (DCM/CS) in $CDCl_3$ P119
Figure S105. ^{1}H-NMR for compound **7** (DCM/CS) in $CDCl_3$ P120
Figure S106. ^{13}C-NMR for compound **7** (DCM/CS) in $CDCl_3$ P121
Figure S107. DEPT-135 for compound **7** (DCM/CS) in $CDCl_3$ P122

Figure S108. ^{1}H NMR for compound **8** (DCM/CS) in $CDCl_3$ P123

Figure S109. ^{13}C NMR for compound **8** (DCM/CS) in $CDCl_3$ P124

Figure S110. DEPT-135 for compound **8** (DCM/CS) in $CDCl_3$ P125

Table S5. ^{13}C-NMR values for compound Acetyl aleuritolic acid 6 (CS-BD-90) P126

Table S6. ^{13}C-NMR values for compound Caryophyllene oxide 7 (CS-BD-X-5-8) P127

Table S7. ^{13}C-*NMR* values for compound Trans-Phytol (3,7,11,15-tetramethylhexadec-2-en-1-ol) (CS-BD-E1) P128

Table S8. Compounds **1–15** used in docking study P129

Table 9. Binding Affinity and Physicochemical Properties of Compounds for Protein Targets 1E3G and 3KCX P132

Docking studies of terpenoids 6–8: Comparison with Ligand 6, Ligand 7 and Ligand 8 P132

Table S10: Comparison with Ligand 6, Ligand 7 and Ligand 8 P133

A comparative analysis of Compounds 1, 2, 7, and 12 against both androgen receptor (HAR, PDB ID: 1E3G) and hypoxia-inducible factor-1 alpha (HIF-1α, PDB ID: 3KCX) P134

Table S11: ADMET predictions P135

Figure S111. Compound **8**-1E3G and-3KCX: 2D and 3D image of the interaction between compound **8** with 1E3G and-3KCX receptor. P136

Figure S112. Compound **10**-1E3G and-3KCX: 2D and 3D image of the interaction between compound **10** with 1E3G and-3KCX receptor. P137

Figure S113. Compound **11**-1E3G and-3KCX: 2D and 3D image of the interaction between compound **11** with 1E3G and-3KCX receptor. P137

Figure S114. Compound **17**-1E3G and-3KCX: 2D and 3D image of the interaction between compound **17** with 1E3G and-3KCX receptor. P138

Materials and Methods

1. *Chemicals and instruments*

General Experimental Procedures: Buchi Re 120 rotary evaporator was using for evaporating solvents under reduced pressure at 40°C, Nuclear magnetic resonance (NMR) spectra were obtained using a 400MHz ou 100MHz Bruker AVANCE III NMR spectrometer (CDCl3 99,8%, Cambridge Isotope Labotories, Inc, USA), with tetramethylsilane (TMS) serving as an internal reference. Chemical shifts were reported as δ values in parts per million (ppm) and processed using Bruker NMR Academic Topspin software. Analytical thin-layer chromatography (TLC) was conducted on silica gel 60 F254 plates (0.20 mm thickness) from Machery-Nagel GmbH & Co.KG, Neumann-Neander-Str. 6-8. 52355, Germany. Colunm chromatography (CC) separations were carried using silica gel Merck 773429 (< 0,06 mm thickness, 1-3 cm diameter).

Chemicals: Methanol was purchased from Saarchem, Gauteng, South Africa and dichloromethan was purchased from Protea Chemicals, Gauteng, South Africa.

2. Extraction

Extractions were carried out using analytical-grade solvents obtained from Protea. Chemicals, Gauteng, South Africa, and purified by distillation in increasing polarity: first dichloromethane (DCM), followed by methanol (ME).

Bark powder (300 g) was macerated in 3 L DCM for 48 h at room temperature. The mixture was then filtered using Whatman No.1 filter paper, and the solvent was evaporated under reduced pressure at 40° C using a Buchi Re 120 rotary evaporator), yielding the dichloromethane extract (EDCM extract, 5.2 g).

Under similar conditions, the residual solid from the first extraction was macerated in 2 L methanol. After filtration, the solvent was evaporated under reduced pressure, yielding both the residue and the methanolic extract (EME extract, 10.3 g). The residual marc was used to produce the aqueous extract under similar conditions (8.3 g). The Scheme S1 below illustrates the process.

3. Phytochemical screening test

The presence of major classes of secondary metabolites was confirmed using phytochemical tests. These included Dragendorff's test for alkaloids, Shinoda's test for flavonoids, and Liebermann-Burchard's test for steroids, among others. Phytochemical analysis was conducted to detect secondary metabolites including anthocyanins, alkaloids, flavonoids, tannins, triterpenoids, saponins, and steroids.

Flavonoid test

In a test tube, add a few drops of Shinoda reagent (Ethanol 95%, conc. HCl, H_2O) to 3 mL of extract, followed by a few Mg chips. Effervescence is observed. Then add a few drops of isoamyl alcohol. Shake and let it stand. The presence of flavonoids is indicated by the formation of an orange-colored thin film in the isoamyl alcohol supernatant layer.

Tannin test

Place 1 mL of the aqueous filtrate into a test tube, then add 1 mL of $FeCl_3$ 2% solution. The appearance of a green coloration with or without a precipitate indicates that the sample contains tannins.

Anthocyanin test

Add 2 mL of 20% HCl solution to 3 mL of the aqueous filtrate, heat in a water bath for few seconds, and allow it to cool. When cooled, a positive test is indicated by the solution turning dark pink, while when heated again, a reddish-purple coloration of anthocyanin chloride, which may crystallize, will develop.

Leuco-anthocyanin test

To 3 mL of the aqueous filtrate, add a few drops of Shinoda reagent and isoamyl alcohol, then heat in a water bath. A red or violet coloration in the supernatant indicates the presence of leuco-anthocyanins.

Free quinone test

Place 5 mL of the organic filtrate in a test tube, then add a few drops of Borntrager reagent and shake vigorously. The appearance of a coloration ranging from orange to bright red indicates a positive test.

Saponin test

The saponin test is performed by placing a few milliliters of the aqueous filtrate into a test tube, then shaking vigorously for a few seconds. Should the foam (1 cm high) be formed and persist after 15 minutes rest, the test is positive.

Coumarin test

Introduce a few mL of the aqueous filtrate into a test tube and then add 1 mL of 10% ammonia solution (NH_4OH 10%). The appearance of a yellow coloration indicates the presence of coumarins.

Alkaloid test

Place 3 mL of the aqueous filtrate into 3 test tubes, then add 1 mL of 0.1 N HCl solution and Mayer reagent (a mixture of 1.36 g of HgCl and 5.0 g KI in 100 mL of distilled water) tube I, Wagner reagent (A mixture of 1.27 g of I_2 and 2 g of KI in 100 mL of distilled water) tube II and Dragendroff reagent (a mixture of 0.85 g of $Bi(NO_3)_3$, 8 g of KI and 20 mL of glacial CH_3COOH in 100 mL of distilled H_2O) tube III each 5 drops. If a precipitate is formed, it indicates that the sample contains alkaloids, with the Mayer reagent giving a white precipitate, the Wagner reagent giving a brown precipitate, and the Dragendroff reagent giving a yellow (cloudy) precipitate.

Steroid and terpenoid test

Place 3 mL of organic extract in a test tube, and then add a few drops of Liebermann-Burchard reagent ($CH_3COOH:H_2SO_4$, 3:1). A violet coloration indicates the presence of triterpenoids and steroids. Separately, triterpenoids develop a purple/violet or blue coloration, while steroids develop a green coloration.

4. Purification and structure elucidation of major constituents from *Croton sylvaticus*

The dichloromethane extract of *Croton sylvaticus* trunk bark was subjected to preparative column chromatography using successively DCM 100%, DCM-MeOH 1%, DCM-MeOH 3% and DCM- MeOH 5% as the eluent system. Two hundred (200) fractions of 20 mL each were collected. After follow-up TLC (with the n-hexane-DCM system (25%, 50%, 75%) and 100% DCM) and ^{1}H-NMR spectral analysis to guide purification, the fractions were grouped, renamed and transferred to other, smaller columns. The compounds below (Figure 1,B) acetyl aleuritolic acid 6, being pure was isolated directly from the 1% DCM-MeOH system and the ^{13}C-NMR spectrum, (100 MHz, $CDCl_3$) shows 32 carbon atoms: 2 for the acetyl group and 30 for the pentacyclic triterpene having nine quaternary carbons (C-4, 8, 10, 13, 14, 17, 20, 28, 1'), five methine groups (CH-3, 5, 9, 15, 18), ten methylene groups (CH_2-1, 2, 6, 7, 11, 12, 16, 19, 21, 22) and eight methyl groups (CH_3-23, 24, 25, 26, 27, 29, 30, 2'') directly detected in the extract except for quaternary carbons (C-14, 28). Caryophyllene oxide 7 was obtained after isolation of a second purification of one of the fractions from the DCM- MeOH 5% system and the ^{13}C-NMR and DEPT spectra (100 MHz, $CDCl_3$) show signals from 15 carbon atoms in total, with 2 signals from olefinic carbons at δ 152.06 ppm (C-8) and 112.97 ppm (C-13) ppm, three quaternary carbons (C-4, 8, 11), five methylenes (C-2, 3, 6, 7, 10, 13), three methyl (C-12, 14, 15), three methine (C-1, 5, 9) which were all detected in the extract. Phytol 8 was also obtained after a second purification of one of the fractions from the 1% DCM-MeOH system and the ^{13}C-NMR and DEPT spectra, 100 MHz, $CDCl_3$ compared with those in the literature indicate signals from 20 carbon atoms in total with 2 olefinics carbon signals at C_δ 123.3 (C-2) and 140.6 (C-3) ppm, one signal at δ 59.7 (C-1) ppm, five methyl (C-16, 17, 18, 19, 20), ten methylene (C-1, 4, 5, 6, 8, 9, 10, 12, 13, 14), four methine (C-2, 7, 11, 15) and one quaternary (C-3) not detectable in the extract. The figure S2 describes the purification of the dichloromethane extract of *Croton sylvaticus* (EDCM/CS).

Table S1. Types of compounds, numbers and rank in MixoNat

			Data Base 1		Data Base 2	
**N°**	**Compounds**	**Types**	**Numbers**	**Rank**	**Numbers**	**Rank**
**1**	**Terpenes**	Triterpenes	10	131-132, 263-264, 402, 406, 409, 411-413, 414.	222	93, 96, 100, 122, 124, 133, 141, 144, 146-147, 149, 152-153, 156, 161, 164-167, 169-172, 174, 176-177, 179-189, 192-195, 197, 203-204, 207-225, 227-249, 250-258, 260-298, 300-326, 328-353, 355-359, 362-379, 383, 385, 394, 402-404, 407, 424, 428, 443, 456-457.
		Diterpenes	34	80, 138, 142, 144-148, 150-159, 309-313, 393-394, 397, 403-405, 407-408, 410, 412, 450,	105	62, 64-65, 69-70, 75-77, 81-82, 84-86, 88-89, 95, 99, 103, 110-111, 116-117, 120, 126-132, 134-135, 137, 139-140, 145, 150-151, 155, 158, 162, 175, 190, 196, 299, 354, 360-361, 380, 404, 422, 425, 431, 436-439, 444-445, 449, 451-455, 459-461, 464-500.
		Sesquiterpenes	181	12, 22, 33, 37-38, 41-42, 49-50, 57, 59, 61, 63, 64-65, 67-69, 71, 75, 79, 81, 86, 88-90, 93, 96-98, 100, 101, 103, 104, 105-110, 113-115, 118-119, 121, 123, 126, 128, 176, 180, 182, 184, 185-262, 451-500.	44	16-18, 24, 28-29, 34, 41-42, 44, 50, 53, 55, 58, 60-61, 66-67, 71, 73, 79-80, 83, 87, 90-92, 94, 97-98, 104-106, 108-109, 112-115, 123, 125, 136, 138, 148.
		Monoterpenes	151	1-2, 18, 20-21, 23-32, 34, 39, 40, 43, 44-46, 48, 52-55, 58, 60, 62, 66, 70, 72-74, 76, 78, 82-83, 85, 91-92, 94-95, 102, 111-112, 117, 120, 124, 269, 281-298, 305-308, 316-321, 323-325, 327-329, 333-392, 395-396, 398-401.	20	2, 6-9, 19, 25-27, 30, 37-40, 43, 45, 49, 59, 101-102
**2**	**Stéroids**		05	129, 133, 134, 177, 301.	58	107, 121, 154, 157, 159-160, 163, 168, 173, 178, 4, 198-202, 205-206, 226, 259, 327, 382, 384, 386-393, 395-401, 405-406, 408-420, 423, 426-427, 432-433, 442.
**3**	**Alkaloid**		00	-	01	11

4	**Fatty acids**		22	136-137, 139-141, 143, 149, 160-161, 164-167, 169-171, 173, 178-179, 266-267, 290.	08	22-23, 429, 434-435, 446-448,
5	**Amino acids**		00	-	06	3-4, 10, 12, 15, 21
6	**Others**		97		36	
7	**Total**		**500**		**500**	

Table S2. Skeletons of compounds detected and their rank in MixoNat

Types	Skeletons	Data Base 1 Rank	Data Base 1 Numbers	Data Base 2 Rank	Data Base 2 Numbers
**Triterpenes**					
• Pentacyclic	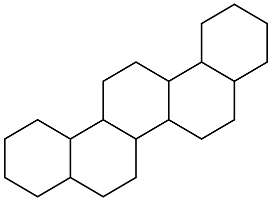<br>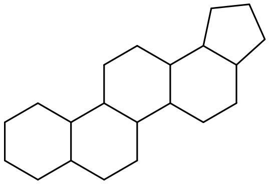	131, 132, 263-264, 402, 406, 409, 411, 413, 414	10	93, 96, 100, 122, 124, 133, 141, 144, 146-147, 149, 152-153, 156, 161, 164-167, 169-172, 174, 176-177, 179-189, 192-195, 197, 203-204, 207-225, 227-249, 250-258, 260-298, 300-326, 328-353, 355-359, 362-379, 383, 385, 394, 402-404, 407, 424, 428, 443, 456-457.	222
**Steroids**					
	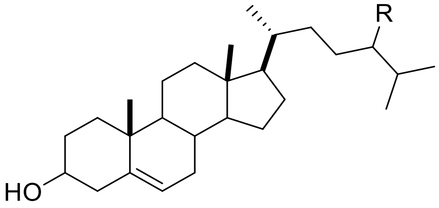	129, 133-134	3	107, 121, 154, 157, 159-160, 163, 168, 173, 178, 4, 198-202, 205-206, 226, 259, 327, 382, 384, 386-393, 395-401, 405-406, 408-420, 423, 426-427, 432-433, 442.	58
**Diterpenes**					

Lathyrane	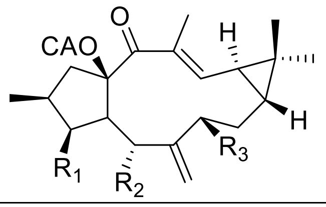	No detected	00	299, 354, 360-361, 380, 404, 422, 425, 431, 439, 444	11
Atisane	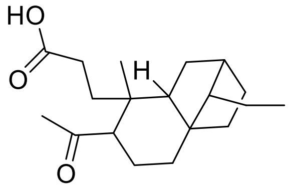	No detected	00	137, 140, 145, 150-151, 155, 158, 175, 190, 196, 452, 454, 472	13
Erythroxylane	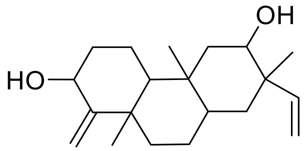	No detected	00	464	01
Beyerane	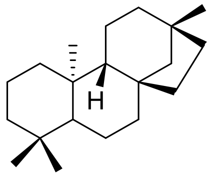	No detected	00	478-479	02
Cleistanthane	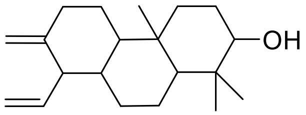	No detected	00	75, 162	02
• Kaurane	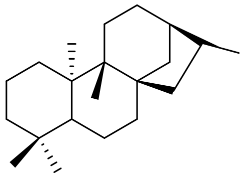	146, 309, 408	3	77, 86, 451, 492-493, 500	06
• Labdane	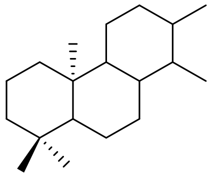	142, 150-151, 154, 158, 310, 393-394, 404-405	10	76, 95, 461, 99, 103, 110-111, 116-117, 126-128, 130-132, 139, 465, 471, 477, 481-483, 485, 488-490, 497-499	29

• Clerodane	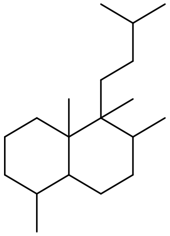	80, 138, 156, 312-313, 410, 450	7	81-82, 84-85, 88-89, 120, 134-135, 445, 453, 455, 487, 491	14
• Abietane	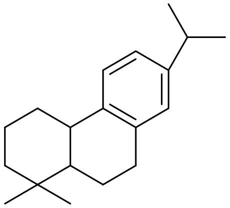	152-153, 155, 157, 159, 311, 403, 407, 412	9	470, 484	02
• Isopimarane	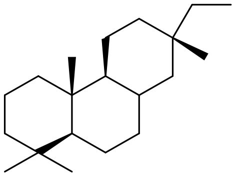	147	1	474	01
• Trachylobane	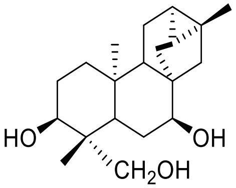	No detected	00	62, 64, 70, 468, 473, 480, 486, 495-496	09
• Cembrane	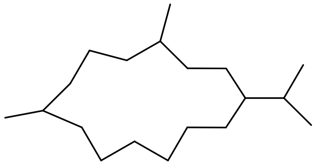	145	1	469	01
• Linear/ Phytane	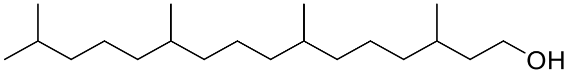	144, 148, 397	3	69, 436-438, 459-460, 466, 475	08
Alkaloid				11	1

Table S3. Scoring and ranking of isolated **6–8** dereplication compounds and their similar compounds in MixoNat

Compound	Structure	Data Base 1				Data Base 2			
		**Rank1/ relatives ou Identique**	**Rank2/ Similars**	**Scores**	**Carbon**	**Rank1/relatives ou Identique**	**Rank2/Similars**	**Scores**	**Carbon**
**6**	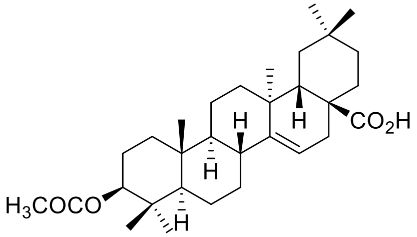	263/0.93 (28/30)	402	0.9	27/30	No detected	266	0.97	31/32
			409	0.9	27/30		268	0.97	31/32
			411	0.9	27/30		273	0.97	31/32
							274	0.97	31/32
**7**	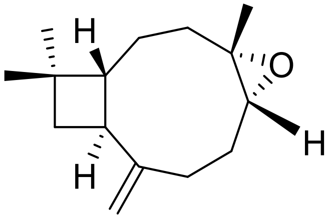	180/0.93 (14/15) 185/0.93 (14/15)	63	1.0	15/15	No detected	90	1.0	15/15
			88	1.0	15/15		104	1.0	15/15
			89	1.0	15/15		105	1.0	15/15
			107	1.0	15/15				
			188	0.93	14/15				
			191	0.93	14/15				
			192	0.93	14/15				
			207	0.93	14/15				
			256	0.93	14/15				
			271	0.93	14/15				
**8**	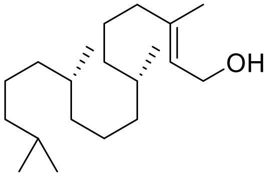	144/0.95 (19/20)	148	0.95	19/20	459/0.95 (19/20) 466/0.95 (19/20) 467/0.95 (19/20) 475/0.95 (19/20)	460	0.95	19/20
			397	0.9	18/20				

Table S4. Scores et Rank compounds in MixoNat

Scores	Rank compound Data base 1	Rank compound Data base 2
1.0	1- 130	1-249
0.97-0.96	131-137	250-445
0.95-0.90	138-415	446-500
0.89-0.87	416-500	-

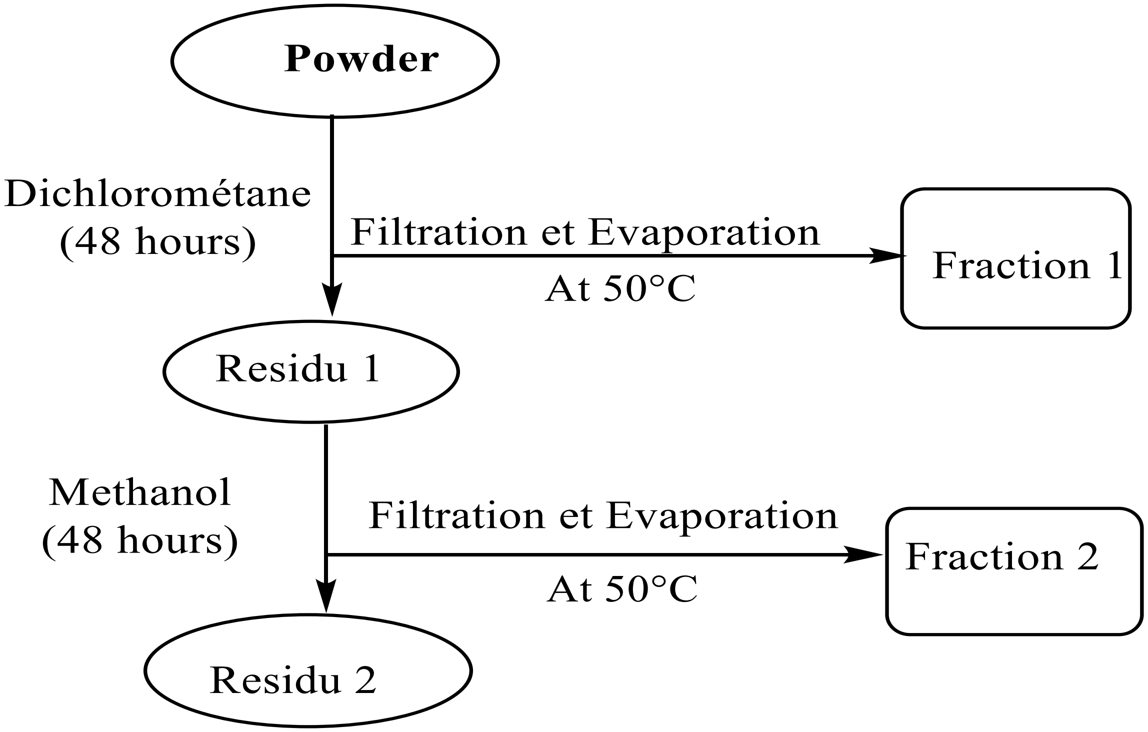

Figure S1. Scheme illustrates the extraction process

A. Compound Data base 1

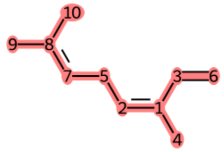

Rank: 1 MW: 136.23
(Z)-beta-Ocimene
Score: 1.0 (10/10 C)
Deviation : 0.61 ppm

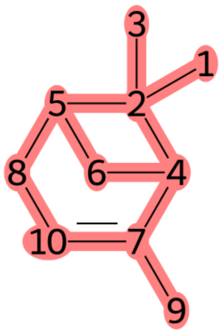

Rank: 2 MW: 136.23
alpha-Pinene
Score: 1.0 (10/10 C)
Deviation : 0.89 ppm

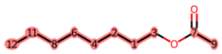

Rank: 3 MW: 172.26
1-Acetoxyoctane
Score: 1.0 (10/10 C)
Deviation : 0.89 ppm

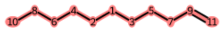

Rank: 4 MW: 154.29
1-Undecene
Score: 1.0 (11/11 C)
Deviation : 1.09 ppm

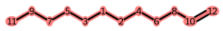

Rank: 5 MW: 168.32
1-Dodecene
Score: 1.0 (12/12 C)
Deviation : 1.2 ppm

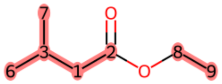

Rank: 6 MW: 130.18
Ethyl isopentanoate
Score: 1.0 (7/7 C)
Deviation : 1.23 ppm

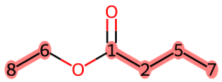

Rank: 7 MW: 116.16
Ethyl butanoate
Score: 1.0 (6/6 C)
Deviation : 1.28 ppm

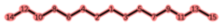

Rank: 8 MW: 212.41
Pentadecane
Score: 1.0 (15/15 C)
Deviation : 1.34 ppm

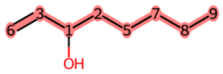

Rank: 9 MW: 128.21
Primaverol
Score: 1.0 (8/8 C)
Deviation : 1.5 ppm

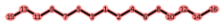

Rank: 10 MW: 226.44
Hexadecane
Score: 1.0 (16/16 C)
Deviation : 1.52 ppm

Figure S2. Figure S11. Dereplication analysis from MixONat, structure of dereplicated compounds: Rank **1-10**.

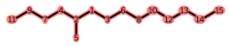

Rank: 11 MW: 212.41
5-Methyltetradecane
Score: 1.0 (15/15 C)
Deviation : 1.58 ppm

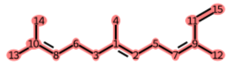

Rank: 12 MW: 204.35
(Z,E)-alpha-Farnesene
Score: 1.0 (15/15 C)
Deviation : 1.59 ppm

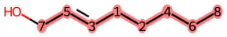

Rank: 13 MW: 128.21
(E)-2-Octenol
Score: 1.0 (8/8 C)
Deviation : 1.59 ppm

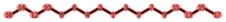

Rank: 14 MW: 240.47
Heptadecane
Score: 1.0 (17/17 C)
Deviation : 1.67 ppm

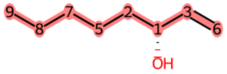

Rank: 15 MW: 128.21
(-)-Matsutakeol
Score: 1.0 (8/8 C)
Deviation : 1.73 ppm

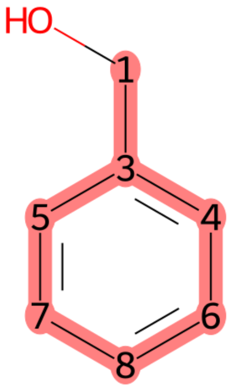

Rank: 16 MW: 108.14
Benzenemethanol
Score: 1.0 (7/7 C)
Deviation : 1.97 ppm

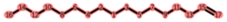

Rank: 17 MW: 238.45
1-Heptadecene
Score: 1.0 (17/17 C)
Deviation : 2.07 ppm

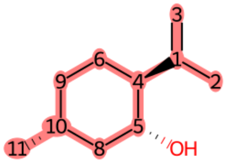

Rank: 18 MW: 154.25
Isopulegol
Score: 1.0 (10/10 C)
Deviation : 2.15 ppm

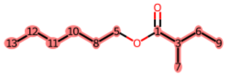

Rank: 19 MW: 186.29
CAS-10032-15-2
Score: 1.0 (11/11 C)
Deviation : 2.18 ppm

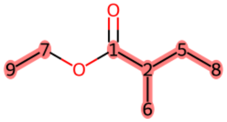

Rank: 20 MW: 130.18
Ethyl 2-methylbutyrate
Score: 1.0 (7/7 C)
Deviation : 2.19 ppm

Figure S3. Figure S12. Dereplication analysis from MixONat, structure of dereplicated compounds: Rank **11-20**

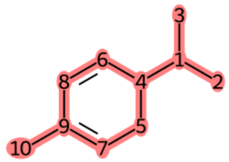

Rank: 21 MW: 136.23
alpha-Phellandrene
Score: 1.0 (10/10 C)
Deviation : 2.29 ppm

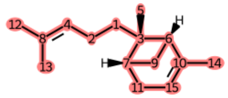

Rank: 22 MW: 204.35
cis-alpha-Bergamotene
Score: 1.0 (15/15 C)
Deviation : 2.34 ppm

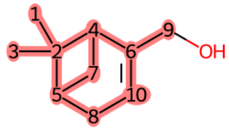

Rank: 23 MW: 152.23
Myrtenol
Score: 1.0 (10/10 C)
Deviation : 2.4 ppm

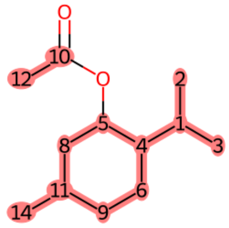

Rank: 24 MW: 198.3
Menthyl acetate
Score: 1.0 (12/12 C)
Deviation : 2.43 ppm

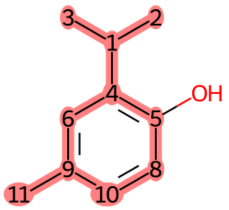

Rank: 25 MW: 150.22
Isothymol
Score: 1.0 (10/10 C)
Deviation : 2.48 ppm

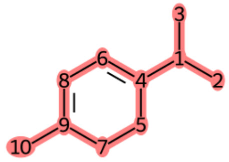

Rank: 26 MW: 136.23
alpha-Terpinene
Score: 1.0 (10/10 C)
Deviation : 2.52 ppm

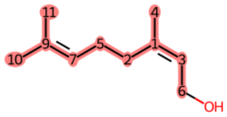

Rank: 27 MW: 154.25
Nerol
Score: 1.0 (10/10 C)
Deviation : 2.54 ppm

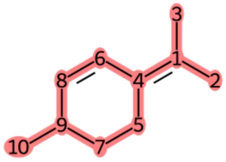

Rank: 28 MW: 136.23
Isoterpinolene
Score: 1.0 (10/10 C)
Deviation : 2.55 ppm

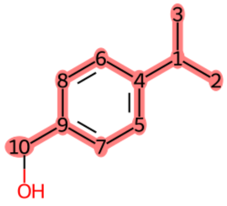

Rank: 29 MW: 150.22
Cuminic alcohol
Score: 1.0 (10/10 C)
Deviation : 2.57 ppm

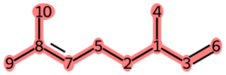

Rank: 30 MW: 138.25
beta-Citronellene
Score: 1.0 (10/10 C)
Deviation : 2.58 ppm

Figure S4. Dereplication analysis from MixONat, structure of dereplicated compounds: Rank **21-30**.

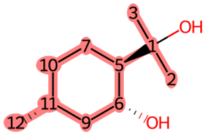

Rank: 31 MW: 172.26
Isopulegol hydrate
Score: 1.0 (10/10 C)
Deviation : 2.58 ppm

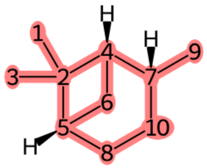

Rank: 32 MW: 138.25
cis-Pinane
Score: 1.0 (10/10 C)
Deviation : 2.6 ppm

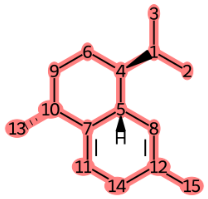

Rank: 33 MW: 204.35
(-)-Cadina-1,4-diene
Score: 1.0 (15/15 C)
Deviation : 2.61 ppm

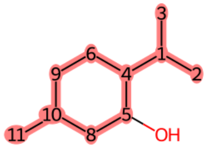

Rank: 34 MW: 156.27
Menthol
Score: 1.0 (10/10 C)
Deviation : 2.61 ppm

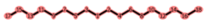

Rank: 35 MW: 254.49
Octadecane
Score: 1.0 (18/18 C)
Deviation : 2.62 ppm

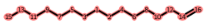

Rank: 36 MW: 224.43
1-Hexadecene
Score: 1.0 (16/16 C)
Deviation : 2.65 ppm

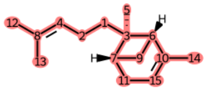

Rank: 37 MW: 204.35
CAS-13474-59-4
Score: 1.0 (15/15 C)
Deviation : 2.66 ppm

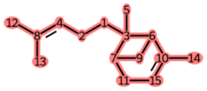

Rank: 38 MW: 204.35
alpha-Bergamotene
Score: 1.0 (15/15 C)
Deviation : 2.66 ppm

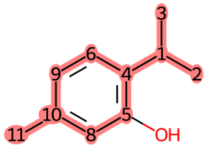

Rank: 39 MW: 150.22
m-Thymol
Score: 1.0 (10/10 C)
Deviation : 2.77 ppm

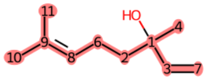

Rank: 40 MW: 154.25
Linalol
Score: 1.0 (10/10 C)
Deviation : 2.86 ppm

Figure S5. Dereplication analysis from MixONat, structure of dereplicated compounds: Rank **31-40.**

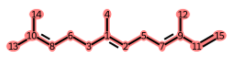

Rank: 41 MW: 204.35
alpha-Farnesene
Score: 1.0 (15/15 C)
Deviation : 2.94 ppm

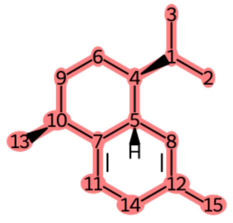

Rank: 42 MW: 204.35
Cadina-1,4-diene
Score: 1.0 (15/15 C)
Deviation : 2.97 ppm

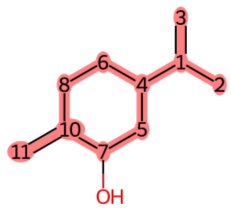

Rank: 43 MW: 152.23
Isocarveol
Score: 1.0 (10/10 C)
Deviation : 3.02 ppm

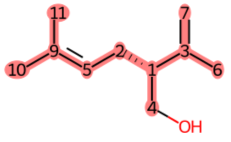

Rank: 44 MW: 154.25
(-)-Lavandulol
Score: 1.0 (10/10 C)
Deviation : 3.02 ppm

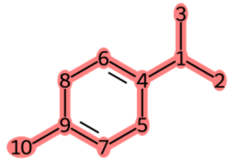

Rank: 45 MW: 136.23
gamma-Terpinene
Score: 1.0 (10/10 C)
Deviation : 3.07 ppm

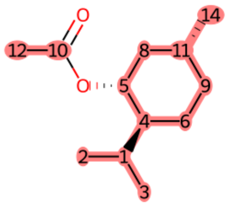

Rank: 46 MW: 198.3
Menthyl acetate
Score: 1.0 (12/12 C)
Deviation : 3.07 ppm

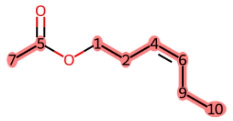

Rank: 47 MW: 142.2
(Z)-3-Hexenyl acetate
Score: 1.0 (8/8 C)
Deviation : 3.08 ppm

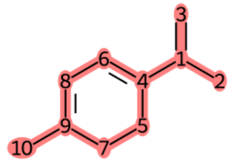

Rank: 48 MW: 134.22
p-Menta-1,3,8-triene
Score: 1.0 (10/10 C)
Deviation : 3.11 ppm

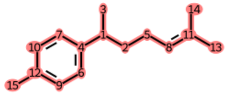

Rank: 49 MW: 202.34
alpha-Curcumene
Score: 1.0 (15/15 C)
Deviation : 3.14 ppm

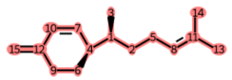

Rank: 50 MW: 204.35
CAS-20307-83-9
Score: 1.0 (15/15 C)
Deviation : 3.14 ppm

Figure S6. Dereplication analysis from MixONat, structure of dereplicated compounds: Rank **41-50**.

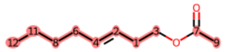

Rank: 51 MW: 170.25
3-Octenyl acetate
Score: 1.0 (10/10 C)
Deviation : 3.2 ppm

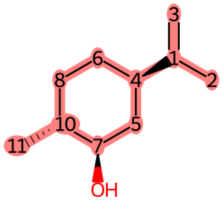

Rank: 52 MW: 154.25
Dihydrocarveol
Score: 1.0 (10/10 C)
Deviation : 3.23 ppm

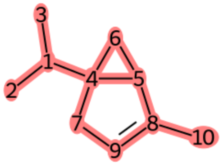

Rank: 53 MW: 136.23
Origanene
Score: 1.0 (10/10 C)
Deviation : 3.26 ppm

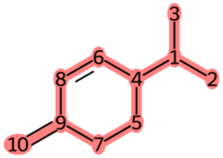

Rank: 54 MW: 136.23
beta-Phellandrene
Score: 1.0 (10/10 C)
Deviation : 3.28 ppm

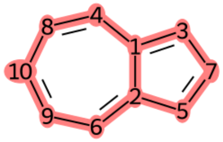

Rank: 55 MW: 128.17
Azulene
Score: 1.0 (10/10 C)
Deviation : 3.31 ppm

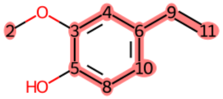

Rank: 56 MW: 150.17
p-Vinylguaiacol
Score: 1.0 (9/9 C)
Deviation : 3.33 ppm

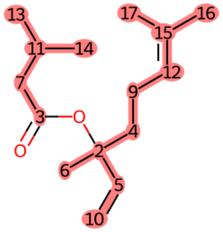

Rank: 57 MW: 238.37
Linalyl isovalerate
Score: 1.0 (15/15 C)
Deviation : 3.34 ppm

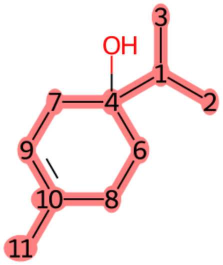

Rank: 58 MW: 154.25
4-Terpineol
Score: 1.0 (10/10 C)
Deviation : 3.36 ppm

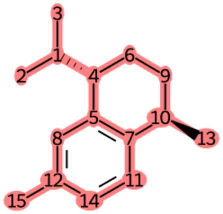

Rank: 59 MW: 202.34
trans-Calamenene
Score: 1.0 (15/15 C)
Deviation : 3.37 ppm

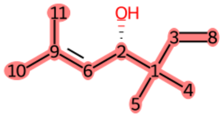

Rank: 60 MW: 154.25
(-)-Artemisia alcohol
Score: 1.0 (10/10 C)
Deviation : 3.37 ppm

Figure S7. Dereplication analysis from MixONat, structure of dereplicated compounds: Rank **51-60**.

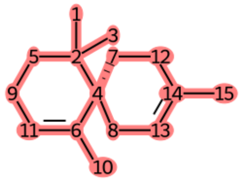

Rank: 61 MW: 204.35
(-)-alpha-Chamigrene
Score: 1.0 (15/15 C)
Deviation : 3.49 ppm

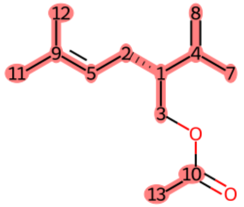

Rank: 62 MW: 196.29
(-)-Lavandulyl acetate
Score: 1.0 (12/12 C)
Deviation : 3.5 ppm

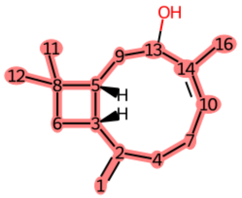

Rank: 63 MW: 220.35
CAS-1334513-72-2
Score: 1.0 (15/15 C)
Deviation : 3.51 ppm

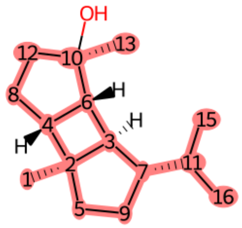

Rank: 64 MW: 222.37
Bourbonanol
Score: 1.0 (15/15 C)
Deviation : 3.52 ppm

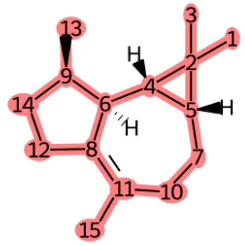

Rank: 65 MW: 204.35
(+)-Ledene
Score: 1.0 (15/15 C)
Deviation : 3.55 ppm

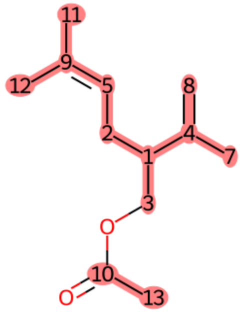

Rank: 66 MW: 196.29
Lavandulol acetate
Score: 1.0 (12/12 C)
Deviation : 3.6 ppm

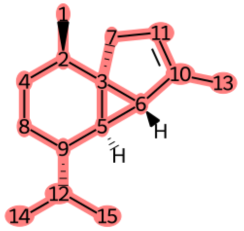

Rank: 67 MW: 204.35
(-)-alpha-Cubebene
Score: 1.0 (15/15 C)
Deviation : 3.62 ppm

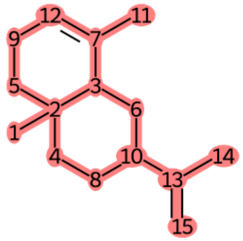

Rank: 68 MW: 204.35
epi-alpha-Selinene
Score: 1.0 (15/15 C)
Deviation : 3.64 ppm

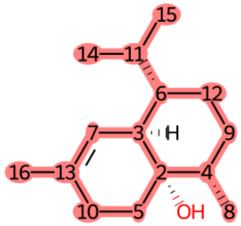

Rank: 69 MW: 222.37
1,10-Diepicubenol
Score: 1.0 (15/15 C)
Deviation : 3.7 ppm

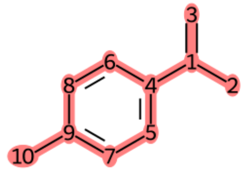

Rank: 70 MW: 132.2
p-Cymenene
Score: 1.0 (10/10 C)
Deviation : 3.71 ppm

Figure S8. Dereplication analysis from MixONat, structure of dereplicated compounds: Rank **61-70**.

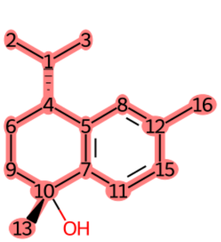

Rank: 71 MW: 218.33
cis-Calamenen-10-ol
Score: 1.0 (15/15 C)
Deviation : 3.71 ppm

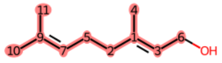

Rank: 72 MW: 154.25
Citrol
Score: 1.0 (10/10 C)
Deviation : 3.74 ppm

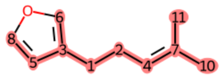

Rank: 73 MW: 150.22
Perillene
Score: 1.0 (10/10 C)
Deviation : 3.76 ppm

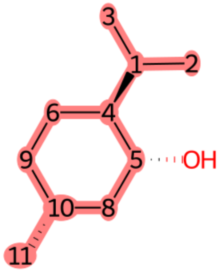

Rank: 74 MW: 156.27
(-)-Menthol
Score: 1.0 (10/10 C)
Deviation : 3.8 ppm

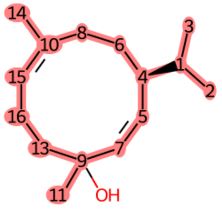

Rank: 75 MW: 222.37
Germacrene D-4-ol
Score: 1.0 (15/15 C)
Deviation : 3.8 ppm

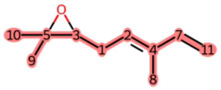

Rank: 76 MW: 152.23
Myroxide
Score: 1.0 (10/10 C)
Deviation : 3.84 ppm

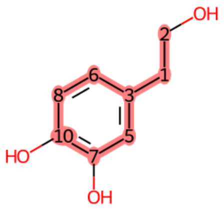

Rank: 77 MW: 154.16
3-Hydroxytyrosol
Score: 1.0 (8/8 C)
Deviation : 3.85 ppm

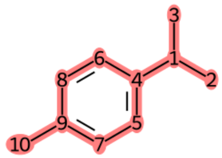

Rank: 78 MW: 134.22
p-Cymene
Score: 1.0 (10/10 C)
Deviation : 3.88 ppm

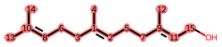

Rank: 79 MW: 222.37
Farnesol
Score: 1.0 (15/15 C)
Deviation : 3.88 ppm

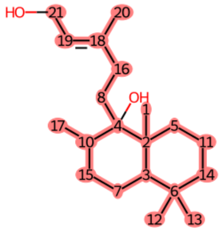

Rank: 80 MW: 308.5
CAS-189371-55-9
Score: 1.0 (20/20 C)
Deviation : 3.91 ppm

Figure S9. Dereplication analysis from MixONat, structure of dereplicated compounds: Rank **71-80**.

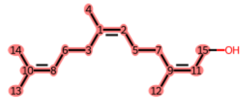

Rank: 81 MW: 222.37
(Z,Z)-Farnesol
Score: 1.0 (15/15 C)
Deviation : 3.93 ppm

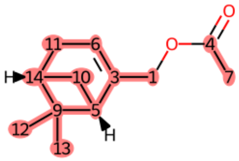

Rank: 82 MW: 194.27
(+)-Myrtenyl acetate
Score: 1.0 (12/12 C)
Deviation : 3.94 ppm

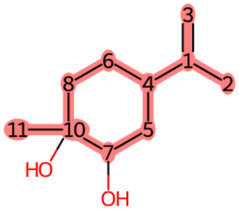

Rank: 83 MW: 170.25
Limonene glycol
Score: 1.0 (10/10 C)
Deviation : 3.95 ppm

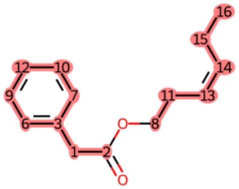

Rank: 84 MW: 218.29
CAS-42436-07-7
Score: 1.0 (14/14 C)
Deviation : 3.99 ppm

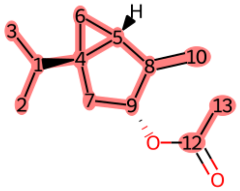

Rank: 85 MW: 194.27
cis-Sabinyl acetate
Score: 1.0 (12/12 C)
Deviation : 4.01 ppm

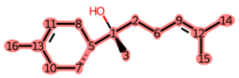

Rank: 86 MW: 222.37
alpha-Bisabolol
Score: 1.0 (15/15 C)
Deviation : 4.02 ppm

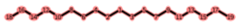

Rank: 87 MW: 268.52
Nonadecane
Score: 1.0 (19/19 C)
Deviation : 4.03 ppm

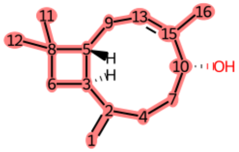

Rank: 88 MW: 220.35
Caryophyllenol II
Score: 1.0 (15/15 C)
Deviation : 4.04 ppm

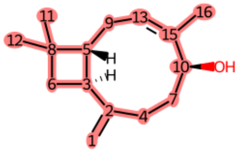

Rank: 89 MW: 220.35
Caryophyllenol I
Score: 1.0 (15/15 C)
Deviation : 4.08 ppm

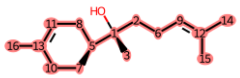

Rank: 90 MW: 222.37
(-)-Anymol
Score: 1.0 (15/15 C)
Deviation : 4.08 ppm

Figure S10. Dereplication analysis from MixONat, structure of dereplicated compounds: Rank **81-90**.

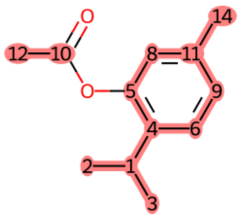

Rank: 91 MW: 192.25
Thymyl acetate
Score: 1.0 (12/12 C)
Deviation : 4.14 ppm

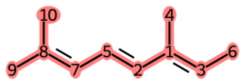

Rank: 92 MW: 136.23
Alloocimene
Score: 1.0 (10/10 C)
Deviation : 4.15 ppm

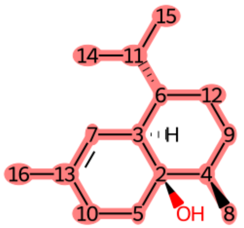

Rank: 93 MW: 222.37
(-)-Cubenol
Score: 1.0 (15/15 C)
Deviation : 4.3 ppm

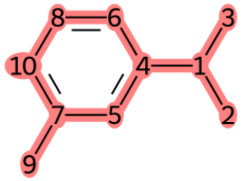

Rank: 94 MW: 134.22
m-Cymene
Score: 1.0 (10/10 C)
Deviation : 4.31 ppm

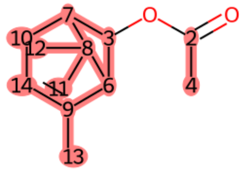

Rank: 95 MW: 194.27
CAS-67999-48-8
Score: 1.0 (12/12 C)
Deviation : 4.31 ppm

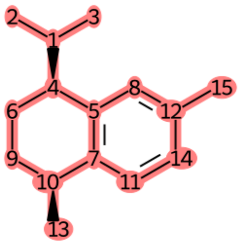

Rank: 96 MW: 202.34
cis-Calamenene
Score: 1.0 (15/15 C)
Deviation : 4.35 ppm

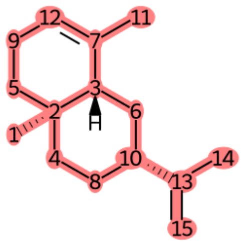

Rank: 97 MW: 204.35
(-)-alpha-Selinene
Score: 1.0 (15/15 C)
Deviation : 4.4 ppm

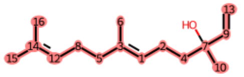

Rank: 98 MW: 222.37
(E)-Nerolidol
Score: 1.0 (15/15 C)
Deviation : 4.4 ppm

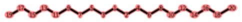

Rank: 99 MW: 282.55
Eicosane
Score: 1.0 (20/20 C)
Deviation : 4.54 ppm

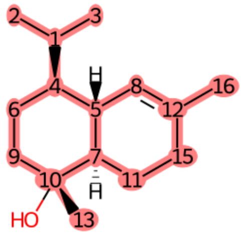

Rank: 100 MW: 222.37
(-)-alpha-Cadinol
Score: 1.0 (15/15 C)
Deviation : 4.56 ppm

Figure S11. Dereplication analysis from MixONat, structure of dereplicated compounds: Rank **91-100**.

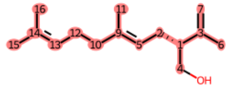

Rank: 101 MW: 222.37
trans-Sesquilavandulol
Score: 1.0 (15/15 C)
Deviation : 4.56 ppm

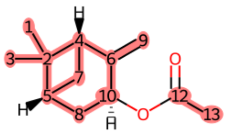

Rank: 102 MW: 194.27
CAS-1686-15-3
Score: 1.0 (12/12 C)
Deviation : 4.58 ppm

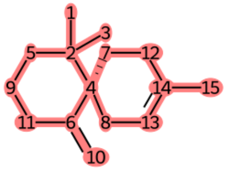

Rank: 103 MW: 204.35
(-)-beta-Chamigrene
Score: 1.0 (15/15 C)
Deviation : 4.59 ppm

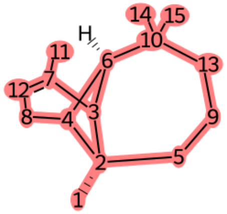

Rank: 104 MW: 204.35
(+)-alpha-Longipinene
Score: 1.0 (15/15 C)
Deviation : 4.63 ppm

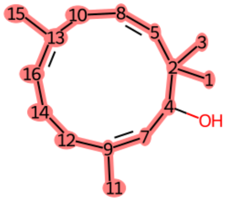

Rank: 105 MW: 220.35
CAS-108043-85-2
Score: 1.0 (15/15 C)
Deviation : 4.68 ppm

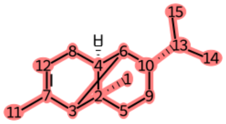

Rank: 106 MW: 204.35
(-)-alpha-Copaene
Score: 1.0 (15/15 C)
Deviation : 4.69 ppm

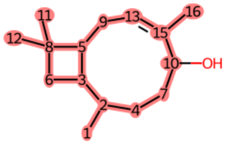

Rank: 107 MW: 222.37
Caryophyllenyl alcohol
Score: 1.0 (15/15 C)
Deviation : 4.71 ppm

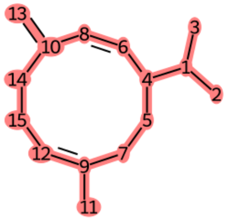

Rank: 108 MW: 204.35
CAS-37839-63-7
Score: 1.0 (15/15 C)
Deviation : 4.86 ppm

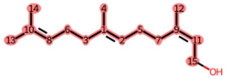

Rank: 109 MW: 222.37
(Z,E)-Farnesol
Score: 1.0 (15/15 C)
Deviation : 4.96 ppm

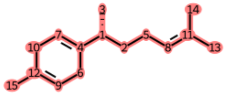

Rank: 110 MW: 204.35
(-)-beta-Curcumene
Score: 1.0 (15/15 C)
Deviation : 5.07 ppm

Figure S12. Dereplication analysis from MixONat, structure of dereplicated compounds: Rank **101-110**.

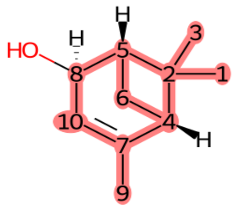

Rank: 111 MW: 152.23
trans-Verbenol
Score: 1.0 (10/10 C)
Deviation : 5.11 ppm

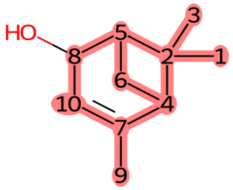

Rank: 112 MW: 152.23
Verbenol
Score: 1.0 (10/10 C)
Deviation : 5.16 ppm

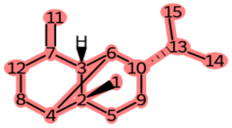

Rank: 113 MW: 204.35
cis-beta-Copaene
Score: 1.0 (15/15 C)
Deviation : 5.19 ppm

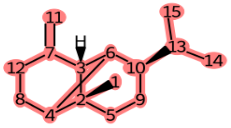

Rank: 114 MW: 204.35
beta-Ylangene
Score: 1.0 (15/15 C)
Deviation : 5.19 ppm

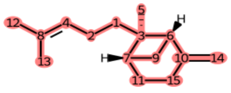

Rank: 115 MW: 204.35
beta-trans-Bergamotene
Score: 1.0 (15/15 C)
Deviation : 5.2 ppm

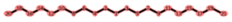

Rank: 116 MW: 296.57
Heneicosane
Score: 1.0 (21/21 C)
Deviation : 5.21 ppm

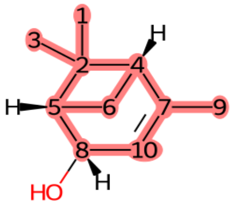

Rank: 117 MW: 152.23
cis-Verbenol
Score: 1.0 (10/10 C)
Deviation : 5.36 ppm

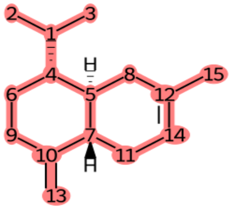

Rank: 118 MW: 204.35
CAS-29336-47-8
Score: 1.0 (15/15 C)
Deviation : 5.45 ppm

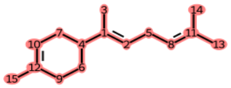

Rank: 119 MW: 204.35
(E)-alpha-Bisabolene
Score: 1.0 (15/15 C)
Deviation : 5.56 ppm

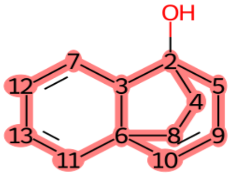

Rank: 120 MW: 174.24
CAS-1233955-15-1
Score: 1.0 (12/12 C)
Deviation : 5.58 ppm

Figure S13. Dereplication analysis from MixONat, structure of dereplicated compounds: Rank **111-120**.

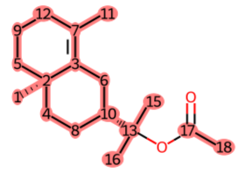

Rank: 121 MW: 264.4
gamma-Eudesmol acetate
Score: 1.0 (17/17 C)
Deviation : 5.82 ppm

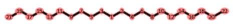

Rank: 122 MW: 310.6
Docosane
Score: 1.0 (22/22 C)
Deviation : 5.84 ppm

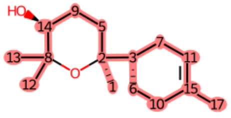

Rank: 123 MW: 238.37
CAS-22567-36-8
Score: 1.0 (15/15 C)
Deviation : 6.07 ppm

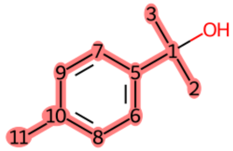

Rank: 124 MW: 150.22
p-Cymene-8-ol
Score: 1.0 (10/10 C)
Deviation : 6.08 ppm

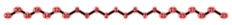

Rank: 125 MW: 324.63
Tricosane
Score: 1.0 (23/23 C)
Deviation : 6.61 ppm

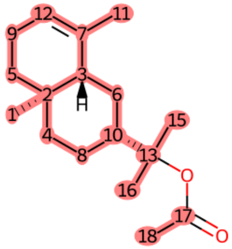

Rank: 126 MW: 264.4
alpha-Eudesmol acetate
Score: 1.0 (17/17 C)
Deviation : 6.84 ppm

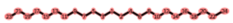

Rank: 127 MW: 338.65
Tetracosane
Score: 1.0 (24/24 C)
Deviation : 7.45 ppm

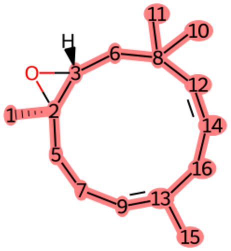

Rank: 128 MW: 220.35
Humulene epoxide I
Score: 1.0 (15/15 C)
Deviation : 7.76 ppm

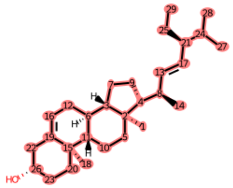

Rank: 129 MW: 412.69
beta-Stigmasterol
Score: 1.0 (29/29 C)
Deviation : 8.17 ppm

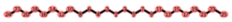

Rank: 130 MW: 352.68
Pentacosane
Score: 1.0 (25/25 C)
Deviation : 8.41 ppm

Figure S14. Dereplication analysis from MixONat, structure of dereplicated compounds: Rank **121-130**.

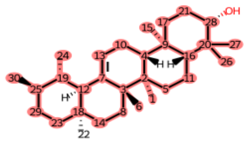

Rank: 131 MW: 426.72
Viminalol
Score: 0.97 (29/30 C)
Deviation : 7.09 ppm

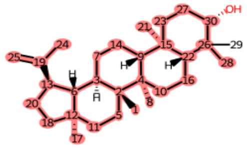

Rank: 132 MW: 426.72
(+)-Lupeol
Score: 0.97 (29/30 C)
Deviation : 9.04 ppm

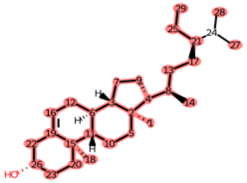

Rank: 133 MW: 414.71
Nimbosterol
Score: 0.97 (28/29 C)
Deviation : 6.13 ppm

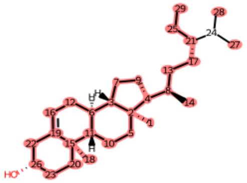

Rank: 134 MW: 414.71
Clionasterol
Score: 0.97 (28/29 C)
Deviation : 6.56 ppm

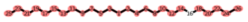

Rank: 135 MW: 366.71
Hexacosane
Score: 0.96 (25/26 C)
Deviation : 8.28 ppm

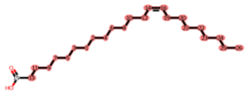

Rank: 136 MW: 366.62
Nevonic acid
Score: 0.96 (23/24 C)
Deviation : 4.5 ppm

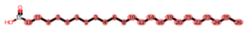

Rank: 137 MW: 368.64
Tetracosanoic acid
Score: 0.96 (23/24 C)
Deviation : 7.24 ppm

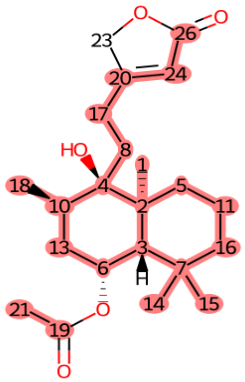

Rank: 138 MW: 378.5
Vitexilactone
Score: 0.95 (21/22 C)
Deviation : 4.94 ppm

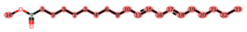

Rank: 139 MW: 322.53
CAS-2463-02-7
Score: 0.95 (20/21 C)
Deviation : 3.14 ppm

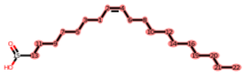

Rank: 140 MW: 310.51
Gadoleic acid
Score: 0.95 (19/20 C)
Deviation : 2.01 ppm

Figure S15. Dereplication analysis from MixONat, structure of dereplicated compounds: Rank **131-140**.

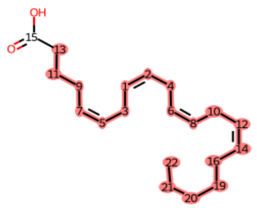

Rank: 141 MW: 304.47
Arachidonic acid
Score: 0.95 (19/20 C)
Deviation : 2.27 ppm

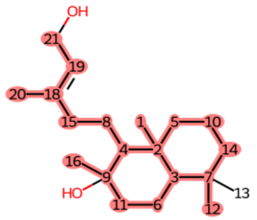

Rank: 142 MW: 308.5
CAS-79434-22-3
Score: 0.95 (19/20 C)
Deviation : 2.29 ppm

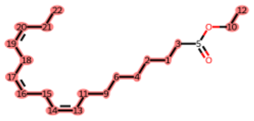

Rank: 143 MW: 306.48
Ethyl alpha-linolenate
Score: 0.95 (19/20 C)
Deviation : 2.53 ppm

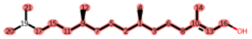

Rank: 144 MW: 296.53
(E)-Phytol
Score: 0.95 (19/20 C)
Deviation : 2.68 ppm

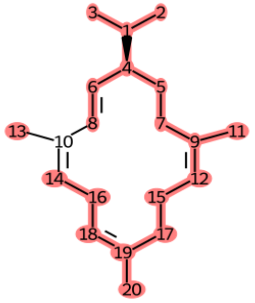

Rank: 145 MW: 272.47
(-)-Cembrene
Score: 0.95 (19/20 C)
Deviation : 3.57 ppm

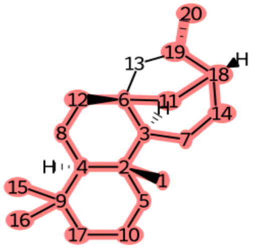

Rank: 146 MW: 274.48
Kaurane
Score: 0.95 (19/20 C)
Deviation : 4.05 ppm

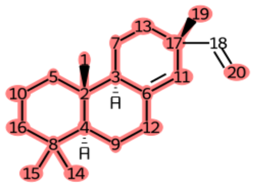

Rank: 147 MW: 272.47
CAS-1686-56-2
Score: 0.95 (19/20 C)
Deviation : 4.08 ppm

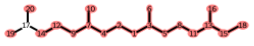

Rank: 148 MW: 282.55
CAS-638-36-8
Score: 0.95 (19/20 C)
Deviation : 4.14 ppm

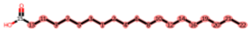

Rank: 149 MW: 312.53
Eicosanoic acid
Score: 0.95 (19/20 C)
Deviation : 4.41 ppm

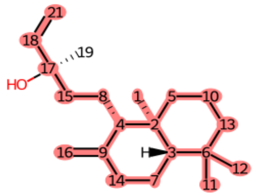

Rank: 150 MW: 290.48
Manool
Score: 0.95 (19/20 C)
Deviation : 4.81 ppm

Figure S16. Dereplication analysis from MixONat, structure of dereplicated compounds: Rank **141-150**.

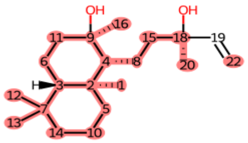

Rank: 151 MW: 308.5
(-)-Sclareol
Score: 0.95 (19/20 C)
Deviation : 4.89 ppm

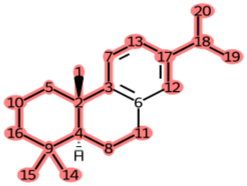

Rank: 152 MW: 270.45
(+)-Dehydroabietadiene
Score: 0.95 (19/20 C)
Deviation : 4.95 ppm

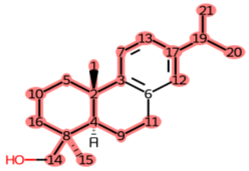

Rank: 153 MW: 286.45
4-Epidehydroabietol
Score: 0.95 (19/20 C)
Deviation : 4.98 ppm

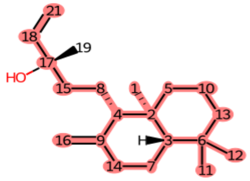

Rank: 154 MW: 290.48
13-Epimanool
Score: 0.95 (19/20 C)
Deviation : 5.72 ppm

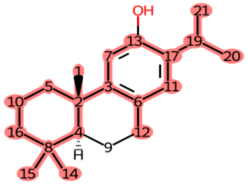

Rank: 155 MW: 286.45
Ferruginol
Score: 0.95 (19/20 C)
Deviation : 6.19 ppm

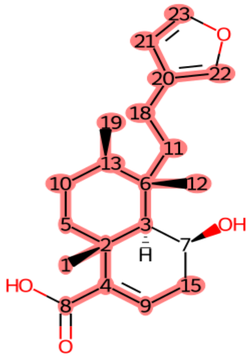

Rank: 156 MW: 332.43
Divinatorin A
Score: 0.95 (19/20 C)
Deviation : 6.34 ppm

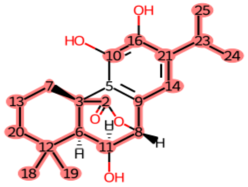

Rank: 157 MW: 346.42
Epiisorosmanol
Score: 0.95 (19/20 C)
Deviation : 6.51 ppm

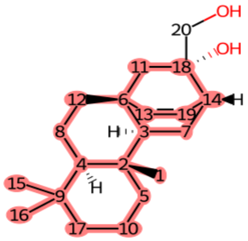

Rank: 158 MW: 304.47
Serradiol
Score: 0.95 (19/20 C)
Deviation : 6.81 ppm

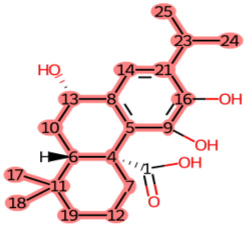

Rank: 159 MW: 348.43
Carnosolic acid
Score: 0.95 (19/20 C)
Deviation : 7.08 ppm

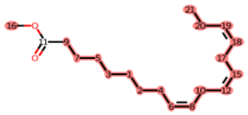

Rank: 160 MW: 292.46
CAS-301-00-8
Score: 0.95 (18/19 C)
Deviation : 1.64 ppm

Figure S17. Dereplication analysis from MixONat, structure of dereplicated compounds: Rank **151-160**.

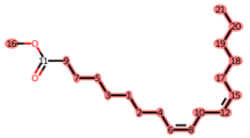

Rank: 161 MW: 294.47
CAS-112-63-0
Score: 0.95 (18/19 C)
Deviation : 1.86 ppm

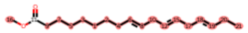

Rank: 162 MW: 292.46
CAS-7361-80-0
Score: 0.95 (18/19 C)
Deviation : 1.89 ppm

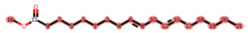

Rank: 163 MW: 294.47
CAS-2462-85-3
Score: 0.95 (18/19 C)
Deviation : 2.32 ppm

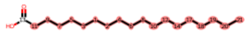

Rank: 164 MW: 298.5
Nonadecanoic acid
Score: 0.95 (18/19 C)
Deviation : 4.05 ppm

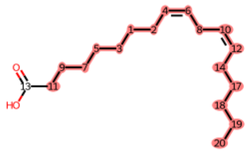

Rank: 165 MW: 280.45
Linoleic acid
Score: 0.94 (17/18 C)
Deviation : 0.97 ppm

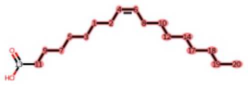

Rank: 166 MW: 282.46
Oleic acid
Score: 0.94 (17/18 C)
Deviation : 1.37 ppm

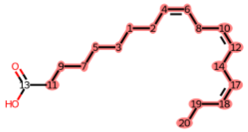

Rank: 167 MW: 278.43
Linolenic acid
Score: 0.94 (17/18 C)
Deviation : 1.45 ppm

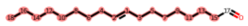

Rank: 168 MW: 266.46
9-Octadecenal
Score: 0.94 (17/18 C)
Deviation : 1.97 ppm

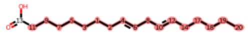

Rank: 169 MW: 280.45
CAS-2197-37-7
Score: 0.94 (17/18 C)
Deviation : 2.01 ppm

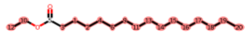

Rank: 170 MW: 284.48
Ethyl palmitate
Score: 0.94 (17/18 C)
Deviation : 2.44 ppm

Figure S18. Dereplication analysis from MixONat, structure of dereplicated compounds: Rank **161-170**.

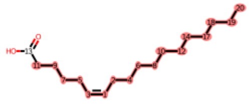

Rank: 171 MW: 282.46
Petroselinic acid
Score: 0.94 (17/18 C)
Deviation : 2.85 ppm

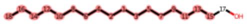

Rank: 172 MW: 270.49
1-Octadecanol
Score: 0.94 (17/18 C)
Deviation : 3.5 ppm

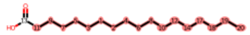

Rank: 173 MW: 284.48
Octadecanoic acid
Score: 0.94 (17/18 C)
Deviation : 4.51 ppm

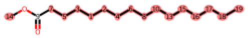

Rank: 174 MW: 270.45
Methyl palmitate
Score: 0.94 (16/17 C)
Deviation : 1.63 ppm

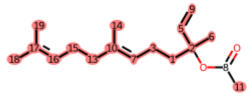

Rank: 175 MW: 264.4
(E)-Nerolidyl acetate
Score: 0.94 (16/17 C)
Deviation : 3.93 ppm

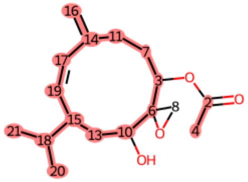

Rank: 176 MW: 294.39
CAS-113581-01-4
Score: 0.94 (16/17 C)
Deviation : 5.64 ppm

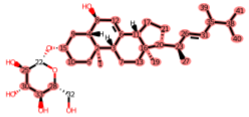

Rank: 177 MW: 576.8
CAS-1639928-98-5
Score: 0.94 (32/34 C)
Deviation : 7.17 ppm

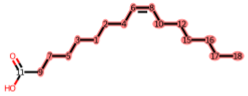

Rank: 178 MW: 254.41
Palmitoleic acid
Score: 0.94 (15/16 C)
Deviation : 1.18 ppm

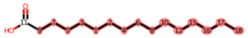

Rank: 179 MW: 256.42
Hexadecanoic acid
Score: 0.94 (15/16 C)
Deviation : 2.0 ppm

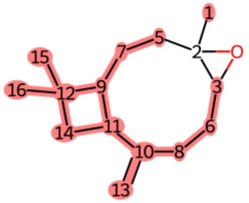

Rank: 180 MW: 220.35
Isocaryophyllene oxide
Score: 0.93 (14/15 C)
Deviation : 1.4 ppm

Figure S19. Dereplication analysis from MixONat, structure of dereplicated compounds: Rank **171-180**.

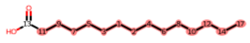

Rank: 181 MW: 242.4
Pentadecanoic acid
Score: 0.93 (14/15 C)
Deviation : 1.43 ppm

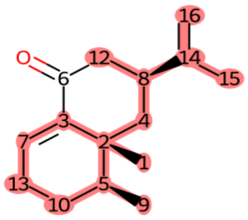

Rank: 182 MW: 218.33
Eremophilone
Score: 0.93 (14/15 C)
Deviation : 2.0 ppm

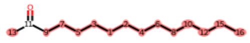

Rank: 183 MW: 226.4
2-Pentadecanone
Score: 0.93 (14/15 C)
Deviation : 2.02 ppm

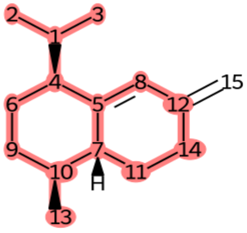

Rank: 184 MW: 204.35
CAS-157477-72-0
Score: 0.93 (14/15 C)
Deviation : 2.14 ppm

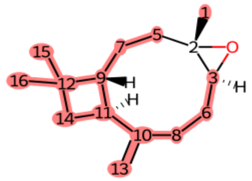

Rank: 185 MW: 220.35
CAS-103475-43-0
Score: 0.93 (14/15 C)
Deviation : 2.33 ppm

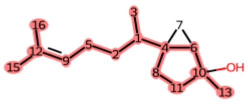

Rank: 186 MW: 222.37
Sesquisabinene hydrate
Score: 0.93 (14/15 C)
Deviation : 2.38 ppm

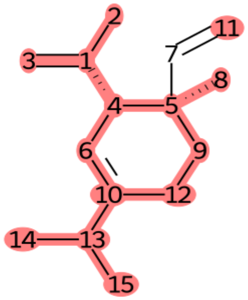

Rank: 187 MW: 204.35
delta-Elemene
Score: 0.93 (14/15 C)
Deviation : 2.41 ppm

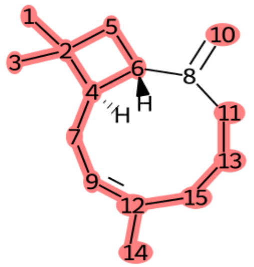

Rank: 188 MW: 204.35
CAS-136296-35-0
Score: 0.93 (14/15 C)
Deviation : 2.41 ppm

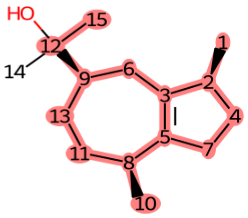

Rank: 189 MW: 222.37
(-)-Guaiol
Score: 0.93 (14/15 C)
Deviation : 2.42 ppm

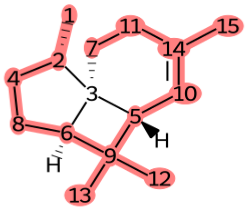

Rank: 190 MW: 204.35
(-)-Italicene
Score: 0.93 (14/15 C)
Deviation : 2.46 ppm

Figure S20. Dereplication analysis from MixONat, structure of dereplicated compounds: Rank **181-190**.

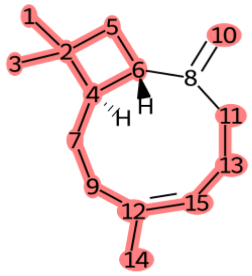

Rank: 191 MW: 204.35
(Z)-beta-Caryophyllene
Score: 0.93 (14/15 C)
Deviation : 2.49 ppm

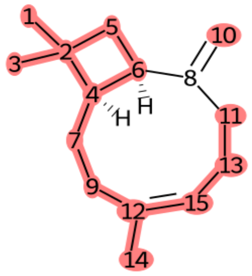

Rank: 192 MW: 204.35
CAS-68832-35-9
Score: 0.93 (14/15 C)
Deviation : 2.49 ppm

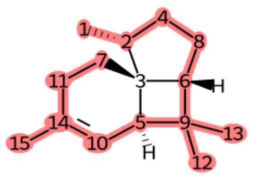

Rank: 193 MW: 204.35
(+)-Isoitalicene
Score: 0.93 (14/15 C)
Deviation : 2.5 ppm

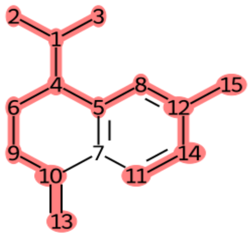

Rank: 194 MW: 200.32
beta-Calacorene
Score: 0.93 (14/15 C)
Deviation : 2.62 ppm

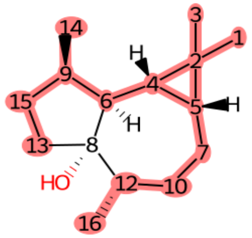

Rank: 195 MW: 222.37
Palustrol
Score: 0.93 (14/15 C)
Deviation : 2.63 ppm

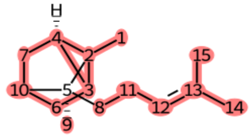

Rank: 196 MW: 204.35
(-)-alpha-Santalene
Score: 0.93 (14/15 C)
Deviation : 2.73 ppm

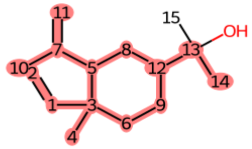

Rank: 197 MW: 222.37
CAS-19078-36-5
Score: 0.93 (14/15 C)
Deviation : 2.77 ppm

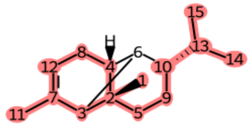

Rank: 198 MW: 204.35
(+)-alpha-Ylangene
Score: 0.93 (14/15 C)
Deviation : 2.82 ppm

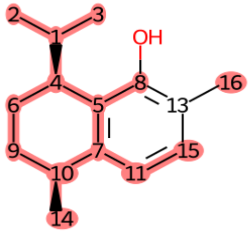

Rank: 199 MW: 218.33
CAS-89015-43-0
Score: 0.93 (14/15 C)
Deviation : 2.88 ppm

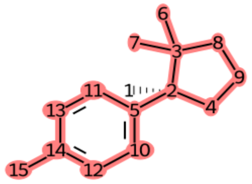

Rank: 200 MW: 202.34
(+)-Cuparene
Score: 0.93 (14/15 C)
Deviation : 2.92 ppm

Figure S21. Dereplication analysis from MixONat, structure of dereplicated compounds: Rank **191-200**.

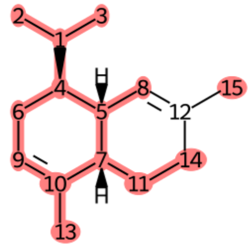

Rank: 201 MW: 204.35
(-)-alpha-Muurolene
Score: 0.93 (14/15 C)
Deviation : 2.98 ppm

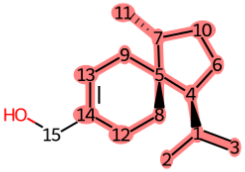

Rank: 202 MW: 220.35
beta-Acoradienol
Score: 0.93 (14/15 C)
Deviation : 3.08 ppm

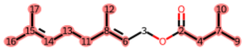

Rank: 203 MW: 238.37
Geraniol isovalerate
Score: 0.93 (14/15 C)
Deviation : 3.09 ppm

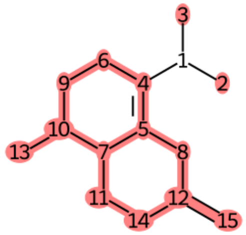

Rank: 204 MW: 204.35
CAS-150320-52-8
Score: 0.93 (14/15 C)
Deviation : 3.11 ppm

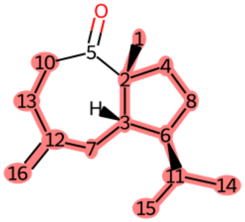

Rank: 205 MW: 220.35
Mintketone
Score: 0.93 (14/15 C)
Deviation : 3.14 ppm

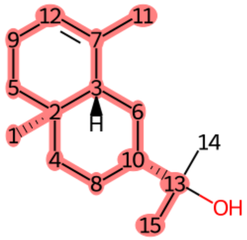

Rank: 206 MW: 222.37
(-)-alpha-Eudesmol
Score: 0.93 (14/15 C)
Deviation : 3.18 ppm

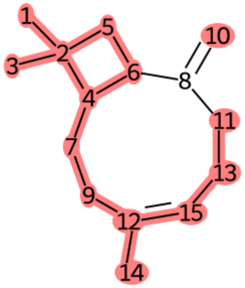

Rank: 207 MW: 204.35
CAS-13877-93-5
Score: 0.93 (14/15 C)
Deviation : 3.23 ppm

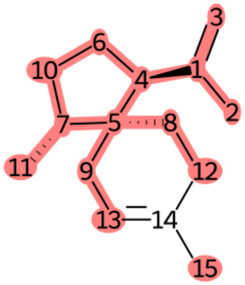

Rank: 208 MW: 204.35
(-)-alpha-Acoradiene
Score: 0.93 (14/15 C)
Deviation : 3.25 ppm

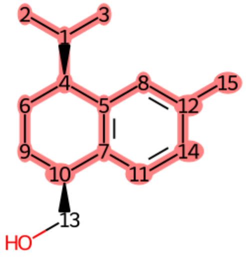

Rank: 209 MW: 218.33
trans-Calamenen-10-ol
Score: 0.93 (14/15 C)
Deviation : 3.27 ppm

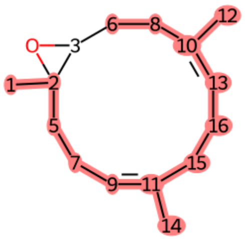

Rank: 210 MW: 220.35
Cedroxide
Score: 0.93 (14/15 C)
Deviation : 3.29 ppm

Figure S22. Dereplication analysis from MixONat, structure of dereplicated compounds: Rank **201-210**.

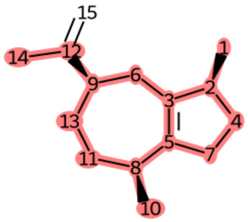

Rank: 211 MW: 204.35
alpha-Guaiene
Score: 0.93 (14/15 C)
Deviation : 3.31 ppm

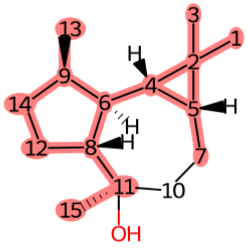

Rank: 212 MW: 222.37
(-)-Globulol
Score: 0.93 (14/15 C)
Deviation : 3.32 ppm

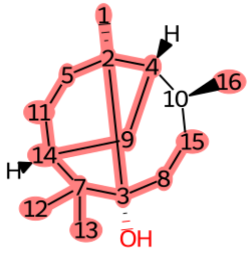

Rank: 213 MW: 222.37
(-)-Patchoulol
Score: 0.93 (14/15 C)
Deviation : 3.34 ppm

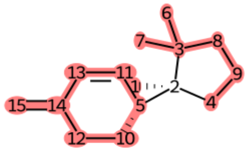

Rank: 214 MW: 204.35
(-)-delta-Cuprenene
Score: 0.93 (14/15 C)
Deviation : 3.34 ppm

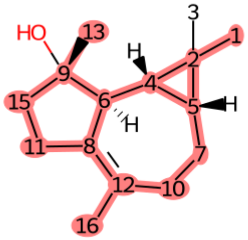

Rank: 215 MW: 220.35
(+)-Isospathulenol
Score: 0.93 (14/15 C)
Deviation : 3.35 ppm

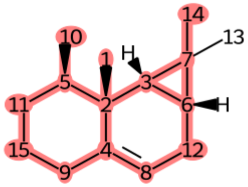

Rank: 216 MW: 204.35
(-)-Aristolene
Score: 0.93 (14/15 C)
Deviation : 3.36 ppm

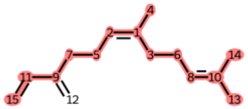

Rank: 217 MW: 204.35
(Z)-beta-Farnesene
Score: 0.93 (14/15 C)
Deviation : 3.42 ppm

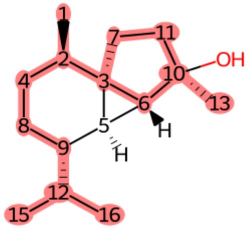

Rank: 218 MW: 222.37
4-Epicubebol
Score: 0.93 (14/15 C)
Deviation : 3.47 ppm

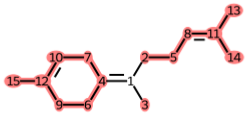

Rank: 219 MW: 204.35
(Z)-gamma-Bisabolene
Score: 0.93 (14/15 C)
Deviation : 3.48 ppm

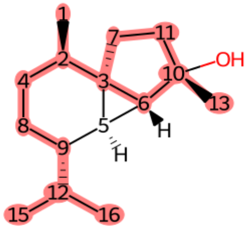

Rank: 220 MW: 222.37
(-)-Cubebol
Score: 0.93 (14/15 C)
Deviation : 3.52 ppm

Figure S23. Dereplication analysis from MixONat, structure of dereplicated compounds: Rank **211-220**.

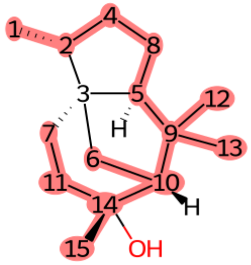

Rank: 221 MW: 222.37
(+)-Cedrol
Score: 0.93 (14/15 C)
Deviation : 3.57 ppm

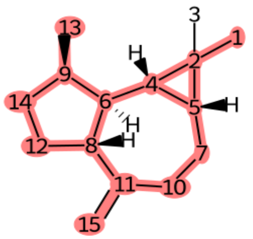

Rank: 222 MW: 204.35
beta-Aromadendrene
Score: 0.93 (14/15 C)
Deviation : 3.61 ppm

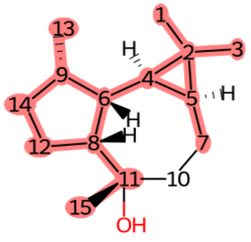

Rank: 223 MW: 222.37
(+)-Ledol
Score: 0.93 (14/15 C)
Deviation : 3.64 ppm

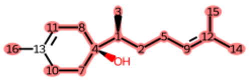

Rank: 224 MW: 222.37
beta-Bisabolol
Score: 0.93 (14/15 C)
Deviation : 3.67 ppm

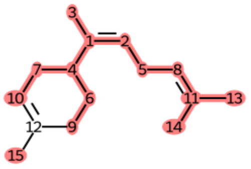

Rank: 225 MW: 204.35
(Z)-alpha-Bisabolene
Score: 0.93 (14/15 C)
Deviation : 3.72 ppm

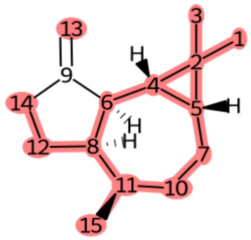

Rank: 226 MW: 204.35
beta-Gurjunene
Score: 0.93 (14/15 C)
Deviation : 3.83 ppm

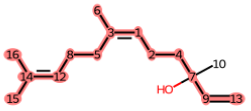

Rank: 227 MW: 222.37
(Z)-Nerolidol
Score: 0.93 (14/15 C)
Deviation : 3.84 ppm

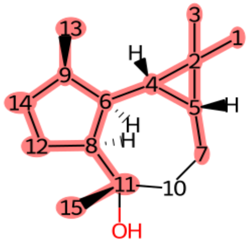

Rank: 228 MW: 222.37
(+)-Viridiflorol
Score: 0.93 (14/15 C)
Deviation : 3.85 ppm

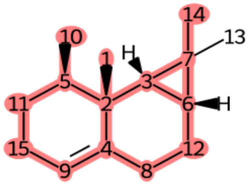

Rank: 229 MW: 204.35
(+)-Calarene
Score: 0.93 (14/15 C)
Deviation : 3.87 ppm

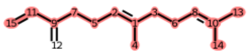

Rank: 230 MW: 204.35
(E)-beta-Farnesene
Score: 0.93 (14/15 C)
Deviation : 3.88 ppm

Figure S24. Dereplication analysis from MixONat, structure of dereplicated compounds: Rank **221-230**.

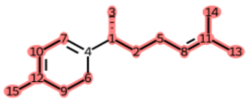

Rank: 231 MW: 204.35
gamma-Curcumene
Score: 0.93 (14/15 C)
Deviation : 3.88 ppm

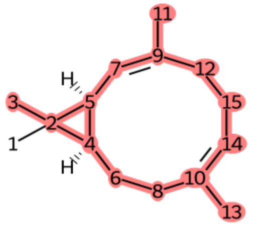

Rank: 232 MW: 204.35
(+)-Bicyclogermacrene
Score: 0.93 (14/15 C)
Deviation : 3.88 ppm

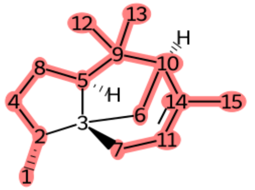

Rank: 233 MW: 204.35
(-)-alpha-Funebrene
Score: 0.93 (14/15 C)
Deviation : 3.93 ppm

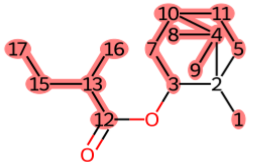

Rank: 234 MW: 238.37
CAS-94200-10-9
Score: 0.93 (14/15 C)
Deviation : 4.03 ppm

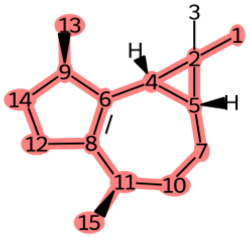

Rank: 235 MW: 204.35
(-)-Isoledene
Score: 0.93 (14/15 C)
Deviation : 4.03 ppm

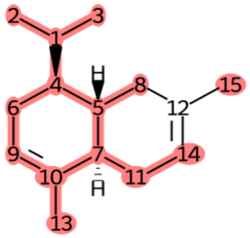

Rank: 236 MW: 204.35
(-)-beta-Cadinene
Score: 0.93 (14/15 C)
Deviation : 4.1 ppm

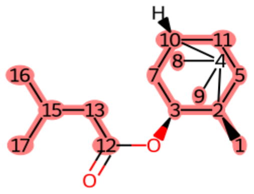

Rank: 237 MW: 238.37
Isobornyl isovalerate
Score: 0.93 (14/15 C)
Deviation : 4.13 ppm

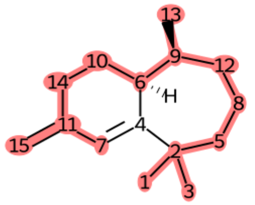

Rank: 238 MW: 204.35
CAS-60909-28-6
Score: 0.93 (14/15 C)
Deviation : 4.13 ppm

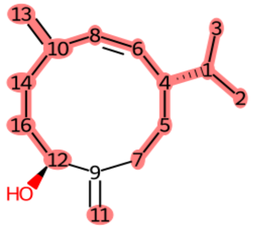

Rank: 239 MW: 220.35
CAS-70191-49-0
Score: 0.93 (14/15 C)
Deviation : 4.16 ppm

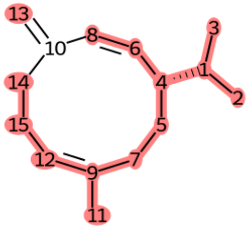

Rank: 240 MW: 204.35
(-)-Germacrene D
Score: 0.93 (14/15 C)
Deviation : 4.23 ppm

Figure S25. Dereplication analysis from MixONat, structure of dereplicated compounds: Rank **231-240**.

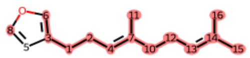

Rank: 241 MW: 218.33
Dendrolasin
Score: 0.93 (14/15 C)
Deviation : 4.23 ppm

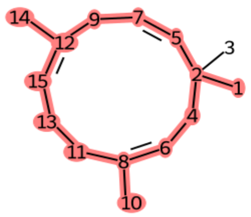

Rank: 242 MW: 204.35
alpha-Humulene
Score: 0.93 (14/15 C)
Deviation : 4.34 ppm

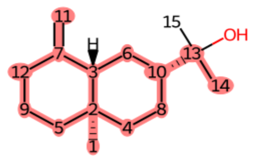

Rank: 243 MW: 222.37
(+)-beta-Eudesmol
Score: 0.93 (14/15 C)
Deviation : 4.34 ppm

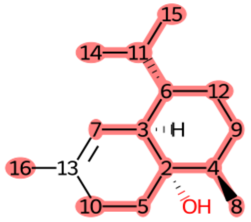

Rank: 244 MW: 222.37
Epicubenol
Score: 0.93 (14/15 C)
Deviation : 4.36 ppm

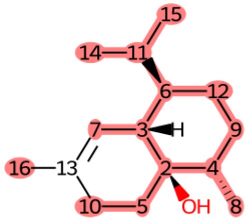

Rank: 245 MW: 222.37
(+)-Epicubenol
Score: 0.93 (14/15 C)
Deviation : 4.36 ppm

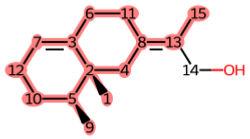

Rank: 246 MW: 220.35
Bicyclovetivenol
Score: 0.93 (14/15 C)
Deviation : 4.41 ppm

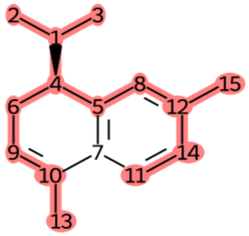

Rank: 247 MW: 200.32
(+)-alpha-Calacorene
Score: 0.93 (14/15 C)
Deviation : 4.41 ppm

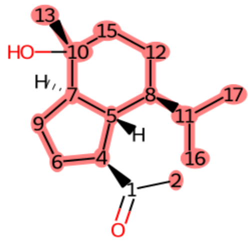

Rank: 248 MW: 238.37
(-)-Oplopanone
Score: 0.93 (14/15 C)
Deviation : 4.44 ppm

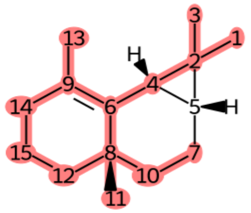

Rank: 249 MW: 204.35
beta-Maaliene
Score: 0.93 (14/15 C)
Deviation : 4.45 ppm

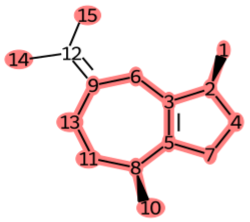

Rank: 250 MW: 204.35
beta-Guaiene
Score: 0.93 (14/15 C)
Deviation : 4.58 ppm

Figure S26. Dereplication analysis from MixONat, structure of dereplicated compounds: Rank **241-250**.

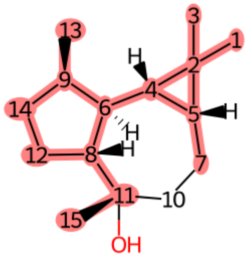

Rank: 251 MW: 222.37
(-)-Epiglobulol
Score: 0.93 (14/15 C)
Deviation : 4.8 ppm

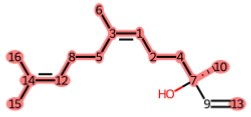

Rank: 252 MW: 222.37
(+)-Nerolidol
Score: 0.93 (14/15 C)
Deviation : 4.84 ppm

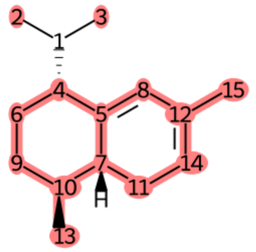

Rank: 253 MW: 204.35
CAS-262352-88-5
Score: 0.93 (14/15 C)
Deviation : 5.03 ppm

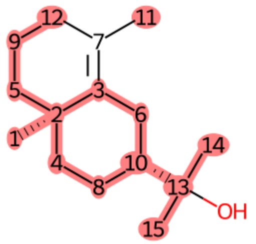

Rank: 254 MW: 222.37
(+)-gamma-Eudesmol
Score: 0.93 (14/15 C)
Deviation : 5.13 ppm

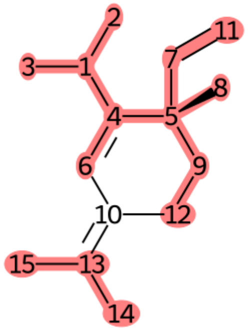

Rank: 255 MW: 204.35
(+)-alpha-Elemene
Score: 0.93 (14/15 C)
Deviation : 5.21 ppm

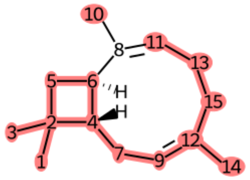

Rank: 256 MW: 204.35
CAS-2146092-14-8
Score: 0.93 (14/15 C)
Deviation : 5.38 ppm

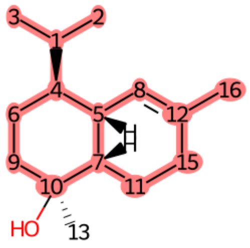

Rank: 257 MW: 222.37
(-)-T-Muurolol
Score: 0.93 (14/15 C)
Deviation : 5.46 ppm

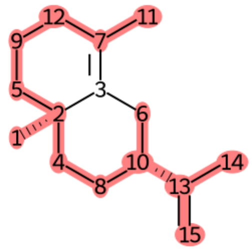

Rank: 258 MW: 204.35
alpha-Cyperene
Score: 0.93 (14/15 C)
Deviation : 5.67 ppm

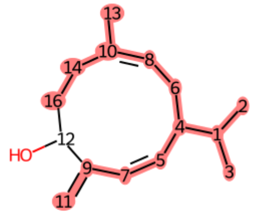

Rank: 259 MW: 220.35
CAS-129927-17-9
Score: 0.93 (14/15 C)
Deviation : 5.88 ppm

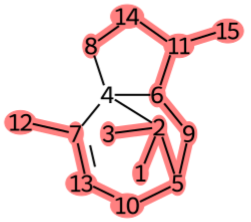

Rank: 260 MW: 204.35
alpha-Patchoulene
Score: 0.93 (14/15 C)
Deviation : 5.99 ppm

Figure S27. Dereplication analysis from MixONat, structure of dereplicated compounds: Rank **251-260**.

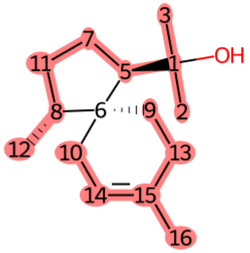

Rank: 261 MW: 222.37
(-)-alpha-Acorenol
Score: 0.93 (14/15 C)
Deviation : 6.3 ppm

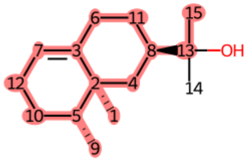

Rank: 262 MW: 222.37
(+)-Valerianol
Score: 0.93 (14/15 C)
Deviation : 6.35 ppm

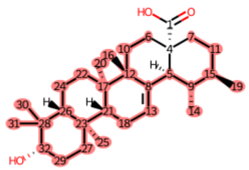

Rank: 263 MW: 456.7
(+)-Ursolic acid
Score: 0.93 (28/30 C)
Deviation : 6.97 ppm

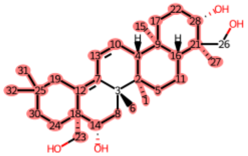

Rank: 264 MW: 472.7
Saikogenin A
Score: 0.93 (28/30 C)
Deviation : 7.14 ppm

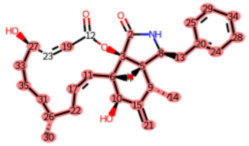

Rank: 265 MW: 479.61
Cytochalasin B
Score: 0.93 (27/29 C)
Deviation : 8.62 ppm

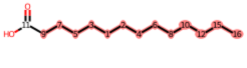

Rank: 266 MW: 228.37
Tetradecanoic acid
Score: 0.93 (13/14 C)
Deviation : 0.82 ppm

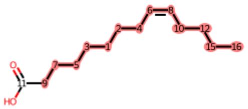

Rank: 267 MW: 226.35
Myristoleic Acid
Score: 0.93 (13/14 C)
Deviation : 0.84 ppm

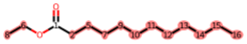

Rank: 268 MW: 228.37
CAS-106-33-2
Score: 0.93 (13/14 C)
Deviation : 1.78 ppm

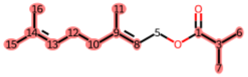

Rank: 269 MW: 224.34
Geranyl isobutanoate
Score: 0.93 (13/14 C)
Deviation : 3.05 ppm

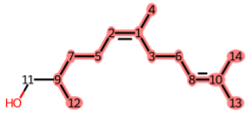

Rank: 270 MW: 210.36
(Z)-Dihydroapofarnesol
Score: 0.93 (13/14 C)
Deviation : 3.25 ppm

Figure S28. Dereplication analysis from MixONat, structure of dereplicated compounds: Rank **261-270**.

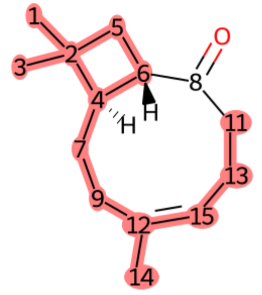

Rank: 271 MW: 206.32
CAS-60362-45-0
Score: 0.93 (13/14 C)
Deviation : 3.76 ppm

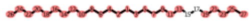

Rank: 272 MW: 380.73
Heptacosane
Score: 0.93 (25/27 C)
Deviation : 8.24 ppm

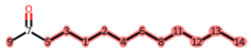

Rank: 273 MW: 198.34
2-Tridecanone
Score: 0.92 (12/13 C)
Deviation : 1.78 ppm

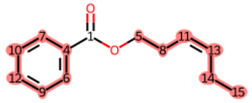

Rank: 274 MW: 204.26
cis-3-Hexenyl benzoate
Score: 0.92 (12/13 C)
Deviation : 2.03 ppm

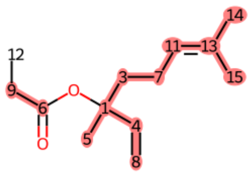

Rank: 275 MW: 210.31
Linalool propionate
Score: 0.92 (12/13 C)
Deviation : 3.08 ppm

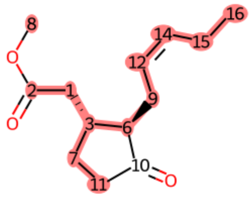

Rank: 276 MW: 224.3
(-)-Methyl jasmonate
Score: 0.92 (12/13 C)
Deviation : 3.18 ppm

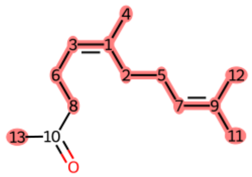

Rank: 277 MW: 194.31
cis-Geranylacetone
Score: 0.92 (12/13 C)
Deviation : 3.62 ppm

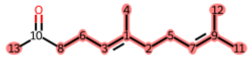

Rank: 278 MW: 194.31
trans-Geranylacetone
Score: 0.92 (12/13 C)
Deviation : 4.04 ppm

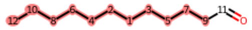

Rank: 279 MW: 184.32
Dodecanal
Score: 0.92 (11/12 C)
Deviation : 0.7 ppm

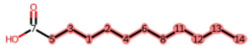

Rank: 280 MW: 200.32
Dodecanoic acid
Score: 0.92 (11/12 C)
Deviation : 0.73 ppm

Figure S29. Dereplication analysis from MixONat, structure of dereplicated compounds: Rank **271-280**.

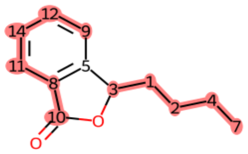

Rank: 281 MW: 190.24
3-n-Butylphthalide
Score: 0.92 (11/12 C)
Deviation : 1.33 ppm

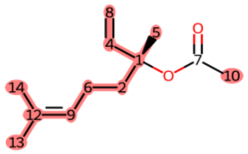

Rank: 282 MW: 196.29
(-)-Linalyl acetate
Score: 0.92 (11/12 C)
Deviation : 1.41 ppm

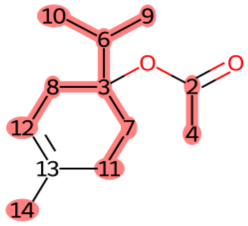

Rank: 283 MW: 196.29
4-Terpineol acetate
Score: 0.92 (11/12 C)
Deviation : 1.53 ppm

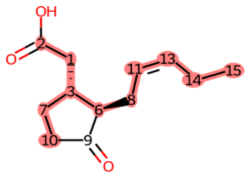

Rank: 284 MW: 210.27
(-)-Jasmonic acid
Score: 0.92 (11/12 C)
Deviation : 1.68 ppm

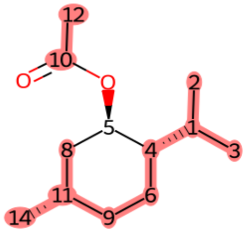

Rank: 285 MW: 198.3
Isomenthol acetate
Score: 0.92 (11/12 C)
Deviation : 1.75 ppm

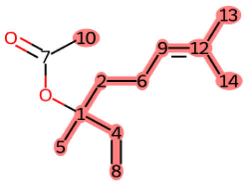

Rank: 286 MW: 196.29
Linalyl acetate
Score: 0.92 (11/12 C)
Deviation : 1.8 ppm

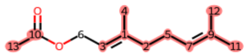

Rank: 287 MW: 196.29
trans-Geranyl acetate
Score: 0.92 (11/12 C)
Deviation : 1.9 ppm

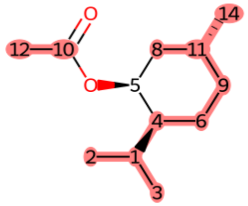

Rank: 288 MW: 198.3
Neomenthol acetate
Score: 0.92 (11/12 C)
Deviation : 2.04 ppm

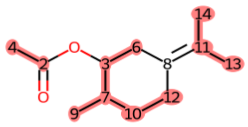

Rank: 289 MW: 196.29
CAS-13461-20-6
Score: 0.92 (11/12 C)
Deviation : 2.39 ppm

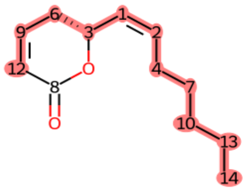

Rank: 290 MW: 194.27
(-)-Argentilactone
Score: 0.92 (11/12 C)
Deviation : 2.8 ppm

Figure S30. Dereplication analysis from MixONat, structure of dereplicated compounds: Rank **281-290**.

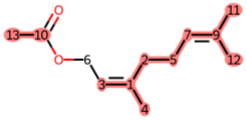

Rank: 291 MW: 196.29
Neryl acetate
Score: 0.92 (11/12 C)
Deviation : 2.84 ppm

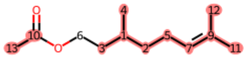

Rank: 292 MW: 198.3
Citronellyl acetate
Score: 0.92 (11/12 C)
Deviation : 2.85 ppm

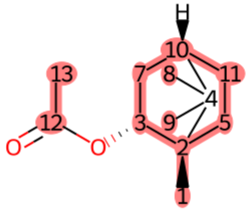

Rank: 293 MW: 196.29
(-)-Borneol acetate
Score: 0.92 (11/12 C)
Deviation : 3.31 ppm

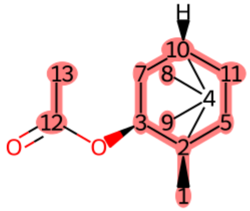

Rank: 294 MW: 196.29
Isobornyl acetate
Score: 0.92 (11/12 C)
Deviation : 3.51 ppm

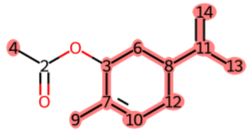

Rank: 295 MW: 194.27
Carveol acetate
Score: 0.92 (11/12 C)
Deviation : 3.57 ppm

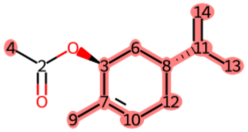

Rank: 296 MW: 194.27
trans-Carveyl acetate
Score: 0.92 (11/12 C)
Deviation : 3.57 ppm

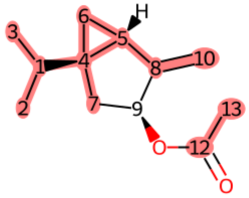

Rank: 297 MW: 194.27
trans-Sabinyl acetate
Score: 0.92 (11/12 C)
Deviation : 4.12 ppm

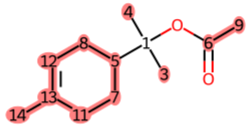

Rank: 298 MW: 196.29
alpha-Terpinyl acetate
Score: 0.92 (11/12 C)
Deviation : 4.23 ppm

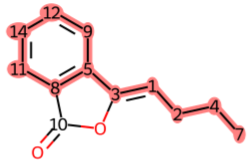

Rank: 299 MW: 188.22
3-Butylidenephthalide
Score: 0.92 (11/12 C)
Deviation : 4.79 ppm

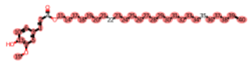

Rank: 300 MW: 558.88
Hexacosyl (E)-ferulate
Score: 0.92 (33/36 C)
Deviation : 7.79 ppm

Figure S31. Dereplication analysis from MixONat, structure of dereplicated compounds: Rank **291-300**.

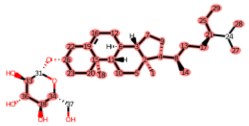

Rank: 301 MW: 576.85
beta-Daucosterol
Score: 0.91 (32/35 C)
Deviation : 7.7 ppm

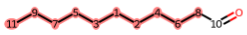

Rank: 302 MW: 170.29
Undecanal
Score: 0.91 (10/11 C)
Deviation : 0.76 ppm

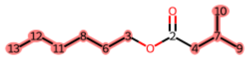

Rank: 303 MW: 186.29
n-Hexyl isovalerate
Score: 0.91 (10/11 C)
Deviation : 1.25 ppm

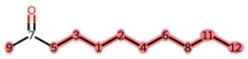

Rank: 304 MW: 170.29
2-Undecanone
Score: 0.91 (10/11 C)
Deviation : 1.73 ppm

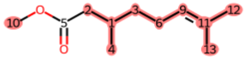

Rank: 305 MW: 184.28
Methyl citronellate
Score: 0.91 (10/11 C)
Deviation : 1.96 ppm

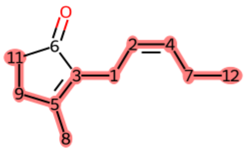

Rank: 306 MW: 164.24
Jasmone
Score: 0.91 (10/11 C)
Deviation : 2.51 ppm

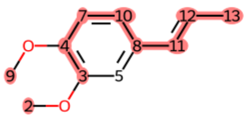

Rank: 307 MW: 178.23
Isohomoeugenol
Score: 0.91 (10/11 C)
Deviation : 3.72 ppm

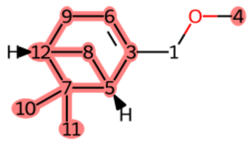

Rank: 308 MW: 166.26
Myrtenol methyl ether
Score: 0.91 (10/11 C)
Deviation : 3.91 ppm

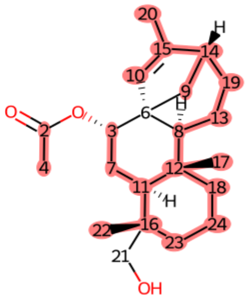

Rank: 309 MW: 346.5
Siderol
Score: 0.91 (20/22 C)
Deviation : 4.98 ppm

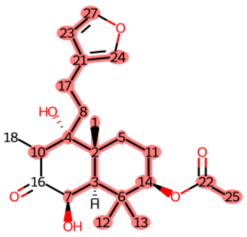

Rank: 310 MW: 392.49
(-)-Leosibirin
Score: 0.91 (20/22 C)
Deviation : 5.94 ppm

Figure S32. Dereplication analysis from MixONat, structure of dereplicated compounds: Rank **301-310**.

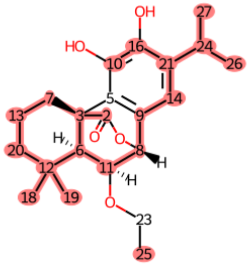

Rank: 311 MW: 374.47
CAS-177027-96-2
Score: 0.91 (20/22 C)
Deviation : 7.73 ppm

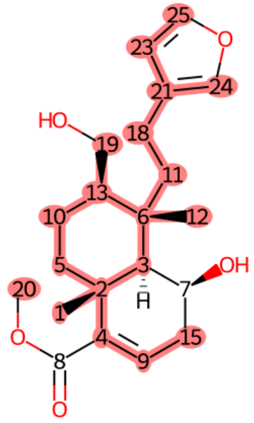

Rank: 312 MW: 362.46
Divinatorin B
Score: 0.9 (19/21 C)
Deviation : 7.39 ppm

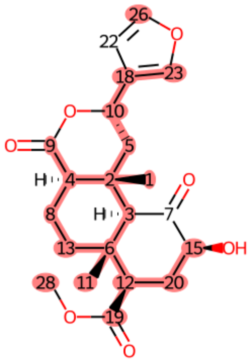

Rank: 313 MW: 390.43
(-)-Salvinorin B
Score: 0.9 (19/21 C)
Deviation : 9.23 ppm

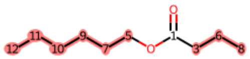

Rank: 314 MW: 172.26
Hexyl butanoate
Score: 0.9 (9/10 C)
Deviation : 0.39 ppm

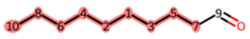

Rank: 315 MW: 156.27
Decanal
Score: 0.9 (9/10 C)
Deviation : 0.57 ppm

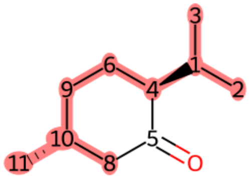

Rank: 316 MW: 154.25
Menthone
Score: 0.9 (9/10 C)
Deviation : 0.92 ppm

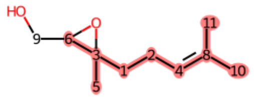

Rank: 317 MW: 170.25
CAS-50727-94-1
Score: 0.9 (9/10 C)
Deviation : 1.08 ppm

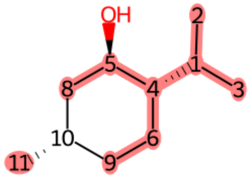

Rank: 318 MW: 156.27
Isomenthol
Score: 0.9 (9/10 C)
Deviation : 1.11 ppm

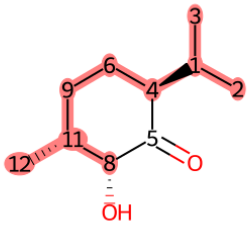

Rank: 319 MW: 170.25
CAS-128946-15-6
Score: 0.9 (9/10 C)
Deviation : 1.23 ppm

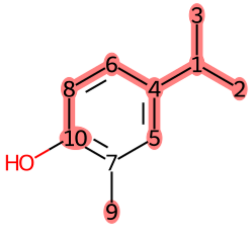

Rank: 320 MW: 150.22
Isocarvacrol
Score: 0.9 (9/10 C)
Deviation : 1.24 ppm

Figure S33. Dereplication analysis from MixONat, structure of dereplicated compounds: Rank **311-320**.

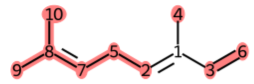

Rank: 321 MW: 136.23
beta-Ocimene
Score: 0.9 (9/10 C)
Deviation : 1.28 ppm

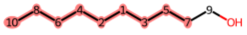

Rank: 322 MW: 158.28
1-Decanol
Score: 0.9 (9/10 C)
Deviation : 1.34 ppm

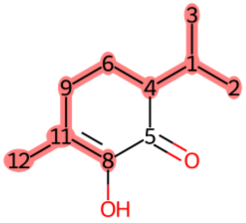

Rank: 323 MW: 168.23
Diosphenol
Score: 0.9 (9/10 C)
Deviation : 1.43 ppm

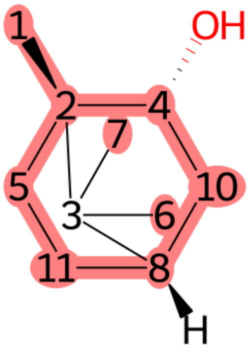

Rank: 324 MW: 154.25
(-)-Borneol
Score: 0.9 (9/10 C)
Deviation : 1.48 ppm

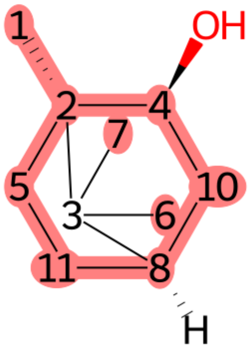

Rank: 325 MW: 154.25
Borneol
Score: 0.9 (9/10 C)
Deviation : 1.48 ppm

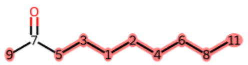

Rank: 326 MW: 156.27
2-Decanone
Score: 0.9 (9/10 C)
Deviation : 1.48 ppm

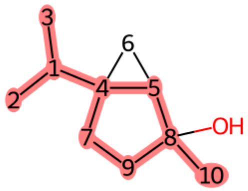

Rank: 327 MW: 154.25
Sabinene hydrate
Score: 0.9 (9/10 C)
Deviation : 1.64 ppm

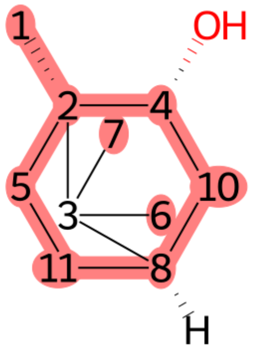

Rank: 328 MW: 154.25
Isoborneol
Score: 0.9 (9/10 C)
Deviation : 1.66 ppm

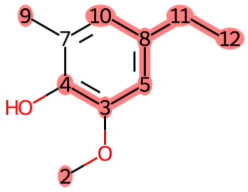

Rank: 329 MW: 166.22
CAS-120550-70-1
Score: 0.9 (9/10 C)
Deviation : 1.76 ppm

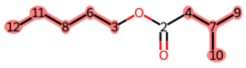

Rank: 330 MW: 172.26
Pentyl isopentanoate
Score: 0.9 (9/10 C)
Deviation : 1.77 ppm

Figure S34. Dereplication analysis from MixONat, structure of dereplicated compounds: Rank **321-330**.

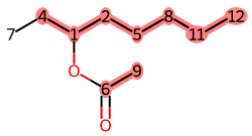

Rank: 331 MW: 172.26
3-Octyl acetate
Score: 0.9 (9/10 C)
Deviation : 1.8 ppm

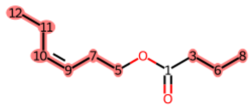

Rank: 332 MW: 170.25
(3Z)-Hexenyl butanoate
Score: 0.9 (9/10 C)
Deviation : 1.91 ppm

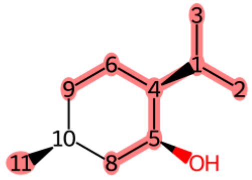

Rank: 333 MW: 156.27
Neoisomenthol
Score: 0.9 (9/10 C)
Deviation : 1.96 ppm

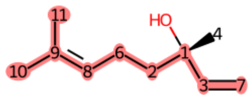

Rank: 334 MW: 154.25
Coriandrol
Score: 0.9 (9/10 C)
Deviation : 2.02 ppm

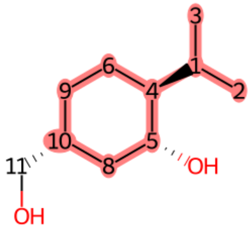

Rank: 335 MW: 172.26
(+)-7-Hydroxymenthol
Score: 0.9 (9/10 C)
Deviation : 2.06 ppm

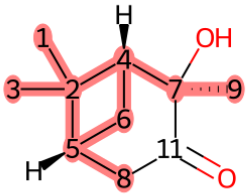

Rank: 336 MW: 168.23
CAS-24047-72-1
Score: 0.9 (9/10 C)
Deviation : 2.08 ppm

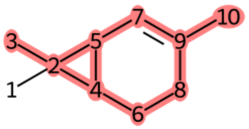

Rank: 337 MW: 136.23
2-Carene
Score: 0.9 (9/10 C)
Deviation : 2.14 ppm

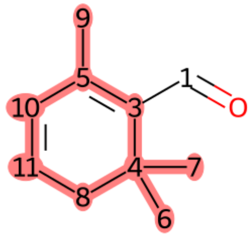

Rank: 338 MW: 150.22
Safranal
Score: 0.9 (9/10 C)
Deviation : 2.15 ppm

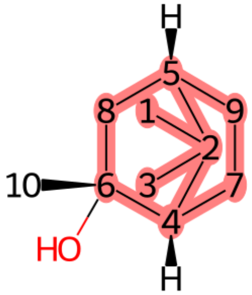

Rank: 339 MW: 154.25
trans-Pinene hydrate
Score: 0.9 (9/10 C)
Deviation : 2.15 ppm

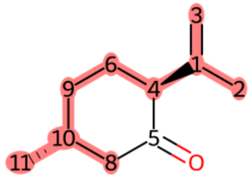

Rank: 340 MW: 152.23
Isopulegone
Score: 0.9 (9/10 C)
Deviation : 2.17 ppm

Figure S35. Dereplication analysis from MixONat, structure of dereplicated compounds: Rank **331-340**.

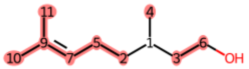

Rank: 341 MW: 156.27
Citronellol
Score: 0.9 (9/10 C)
Deviation : 2.21 ppm

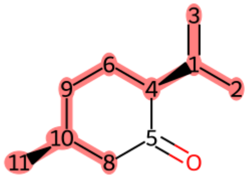

Rank: 342 MW: 152.23
cis-Isopulegone
Score: 0.9 (9/10 C)
Deviation : 2.22 ppm

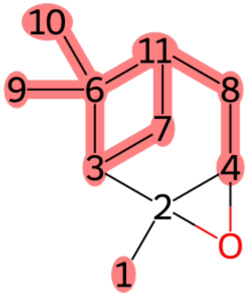

Rank: 343 MW: 152.23
alpha-Pinene epoxide
Score: 0.9 (9/10 C)
Deviation : 2.32 ppm

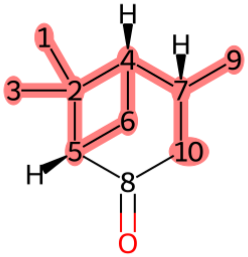

Rank: 344 MW: 152.23
cis-Verbanone
Score: 0.9 (9/10 C)
Deviation : 2.32 ppm

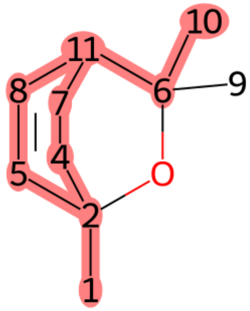

Rank: 345 MW: 152.23
Dehydrocineole
Score: 0.9 (9/10 C)
Deviation : 2.35 ppm

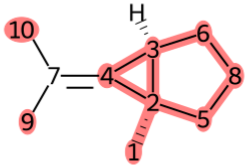

Rank: 346 MW: 136.23
CAS-24524-57-0
Score: 0.9 (9/10 C)
Deviation : 2.36 ppm

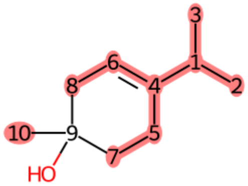

Rank: 347 MW: 154.25
1-Terpineol
Score: 0.9 (9/10 C)
Deviation : 2.38 ppm

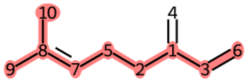

Rank: 348 MW: 136.23
beta-Myrcene
Score: 0.9 (9/10 C)
Deviation : 2.46 ppm

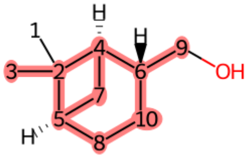

Rank: 349 MW: 154.25
trans-Myrtanol
Score: 0.9 (9/10 C)
Deviation : 2.52 ppm

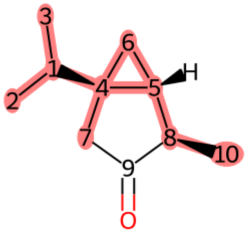

Rank: 350 MW: 152.23
(-)-Thujone
Score: 0.9 (9/10 C)
Deviation : 2.53 ppm

Figure S36. Dereplication analysis from MixONat, structure of dereplicated compounds: Rank **341-350**.

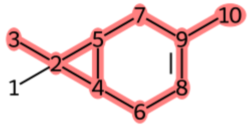

Rank: 351 MW: 136.23
3-Carene
Score: 0.9 (9/10 C)
Deviation : 2.54 ppm

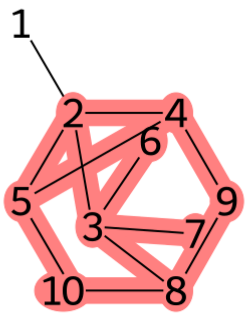

Rank: 352 MW: 136.23
Tricyclene
Score: 0.9 (9/10 C)
Deviation : 2.56 ppm

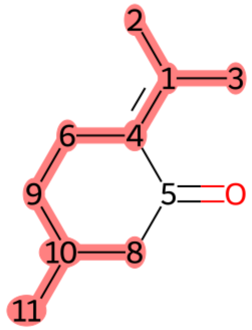

Rank: 353 MW: 152.23
Pulegone
Score: 0.9 (9/10 C)
Deviation : 2.57 ppm

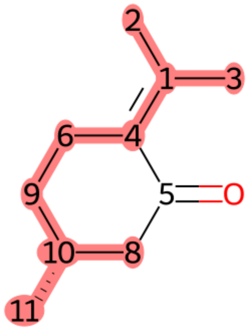

Rank: 354 MW: 152.23
(+)-Pulegone
Score: 0.9 (9/10 C)
Deviation : 2.62 ppm

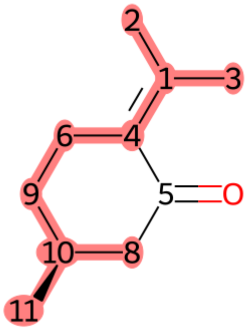

Rank: 355 MW: 152.23
(-)-Pulegone
Score: 0.9 (9/10 C)
Deviation : 2.62 ppm

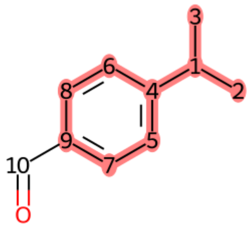

Rank: 356 MW: 148.2
Cuminyl aldehyde
Score: 0.9 (9/10 C)
Deviation : 2.65 ppm

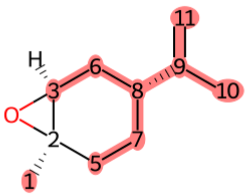

Rank: 357 MW: 152.23
cis-Limonene epoxide
Score: 0.9 (9/10 C)
Deviation : 2.65 ppm

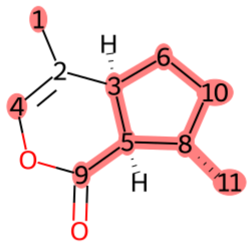

Rank: 358 MW: 166.22
CAS-21651-62-7
Score: 0.9 (9/10 C)
Deviation : 2.65 ppm

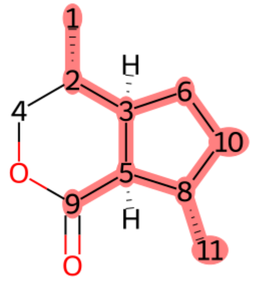

Rank: 359 MW: 168.23
CAS-17672-96-7
Score: 0.9 (9/10 C)
Deviation : 2.67 ppm

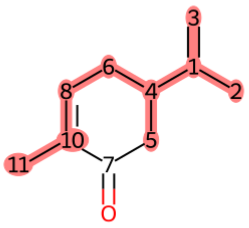

Rank: 360 MW: 150.22
Carvone
Score: 0.9 (9/10 C)
Deviation : 2.7 ppm

Figure S37. Dereplication analysis from MixONat, structure of dereplicated compounds: Rank **351-360**.

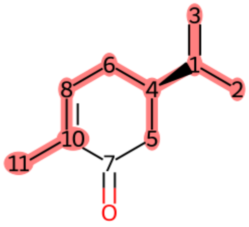

Rank: 361 MW: 150.22
(-)-Carvone
Score: 0.9 (9/10 C)
Deviation : 2.7 ppm

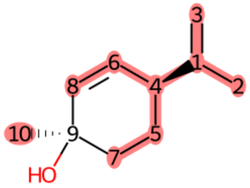

Rank: 362 MW: 152.23
CAS-3886-78-0
Score: 0.9 (9/10 C)
Deviation : 2.73 ppm

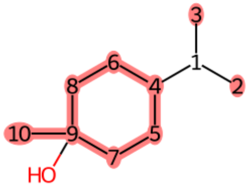

Rank: 363 MW: 156.27
Dihydroterpineol
Score: 0.9 (9/10 C)
Deviation : 2.76 ppm

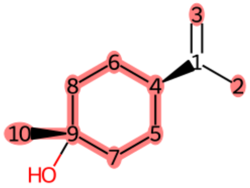

Rank: 364 MW: 154.25
cis-beta-Terpineol
Score: 0.9 (9/10 C)
Deviation : 2.81 ppm

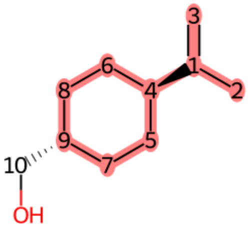

Rank: 365 MW: 154.25
trans-Shisool
Score: 0.9 (9/10 C)
Deviation : 2.87 ppm

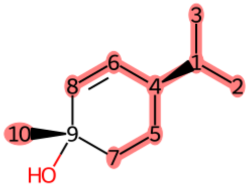

Rank: 366 MW: 154.25
cis-p-Menth-2-en-1-ol
Score: 0.9 (9/10 C)
Deviation : 2.88 ppm

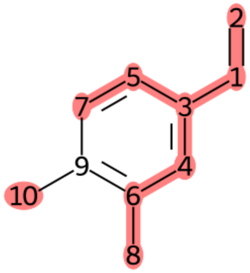

Rank: 367 MW: 132.2
3,4-Dimethylstyrene
Score: 0.9 (9/10 C)
Deviation : 2.89 ppm

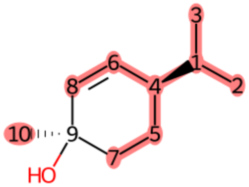

Rank: 368 MW: 154.25
cis-2-Menthenol
Score: 0.9 (9/10 C)
Deviation : 2.91 ppm

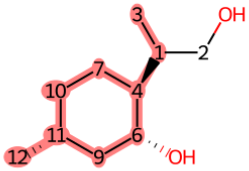

Rank: 369 MW: 172.26
CAS-200573-67-7
Score: 0.9 (9/10 C)
Deviation : 2.99 ppm

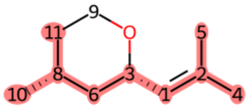

Rank: 370 MW: 154.25
cis-Rose oxide
Score: 0.9 (9/10 C)
Deviation : 3.01 ppm

Figure S38. Dereplication analysis from MixONat, structure of dereplicated compounds: Rank **361-370**.

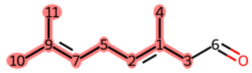

Rank: 371 MW: 152.23
trans-Isocritral
Score: 0.9 (9/10 C)
Deviation : 3.04 ppm

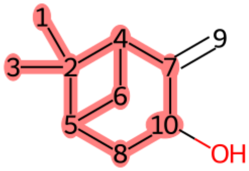

Rank: 372 MW: 152.23
Pinocarveol
Score: 0.9 (9/10 C)
Deviation : 3.11 ppm

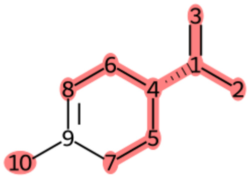

Rank: 373 MW: 136.23
(+)-Limonene
Score: 0.9 (9/10 C)
Deviation : 3.16 ppm

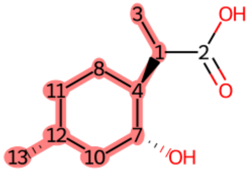

Rank: 374 MW: 186.25
CAS-945655-94-7
Score: 0.9 (9/10 C)
Deviation : 3.16 ppm

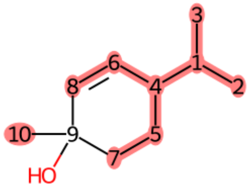

Rank: 375 MW: 154.25
2-Menthen-1-ol
Score: 0.9 (9/10 C)
Deviation : 3.17 ppm

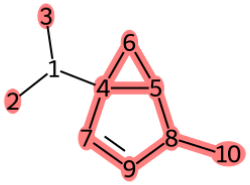

Rank: 376 MW: 136.23
beta-Thujene
Score: 0.9 (9/10 C)
Deviation : 3.17 ppm

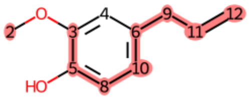

Rank: 377 MW: 164.2
p-Eugenol
Score: 0.9 (9/10 C)
Deviation : 3.18 ppm

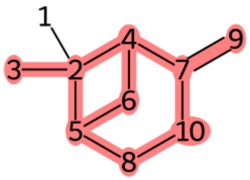

Rank: 378 MW: 136.23
beta-Pinene
Score: 0.9 (9/10 C)
Deviation : 3.22 ppm

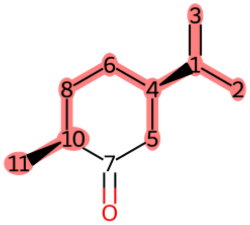

Rank: 379 MW: 152.23
cis-Dihydrocarvone
Score: 0.9 (9/10 C)
Deviation : 3.28 ppm

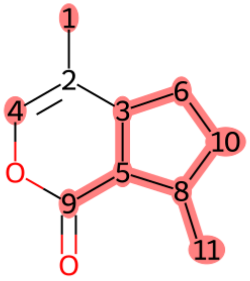

Rank: 380 MW: 166.22
Nepetalactone
Score: 0.9 (9/10 C)
Deviation : 3.3 ppm

Figure S39. Dereplication analysis from MixONat, structure of dereplicated compounds: Rank **371-380**.

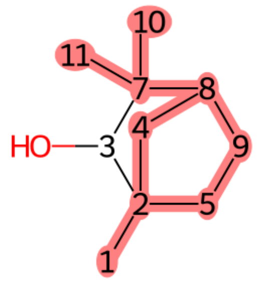

Rank: 381 MW: 154.25
Fenchol
Score: 0.9 (9/10 C)
Deviation : 3.31 ppm

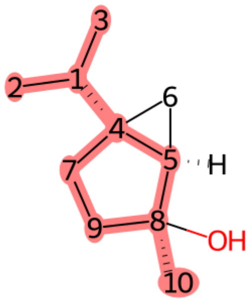

Rank: 382 MW: 154.25
cis-Sabinene hydrate
Score: 0.9 (9/10 C)
Deviation : 3.32 ppm

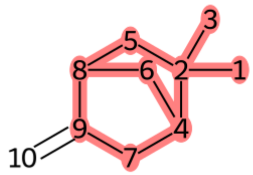

Rank: 383 MW: 136.23
beta-Fenchene
Score: 0.9 (9/10 C)
Deviation : 3.33 ppm

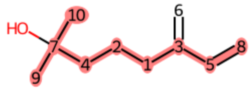

Rank: 384 MW: 154.25
Myrcenol
Score: 0.9 (9/10 C)
Deviation : 3.33 ppm

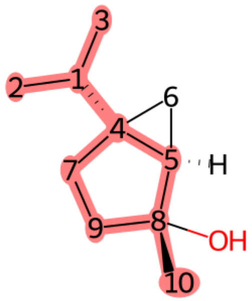

Rank: 385 MW: 154.25
trans-Sabinene hydrate
Score: 0.9 (9/10 C)
Deviation : 3.43 ppm

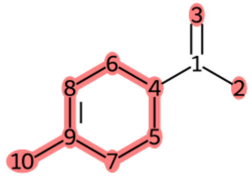

Rank: 386 MW: 136.23
Limonene
Score: 0.9 (9/10 C)
Deviation : 3.48 ppm

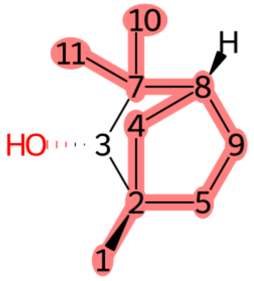

Rank: 387 MW: 154.25
alpha-Fenchol
Score: 0.9 (9/10 C)
Deviation : 3.51 ppm

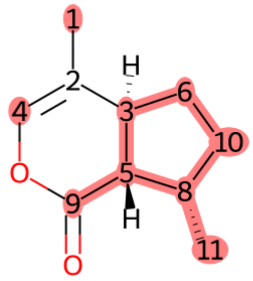

Rank: 388 MW: 166.22
Isonepetalactone
Score: 0.9 (9/10 C)
Deviation : 3.69 ppm

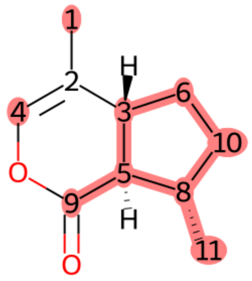

Rank: 389 MW: 166.22
CAS-21651-48-9
Score: 0.9 (9/10 C)
Deviation : 3.73 ppm

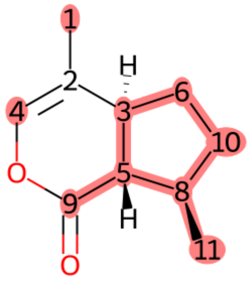

Rank: 390 MW: 166.22
CAS-108944-20-3
Score: 0.9 (9/10 C)
Deviation : 3.73 ppm

Figure S40. Dereplication analysis from MixONat, structure of dereplicated compounds: Rank **381-390**.

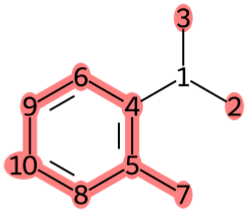

Rank: 391 MW: 134.22
o-Cymene
Score: 0.9 (9/10 C)
Deviation : 3.77 ppm

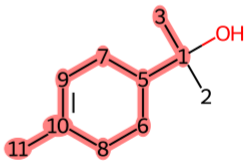

Rank: 392 MW: 154.25
alpha-Terpineol
Score: 0.9 (9/10 C)
Deviation : 3.85 ppm

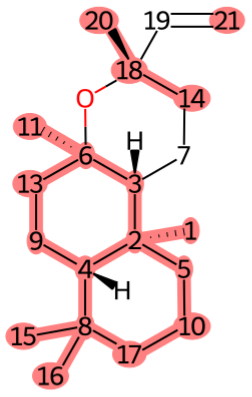

Rank: 393 MW: 290.48
(+)-13-Epimanoyl oxide
Score: 0.9 (18/20 C)
Deviation : 3.86 ppm

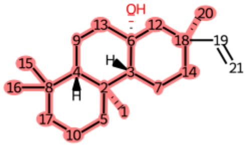

Rank: 394 MW: 290.48
Nejukol
Score: 0.9 (18/20 C)
Deviation : 3.99 ppm

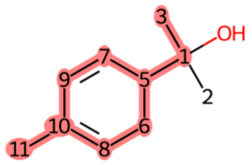

Rank: 395 MW: 152.23
alpha-Phellandren-8-ol
Score: 0.9 (9/10 C)
Deviation : 4.04 ppm

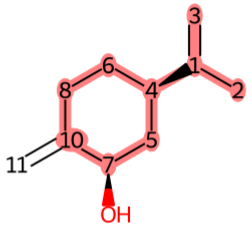

Rank: 396 MW: 152.23
CAS-22626-43-3
Score: 0.9 (9/10 C)
Deviation : 4.05 ppm

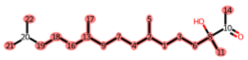

Rank: 397 MW: 312.53
Crassifol
Score: 0.9 (18/20 C)
Deviation : 4.06 ppm

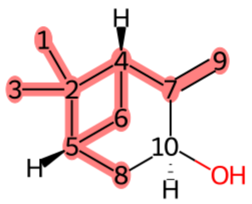

Rank: 398 MW: 152.23
trans-Pinocarveol
Score: 0.9 (9/10 C)
Deviation : 4.1 ppm

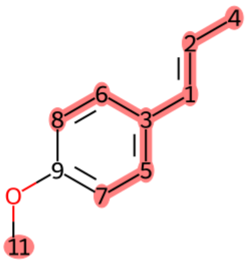

Rank: 399 MW: 148.2
(E)-Anethole
Score: 0.9 (9/10 C)
Deviation : 4.13 ppm

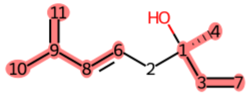

Rank: 400 MW: 152.23
Hotrienol
Score: 0.9 (9/10 C)
Deviation : 4.38 ppm

Figure S41. Dereplication analysis from MixONat, structure of dereplicated compounds: Rank **391-400**.

$

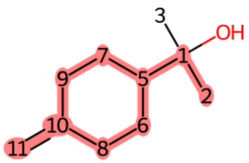

Rank: 401 MW: 154.25
delta-Terpineol
Score: 0.9 (9/10 C)
Deviation : 4.6 ppm

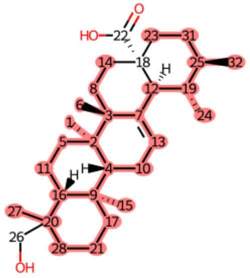

Rank: 402 MW: 456.7
CAS-1227736-23-3
Score: 0.9 (27/30 C)
Deviation : 4.92 ppm

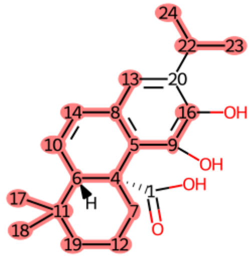

Rank: 403 MW: 330.42
CAS-951125-99-8
Score: 0.9 (18/20 C)
Deviation : 5.16 ppm

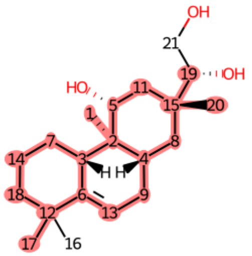

Rank: 404 MW: 322.48
Lagascatriol
Score: 0.9 (18/20 C)
Deviation : 5.4 ppm

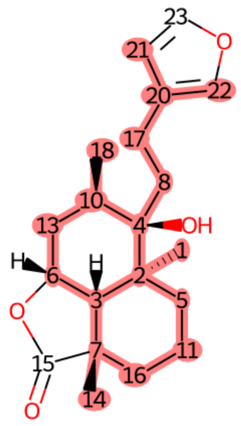

Rank: 405 MW: 332.43
(+)-Marrubiin
Score: 0.9 (18/20 C)
Deviation : 5.54 ppm

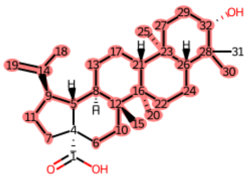

Rank: 406 MW: 456.7
(+)-Betulinic acid
Score: 0.9 (27/30 C)
Deviation : 5.84 ppm

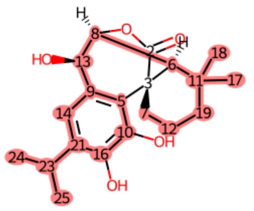

Rank: 407 MW: 346.42
Epirosmanol
Score: 0.9 (18/20 C)
Deviation : 6.69 ppm

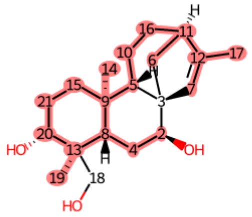

Rank: 408 MW: 320.47
Isofoliol
Score: 0.9 (18/20 C)
Deviation : 6.72 ppm

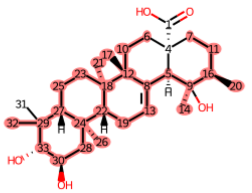

Rank: 409 MW: 488.7
Tormentic acid
Score: 0.9 (27/30 C)
Deviation : 6.76 ppm

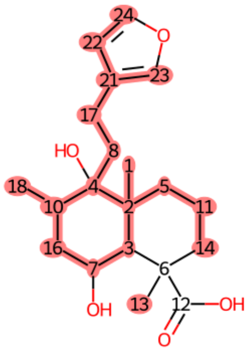

Rank: 410 MW: 350.45
CAS-101060-33-7
Score: 0.9 (18/20 C)
Deviation : 6.89 ppm

Figure S42. Dereplication analysis from MixONat, structure of dereplicated compounds: Rank **401-410**.

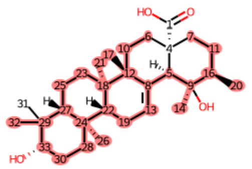

Rank: 411 MW: 472.7
Pomolic acid
Score: 0.9 (27/30 C)
Deviation : 7.43 ppm

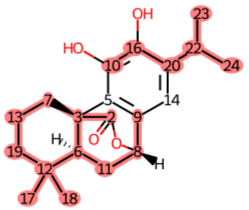

Rank: 412 MW: 330.42
Carnosol
Score: 0.9 (18/20 C)
Deviation : 8.21 ppm

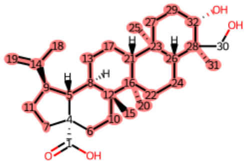

Rank: 413 MW: 472.7
Anemosapogenin
Score: 0.9 (27/30 C)
Deviation : 8.28 ppm

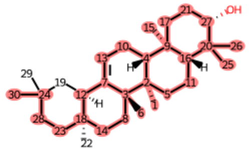

Rank: 414 MW: 426.72
(+)-beta-Amyrin
Score: 0.9 (27/30 C)
Deviation : 8.97 ppm

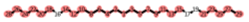

Rank: 415 MW: 408.79
Nonacosane
Score: 0.9 (26/29 C)
Deviation : 9.48 ppm

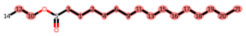

Rank: 416 MW: 298.5
Propyl palmitate
Score: 0.89 (17/19 C)
Deviation : 2.62 ppm

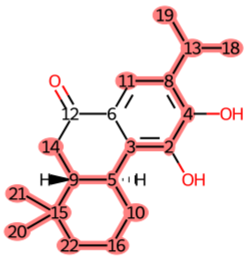

Rank: 417 MW: 302.41
CAS-951126-00-4
Score: 0.89 (17/19 C)
Deviation : 6.04 ppm

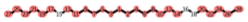

Rank: 418 MW: 394.76
Octacosane
Score: 0.89 (25/28 C)
Deviation : 8.2 ppm

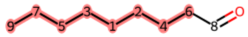

Rank: 419 MW: 142.24
Nonanal
Score: 0.89 (8/9 C)
Deviation : 0.38 ppm

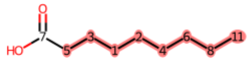

Rank: 420 MW: 158.24
Nonanoic acid
Score: 0.89 (8/9 C)
Deviation : 0.71 ppm

Figure S43. Dereplication analysis from MixONat, structure of dereplicated compounds: Rank **411-420**.

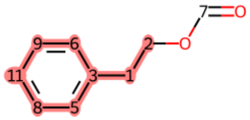

Rank: 421 MW: 150.17
beta-Phenethyl formate
Score: 0.89 (8/9 C)
Deviation : 1.16 ppm

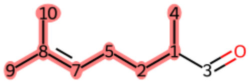

Rank: 422 MW: 140.22
Bergamal
Score: 0.89 (8/9 C)
Deviation : 1.29 ppm

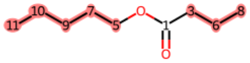

Rank: 423 MW: 158.24
Pentyl butanoate
Score: 0.89 (8/9 C)
Deviation : 1.37 ppm

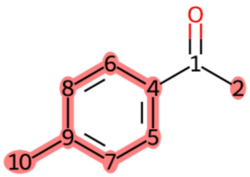

Rank: 424 MW: 134.18
Melilotal
Score: 0.89 (8/9 C)
Deviation : 1.38 ppm

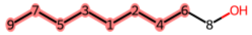

Rank: 425 MW: 144.25
Nonanol
Score: 0.89 (8/9 C)
Deviation : 1.41 ppm

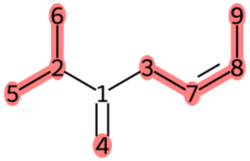

Rank: 426 MW: 124.22
(Z)-Salvene
Score: 0.89 (8/9 C)
Deviation : 1.97 ppm

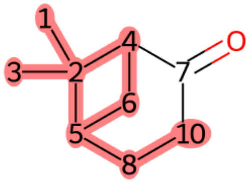

Rank: 427 MW: 138.21
beta-Pinone
Score: 0.89 (8/9 C)
Deviation : 2.04 ppm

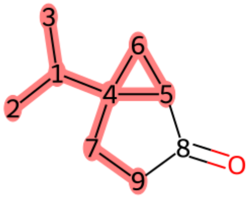

Rank: 428 MW: 138.21
Sabina ketone
Score: 0.89 (8/9 C)
Deviation : 2.15 ppm

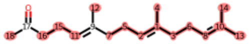

Rank: 429 MW: 262.43
Farnesylacetone A
Score: 0.89 (16/18 C)
Deviation : 2.52 ppm

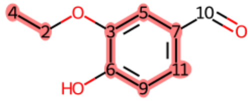

Rank: 430 MW: 166.17
Vanillal
Score: 0.89 (8/9 C)
Deviation : 3.29 ppm

Figure S44. Dereplication analysis from MixONat, structure of dereplicated compounds: Rank **421-430**.

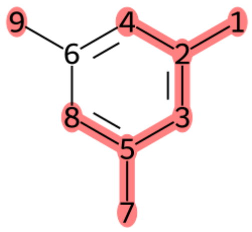

Rank: 431 MW: 120.19
Mesitylene
Score: 0.89 (8/9 C)
Deviation : 3.61 ppm

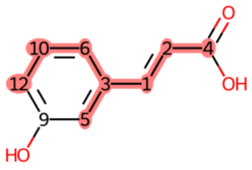

Rank: 432 MW: 164.16
m-Coumaric acid
Score: 0.89 (8/9 C)
Deviation : 4.1 ppm

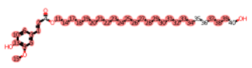

Rank: 433 MW: 574.87
CAS-1242279-57-7
Score: 0.89 (32/36 C)
Deviation : 8.28 ppm

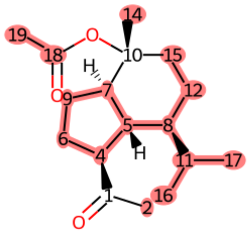

Rank: 434 MW: 280.4
Oplopanonyl acetate
Score: 0.88 (15/17 C)
Deviation : 4.24 ppm

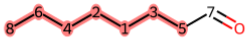

Rank: 435 MW: 128.21
Octanal
Score: 0.88 (7/8 C)
Deviation : 0.5 ppm

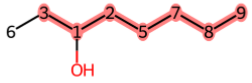

Rank: 436 MW: 130.23
3-Octanol
Score: 0.88 (7/8 C)
Deviation : 0.53 ppm

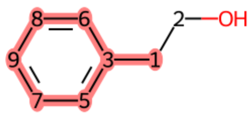

Rank: 437 MW: 122.16
Benzeneethanol
Score: 0.88 (7/8 C)
Deviation : 1.17 ppm

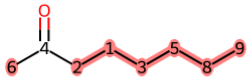

Rank: 438 MW: 128.21
2-Octanone
Score: 0.88 (7/8 C)
Deviation : 1.32 ppm

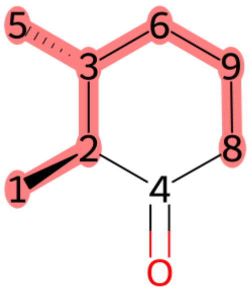

Rank: 439 MW: 126.2
CAS-1551-89-9
Score: 0.88 (7/8 C)
Deviation : 1.38 ppm

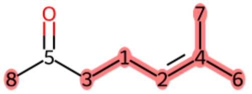

Rank: 440 MW: 126.2
Isoprenylacetone
Score: 0.88 (7/8 C)
Deviation : 1.39 ppm

Figure S45. Dereplication analysis from MixONat, structure of dereplicated compounds: Rank **431-440**.

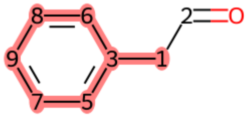

Rank: 441 MW: 120.15
Benzeneacetaldehyde
Score: 0.88 (7/8 C)
Deviation : 1.46 ppm

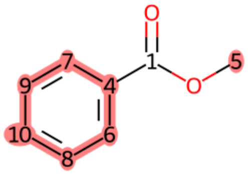

Rank: 442 MW: 136.15
Methyl benzoate
Score: 0.88 (7/8 C)
Deviation : 1.69 ppm

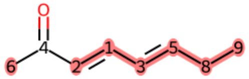

Rank: 443 MW: 124.18
3,5-Octadien-2-one
Score: 0.88 (7/8 C)
Deviation : 1.71 ppm

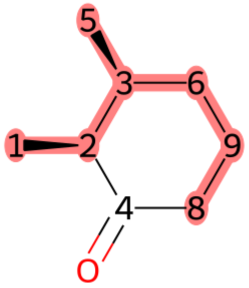

Rank: 444 MW: 126.2
CAS-1551-88-8
Score: 0.88 (7/8 C)
Deviation : 1.94 ppm

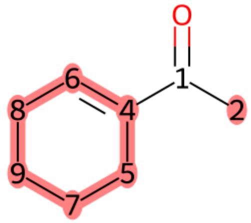

Rank: 445 MW: 124.18
1-Acetylcyclohexene
Score: 0.88 (7/8 C)
Deviation : 2.38 ppm

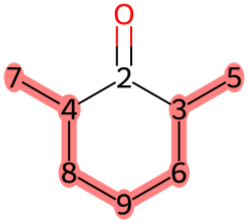

Rank: 446 MW: 126.2
CAS-2816-57-1
Score: 0.88 (7/8 C)
Deviation : 3.09 ppm

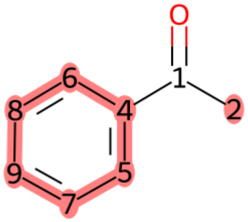

Rank: 447 MW: 120.15
Acetophenone
Score: 0.88 (7/8 C)
Deviation : 3.13 ppm

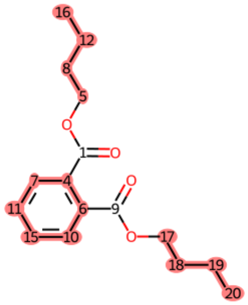

Rank: 448 MW: 278.34
Dibutyl phthalate
Score: 0.88 (14/16 C)
Deviation : 3.15 ppm

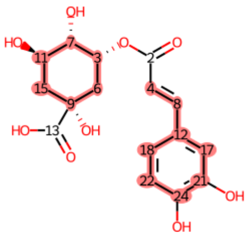

Rank: 449 MW: 354.31
CAS-906-33-2
Score: 0.88 (14/16 C)
Deviation : 7.61 ppm

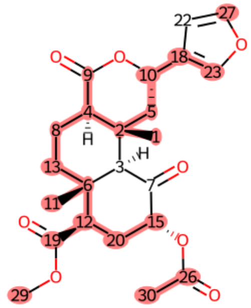

Rank: 450 MW: 432.46
2-epi-Salvinorin A
Score: 0.87 (20/23 C)
Deviation : 8.72 ppm

Figure S46. Dereplication analysis from MixONat, structure of dereplicated compounds: Rank **441-450**.

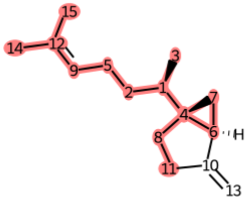

Rank: 451 MW: 204.35
(-)-Sesquisabinene
Score: 0.87 (13/15 C)
Deviation : 1.18 ppm

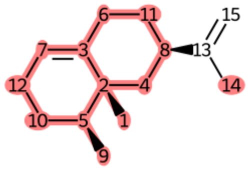

Rank: 452 MW: 204.35
Eremophilene
Score: 0.87 (13/15 C)
Deviation : 1.48 ppm

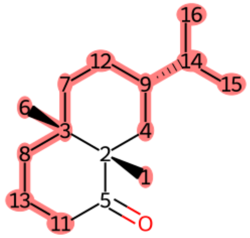

Rank: 453 MW: 222.37
Valeranone
Score: 0.87 (13/15 C)
Deviation : 1.72 ppm

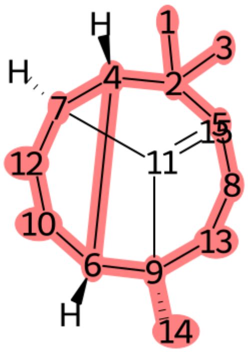

Rank: 454 MW: 204.35
(+)-Longifolene
Score: 0.87 (13/15 C)
Deviation : 1.81 ppm

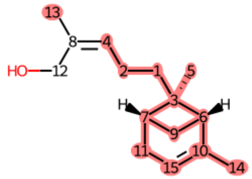

Rank: 455 MW: 220.35
CAS-88034-74-6
Score: 0.87 (13/15 C)
Deviation : 1.88 ppm

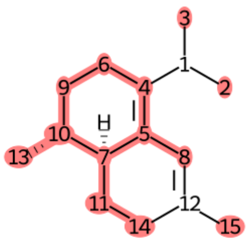

Rank: 456 MW: 204.35
(+)-Epizonarene
Score: 0.87 (13/15 C)
Deviation : 1.95 ppm

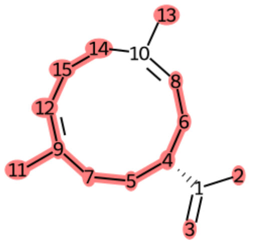

Rank: 457 MW: 204.35
(+)-Germacrene A
Score: 0.87 (13/15 C)
Deviation : 1.98 ppm

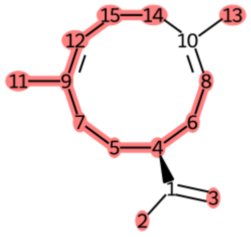

Rank: 458 MW: 204.35
(-)-Germacrene A
Score: 0.87 (13/15 C)
Deviation : 1.98 ppm

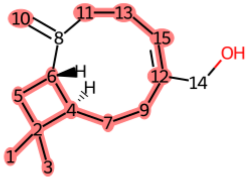

Rank: 459 MW: 220.35
CAS-79768-25-5
Score: 0.87 (13/15 C)
Deviation : 2.35 ppm

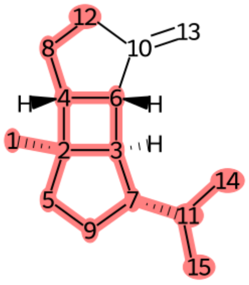

Rank: 460 MW: 204.35
(-)-beta-Bourbonene
Score: 0.87 (13/15 C)
Deviation : 2.5 ppm

Figure S47. Dereplication analysis from MixONat, structure of dereplicated compounds: Rank **451-460**.

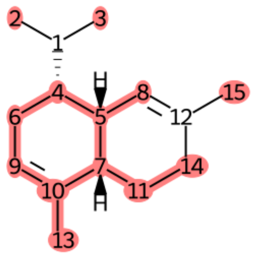

Rank: 461 MW: 204.35
(+)-alpha-Amorphene
Score: 0.87 (13/15 C)
Deviation : 2.52 ppm

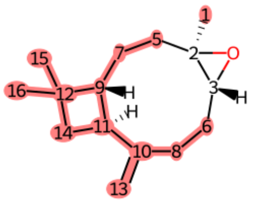

Rank: 462 MW: 220.35
CAS-1139-30-6
Score: 0.87 (13/15 C)
Deviation : 2.56 ppm

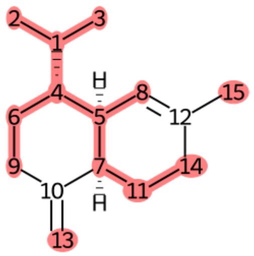

Rank: 463 MW: 204.35
gamma-Muurolene
Score: 0.87 (13/15 C)
Deviation : 2.57 ppm

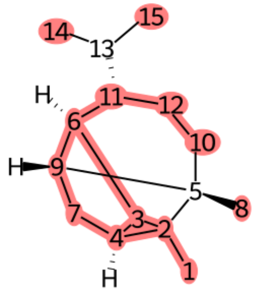

Rank: 464 MW: 204.35
(+)-Cyclosativene
Score: 0.87 (13/15 C)
Deviation : 2.62 ppm

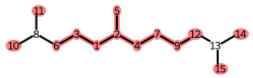

Rank: 465 MW: 212.41
CAS-31295-56-4
Score: 0.87 (13/15 C)
Deviation : 2.71 ppm

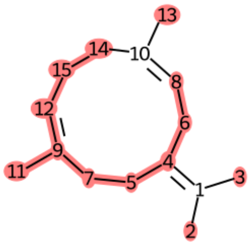

Rank: 466 MW: 204.35
Germacrene B
Score: 0.87 (13/15 C)
Deviation : 2.72 ppm

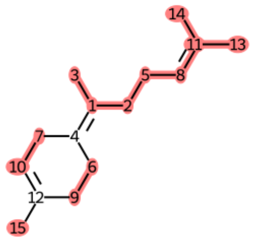

Rank: 467 MW: 204.35
(E)-gamma-Bisabolene
Score: 0.87 (13/15 C)
Deviation : 2.81 ppm

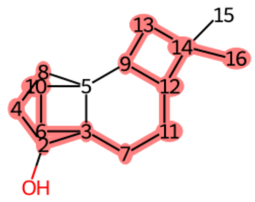

Rank: 468 MW: 220.35
CAS-783343-46-4
Score: 0.87 (13/15 C)
Deviation : 2.87 ppm

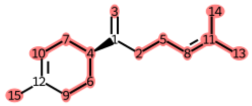

Rank: 469 MW: 204.35
(-)-beta-Bisabolene
Score: 0.87 (13/15 C)
Deviation : 2.97 ppm

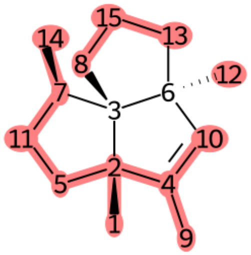

Rank: 470 MW: 204.35
(-)-alpha-Isocomene
Score: 0.87 (13/15 C)
Deviation : 2.98 ppm

Figure S48. Dereplication analysis from MixONat, structure of dereplicated compounds: Rank **461-470**.

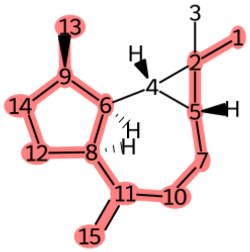

Rank: 471 MW: 204.35
(-)-Alloaromadendrene
Score: 0.87 (13/15 C)
Deviation : 2.99 ppm

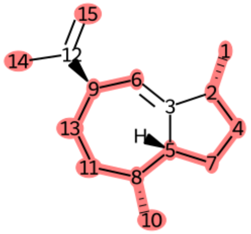

Rank: 472 MW: 204.35
(+)-gamma-Gurjunene
Score: 0.87 (13/15 C)
Deviation : 3.0 ppm

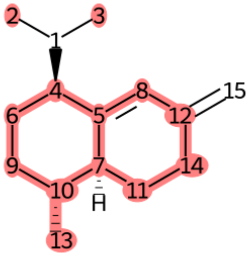

Rank: 473 MW: 204.35
CAS-54274-73-6
Score: 0.87 (13/15 C)
Deviation : 3.03 ppm

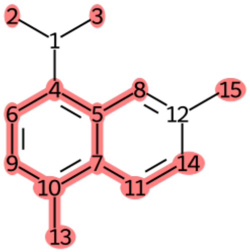

Rank: 474 MW: 198.3
Cadalene
Score: 0.87 (13/15 C)
Deviation : 3.05 ppm

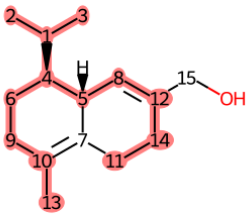

Rank: 475 MW: 220.35
CAS-153408-92-5
Score: 0.87 (13/15 C)
Deviation : 3.09 ppm

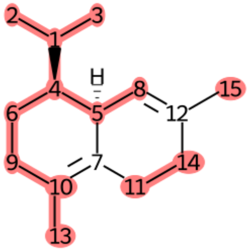

Rank: 476 MW: 204.35
(+)-delta-Amorphene
Score: 0.87 (13/15 C)
Deviation : 3.1 ppm

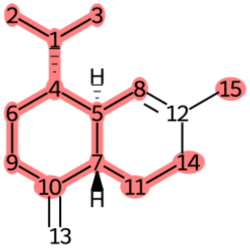

Rank: 477 MW: 204.35
gamma-Cadinene
Score: 0.87 (13/15 C)
Deviation : 3.23 ppm

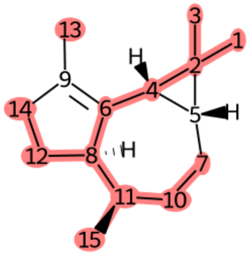

Rank: 478 MW: 204.35
(-)-alpha-Gurjunene
Score: 0.87 (13/15 C)
Deviation : 3.26 ppm

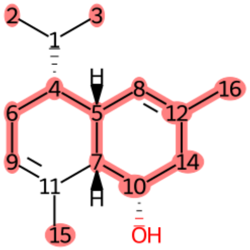

Rank: 479 MW: 220.35
Amorpha-4,9-dien-2-ol
Score: 0.87 (13/15 C)
Deviation : 3.31 ppm

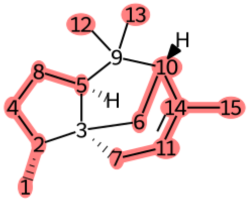

Rank: 480 MW: 204.35
(-)-alpha-Cedrene
Score: 0.87 (13/15 C)
Deviation : 3.33 ppm

Figure S49. Dereplication analysis from MixONat, structure of dereplicated compounds: Rank **471-480**.

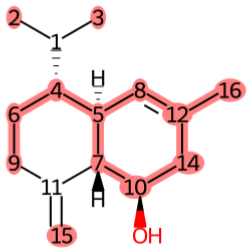

Rank: 481 MW: 220.35
Khusinol
Score: 0.87 (13/15 C)
Deviation : 3.38 ppm

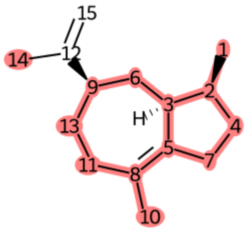

Rank: 482 MW: 204.35
delta-Guaiene
Score: 0.87 (13/15 C)
Deviation : 3.4 ppm

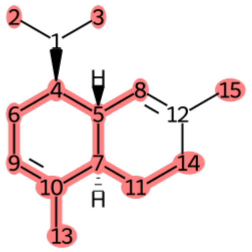

Rank: 483 MW: 204.35
(-)-a-Cadinene
Score: 0.87 (13/15 C)
Deviation : 3.46 ppm

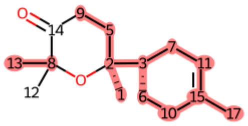

Rank: 484 MW: 236.35
CAS-22567-38-0
Score: 0.87 (13/15 C)
Deviation : 3.52 ppm

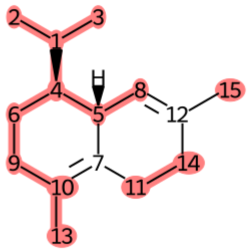

Rank: 485 MW: 204.35
(+)-delta-Cadinene
Score: 0.87 (13/15 C)
Deviation : 3.58 ppm

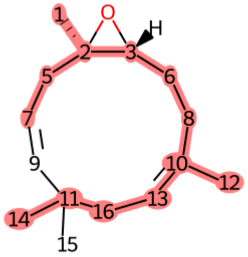

Rank: 486 MW: 220.35
Humulene epoxide II
Score: 0.87 (13/15 C)
Deviation : 3.62 ppm

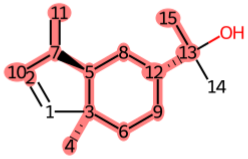

Rank: 487 MW: 222.37
beta-Elemol
Score: 0.87 (13/15 C)
Deviation : 3.63 ppm

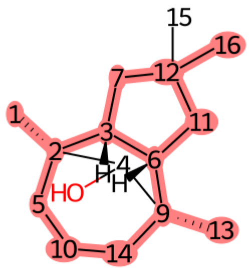

Rank: 488 MW: 222.37
CAS-4586-22-5
Score: 0.87 (13/15 C)
Deviation : 3.64 ppm

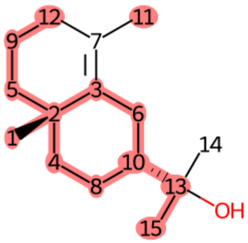

Rank: 489 MW: 222.37
CAS-15051-81-7
Score: 0.87 (13/15 C)
Deviation : 3.7 ppm

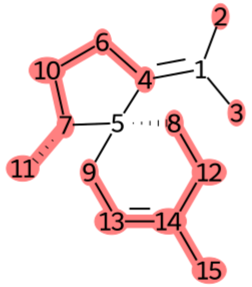

Rank: 490 MW: 204.35
(-)-alpha-Alaskene
Score: 0.87 (13/15 C)
Deviation : 3.73 ppm

Figure S50. Dereplication analysis from MixONat, structure of dereplicated compounds: Rank **481-490**.

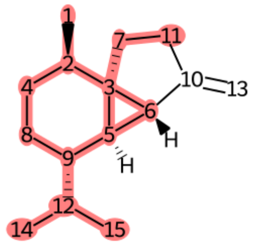

Rank: 491 MW: 204.35
(-)-beta-Cubebene
Score: 0.87 (13/15 C)
Deviation : 3.75 ppm

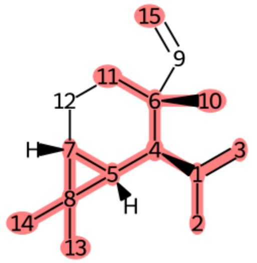

Rank: 492 MW: 204.35
(-)-Bicycloelemene
Score: 0.87 (13/15 C)
Deviation : 3.79 ppm

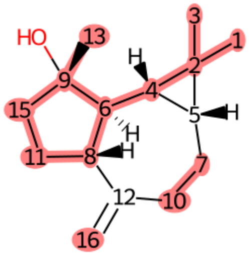

Rank: 493 MW: 220.35
(+)-Spathulenol
Score: 0.87 (13/15 C)
Deviation : 3.85 ppm

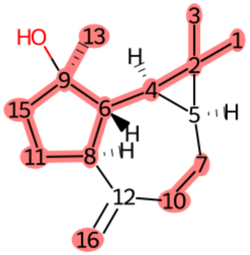

Rank: 494 MW: 220.35
(-)-Spathulenol
Score: 0.87 (13/15 C)
Deviation : 3.85 ppm

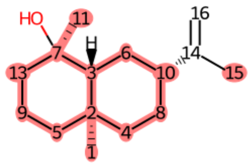

Rank: 495 MW: 222.37
Kongol
Score: 0.87 (13/15 C)
Deviation : 3.86 ppm

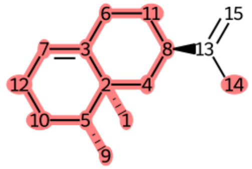

Rank: 496 MW: 204.35
(+)-Valencene
Score: 0.87 (13/15 C)
Deviation : 3.91 ppm

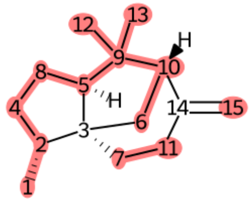

Rank: 497 MW: 204.35
(+)-beta-Cedrene
Score: 0.87 (13/15 C)
Deviation : 3.93 ppm

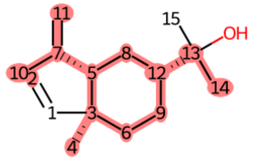

Rank: 498 MW: 222.37
(-)-Elemol
Score: 0.87 (13/15 C)
Deviation : 3.94 ppm

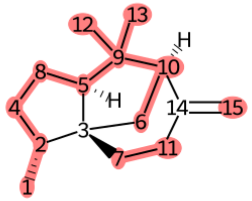

Rank: 499 MW: 204.35
(-)-beta-Funebrene
Score: 0.87 (13/15 C)
Deviation : 3.96 ppm

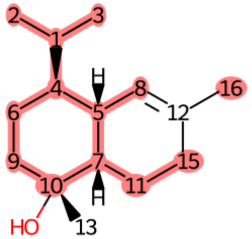

Rank: 500 MW: 222.37
(-)-Torreyol
Score: 0.87 (13/15 C)
Deviation : 4.01 ppm

Figure S51. Dereplication analysis from MixONat, structure of dereplicated compounds: Rank **491-500**.

Compound Data base 2

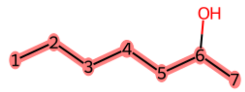

Rank: 1 MW: 116.2
LTS0147404
Score: 1.0 (7/7 C)
Deviation : 0.6 ppm

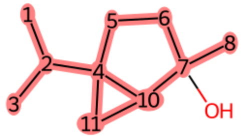

Rank: 2 MW: 154.25
LTS0236165
Score: 1.0 (10/10 C)
Deviation : 1.03 ppm

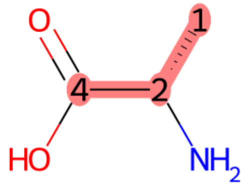

Rank: 3 MW: 89.09
LTS0042208
Score: 1.0 (3/3 C)
Deviation : 1.13 ppm

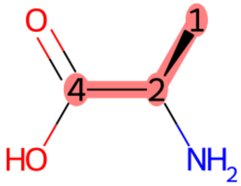

Rank: 4 MW: 89.09
LTS0272178
Score: 1.0 (3/3 C)
Deviation : 1.13 ppm

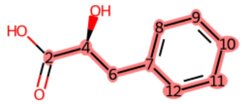

Rank: 5 MW: 166.17
LTS0008905
Score: 1.0 (9/9 C)
Deviation : 1.23 ppm

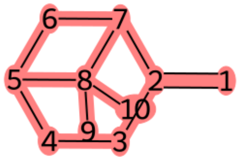

Rank: 6 MW: 136.23
LTS0117550
Score: 1.0 (10/10 C)
Deviation : 1.24 ppm

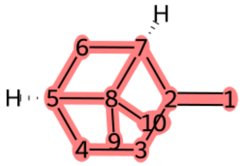

Rank: 7 MW: 136.23
LTS0108757
Score: 1.0 (10/10 C)
Deviation : 1.24 ppm

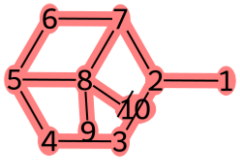

Rank: 8 MW: 136.23
LTS0132416
Score: 1.0 (10/10 C)
Deviation : 1.39 ppm

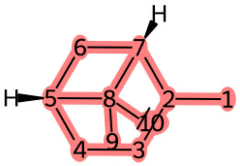

Rank: 9 MW: 136.23
LTS0211102
Score: 1.0 (10/10 C)
Deviation : 1.39 ppm

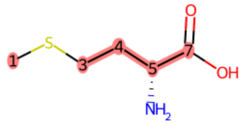

Rank: 10 MW: 149.21
LTS0108782
Score: 1.0 (5/5 C)
Deviation : 1.43 ppm

Figure S52: Dereplication analysis from MixONat, structure of dereplicated compounds: Rank **1-10**.

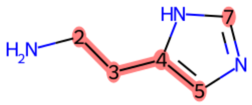

Rank: 11 MW: 111.15
LTS0016235
Score: 1.0 (5/5 C)
Deviation : 1.43 ppm

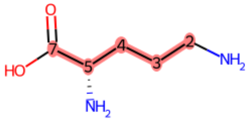

Rank: 12 MW: 132.16
LTS0093444
Score: 1.0 (5/5 C)
Deviation : 1.46 ppm

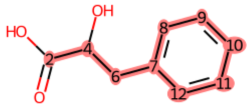

Rank: 13 MW: 166.17
LTS0175088
Score: 1.0 (9/9 C)
Deviation : 1.48 ppm

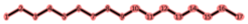

Rank: 14 MW: 240.47
LTS0038303
Score: 1.0 (17/17 C)
Deviation : 1.53 ppm

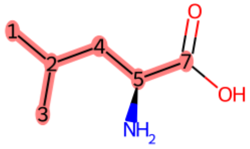

Rank: 15 MW: 131.17
LTS0113423
Score: 1.0 (6/6 C)
Deviation : 1.53 ppm

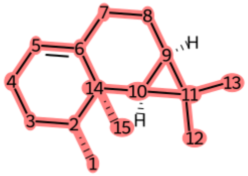

Rank: 16 MW: 204.35
LTS0234436
Score: 1.0 (15/15 C)
Deviation : 1.86 ppm

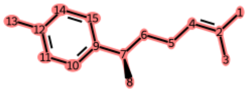

Rank: 17 MW: 202.34
LTS0216936
Score: 1.0 (15/15 C)
Deviation : 2.09 ppm

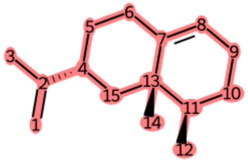

Rank: 18 MW: 204.35
LTS0110395
Score: 1.0 (15/15 C)
Deviation : 2.09 ppm

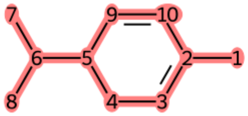

Rank: 19 MW: 136.23
LTS0157173
Score: 1.0 (10/10 C)
Deviation : 2.14 ppm

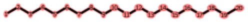

Rank: 20 MW: 282.55
LTS0268882
Score: 1.0 (20/20 C)
Deviation : 2.17 ppm

Figure S53: Dereplication analysis from MixONat, structure of dereplicated compounds: Rank **11-20**.

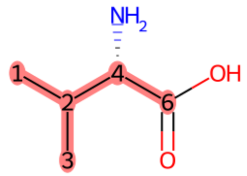

Rank: 21 MW: 117.15
LTS0231703
Score: 1.0 (5/5 C)
Deviation : 2.24 ppm

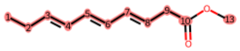

Rank: 22 MW: 180.24
LTS0118968
Score: 1.0 (11/11 C)
Deviation : 2.25 ppm

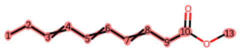

Rank: 23 MW: 180.24
LTS0168847
Score: 1.0 (11/11 C)
Deviation : 2.25 ppm

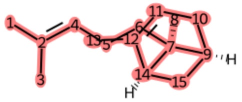

Rank: 24 MW: 204.35
LTS0153835
Score: 1.0 (15/15 C)
Deviation : 2.37 ppm

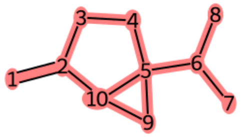

Rank: 25 MW: 136.23
LTS0224133
Score: 1.0 (10/10 C)
Deviation : 2.48 ppm

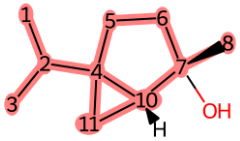

Rank: 26 MW: 154.25
LTS0265986
Score: 1.0 (10/10 C)
Deviation : 2.55 ppm

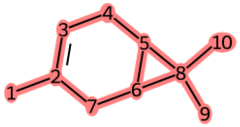

Rank: 27 MW: 136.23
LTS0106881
Score: 1.0 (10/10 C)
Deviation : 2.57 ppm

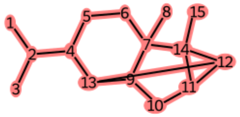

Rank: 28 MW: 204.35
LTS0040030
Score: 1.0 (15/15 C)
Deviation : 2.64 ppm

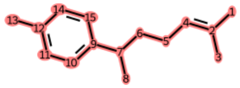

Rank: 29 MW: 202.34
LTS0190074
Score: 1.0 (15/15 C)
Deviation : 2.7 ppm

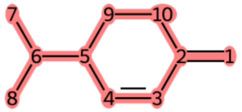

Rank: 30 MW: 136.23
LTS0124668
Score: 1.0 (10/10 C)
Deviation : 2.78 ppm

Figure S54: Dereplication analysis from MixONat, structure of dereplicated compounds: Rank **21-30**.

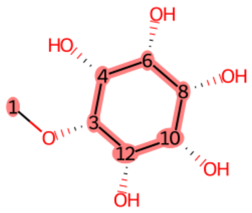

Rank: 31 MW: 194.18
LTS0220361
Score: 1.0 (7/7 C)
Deviation : 2.81 ppm

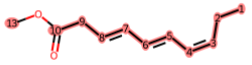

Rank: 32 MW: 180.24
LTS0185398
Score: 1.0 (11/11 C)
Deviation : 2.83 ppm

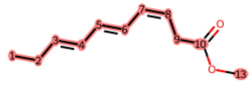

Rank: 33 MW: 180.24
LTS0188271
Score: 1.0 (11/11 C)
Deviation : 2.91 ppm

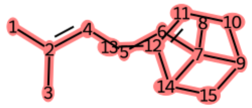

Rank: 34 MW: 204.35
LTS0226115
Score: 1.0 (15/15 C)
Deviation : 2.97 ppm

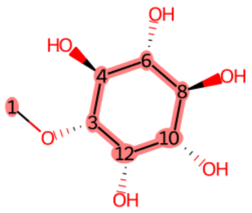

Rank: 35 MW: 194.18
LTS0249934
Score: 1.0 (7/7 C)
Deviation : 3.02 ppm

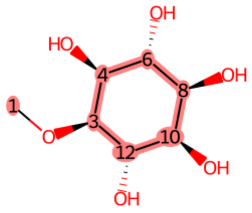

Rank: 36 MW: 194.18
LTS0051765
Score: 1.0 (7/7 C)
Deviation : 3.02 ppm

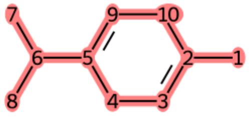

Rank: 37 MW: 136.23
LTS0232891
Score: 1.0 (10/10 C)
Deviation : 3.1 ppm

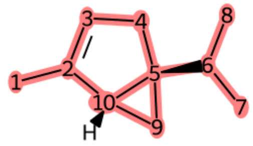

Rank: 38 MW: 136.23
LTS0092688
Score: 1.0 (10/10 C)
Deviation : 3.2 ppm

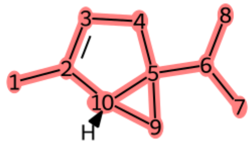

Rank: 39 MW: 136.23
LTS0176954
Score: 1.0 (10/10 C)
Deviation : 3.2 ppm

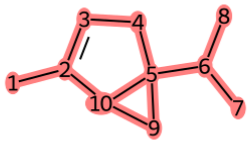

Rank: 40 MW: 136.23
LTS0185078
Score: 1.0 (10/10 C)
Deviation : 3.2 ppm

Figure S55: Dereplication analysis from MixONat, structure of dereplicated compounds: Rank **31-40**.

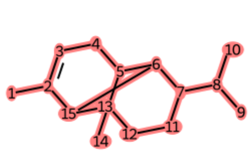

Rank: 41 MW: 204.35
LTS0207598
Score: 1.0 (15/15 C)
Deviation : 3.28 ppm

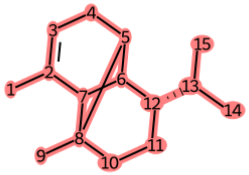

Rank: 42 MW: 204.35
LTS0190031
Score: 1.0 (15/15 C)
Deviation : 3.28 ppm

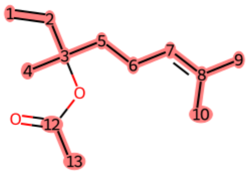

Rank: 43 MW: 196.29
LTS0167325
Score: 1.0 (12/12 C)
Deviation : 3.31 ppm

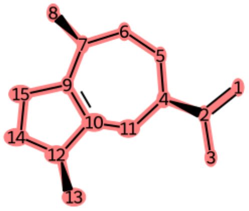

Rank: 44 MW: 204.35
LTS0039431
Score: 1.0 (15/15 C)
Deviation : 3.34 ppm

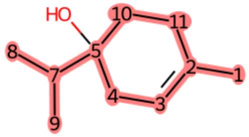

Rank: 45 MW: 154.25
LTS0253733
Score: 1.0 (10/10 C)
Deviation : 3.36 ppm

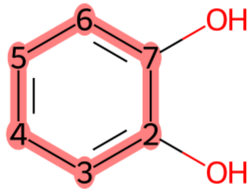

Rank: 46 MW: 110.11
LTS0178554
Score: 1.0 (6/6 C)
Deviation : 3.38 ppm

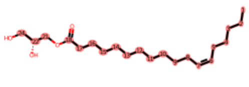

Rank: 47 MW: 356.54
LTS0016216
Score: 1.0 (21/21 C)
Deviation : 3.42 ppm

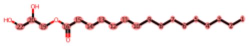

Rank: 48 MW: 330.5
LTS0073260
Score: 1.0 (19/19 C)
Deviation : 3.47 ppm

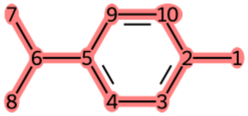

Rank: 49 MW: 134.22
LTS0181568
Score: 1.0 (10/10 C)
Deviation : 3.53 ppm

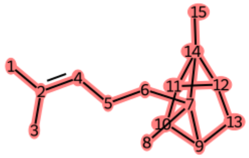

Rank: 50 MW: 204.35
LTS0197179
Score: 1.0 (15/15 C)
Deviation : 3.55 ppm

Figure S56: Dereplication analysis from MixONat, structure of dereplicated compounds: Rank **41-50**.

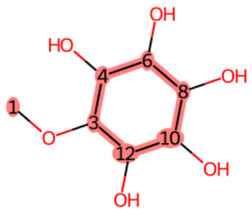

Rank: 51 MW: 194.18
LTS0194724
Score: 1.0 (7/7 C)
Deviation : 3.56 ppm

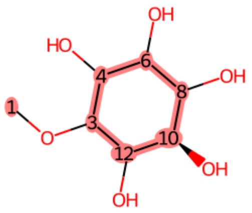

Rank: 52 MW: 194.18
LTS0170182
Score: 1.0 (7/7 C)
Deviation : 3.56 ppm

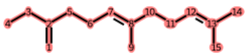

Rank: 53 MW: 206.37
LTS0154516
Score: 1.0 (15/15 C)
Deviation : 3.59 ppm

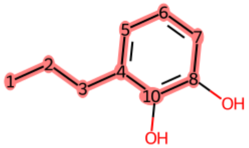

Rank: 54 MW: 152.19
LTS0104472
Score: 1.0 (9/9 C)
Deviation : 3.62 ppm

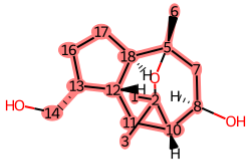

Rank: 55 MW: 254.37
LTS0131420
Score: 1.0 (15/15 C)
Deviation : 3.65 ppm

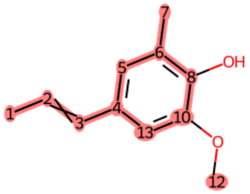

Rank: 56 MW: 178.23
LTS0066704
Score: 1.0 (11/11 C)
Deviation : 3.76 ppm

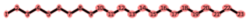

Rank: 57 MW: 324.63
LTS0089836
Score: 1.0 (23/23 C)
Deviation : 3.8 ppm

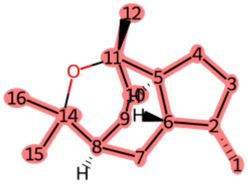

Rank: 58 MW: 222.37
LTS0258624
Score: 1.0 (15/15 C)
Deviation : 3.85 ppm

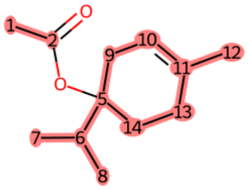

Rank: 59 MW: 196.29
LTS0008191
Score: 1.0 (12/12 C)
Deviation : 3.91 ppm

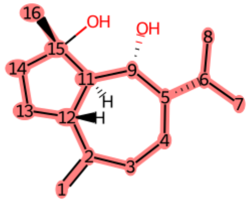

Rank: 60 MW: 238.37
LTS0194689
Score: 1.0 (15/15 C)
Deviation : 4.0 ppm

Figure S57: Dereplication analysis from MixONat, structure of dereplicated compounds: Rank **51-60**.

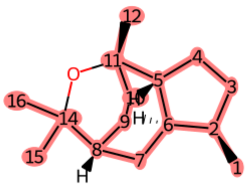

Rank: 61 MW: 222.37
LTS0123323
Score: 1.0 (15/15 C)
Deviation : 4.02 ppm

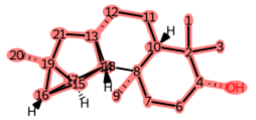

Rank: 62 MW: 288.47
LTS0221385
Score: 1.0 (20/20 C)
Deviation : 4.03 ppm

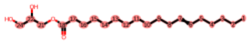

Rank: 63 MW: 356.54
LTS0213509
Score: 1.0 (21/21 C)
Deviation : 4.09 ppm

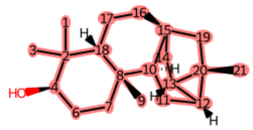

Rank: 64 MW: 288.47
LTS0180337
Score: 1.0 (20/20 C)
Deviation : 4.1 ppm

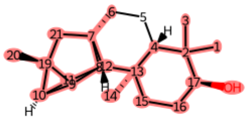

Rank: 65 MW: 288.47
LTS0098193
Score: 1.0 (20/20 C)
Deviation : 4.18 ppm

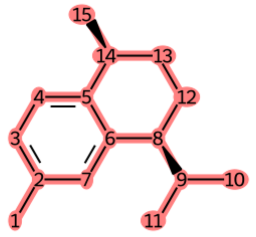

Rank: 66 MW: 202.34
LTS0139634
Score: 1.0 (15/15 C)
Deviation : 4.22 ppm

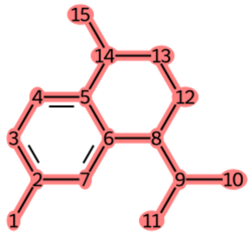

Rank: 67 MW: 202.34
LTS0228241
Score: 1.0 (15/15 C)
Deviation : 4.28 ppm

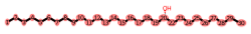

Rank: 68 MW: 424.79
LTS0221297
Score: 1.0 (29/29 C)
Deviation : 4.29 ppm

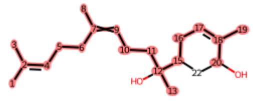

Rank: 69 MW: 306.48
LTS0060774
Score: 1.0 (19/19 C)
Deviation : 4.39 ppm

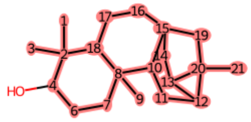

Rank: 70 MW: 288.47
LTS0042831
Score: 1.0 (20/20 C)
Deviation : 4.4 ppm

Figure S58: Dereplication analysis from MixONat, structure of dereplicated compounds: Rank **61-70**.

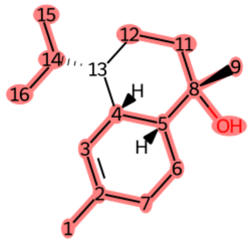

Rank: 71 MW: 222.37
LTS0234538
Score: 1.0 (15/15 C)
Deviation : 4.53 ppm

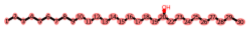

Rank: 72 MW: 424.79
LTS0157578
Score: 1.0 (29/29 C)
Deviation : 4.53 ppm

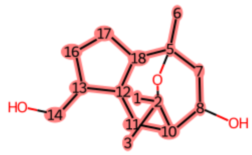

Rank: 73 MW: 254.37
LTS0011561
Score: 1.0 (15/15 C)
Deviation : 4.6 ppm

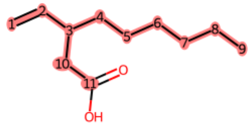

Rank: 74 MW: 184.28
LTS0115291
Score: 1.0 (11/11 C)
Deviation : 4.73 ppm

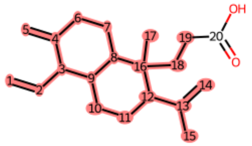

Rank: 75 MW: 302.45
LTS0007432
Score: 1.0 (19/19 C)
Deviation : 4.74 ppm

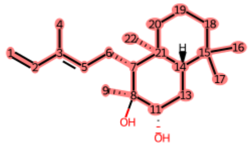

Rank: 76 MW: 306.48
LTS0148230
Score: 1.0 (20/20 C)
Deviation : 4.76 ppm

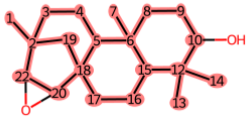

Rank: 77 MW: 304.47
LTS0038951
Score: 1.0 (20/20 C)
Deviation : 4.78 ppm

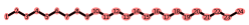

Rank: 78 MW: 338.65
LTS0090497
Score: 1.0 (24/24 C)
Deviation : 4.79 ppm

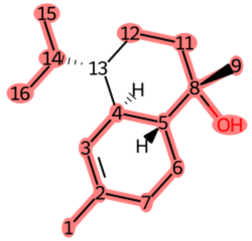

Rank: 79 MW: 222.37
LTS0178794
Score: 1.0 (15/15 C)
Deviation : 4.81 ppm

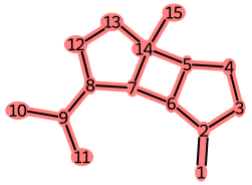

Rank: 80 MW: 204.35
LTS0167513
Score: 1.0 (15/15 C)
Deviation : 4.86 ppm

Figure S59: Dereplication analysis from MixONat, structure of dereplicated compounds: Rank **71-80**.

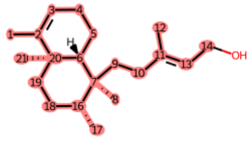

Rank: 81 MW: 290.48
LTS0219725
Score: 1.0 (20/20 C)
Deviation : 4.97 ppm

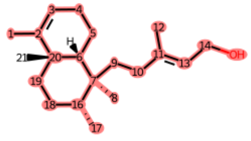

Rank: 82 MW: 290.48
LTS0132915
Score: 1.0 (20/20 C)
Deviation : 4.97 ppm

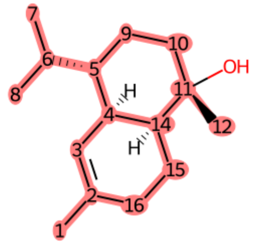

Rank: 83 MW: 222.37
LTS0013807
Score: 1.0 (15/15 C)
Deviation : 5.03 ppm

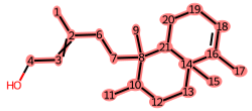

Rank: 84 MW: 290.48
LTS0033525
Score: 1.0 (20/20 C)
Deviation : 5.03 ppm

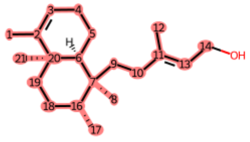

Rank: 85 MW: 290.48
LTS0211348
Score: 1.0 (20/20 C)
Deviation : 5.03 ppm

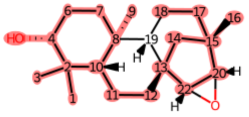

Rank: 86 MW: 304.47
LTS0158008
Score: 1.0 (20/20 C)
Deviation : 5.04 ppm

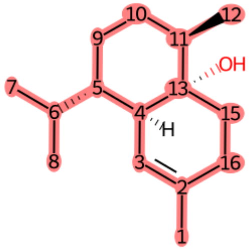

Rank: 87 MW: 222.37
LTS0077197
Score: 1.0 (15/15 C)
Deviation : 5.08 ppm

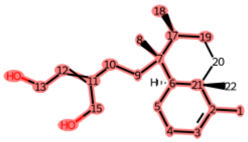

Rank: 88 MW: 306.48
LTS0129943
Score: 1.0 (20/20 C)
Deviation : 5.09 ppm

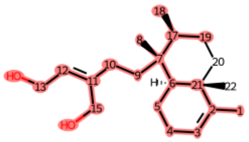

Rank: 89 MW: 306.48
LTS0125737
Score: 1.0 (20/20 C)
Deviation : 5.09 ppm

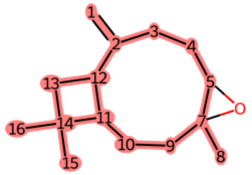

Rank: 90 MW: 220.35
LTS0159789
Score: 1.0 (15/15 C)
Deviation : 5.14 ppm

Figure S60: Dereplication analysis from MixONat, structure of dereplicated compounds: Rank **81-90**.

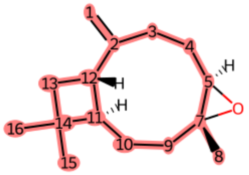

Rank: 91 MW: 220.35
LTS0213960
Score: 1.0 (15/15 C)
Deviation : 5.19 ppm

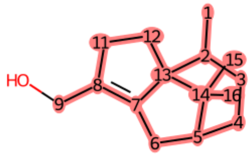

Rank: 92 MW: 220.35
LTS0094127
Score: 1.0 (15/15 C)
Deviation : 5.23 ppm

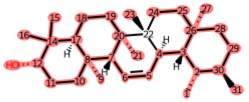

Rank: 93 MW: 426.72
LTS0017325
Score: 1.0 (30/30 C)
Deviation : 5.25 ppm

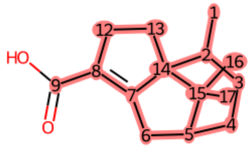

Rank: 94 MW: 234.33
LTS0166341
Score: 1.0 (15/15 C)
Deviation : 5.28 ppm

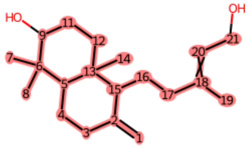

Rank: 95 MW: 306.48
LTS0203158
Score: 1.0 (20/20 C)
Deviation : 5.28 ppm

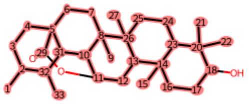

Rank: 96 MW: 456.7
LTS0132809
Score: 1.0 (30/30 C)
Deviation : 5.29 ppm

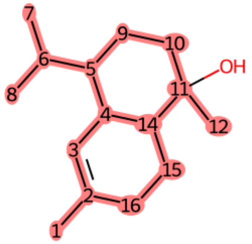

Rank: 97 MW: 222.37
LTS0272380
Score: 1.0 (15/15 C)
Deviation : 5.31 ppm

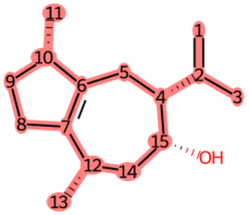

Rank: 98 MW: 220.35
LTS0266145
Score: 1.0 (15/15 C)
Deviation : 5.37 ppm

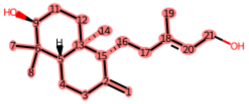

Rank: 99 MW: 306.48
LTS0246088
Score: 1.0 (20/20 C)
Deviation : 5.42 ppm

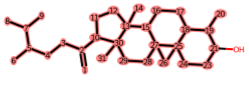

Rank: 100 MW: 426.72
LTS0146482
Score: 1.0 (30/30 C)
Deviation : 5.42 ppm

Figure S61: Dereplication analysis from MixONat, structure of dereplicated compounds: Rank **91-100**.

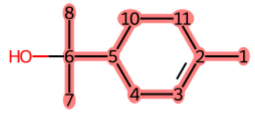

Rank: 101 MW: 154.25
LTS0136148
Score: 1.0 (10/10 C)
Deviation : 5.43 ppm

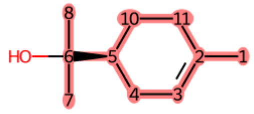

Rank: 102 MW: 154.25
LTS0258249
Score: 1.0 (10/10 C)
Deviation : 5.43 ppm

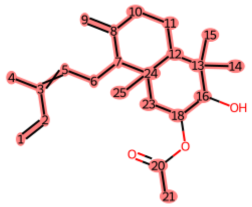

Rank: 103 MW: 346.5
LTS0083446
Score: 1.0 (22/22 C)
Deviation : 5.47 ppm

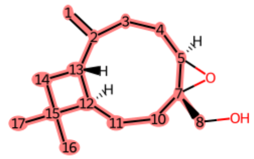

Rank: 104 MW: 236.35
LTS0081594
Score: 1.0 (15/15 C)
Deviation : 5.49 ppm

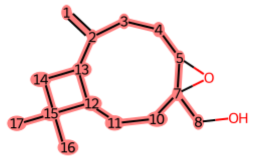

Rank: 105 MW: 236.35
LTS0135854
Score: 1.0 (15/15 C)
Deviation : 5.53 ppm

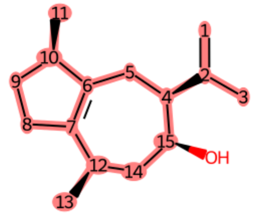

Rank: 106 MW: 220.35
LTS0196849
Score: 1.0 (15/15 C)
Deviation : 5.54 ppm

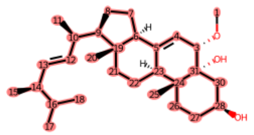

Rank: 107 MW: 444.69
LTS0265426
Score: 1.0 (29/29 C)
Deviation : 5.57 ppm

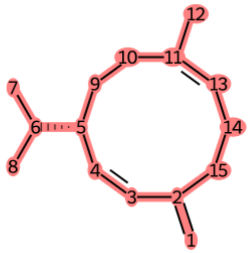

Rank: 108 MW: 204.35
LTS0065195
Score: 1.0 (15/15 C)
Deviation : 5.59 ppm

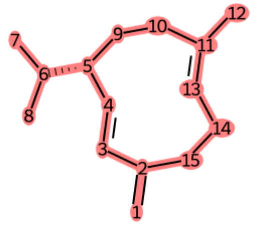

Rank: 109 MW: 204.35
LTS0059194
Score: 1.0 (15/15 C)
Deviation : 5.6 ppm

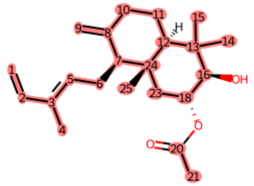

Rank: 110 MW: 346.5
LTS0167362
Score: 1.0 (22/22 C)
Deviation : 5.62 ppm

Figure S62: Dereplication analysis from MixONat, structure of dereplicated compounds: Rank **101-110**.

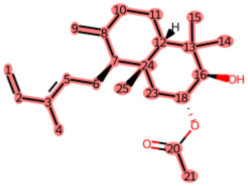

Rank: 111 MW: 346.5
LTS0066064
Score: 1.0 (22/22 C)
Deviation : 5.62 ppm

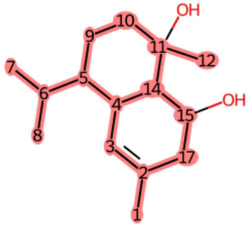

Rank: 112 MW: 238.37
LTS0171271
Score: 1.0 (15/15 C)
Deviation : 5.67 ppm

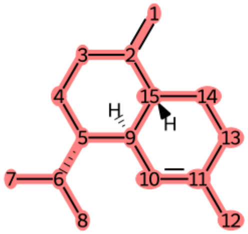

Rank: 113 MW: 204.35
LTS0103949
Score: 1.0 (15/15 C)
Deviation : 5.69 ppm

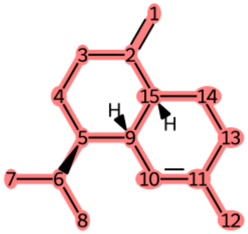

Rank: 114 MW: 204.35
LTS0211054
Score: 1.0 (15/15 C)
Deviation : 5.69 ppm

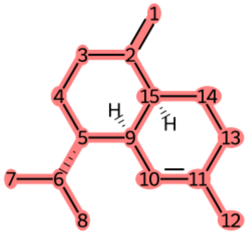

Rank: 115 MW: 204.35
LTS0052920
Score: 1.0 (15/15 C)
Deviation : 5.69 ppm

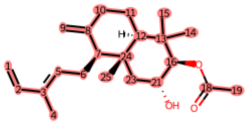

Rank: 116 MW: 346.5
LTS0216645
Score: 1.0 (22/22 C)
Deviation : 5.74 ppm

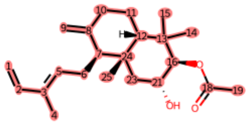

Rank: 117 MW: 346.5
LTS0015388
Score: 1.0 (22/22 C)
Deviation : 5.74 ppm

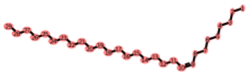

Rank: 118 MW: 406.77
LTS0260268
Score: 1.0 (29/29 C)
Deviation : 5.78 ppm

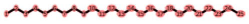

Rank: 119 MW: 352.68
LTS0080764
Score: 1.0 (25/25 C)
Deviation : 5.81 ppm

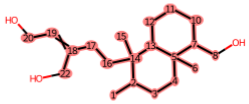

Rank: 120 MW: 324.5
LTS0255144
Score: 1.0 (20/20 C)
Deviation : 5.84 ppm

Figure S63: Dereplication analysis from MixONat, structure of dereplicated compounds: Rank **111-120**.

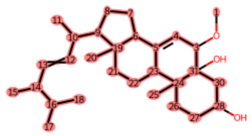

Rank: 121 MW: 444.69
LTS0096690
Score: 1.0 (29/29 C)
Deviation : 5.85 ppm

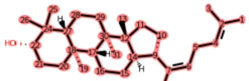

Rank: 122 MW: 426.72
LTS0059240
Score: 1.0 (30/30 C)
Deviation : 5.95 ppm

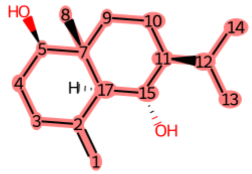

Rank: 123 MW: 238.37
LTS0071183
Score: 1.0 (15/15 C)
Deviation : 5.95 ppm

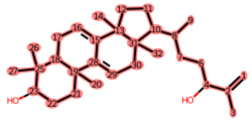

Rank: 124 MW: 440.7
LTS0118125
Score: 1.0 (30/30 C)
Deviation : 5.97 ppm

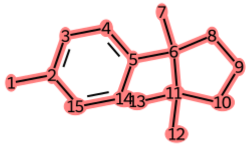

Rank: 125 MW: 202.34
LTS0261288
Score: 1.0 (15/15 C)
Deviation : 6.01 ppm

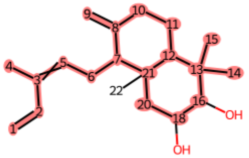

Rank: 126 MW: 304.47
LTS0236384
Score: 1.0 (19/19 C)
Deviation : 6.01 ppm

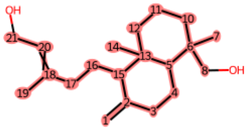

Rank: 127 MW: 306.48
LTS0133793
Score: 1.0 (20/20 C)
Deviation : 6.03 ppm

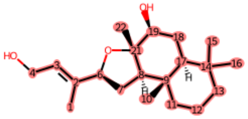

Rank: 128 MW: 322.48
LTS0223075
Score: 1.0 (20/20 C)
Deviation : 6.1 ppm

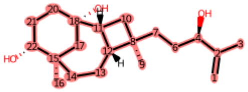

Rank: 129 MW: 322.48
LTS0179756
Score: 1.0 (20/20 C)
Deviation : 6.13 ppm

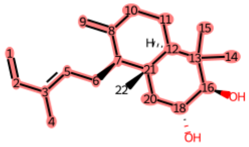

Rank: 130 MW: 304.47
LTS0266564
Score: 1.0 (19/19 C)
Deviation : 6.16 ppm

Figure S64: Dereplication analysis from MixONat, structure of dereplicated compounds: Rank **121-130**.

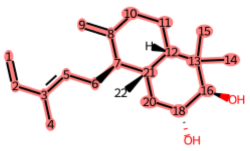

Rank: 131 MW: 304.47
LTS0081000
Score: 1.0 (19/19 C)
Deviation : 6.16 ppm

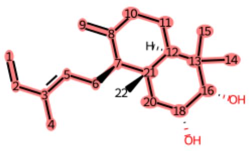

Rank: 132 MW: 304.47
LTS0275828
Score: 1.0 (19/19 C)
Deviation : 6.16 ppm

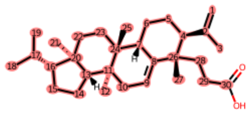

Rank: 133 MW: 440.7
LTS0131565
Score: 1.0 (30/30 C)
Deviation : 6.21 ppm

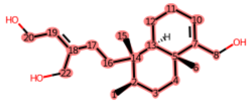

Rank: 134 MW: 322.48
LTS0159229
Score: 1.0 (20/20 C)
Deviation : 6.22 ppm

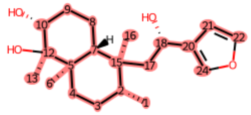

Rank: 135 MW: 336.47
LTS0029754
Score: 1.0 (20/20 C)
Deviation : 6.25 ppm

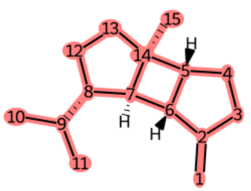

Rank: 136 MW: 204.35
LTS0074484
Score: 1.0 (15/15 C)
Deviation : 6.29 ppm

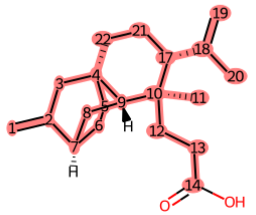

Rank: 137 MW: 302.45
LTS0236788
Score: 1.0 (20/20 C)
Deviation : 6.36 ppm

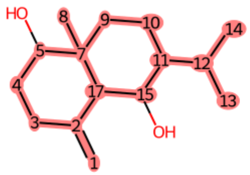

Rank: 138 MW: 238.37
LTS0190508
Score: 1.0 (15/15 C)
Deviation : 6.39 ppm

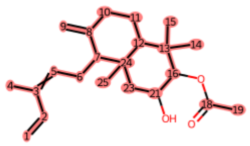

Rank: 139 MW: 346.5
LTS0138991
Score: 1.0 (22/22 C)
Deviation : 6.44 ppm

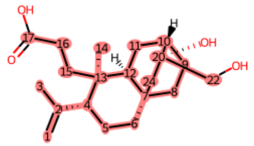

Rank: 140 MW: 336.47
LTS0227153
Score: 1.0 (20/20 C)
Deviation : 6.49 ppm

Figure S65: Dereplication analysis from MixONat, structure of dereplicated compounds: Rank **131-140**.

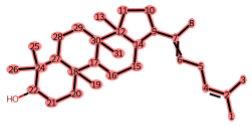

Rank: 141 MW: 426.72
LTS0172678
Score: 1.0 (30/30 C)
Deviation : 6.52 ppm

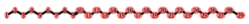

Rank: 142 MW: 406.77
LTS0146665
Score: 1.0 (29/29 C)
Deviation : 6.53 ppm

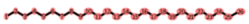

Rank: 143 MW: 366.71
LTS0079361
Score: 1.0 (26/26 C)
Deviation : 6.57 ppm

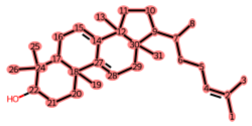

Rank: 144 MW: 424.7
LTS0192561
Score: 1.0 (30/30 C)
Deviation : 6.72 ppm

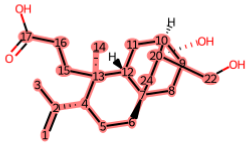

Rank: 145 MW: 336.47
LTS0072343
Score: 1.0 (20/20 C)
Deviation : 6.81 ppm

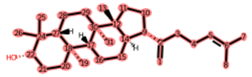

Rank: 146 MW: 426.72
LTS0080913
Score: 1.0 (30/30 C)
Deviation : 6.84 ppm

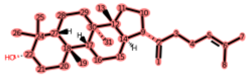

Rank: 147 MW: 426.72
LTS0070633
Score: 1.0 (30/30 C)
Deviation : 6.84 ppm

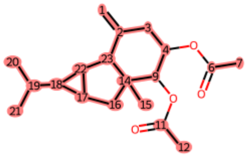

Rank: 148 MW: 320.42
LTS0174474
Score: 1.0 (19/19 C)
Deviation : 6.9 ppm

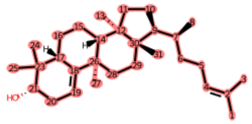

Rank: 149 MW: 426.72
LTS0207566
Score: 1.0 (30/30 C)
Deviation : 6.92 ppm

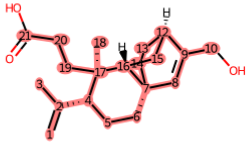

Rank: 150 MW: 318.45
LTS0156592
Score: 1.0 (20/20 C)
Deviation : 6.99 ppm

Figure S66: Dereplication analysis from MixONat, structure of dereplicated compounds: Rank **141-150**.

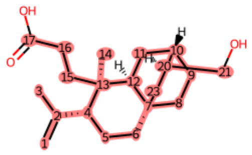

Rank: 151 MW: 320.47
LTS0075926
Score: 1.0 (20/20 C)
Deviation : 7.07 ppm

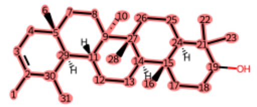

Rank: 152 MW: 426.72
LTS0269929
Score: 1.0 (30/30 C)
Deviation : 7.17 ppm

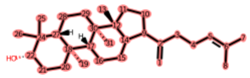

Rank: 153 MW: 426.72
LTS0215354
Score: 1.0 (30/30 C)
Deviation : 7.18 ppm

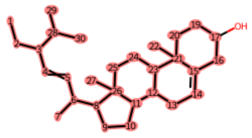

Rank: 154 MW: 412.69
LTS0024262
Score: 1.0 (29/29 C)
Deviation : 7.35 ppm

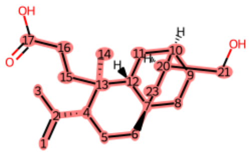

Rank: 155 MW: 320.47
LTS0051016
Score: 1.0 (20/20 C)
Deviation : 7.37 ppm

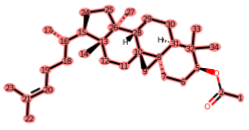

Rank: 156 MW: 468.76
LTS0218833
Score: 1.0 (32/32 C)
Deviation : 7.38 ppm

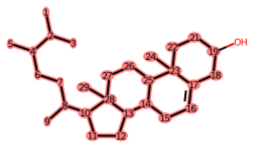

Rank: 157 MW: 400.68
LTS0046755
Score: 1.0 (28/28 C)
Deviation : 7.4 ppm

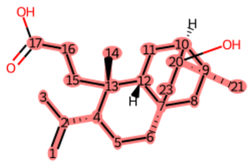

Rank: 158 MW: 320.47
LTS0255093
Score: 1.0 (20/20 C)
Deviation : 7.43 ppm

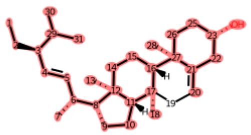

Rank: 159 MW: 426.72
LTS0254062
Score: 1.0 (30/30 C)
Deviation : 7.49 ppm

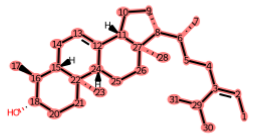

Rank: 160 MW: 426.72
LTS0155418
Score: 1.0 (30/30 C)
Deviation : 7.49 ppm

Figure S67: Dereplication analysis from MixONat, structure of dereplicated compounds: Rank **151-160**.

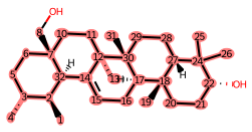

Rank: 161 MW: 442.72
LTS0263141
Score: 1.0 (30/30 C)
Deviation : 7.52 ppm

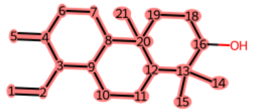

Rank: 162 MW: 288.47
LTS0182050
Score: 1.0 (20/20 C)
Deviation : 7.54 ppm

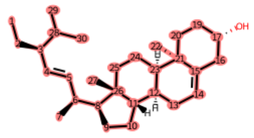

Rank: 163 MW: 412.69
LTS0125864
Score: 1.0 (29/29 C)
Deviation : 7.62 ppm

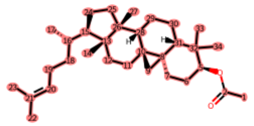

Rank: 164 MW: 468.76
LTS0252804
Score: 1.0 (32/32 C)
Deviation : 7.65 ppm

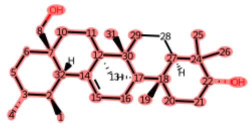

Rank: 165 MW: 442.72
LTS0136738
Score: 1.0 (30/30 C)
Deviation : 7.68 ppm

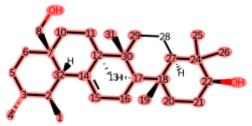

Rank: 166 MW: 442.72
LTS0008025
Score: 1.0 (30/30 C)
Deviation : 7.68 ppm

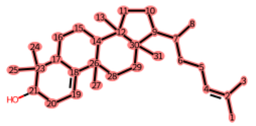

Rank: 167 MW: 426.72
LTS0033454
Score: 1.0 (30/30 C)
Deviation : 7.69 ppm

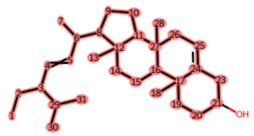

Rank: 168 MW: 426.72
LTS0200237
Score: 1.0 (30/30 C)
Deviation : 7.74 ppm

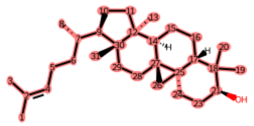

Rank: 169 MW: 426.72
LTS0072604
Score: 1.0 (30/30 C)
Deviation : 7.77 ppm

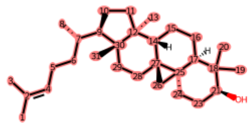

Rank: 170 MW: 426.72
LTS0028976
Score: 1.0 (30/30 C)
Deviation : 7.77 ppm

Figure S68: Dereplication analysis from MixONat, structure of dereplicated compounds: Rank **161-170**.

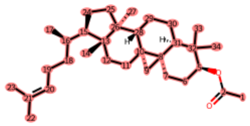

Rank: 171 MW: 468.76
LTS0161433
Score: 1.0 (32/32 C)
Deviation : 7.89 ppm

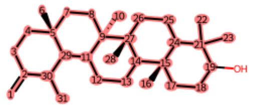

Rank: 172 MW: 426.72
LTS0274865
Score: 1.0 (30/30 C)
Deviation : 7.89 ppm

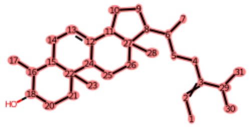

Rank: 173 MW: 426.72
LTS0024105
Score: 1.0 (30/30 C)
Deviation : 8.01 ppm

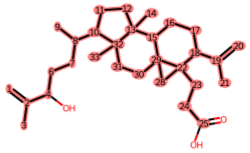

Rank: 174 MW: 456.7
LTS0084374
Score: 1.0 (30/30 C)
Deviation : 8.02 ppm

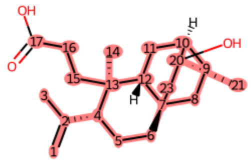

Rank: 175 MW: 320.47
LTS0205160
Score: 1.0 (20/20 C)
Deviation : 8.02 ppm

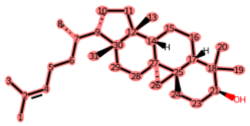

Rank: 176 MW: 426.72
LTS0092470
Score: 1.0 (30/30 C)
Deviation : 8.03 ppm

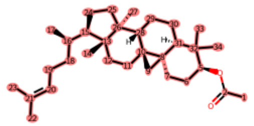

Rank: 177 MW: 468.76
LTS0217131
Score: 1.0 (32/32 C)
Deviation : 8.04 ppm

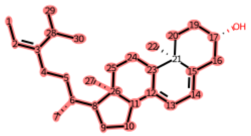

Rank: 178 MW: 410.68
LTS0129084
Score: 1.0 (28/28 C)
Deviation : 8.05 ppm

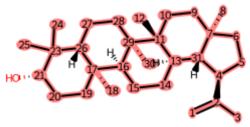

Rank: 179 MW: 426.72
LTS0121325
Score: 1.0 (30/30 C)
Deviation : 8.05 ppm

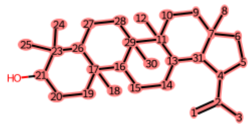

Rank: 180 MW: 426.72
LTS0088634
Score: 1.0 (30/30 C)
Deviation : 8.05 ppm

Figure S69: Dereplication analysis from MixONat, structure of dereplicated compounds: Rank **171-180**.

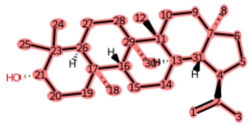

Rank: 181 MW: 426.72
LTS0193963
Score: 1.0 (30/30 C)
Deviation : 8.05 ppm

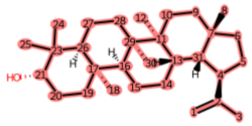

Rank: 182 MW: 426.72
LTS0183989
Score: 1.0 (30/30 C)
Deviation : 8.05 ppm

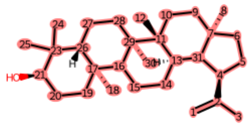

Rank: 183 MW: 426.72
LTS0250511
Score: 1.0 (30/30 C)
Deviation : 8.05 ppm

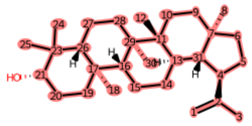

Rank: 184 MW: 426.72
LTS0256952
Score: 1.0 (30/30 C)
Deviation : 8.05 ppm

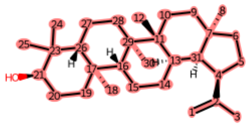

Rank: 185 MW: 426.72
LTS0255487
Score: 1.0 (30/30 C)
Deviation : 8.05 ppm

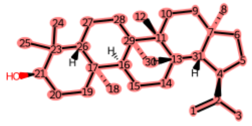

Rank: 186 MW: 426.72
LTS0228312
Score: 1.0 (30/30 C)
Deviation : 8.05 ppm

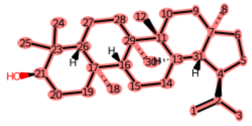

Rank: 187 MW: 426.72
LTS0108662
Score: 1.0 (30/30 C)
Deviation : 8.05 ppm

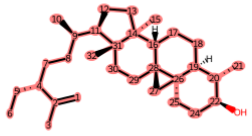

Rank: 188 MW: 440.75
LTS0003384
Score: 1.0 (31/31 C)
Deviation : 8.1 ppm

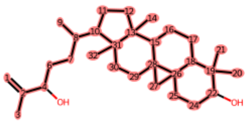

Rank: 189 MW: 442.72
LTS0049555
Score: 1.0 (30/30 C)
Deviation : 8.19 ppm

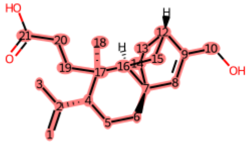

Rank: 190 MW: 318.45
LTS0256010
Score: 1.0 (20/20 C)
Deviation : 8.3 ppm

Figure S70: Dereplication analysis from MixONat, structure of dereplicated compounds: Rank **181-190**.

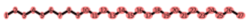

Rank: 191 MW: 380.73
LTS0150428
Score: 1.0 (27/27 C)
Deviation : 8.32 ppm

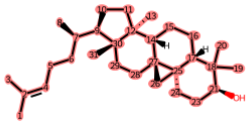

Rank: 192 MW: 426.72
LTS0061645
Score: 1.0 (30/30 C)
Deviation : 8.33 ppm

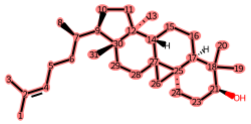

Rank: 193 MW: 426.72
LTS0062833
Score: 1.0 (30/30 C)
Deviation : 8.33 ppm

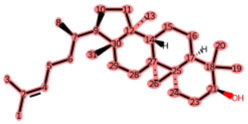

Rank: 194 MW: 426.72
LTS0260410
Score: 1.0 (30/30 C)
Deviation : 8.33 ppm

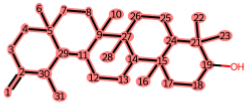

Rank: 195 MW: 426.72
LTS0256994
Score: 1.0 (30/30 C)
Deviation : 8.34 ppm

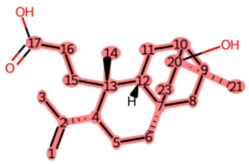

Rank: 196 MW: 320.47
LTS0152771
Score: 1.0 (20/20 C)
Deviation : 8.39 ppm

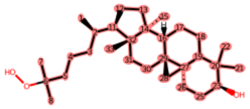

Rank: 197 MW: 460.73
LTS0199319
Score: 1.0 (30/30 C)
Deviation : 8.4 ppm

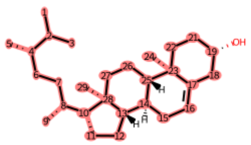

Rank: 198 MW: 400.68
LTS0204629
Score: 1.0 (28/28 C)
Deviation : 8.4 ppm

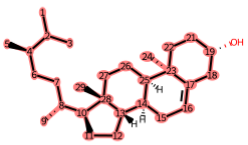

Rank: 199 MW: 400.68
LTS0165607
Score: 1.0 (28/28 C)
Deviation : 8.4 ppm

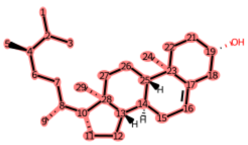

Rank: 200 MW: 400.68
LTS0029429
Score: 1.0 (28/28 C)
Deviation : 8.4 ppm

Figure S71: Dereplication analysis from MixONat, structure of dereplicated compounds: Rank **191-200**.

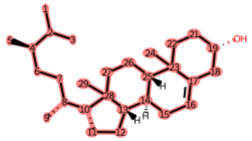

Rank: 201 MW: 400.68
LTS0057877
Score: 1.0 (28/28 C)
Deviation : 8.4 ppm

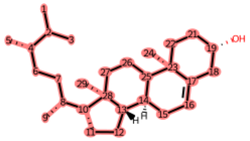

Rank: 202 MW: 400.68
LTS0050376
Score: 1.0 (28/28 C)
Deviation : 8.4 ppm

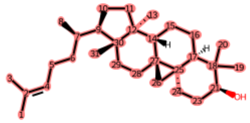

Rank: 203 MW: 426.72
LTS0269561
Score: 1.0 (30/30 C)
Deviation : 8.43 ppm

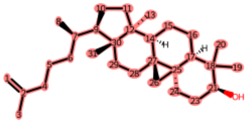

Rank: 204 MW: 426.72
LTS0057509
Score: 1.0 (30/30 C)
Deviation : 8.53 ppm

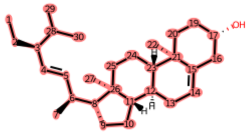

Rank: 205 MW: 412.69
LTS0169213
Score: 1.0 (29/29 C)
Deviation : 8.55 ppm

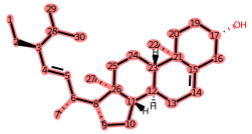

Rank: 206 MW: 412.69
LTS0029311
Score: 1.0 (29/29 C)
Deviation : 8.55 ppm

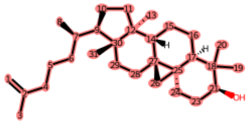

Rank: 207 MW: 426.72
LTS0255871
Score: 1.0 (30/30 C)
Deviation : 8.63 ppm

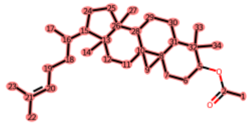

Rank: 208 MW: 468.76
LTS0026455
Score: 1.0 (32/32 C)
Deviation : 8.69 ppm

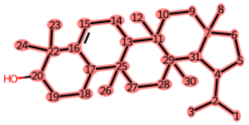

Rank: 209 MW: 426.72
LTS0205525
Score: 1.0 (30/30 C)
Deviation : 8.69 ppm

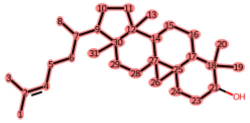

Rank: 210 MW: 426.72
LTS0060131
Score: 1.0 (30/30 C)
Deviation : 8.75 ppm

Figure S72: Dereplication analysis from MixONat, structure of dereplicated compounds: Rank **201-210**.

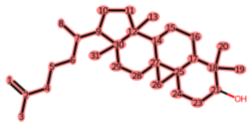

Rank: 211 MW: 426.72
LTS0208485
Score: 1.0 (30/30 C)
Deviation : 8.75 ppm

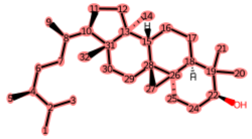

Rank: 212 MW: 442.76
LTS0075991
Score: 1.0 (31/31 C)
Deviation : 8.79 ppm

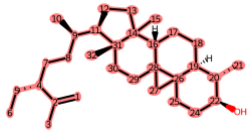

Rank: 213 MW: 440.75
LTS0265982
Score: 1.0 (31/31 C)
Deviation : 8.83 ppm

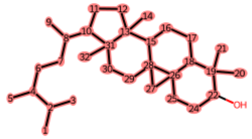

Rank: 214 MW: 442.76
LTS0044258
Score: 1.0 (31/31 C)
Deviation : 8.93 ppm

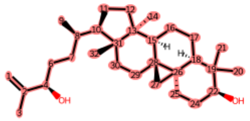

Rank: 215 MW: 442.72
LTS0141442
Score: 1.0 (30/30 C)
Deviation : 9.07 ppm

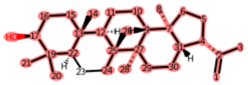

Rank: 216 MW: 426.72
LTS0163461
Score: 1.0 (30/30 C)
Deviation : 9.14 ppm

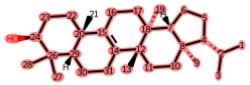

Rank: 217 MW: 426.72
LTS0093446
Score: 1.0 (30/30 C)
Deviation : 9.16 ppm

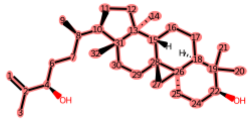

Rank: 218 MW: 442.72
LTS0109908
Score: 1.0 (30/30 C)
Deviation : 9.17 ppm

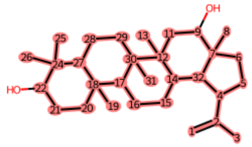

Rank: 219 MW: 442.72
LTS0031215
Score: 1.0 (30/30 C)
Deviation : 9.19 ppm

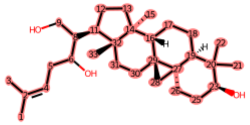

Rank: 220 MW: 458.72
LTS0078568
Score: 1.0 (30/30 C)
Deviation : 9.23 ppm

Figure S73: Dereplication analysis from MixONat, structure of dereplicated compounds: Rank **211-220**.

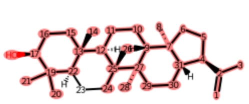

Rank: 221 MW: 426.72
LTS0060154
Score: 1.0 (30/30 C)
Deviation : 9.24 ppm

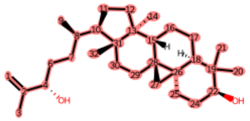

Rank: 222 MW: 442.72
LTS0134504
Score: 1.0 (30/30 C)
Deviation : 9.31 ppm

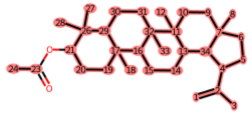

Rank: 223 MW: 468.76
LTS0081577
Score: 1.0 (32/32 C)
Deviation : 9.35 ppm

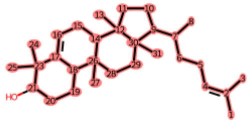

Rank: 224 MW: 426.72
LTS0175466
Score: 1.0 (30/30 C)
Deviation : 9.4 ppm

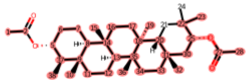

Rank: 225 MW: 528.81
LTS0118158
Score: 1.0 (34/34 C)
Deviation : 9.42 ppm

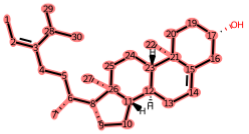

Rank: 226 MW: 412.69
LTS0103350
Score: 1.0 (29/29 C)
Deviation : 9.51 ppm

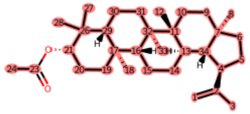

Rank: 227 MW: 468.76
LTS0077599
Score: 1.0 (32/32 C)
Deviation : 9.53 ppm

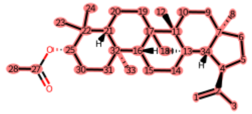

Rank: 228 MW: 468.76
LTS0154178
Score: 1.0 (32/32 C)
Deviation : 9.53 ppm

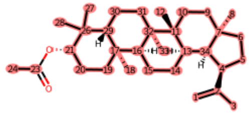

Rank: 229 MW: 468.76
LTS0207713
Score: 1.0 (32/32 C)
Deviation : 9.53 ppm

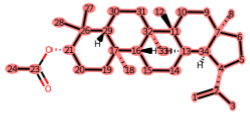

Rank: 230 MW: 468.76
LTS0022628
Score: 1.0 (32/32 C)
Deviation : 9.53 ppm

Figure S74: Dereplication analysis from MixONat, structure of dereplicated compounds: Rank **221-230**.

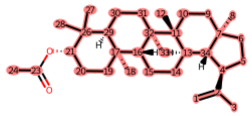

Rank: 231 MW: 468.76
LTS0031573
Score: 1.0 (32/32 C)
Deviation : 9.53 ppm

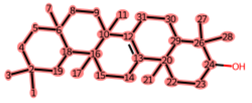

Rank: 232 MW: 426.72
LTS0263482
Score: 1.0 (30/30 C)
Deviation : 9.6 ppm

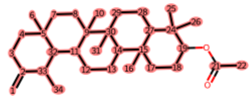

Rank: 233 MW: 468.76
LTS0026037
Score: 1.0 (32/32 C)
Deviation : 9.64 ppm

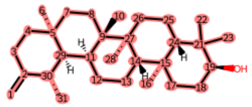

Rank: 234 MW: 426.72
LTS0231948
Score: 1.0 (30/30 C)
Deviation : 9.64 ppm

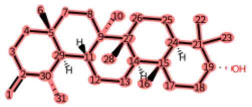

Rank: 235 MW: 426.72
LTS0272170
Score: 1.0 (30/30 C)
Deviation : 9.8 ppm

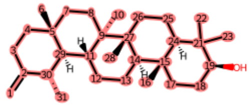

Rank: 236 MW: 426.72
LTS0210066
Score: 1.0 (30/30 C)
Deviation : 9.8 ppm

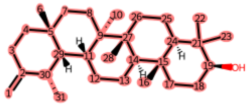

Rank: 237 MW: 426.72
LTS0006950
Score: 1.0 (30/30 C)
Deviation : 9.8 ppm

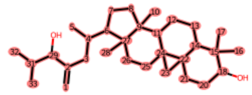

Rank: 238 MW: 456.74
LTS0168868
Score: 1.0 (31/31 C)
Deviation : 9.81 ppm

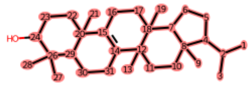

Rank: 239 MW: 426.72
LTS0107567
Score: 1.0 (30/30 C)
Deviation : 9.84 ppm

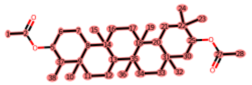

Rank: 240 MW: 528.81
LTS0270086
Score: 1.0 (34/34 C)
Deviation : 9.93 ppm

Figure S75: Dereplication analysis from MixONat, structure of dereplicated compounds: Rank **231-240**.

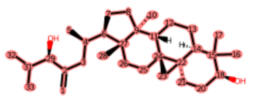

Rank: 241 MW: 456.74
LTS0150813
Score: 1.0 (31/31 C)
Deviation : 9.98 ppm

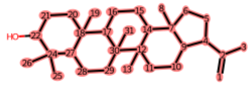

Rank: 242 MW: 426.72
LTS0002506
Score: 1.0 (30/30 C)
Deviation : 10.24 ppm

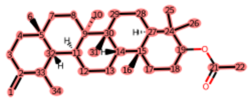

Rank: 243 MW: 468.76
LTS0117429
Score: 1.0 (32/32 C)
Deviation : 10.44 ppm

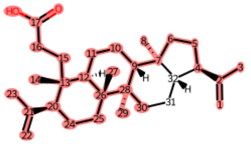

Rank: 244 MW: 440.7
LTS0176169
Score: 1.0 (30/30 C)
Deviation : 10.52 ppm

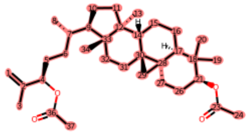

Rank: 245 MW: 526.79
LTS0105863
Score: 1.0 (34/34 C)
Deviation : 10.57 ppm

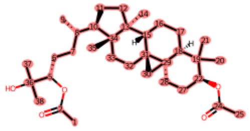

Rank: 246 MW: 544.81
LTS0009360
Score: 1.0 (34/34 C)
Deviation : 10.95 ppm

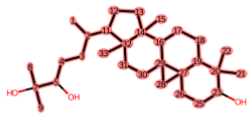

Rank: 247 MW: 460.73
LTS0139499
Score: 1.0 (30/30 C)
Deviation : 10.96 ppm

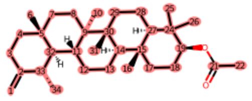

Rank: 248 MW: 468.76
LTS0190545
Score: 1.0 (32/32 C)
Deviation : 11.25 ppm

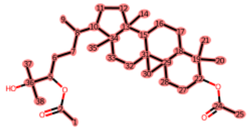

Rank: 249 MW: 544.81
LTS0073059
Score: 1.0 (34/34 C)
Deviation : 11.26 ppm

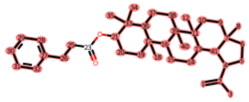

Rank: 250 MW: 558.88
LTS0169332
Score: 0.97 (38/39 C)
Deviation : 10.97 ppm

Figure S76: Dereplication analysis from MixONat, structure of dereplicated compounds: Rank **241-250**.

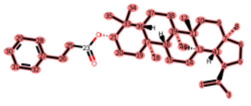

Rank: 251 MW: 558.88
LTS0093820
Score: 0.97 (38/39 C)
Deviation : 10.97 ppm

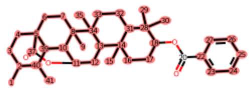

Rank: 252 MW: 560.81
LTS0151966
Score: 0.97 (36/37 C)
Deviation : 6.62 ppm

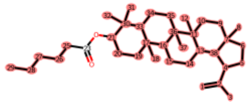

Rank: 253 MW: 524.86
LTS0064484
Score: 0.97 (35/36 C)
Deviation : 9.74 ppm

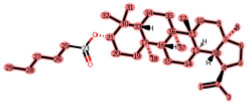

Rank: 254 MW: 524.86
LTS0059576
Score: 0.97 (35/36 C)
Deviation : 10.29 ppm

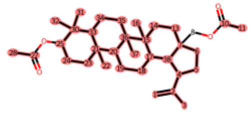

Rank: 255 MW: 526.79
LTS0130209
Score: 0.97 (33/34 C)
Deviation : 9.66 ppm

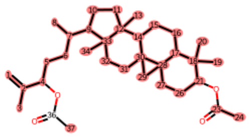

Rank: 256 MW: 526.79
LTS0210707
Score: 0.97 (33/34 C)
Deviation : 9.77 ppm

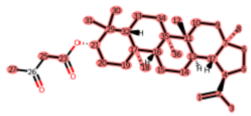

Rank: 257 MW: 510.79
LTS0224230
Score: 0.97 (33/34 C)
Deviation : 11.09 ppm

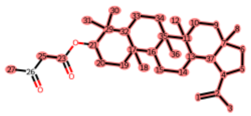

Rank: 258 MW: 510.79
LTS0248425
Score: 0.97 (33/34 C)
Deviation : 11.09 ppm

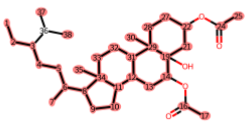

Rank: 259 MW: 532.8
LTS0172837
Score: 0.97 (32/33 C)
Deviation : 8.3 ppm

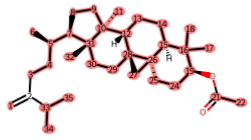

Rank: 260 MW: 482.78
LTS0199596
Score: 0.97 (32/33 C)
Deviation : 8.51 ppm

Figure S77: Dereplication analysis from MixONat, structure of dereplicated compounds: Rank **251-260**.

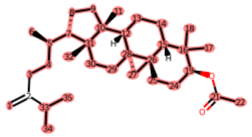

Rank: 261 MW: 482.78
LTS0193295
Score: 0.97 (32/33 C)
Deviation : 8.67 ppm

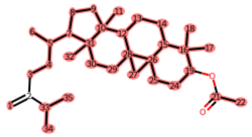

Rank: 262 MW: 482.78
LTS0163383
Score: 0.97 (32/33 C)
Deviation : 10.68 ppm

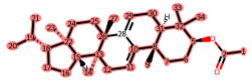

Rank: 263 MW: 466.74
LTS0217242
Score: 0.97 (31/32 C)
Deviation : 7.1 ppm

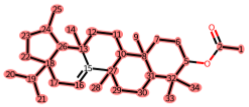

Rank: 264 MW: 468.76
LTS0251090
Score: 0.97 (31/32 C)
Deviation : 7.28 ppm

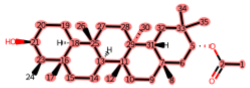

Rank: 265 MW: 486.77
LTS0210805
Score: 0.97 (31/32 C)
Deviation : 7.61 ppm

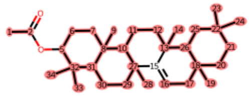

Rank: 266 MW: 468.76
LTS0047312
Score: 0.97 (31/32 C)
Deviation : 7.85 ppm

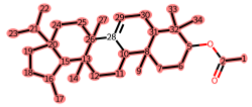

Rank: 267 MW: 468.76
LTS0230737
Score: 0.97 (31/32 C)
Deviation : 7.94 ppm

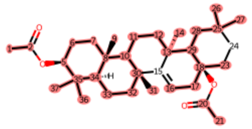

Rank: 268 MW: 512.76
LTS0074761
Score: 0.97 (31/32 C)
Deviation : 7.95 ppm

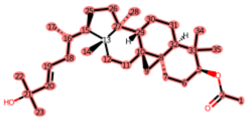

Rank: 269 MW: 484.75
LTS0083998
Score: 0.97 (31/32 C)
Deviation : 7.98 ppm

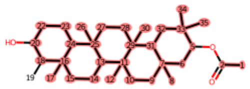

Rank: 270 MW: 486.77
LTS0061013
Score: 0.97 (31/32 C)
Deviation : 8.04 ppm

Figure S78: Dereplication analysis from MixONat, structure of dereplicated compounds: Rank **261-270**.

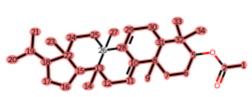

Rank: 271 MW: 466.74
LTS0115136
Score: 0.97 (31/32 C)
Deviation : 8.37 ppm

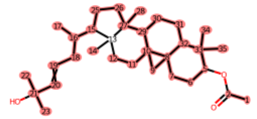

Rank: 272 MW: 484.75
LTS0154491
Score: 0.97 (31/32 C)
Deviation : 8.61 ppm

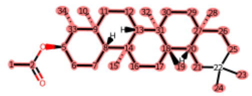

Rank: 273 MW: 470.77
LTS0211471
Score: 0.97 (31/32 C)
Deviation : 9.19 ppm

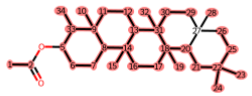

Rank: 274 MW: 470.77
LTS0169461
Score: 0.97 (31/32 C)
Deviation : 9.23 ppm

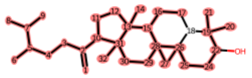

Rank: 275 MW: 440.75
LTS0106700
Score: 0.97 (30/31 C)
Deviation : 5.61 ppm

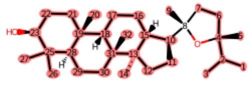

Rank: 276 MW: 458.76
LTS0094713
Score: 0.97 (30/31 C)
Deviation : 7.27 ppm

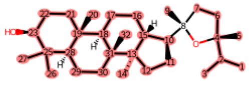

Rank: 277 MW: 458.76
LTS0019263
Score: 0.97 (30/31 C)
Deviation : 7.27 ppm

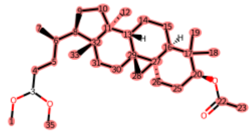

Rank: 278 MW: 488.74
LTS0026839
Score: 0.97 (30/31 C)
Deviation : 7.28 ppm

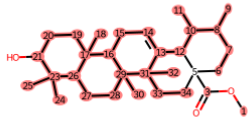

Rank: 279 MW: 470.73
LTS0155914
Score: 0.97 (30/31 C)
Deviation : 7.35 ppm

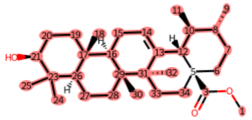

Rank: 280 MW: 470.73
LTS0086911
Score: 0.97 (30/31 C)
Deviation : 7.37 ppm

Figure S79: Dereplication analysis from MixONat, structure of dereplicated compounds: Rank **271-280**.

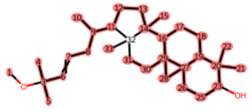

Rank: 281 MW: 456.74
LTS0209109
Score: 0.97 (30/31 C)
Deviation : 7.9 ppm

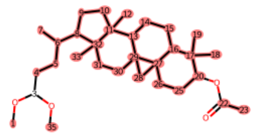

Rank: 282 MW: 488.74
LTS0133783
Score: 0.97 (30/31 C)
Deviation : 8.02 ppm

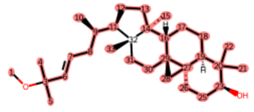

Rank: 283 MW: 456.74
LTS0125088
Score: 0.97 (30/31 C)
Deviation : 8.16 ppm

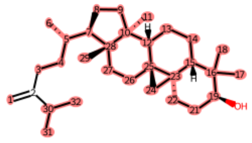

Rank: 284 MW: 440.75
LTS0078310
Score: 0.97 (30/31 C)
Deviation : 8.62 ppm

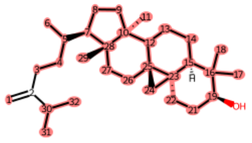

Rank: 285 MW: 440.75
LTS0039804
Score: 0.97 (30/31 C)
Deviation : 8.72 ppm

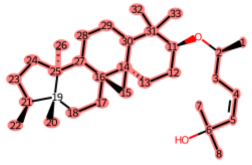

Rank: 286 MW: 456.74
LTS0115025
Score: 0.97 (30/31 C)
Deviation : 8.8 ppm

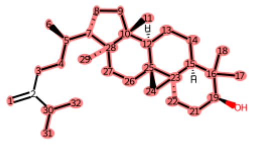

Rank: 287 MW: 440.75
LTS0138719
Score: 0.97 (30/31 C)
Deviation : 8.82 ppm

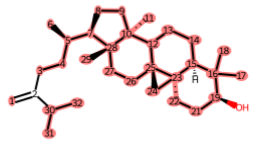

Rank: 288 MW: 440.75
LTS0077845
Score: 0.97 (30/31 C)
Deviation : 8.92 ppm

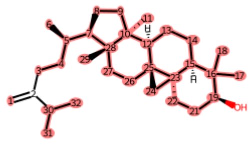

Rank: 289 MW: 440.75
LTS0193059
Score: 0.97 (30/31 C)
Deviation : 8.92 ppm

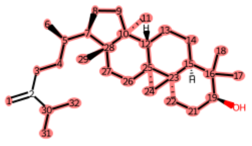

Rank: 290 MW: 440.75
LTS0219542
Score: 0.97 (30/31 C)
Deviation : 8.92 ppm

Figure S80: Dereplication analysis from MixONat, structure of dereplicated compounds: Rank **281-290**.

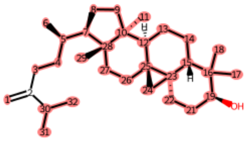

Rank: 291 MW: 440.75
LTS0040878
Score: 0.97 (30/31 C)
Deviation : 8.92 ppm

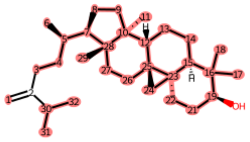

Rank: 292 MW: 440.75
LTS0018584
Score: 0.97 (30/31 C)
Deviation : 9.02 ppm

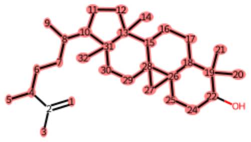

Rank: 293 MW: 440.75
LTS0091769
Score: 0.97 (30/31 C)
Deviation : 9.69 ppm

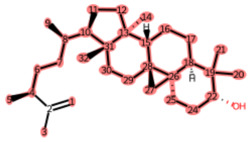

Rank: 294 MW: 440.75
LTS0170111
Score: 0.97 (30/31 C)
Deviation : 10.05 ppm

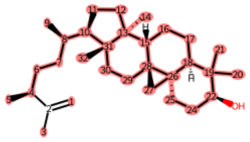

Rank: 295 MW: 440.75
LTS0231558
Score: 0.97 (30/31 C)
Deviation : 10.15 ppm

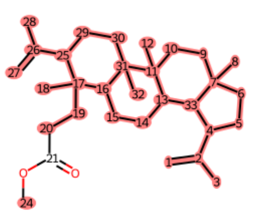

Rank: 296 MW: 454.73
LTS0251756
Score: 0.97 (30/31 C)
Deviation : 10.34 ppm

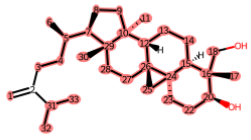

Rank: 297 MW: 456.74
LTS0210385
Score: 0.97 (30/31 C)
Deviation : 10.35 ppm

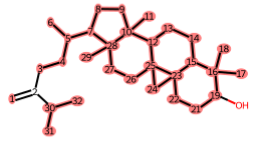

Rank: 298 MW: 440.75
LTS0084326
Score: 0.97 (30/31 C)
Deviation : 10.39 ppm

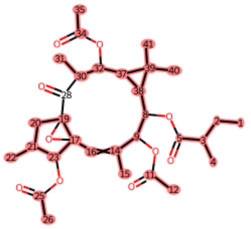

Rank: 299 MW: 576.68
LTS0136700
Score: 0.97 (30/31 C)
Deviation : 10.42 ppm

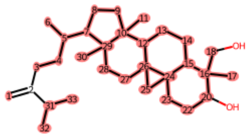

Rank: 300 MW: 456.74
LTS0246775
Score: 0.97 (30/31 C)
Deviation : 11.46 ppm

Figure S81: Dereplication analysis from MixONat, structure of dereplicated compounds: Rank **291-300**.

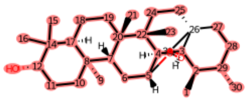

Rank: 301 MW: 456.7
LTS0015731
Score: 0.97 (29/30 C)
Deviation : 3.72 ppm

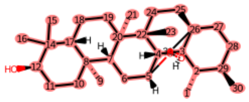

Rank: 302 MW: 456.7
LTS0074100
Score: 0.97 (29/30 C)
Deviation : 3.78 ppm

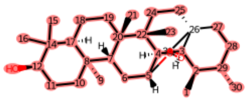

Rank: 303 MW: 456.7
LTS0176408
Score: 0.97 (29/30 C)
Deviation : 3.8 ppm

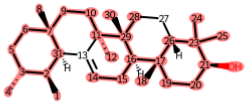

Rank: 304 MW: 426.72
LTS0227808
Score: 0.97 (29/30 C)
Deviation : 5.12 ppm

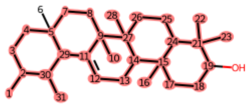

Rank: 305 MW: 426.72
LTS0088267
Score: 0.97 (29/30 C)
Deviation : 5.12 ppm

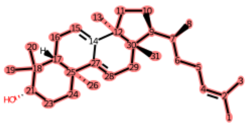

Rank: 306 MW: 424.7
LTS0022408
Score: 0.97 (29/30 C)
Deviation : 5.19 ppm

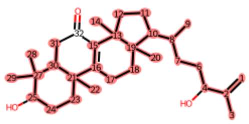

Rank: 307 MW: 456.7
LTS0221514
Score: 0.97 (29/30 C)
Deviation : 5.37 ppm

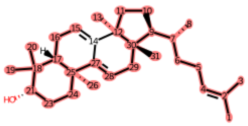

Rank: 308 MW: 424.7
LTS0168053
Score: 0.97 (29/30 C)
Deviation : 5.49 ppm

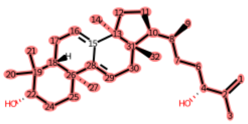

Rank: 309 MW: 440.7
LTS0106974
Score: 0.97 (29/30 C)
Deviation : 5.73 ppm

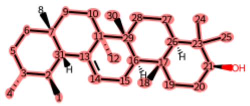

Rank: 310 MW: 426.72
LTS0222826
Score: 0.97 (29/30 C)
Deviation : 5.95 ppm

Figure S82: Dereplication analysis from MixONat, structure of dereplicated compounds: Rank **301-310**.

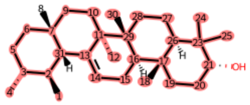

Rank: 311 MW: 426.72
LTS0095922
Score: 0.97 (29/30 C)
Deviation : 5.95 ppm

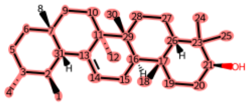

Rank: 312 MW: 426.72
LTS0042180
Score: 0.97 (29/30 C)
Deviation : 5.95 ppm

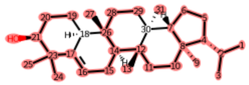

Rank: 313 MW: 426.72
LTS0175295
Score: 0.97 (29/30 C)
Deviation : 5.99 ppm

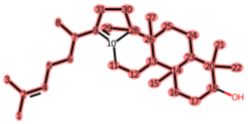

Rank: 314 MW: 426.72
LTS0140527
Score: 0.97 (29/30 C)
Deviation : 6.03 ppm

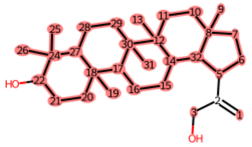

Rank: 315 MW: 442.72
LTS0191991
Score: 0.97 (29/30 C)
Deviation : 6.04 ppm

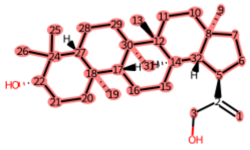

Rank: 316 MW: 442.72
LTS0182745
Score: 0.97 (29/30 C)
Deviation : 6.12 ppm

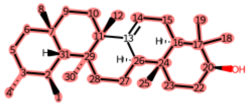

Rank: 317 MW: 426.72
LTS0066104
Score: 0.97 (29/30 C)
Deviation : 6.21 ppm

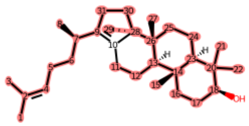

Rank: 318 MW: 426.72
LTS0176445
Score: 0.97 (29/30 C)
Deviation : 6.23 ppm

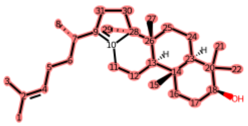

Rank: 319 MW: 426.72
LTS0265799
Score: 0.97 (29/30 C)
Deviation : 6.25 ppm

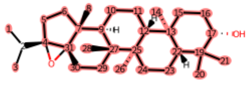

Rank: 320 MW: 442.72
LTS0094704
Score: 0.97 (29/30 C)
Deviation : 6.3 ppm

Figure S83: Dereplication analysis from MixONat, structure of dereplicated compounds: Rank **311-320**.

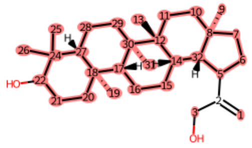

Rank: 321 MW: 442.72
LTS0272446
Score: 0.97 (29/30 C)
Deviation : 6.3 ppm

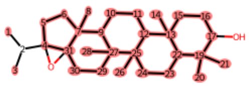

Rank: 322 MW: 442.72
LTS0001153
Score: 0.97 (29/30 C)
Deviation : 6.36 ppm

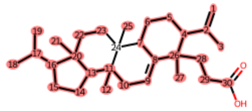

Rank: 323 MW: 440.7
LTS0004192
Score: 0.97 (29/30 C)
Deviation : 6.43 ppm

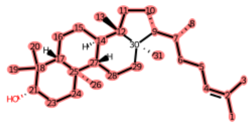

Rank: 324 MW: 428.73
LTS0082195
Score: 0.97 (29/30 C)
Deviation : 6.81 ppm

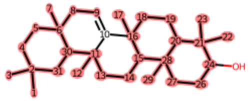

Rank: 325 MW: 426.72
LTS0019099
Score: 0.97 (29/30 C)
Deviation : 6.84 ppm

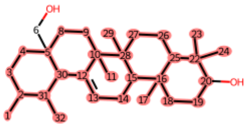

Rank: 326 MW: 442.72
LTS0178123
Score: 0.97 (29/30 C)
Deviation : 6.97 ppm

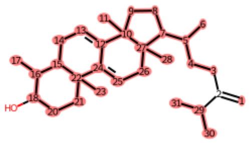

Rank: 327 MW: 424.7
LTS0162997
Score: 0.97 (29/30 C)
Deviation : 6.99 ppm

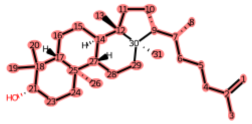

Rank: 328 MW: 428.73
LTS0049801
Score: 0.97 (29/30 C)
Deviation : 7.03 ppm

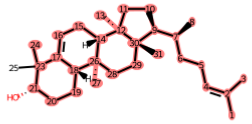

Rank: 329 MW: 426.72
LTS0102270
Score: 0.97 (29/30 C)
Deviation : 7.22 ppm

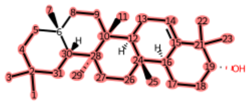

Rank: 330 MW: 426.72
LTS0020705
Score: 0.97 (29/30 C)
Deviation : 7.24 ppm

Figure S84: Dereplication analysis from MixONat, structure of dereplicated compounds: Rank **321-330**.

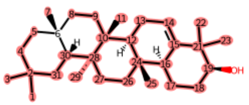

Rank: 331 MW: 426.72
LTS0082830
Score: 0.97 (29/30 C)
Deviation : 7.29 ppm

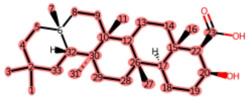

Rank: 332 MW: 458.72
LTS0058300
Score: 0.97 (29/30 C)
Deviation : 7.4 ppm

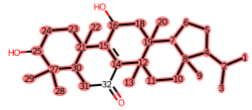

Rank: 333 MW: 456.7
LTS0113831
Score: 0.97 (29/30 C)
Deviation : 7.45 ppm

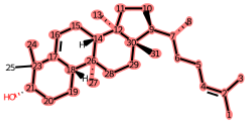

Rank: 334 MW: 426.72
LTS0257028
Score: 0.97 (29/30 C)
Deviation : 7.52 ppm

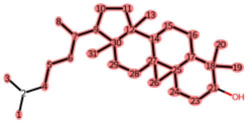

Rank: 335 MW: 428.73
LTS0059585
Score: 0.97 (29/30 C)
Deviation : 7.58 ppm

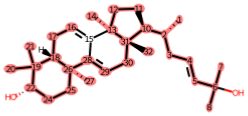

Rank: 336 MW: 440.7
LTS0154382
Score: 0.97 (29/30 C)
Deviation : 7.61 ppm

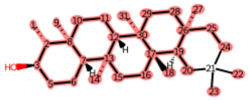

Rank: 337 MW: 428.73
LTS0159359
Score: 0.97 (29/30 C)
Deviation : 7.68 ppm

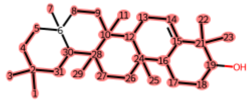

Rank: 338 MW: 426.72
LTS0038580
Score: 0.97 (29/30 C)
Deviation : 7.7 ppm

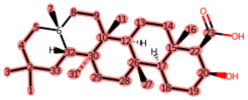

Rank: 339 MW: 458.72
LTS0104019
Score: 0.97 (29/30 C)
Deviation : 7.73 ppm

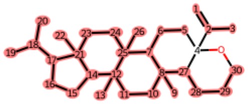

Rank: 340 MW: 426.72
LTS0207356
Score: 0.97 (29/30 C)
Deviation : 7.77 ppm

Figure S85: Dereplication analysis from MixONat, structure of dereplicated compounds: Rank **331-340**.

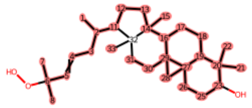

Rank: 341 MW: 458.72
LTS0111501
Score: 0.97 (29/30 C)
Deviation : 7.82 ppm

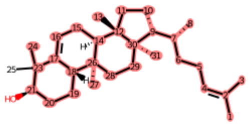

Rank: 342 MW: 426.72
LTS0201674
Score: 0.97 (29/30 C)
Deviation : 7.84 ppm

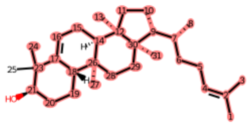

Rank: 343 MW: 426.72
LTS0250416
Score: 0.97 (29/30 C)
Deviation : 7.84 ppm

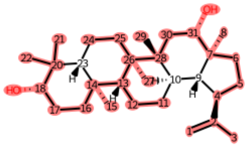

Rank: 344 MW: 442.72
LTS0017475
Score: 0.97 (29/30 C)
Deviation : 7.87 ppm

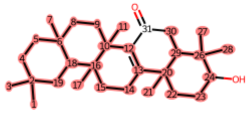

Rank: 345 MW: 440.7
LTS0044477
Score: 0.97 (29/30 C)
Deviation : 7.9 ppm

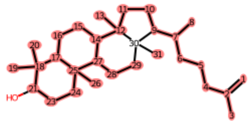

Rank: 346 MW: 428.73
LTS0241648
Score: 0.97 (29/30 C)
Deviation : 7.91 ppm

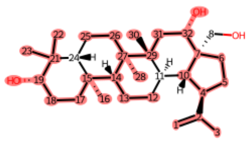

Rank: 347 MW: 458.72
LTS0191324
Score: 0.97 (29/30 C)
Deviation : 7.95 ppm

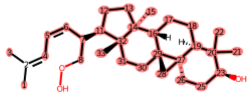

Rank: 348 MW: 456.7
LTS0255447
Score: 0.97 (29/30 C)
Deviation : 7.95 ppm

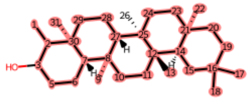

Rank: 349 MW: 428.73
LTS0183109
Score: 0.97 (29/30 C)
Deviation : 7.97 ppm

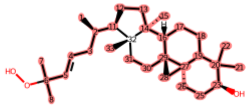

Rank: 350 MW: 458.72
LTS0148591
Score: 0.97 (29/30 C)
Deviation : 7.98 ppm

Figure S86: Dereplication analysis from MixONat, structure of dereplicated compounds: Rank **341-350**.

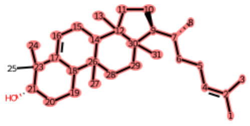

Rank: 351 MW: 426.72
LTS0082127
Score: 0.97 (29/30 C)
Deviation : 7.98 ppm

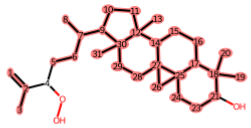

Rank: 352 MW: 458.72
LTS0051117
Score: 0.97 (29/30 C)
Deviation : 8.0 ppm

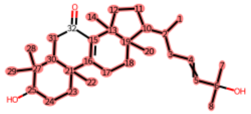

Rank: 353 MW: 456.7
LTS0230498
Score: 0.97 (29/30 C)
Deviation : 8.02 ppm

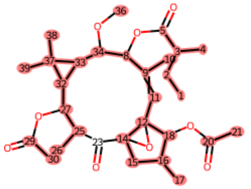

Rank: 354 MW: 548.67
LTS0085638
Score: 0.97 (29/30 C)
Deviation : 8.03 ppm

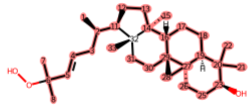

Rank: 355 MW: 458.72
LTS0167763
Score: 0.97 (29/30 C)
Deviation : 8.08 ppm

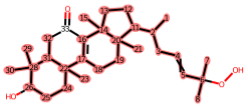

Rank: 356 MW: 472.7
LTS0109408
Score: 0.97 (29/30 C)
Deviation : 8.09 ppm

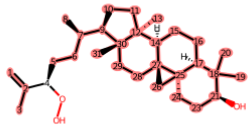

Rank: 357 MW: 458.72
LTS0033019
Score: 0.97 (29/30 C)
Deviation : 8.11 ppm

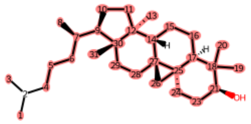

Rank: 358 MW: 428.73
LTS0176903
Score: 0.97 (29/30 C)
Deviation : 8.18 ppm

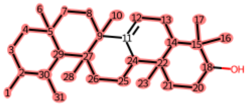

Rank: 359 MW: 426.72
LTS0151229
Score: 0.97 (29/30 C)
Deviation : 8.22 ppm

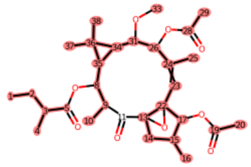

Rank: 360 MW: 548.67
LTS0027386
Score: 0.97 (29/30 C)
Deviation : 8.23 ppm

Figure S87: Dereplication analysis from MixONat, structure of dereplicated compounds: Rank **351-360**.

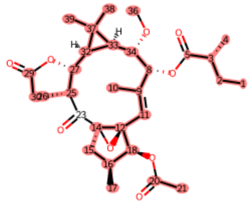

Rank: 361 MW: 548.67
LTS0245374
Score: 0.97 (29/30 C)
Deviation : 8.25 ppm

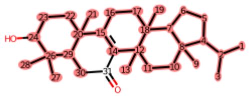

Rank: 362 MW: 440.7
LTS0083193
Score: 0.97 (29/30 C)
Deviation : 8.25 ppm

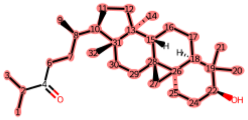

Rank: 363 MW: 442.72
LTS0155653
Score: 0.97 (29/30 C)
Deviation : 8.29 ppm

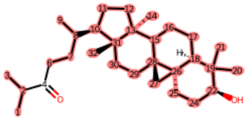

Rank: 364 MW: 442.72
LTS0026546
Score: 0.97 (29/30 C)
Deviation : 8.37 ppm

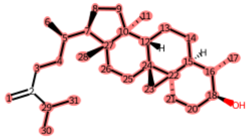

Rank: 365 MW: 426.72
LTS0125739
Score: 0.97 (29/30 C)
Deviation : 8.45 ppm

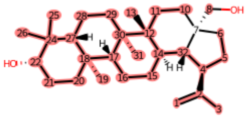

Rank: 366 MW: 442.72
LTS0101863
Score: 0.97 (29/30 C)
Deviation : 8.47 ppm

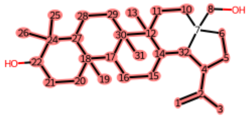

Rank: 367 MW: 442.72
LTS0008250
Score: 0.97 (29/30 C)
Deviation : 8.47 ppm

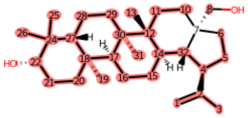

Rank: 368 MW: 442.72
LTS0258663
Score: 0.97 (29/30 C)
Deviation : 8.47 ppm

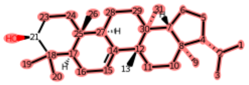

Rank: 369 MW: 426.72
LTS0001668
Score: 0.97 (29/30 C)
Deviation : 8.48 ppm

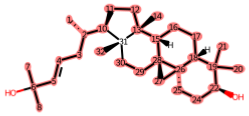

Rank: 370 MW: 442.72
LTS0088281
Score: 0.97 (29/30 C)
Deviation : 8.49 ppm

Figure S88: Dereplication analysis from MixONat, structure of dereplicated compounds: Rank **361-370**.

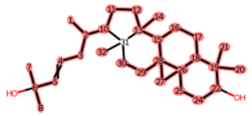

Rank: 371 MW: 442.72
LTS0044138
Score: 0.97 (29/30 C)
Deviation : 8.51 ppm

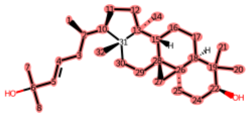

Rank: 372 MW: 442.72
LTS0201732
Score: 0.97 (29/30 C)
Deviation : 8.51 ppm

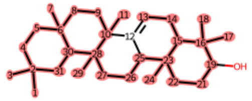

Rank: 373 MW: 426.72
LTS0036231
Score: 0.97 (29/30 C)
Deviation : 8.78 ppm

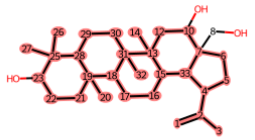

Rank: 374 MW: 458.72
LTS0029127
Score: 0.97 (29/30 C)
Deviation : 8.8 ppm

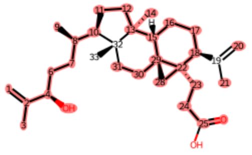

Rank: 375 MW: 456.7
LTS0168531
Score: 0.97 (29/30 C)
Deviation : 9.6 ppm

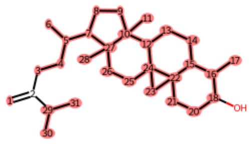

Rank: 376 MW: 426.72
LTS0042683
Score: 0.97 (29/30 C)
Deviation : 9.9 ppm

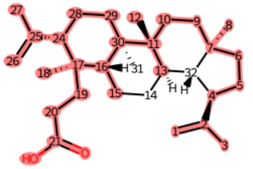

Rank: 377 MW: 440.7
LTS0244499
Score: 0.97 (29/30 C)
Deviation : 10.41 ppm

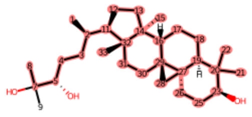

Rank: 378 MW: 460.73
LTS0230722
Score: 0.97 (29/30 C)
Deviation : 10.54 ppm

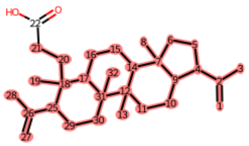

Rank: 379 MW: 440.7
LTS0130792
Score: 0.97 (29/30 C)
Deviation : 10.89 ppm

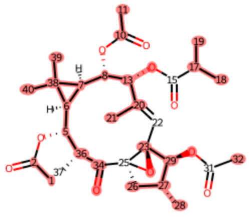

Rank: 380 MW: 562.65
LTS0179670
Score: 0.97 (29/30 C)
Deviation : 10.95 ppm

Figure S89: Dereplication analysis from MixONat, structure of dereplicated compounds: Rank **371-380**.

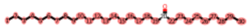

Rank: 381 MW: 422.77
LTS0250310
Score: 0.97 (28/29 C)
Deviation : 3.69 ppm

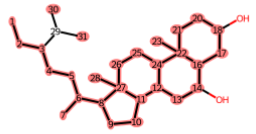

Rank: 382 MW: 432.72
LTS0203987
Score: 0.97 (28/29 C)
Deviation : 4.06 ppm

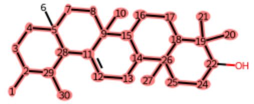

Rank: 383 MW: 412.69
LTS0250490
Score: 0.97 (28/29 C)
Deviation : 5.78 ppm

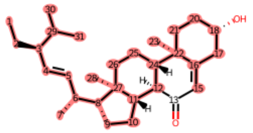

Rank: 384 MW: 426.68
LTS0025704
Score: 0.97 (28/29 C)
Deviation : 6.06 ppm

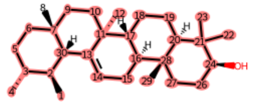

Rank: 385 MW: 412.69
LTS0027944
Score: 0.97 (28/29 C)
Deviation : 6.06 ppm

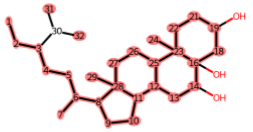

Rank: 386 MW: 448.72
LTS0047756
Score: 0.97 (28/29 C)
Deviation : 6.38 ppm

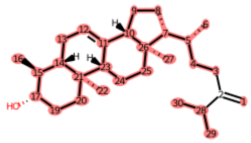

Rank: 387 MW: 412.69
LTS0024189
Score: 0.97 (28/29 C)
Deviation : 6.51 ppm

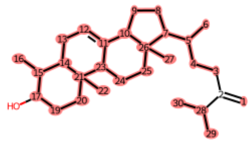

Rank: 388 MW: 412.69
LTS0172153
Score: 0.97 (28/29 C)
Deviation : 6.66 ppm

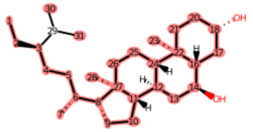

Rank: 389 MW: 432.72
LTS0215026
Score: 0.97 (28/29 C)
Deviation : 6.66 ppm

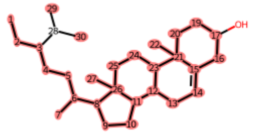

Rank: 390 MW: 414.71
LTS0204616
Score: 0.97 (28/29 C)
Deviation : 6.87 ppm

Figure S90: Dereplication analysis from MixONat, structure of dereplicated compounds: Rank **381-390**.

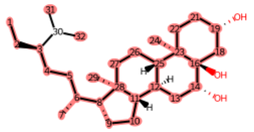

Rank: 391 MW: 448.72
LTS0093459
Score: 0.97 (28/29 C)
Deviation : 6.96 ppm

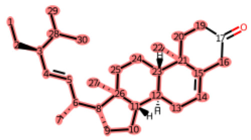

Rank: 392 MW: 410.68
LTS0161389
Score: 0.97 (28/29 C)
Deviation : 7.28 ppm

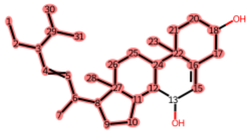

Rank: 393 MW: 428.69
LTS0007957
Score: 0.97 (28/29 C)
Deviation : 7.31 ppm

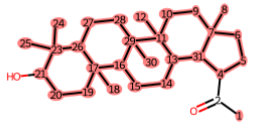

Rank: 394 MW: 428.69
LTS0222305
Score: 0.97 (28/29 C)
Deviation : 7.6 ppm

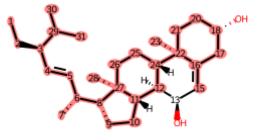

Rank: 395 MW: 428.69
LTS0162005
Score: 0.97 (28/29 C)
Deviation : 7.76 ppm

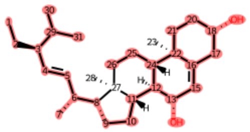

Rank: 396 MW: 428.69
LTS0206976
Score: 0.97 (28/29 C)
Deviation : 7.76 ppm

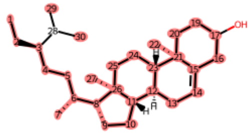

Rank: 397 MW: 414.71
LTS0071224
Score: 0.97 (28/29 C)
Deviation : 7.77 ppm

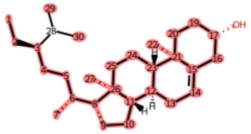

Rank: 398 MW: 414.71
LTS0168132
Score: 0.97 (28/29 C)
Deviation : 7.77 ppm

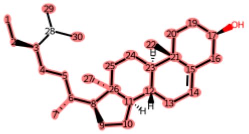

Rank: 399 MW: 414.71
LTS0234781
Score: 0.97 (28/29 C)
Deviation : 7.77 ppm

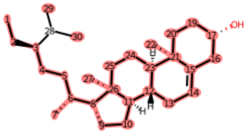

Rank: 400 MW: 414.71
LTS0129695
Score: 0.97 (28/29 C)
Deviation : 7.77 ppm

Figure S91: Dereplication analysis from MixONat, structure of dereplicated compounds: Rank **391-400**.

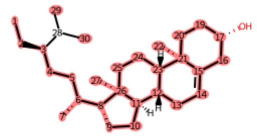

Rank: 401 MW: 414.71
LTS0050520
Score: 0.97 (28/29 C)
Deviation : 7.77 ppm

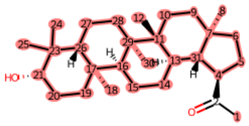

Rank: 402 MW: 428.69
LTS0045927
Score: 0.97 (28/29 C)
Deviation : 7.86 ppm

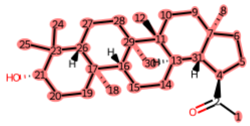

Rank: 403 MW: 428.69
LTS0122908
Score: 0.97 (28/29 C)
Deviation : 7.86 ppm

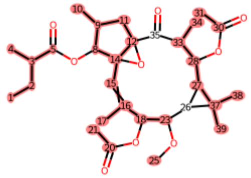

Rank: 404 MW: 548.67
LTS0166799
Score: 0.97 (28/29 C)
Deviation : 7.95 ppm

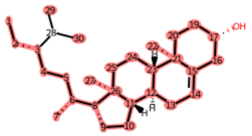

Rank: 405 MW: 414.71
LTS0248660
Score: 0.97 (28/29 C)
Deviation : 7.97 ppm

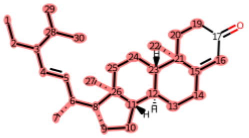

Rank: 406 MW: 410.68
LTS0092943
Score: 0.97 (28/29 C)
Deviation : 8.26 ppm

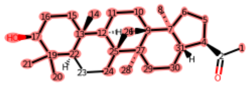

Rank: 407 MW: 428.69
LTS0010988
Score: 0.97 (28/29 C)
Deviation : 8.36 ppm

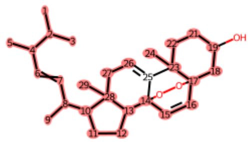

Rank: 408 MW: 426.63
LTS0146648
Score: 0.96 (27/28 C)
Deviation : 5.02 ppm

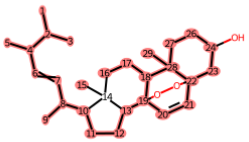

Rank: 409 MW: 428.65
LTS0211330
Score: 0.96 (27/28 C)
Deviation : 5.07 ppm

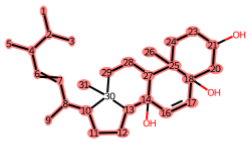

Rank: 410 MW: 430.66
LTS0057531
Score: 0.96 (27/28 C)
Deviation : 5.07 ppm

Figure S92: Dereplication analysis from MixONat, structure of dereplicated compounds: Rank **401-410**.

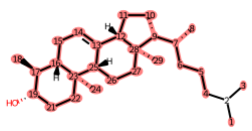

Rank: 411 MW: 400.68
LTS0271727
Score: 0.96 (27/28 C)
Deviation : 5.37 ppm

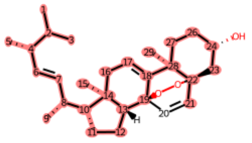

Rank: 412 MW: 426.63
LTS0148355
Score: 0.96 (27/28 C)
Deviation : 5.5 ppm

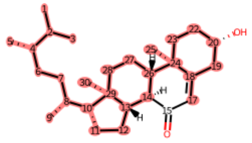

Rank: 413 MW: 414.66
LTS0239641
Score: 0.96 (27/28 C)
Deviation : 5.79 ppm

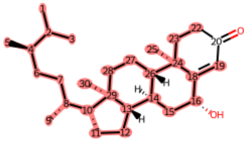

Rank: 414 MW: 414.66
LTS0247160
Score: 0.96 (27/28 C)
Deviation : 5.9 ppm

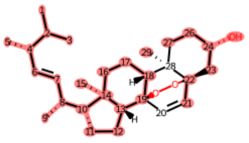

Rank: 415 MW: 428.65
LTS0264710
Score: 0.96 (27/28 C)
Deviation : 5.9 ppm

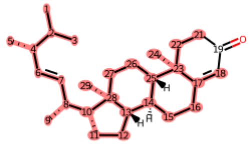

Rank: 416 MW: 396.65
LTS0268130
Score: 0.96 (27/28 C)
Deviation : 6.27 ppm

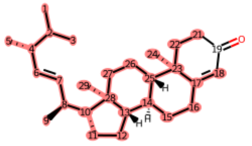

Rank: 417 MW: 396.65
LTS0003366
Score: 0.96 (27/28 C)
Deviation : 6.27 ppm

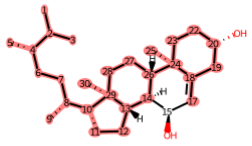

Rank: 418 MW: 416.68
LTS0217262
Score: 0.96 (27/28 C)
Deviation : 6.86 ppm

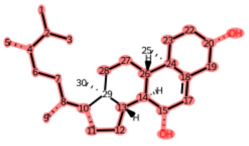

Rank: 419 MW: 416.68
LTS0018780
Score: 0.96 (27/28 C)
Deviation : 6.86 ppm

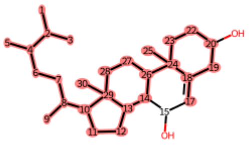

Rank: 420 MW: 416.68
LTS0095810
Score: 0.96 (27/28 C)
Deviation : 7.36 ppm

Figure S93: Dereplication analysis from MixONat, structure of dereplicated compounds: Rank **411-420**.

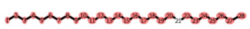

Rank: 421 MW: 394.76
LTS0242387
Score: 0.96 (27/28 C)
Deviation : 8.32 ppm

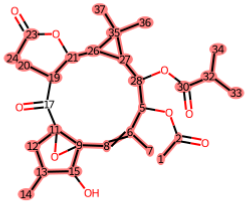

Rank: 422 MW: 520.61
LTS0065316
Score: 0.96 (27/28 C)
Deviation : 9.18 ppm

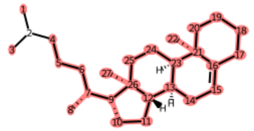

Rank: 423 MW: 370.66
LTS0195417
Score: 0.96 (26/27 C)
Deviation : 6.42 ppm

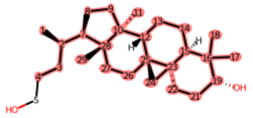

Rank: 424 MW: 402.65
LTS0083646
Score: 0.96 (26/27 C)
Deviation : 7.21 ppm

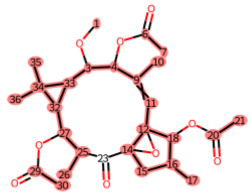

Rank: 425 MW: 506.59
LTS0274044
Score: 0.96 (26/27 C)
Deviation : 7.73 ppm

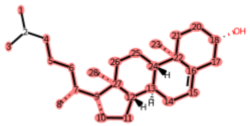

Rank: 426 MW: 386.65
LTS0102304
Score: 0.96 (26/27 C)
Deviation : 7.74 ppm

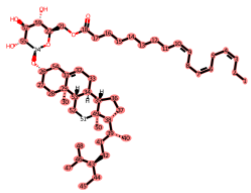

Rank: 427 MW: 837.26
LTS0194229
Score: 0.96 (51/53 C)
Deviation : 10.84 ppm

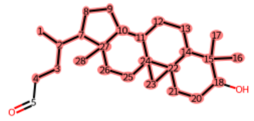

Rank: 428 MW: 386.61
LTS0046952
Score: 0.96 (25/26 C)
Deviation : 3.61 ppm

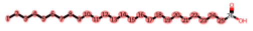

Rank: 429 MW: 396.69
LTS0240902
Score: 0.96 (25/26 C)
Deviation : 7.43 ppm

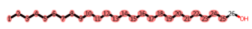

Rank: 430 MW: 382.71
LTS0140051
Score: 0.96 (25/26 C)
Deviation : 7.66 ppm

Figure S94: Dereplication analysis from MixONat, structure of dereplicated compounds: Rank **421-430**.

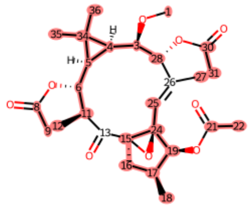

Rank: 431 MW: 506.59
LTS0100999
Score: 0.96 (25/26 C)
Deviation : 8.14 ppm

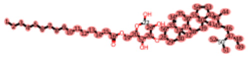

Rank: 432 MW: 815.26
LTS0150510
Score: 0.96 (49/51 C)
Deviation : 9.49 ppm

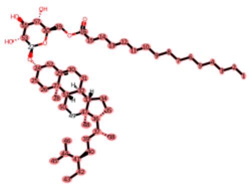

Rank: 433 MW: 815.26
LTS0071215
Score: 0.96 (49/51 C)
Deviation : 10.4 ppm

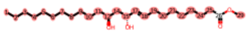

Rank: 434 MW: 412.65
LTS0004027
Score: 0.96 (24/25 C)
Deviation : 3.29 ppm

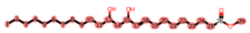

Rank: 435 MW: 412.65
LTS0133724
Score: 0.96 (24/25 C)
Deviation : 3.38 ppm

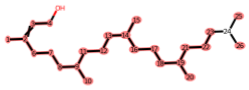

Rank: 436 MW: 366.66
LTS0014537
Score: 0.96 (24/25 C)
Deviation : 3.67 ppm

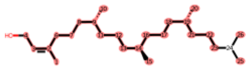

Rank: 437 MW: 366.66
LTS0050346
Score: 0.96 (24/25 C)
Deviation : 5.42 ppm

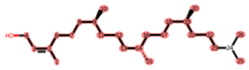

Rank: 438 MW: 366.66
LTS0214641
Score: 0.96 (24/25 C)
Deviation : 5.71 ppm

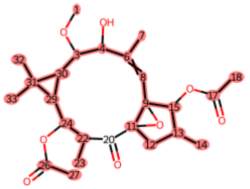

Rank: 439 MW: 464.55
LTS0208339
Score: 0.96 (24/25 C)
Deviation : 8.43 ppm

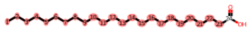

Rank: 440 MW: 368.64
LTS0107726
Score: 0.96 (23/24 C)
Deviation : 5.23 ppm

Figure S95: Dereplication analysis from MixONat, structure of dereplicated compounds: Rank **431-440**.

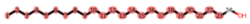

Rank: 441 MW: 354.65
LTS0198932
Score: 0.96 (23/24 C)
Deviation : 5.46 ppm

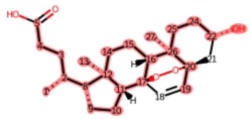

Rank: 442 MW: 404.54
LTS0246039
Score: 0.96 (23/24 C)
Deviation : 5.59 ppm

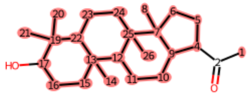

Rank: 443 MW: 360.57
LTS0122466
Score: 0.96 (23/24 C)
Deviation : 5.78 ppm

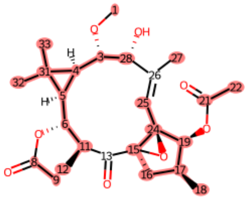

Rank: 444 MW: 464.55
LTS0052270
Score: 0.96 (23/24 C)
Deviation : 7.44 ppm

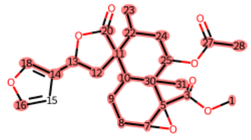

Rank: 445 MW: 432.46
LTS0053843
Score: 0.96 (22/23 C)
Deviation : 8.42 ppm

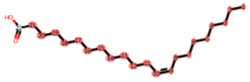

Rank: 446 MW: 338.57
LTS0252257
Score: 0.95 (21/22 C)
Deviation : 1.48 ppm

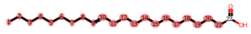

Rank: 447 MW: 338.57
LTS0129110
Score: 0.95 (21/22 C)
Deviation : 3.13 ppm

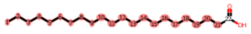

Rank: 448 MW: 340.58
LTS0058784
Score: 0.95 (21/22 C)
Deviation : 3.34 ppm

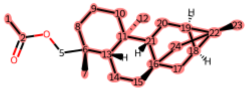

Rank: 449 MW: 330.5
LTS0185756
Score: 0.95 (21/22 C)
Deviation : 3.42 ppm

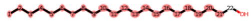

Rank: 450 MW: 326.6
LTS0250870
Score: 0.95 (21/22 C)
Deviation : 3.57 ppm

Figure S96: Dereplication analysis from MixONat, structure of dereplicated compounds: Rank **441-450**.

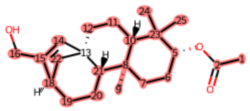

Rank: 451 MW: 346.5
LTS0163257
Score: 0.95 (21/22 C)
Deviation : 6.28 ppm

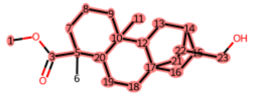

Rank: 452 MW: 334.49
LTS0103074
Score: 0.95 (20/21 C)
Deviation : 5.77 ppm

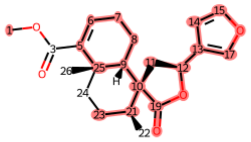

Rank: 453 MW: 358.43
LTS0222781
Score: 0.95 (20/21 C)
Deviation : 5.93 ppm

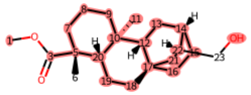

Rank: 454 MW: 334.49
LTS0113666
Score: 0.95 (20/21 C)
Deviation : 6.28 ppm

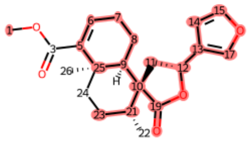

Rank: 455 MW: 358.43
LTS0002035
Score: 0.95 (20/21 C)
Deviation : 6.55 ppm

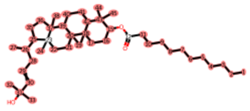

Rank: 456 MW: 625.02
LTS0069637
Score: 0.95 (40/42 C)
Deviation : 9.52 ppm

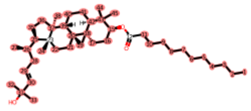

Rank: 457 MW: 625.02
LTS0060395
Score: 0.95 (40/42 C)
Deviation : 9.85 ppm

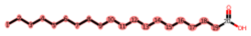

Rank: 458 MW: 312.53
LTS0171823
Score: 0.95 (19/20 C)
Deviation : 2.23 ppm

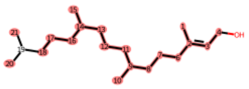

Rank: 459 MW: 296.53
LTS0031808
Score: 0.95 (19/20 C)
Deviation : 2.28 ppm

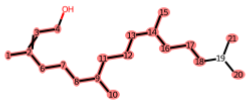

Rank: 460 MW: 296.53
LTS0056933
Score: 0.95 (19/20 C)
Deviation : 2.28 ppm

Figure S97: Dereplication analysis from MixONat, structure of dereplicated compounds: Rank **451-460**.

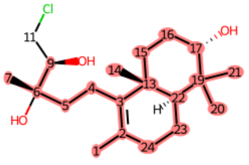

Rank: 461 MW: 358.94
LTS0152912
Score: 0.95 (19/20 C)
Deviation : 2.37 ppm

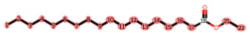

Rank: 462 MW: 312.53
LTS0161491
Score: 0.95 (19/20 C)
Deviation : 2.44 ppm

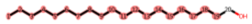

Rank: 463 MW: 298.55
LTS0230409
Score: 0.95 (19/20 C)
Deviation : 2.46 ppm

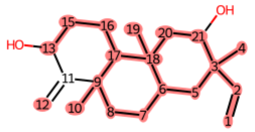

Rank: 464 MW: 304.47
LTS0078555
Score: 0.95 (19/20 C)
Deviation : 2.47 ppm

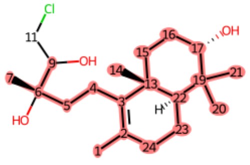

Rank: 465 MW: 358.94
LTS0143276
Score: 0.95 (19/20 C)
Deviation : 2.48 ppm

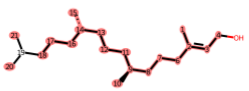

Rank: 466 MW: 296.53
LTS0207261
Score: 0.95 (19/20 C)
Deviation : 3.28 ppm

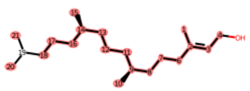

Rank: 467 MW: 296.53
LTS0196846
Score: 0.95 (19/20 C)
Deviation : 3.31 ppm

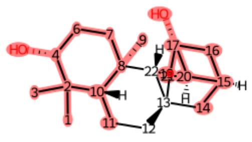

Rank: 468 MW: 304.47
LTS0086509
Score: 0.95 (19/20 C)
Deviation : 3.43 ppm

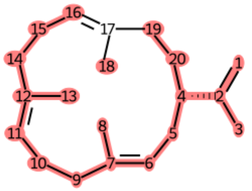

Rank: 469 MW: 272.47
LTS0237318
Score: 0.95 (19/20 C)
Deviation : 3.61 ppm

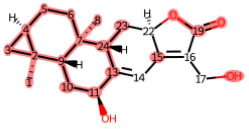

Rank: 470 MW: 330.42
LTS0183580
Score: 0.95 (19/20 C)
Deviation : 3.65 ppm

Figure S98: Dereplication analysis from MixONat, structure of dereplicated compounds: Rank **461-470**.

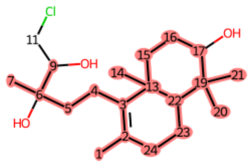

Rank: 471 MW: 358.94
LTS0223974
Score: 0.95 (19/20 C)
Deviation : 3.66 ppm

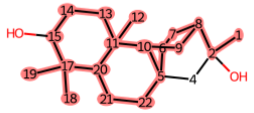

Rank: 472 MW: 306.48
LTS0243529
Score: 0.95 (19/20 C)
Deviation : 3.74 ppm

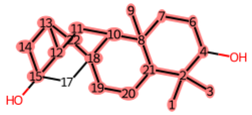

Rank: 473 MW: 304.47
LTS0150214
Score: 0.95 (19/20 C)
Deviation : 3.81 ppm

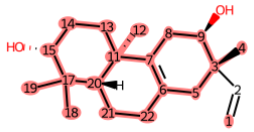

Rank: 474 MW: 304.47
LTS0174743
Score: 0.95 (19/20 C)
Deviation : 3.82 ppm

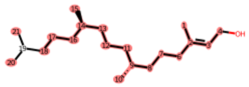

Rank: 475 MW: 296.53
LTS0096073
Score: 0.95 (19/20 C)
Deviation : 3.91 ppm

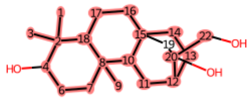

Rank: 476 MW: 322.48
LTS0176011
Score: 0.95 (19/20 C)
Deviation : 3.91 ppm

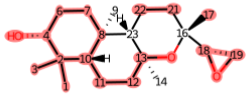

Rank: 477 MW: 322.48
LTS0039344
Score: 0.95 (19/20 C)
Deviation : 3.95 ppm

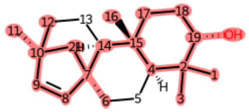

Rank: 478 MW: 288.47
LTS0108079
Score: 0.95 (19/20 C)
Deviation : 4.01 ppm

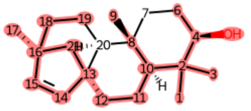

Rank: 479 MW: 288.47
LTS0077339
Score: 0.95 (19/20 C)
Deviation : 4.01 ppm

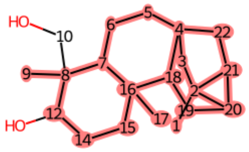

Rank: 480 MW: 304.47
LTS0035574
Score: 0.95 (19/20 C)
Deviation : 4.02 ppm

Figure S99: Dereplication analysis from MixONat, structure of dereplicated compounds: Rank **471-480**.

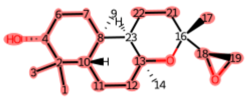

Rank: 481 MW: 322.48
LTS0253706
Score: 0.95 (19/20 C)
Deviation : 4.03 ppm

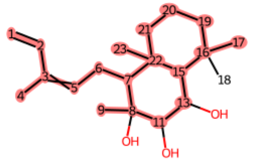

Rank: 482 MW: 322.48
LTS0272012
Score: 0.95 (19/20 C)
Deviation : 4.07 ppm

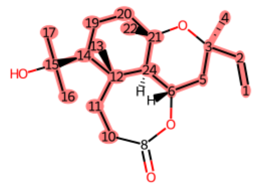

Rank: 483 MW: 336.47
LTS0133909
Score: 0.95 (19/20 C)
Deviation : 4.07 ppm

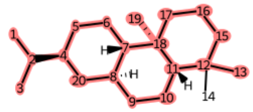

Rank: 484 MW: 276.5
LTS0062446
Score: 0.95 (19/20 C)
Deviation : 4.12 ppm

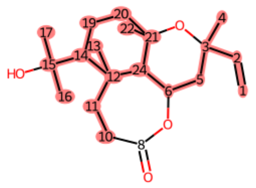

Rank: 485 MW: 336.47
LTS0131385
Score: 0.95 (19/20 C)
Deviation : 4.15 ppm

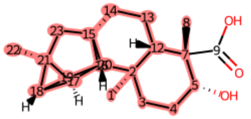

Rank: 486 MW: 318.45
LTS0035477
Score: 0.95 (19/20 C)
Deviation : 4.26 ppm

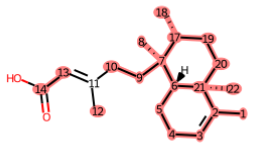

Rank: 487 MW: 304.47
LTS0253107
Score: 0.95 (19/20 C)
Deviation : 4.38 ppm

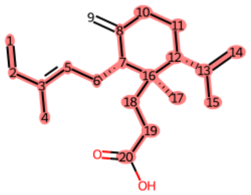

Rank: 488 MW: 302.45
LTS0254647
Score: 0.95 (19/20 C)
Deviation : 4.42 ppm

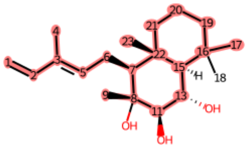

Rank: 489 MW: 322.48
LTS0164561
Score: 0.95 (19/20 C)
Deviation : 4.44 ppm

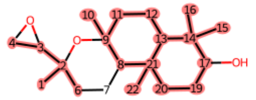

Rank: 490 MW: 322.48
LTS0200522
Score: 0.95 (19/20 C)
Deviation : 4.51 ppm

Figure S100: Dereplication analysis from MixONat, structure of dereplicated compounds: Rank **481-490**.

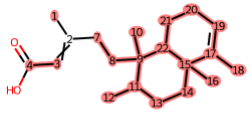

Rank: 491 MW: 304.47
LTS0201482
Score: 0.95 (19/20 C)
Deviation : 4.6 ppm

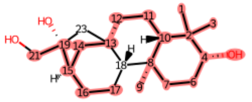

Rank: 492 MW: 322.48
LTS0068200
Score: 0.95 (19/20 C)
Deviation : 4.76 ppm

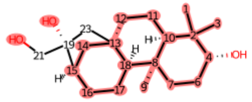

Rank: 493 MW: 322.48
LTS0087822
Score: 0.95 (19/20 C)
Deviation : 4.83 ppm

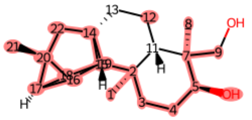

Rank: 494 MW: 304.47
LTS0153489
Score: 0.95 (19/20 C)
Deviation : 4.84 ppm

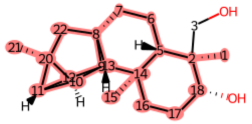

Rank: 495 MW: 304.47
LTS0120842
Score: 0.95 (19/20 C)
Deviation : 4.84 ppm

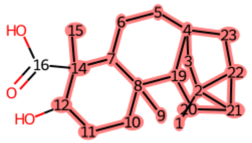

Rank: 496 MW: 318.45
LTS0208314
Score: 0.95 (19/20 C)
Deviation : 4.92 ppm

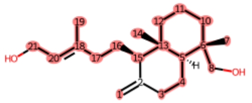

Rank: 497 MW: 306.48
LTS0068647
Score: 0.95 (19/20 C)
Deviation : 5.0 ppm

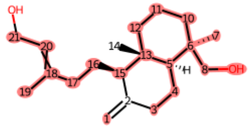

Rank: 498 MW: 306.48
LTS0177350
Score: 0.95 (19/20 C)
Deviation : 5.04 ppm

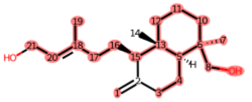

Rank: 499 MW: 306.48
LTS0261076
Score: 0.95 (19/20 C)
Deviation : 5.04 ppm

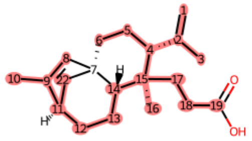

Rank: 500 MW: 302.45
LTS0271882
Score: 0.95 (19/20 C)
Deviation : 5.07 ppm

Figure S101: Dereplication analysis from MixONat, structure of dereplicated compounds: Rank **491-500**.

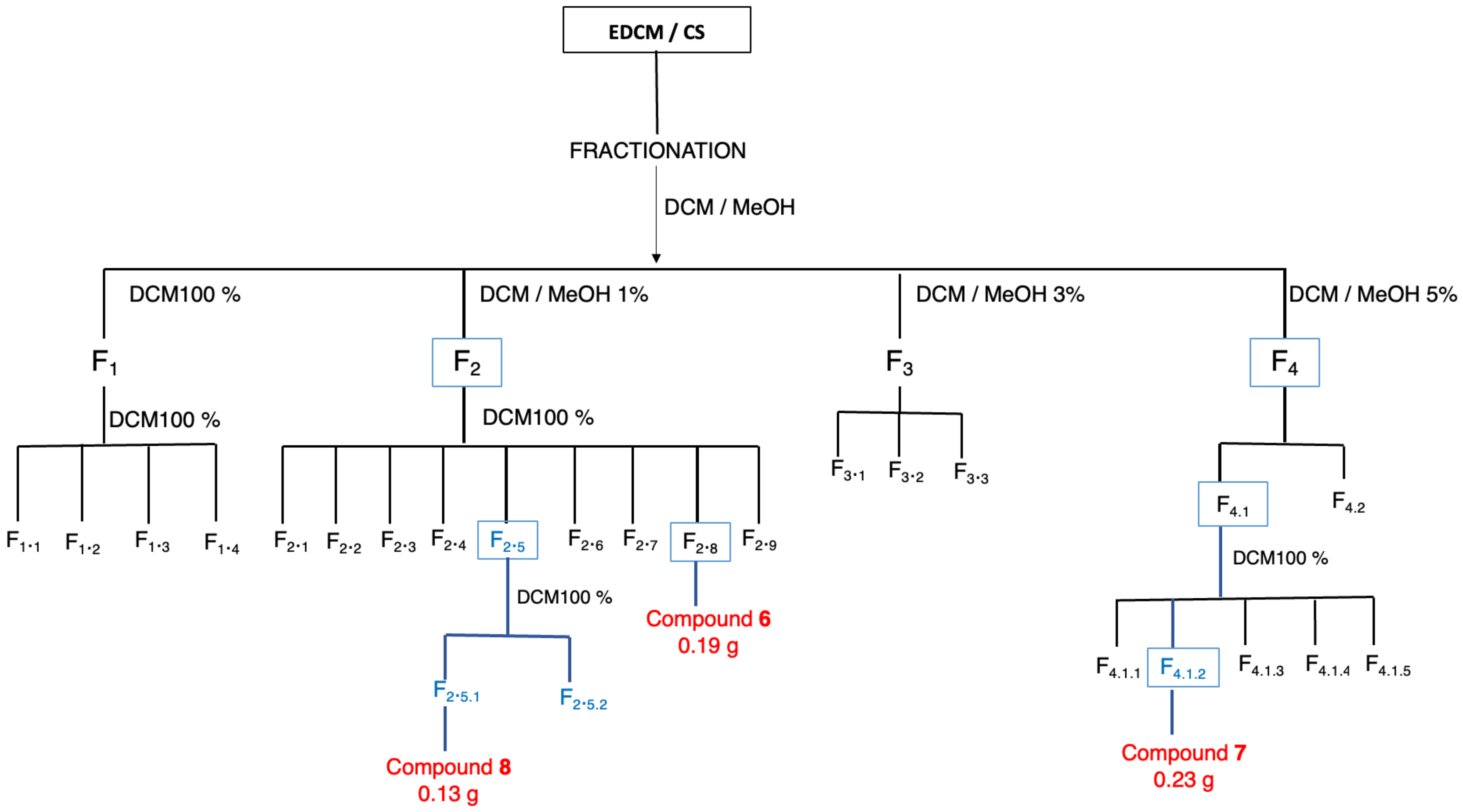

Figure S102. Scheme describes the purification of the dichloromethane extract of *Croton sylvaticus* (EDCM/CS).

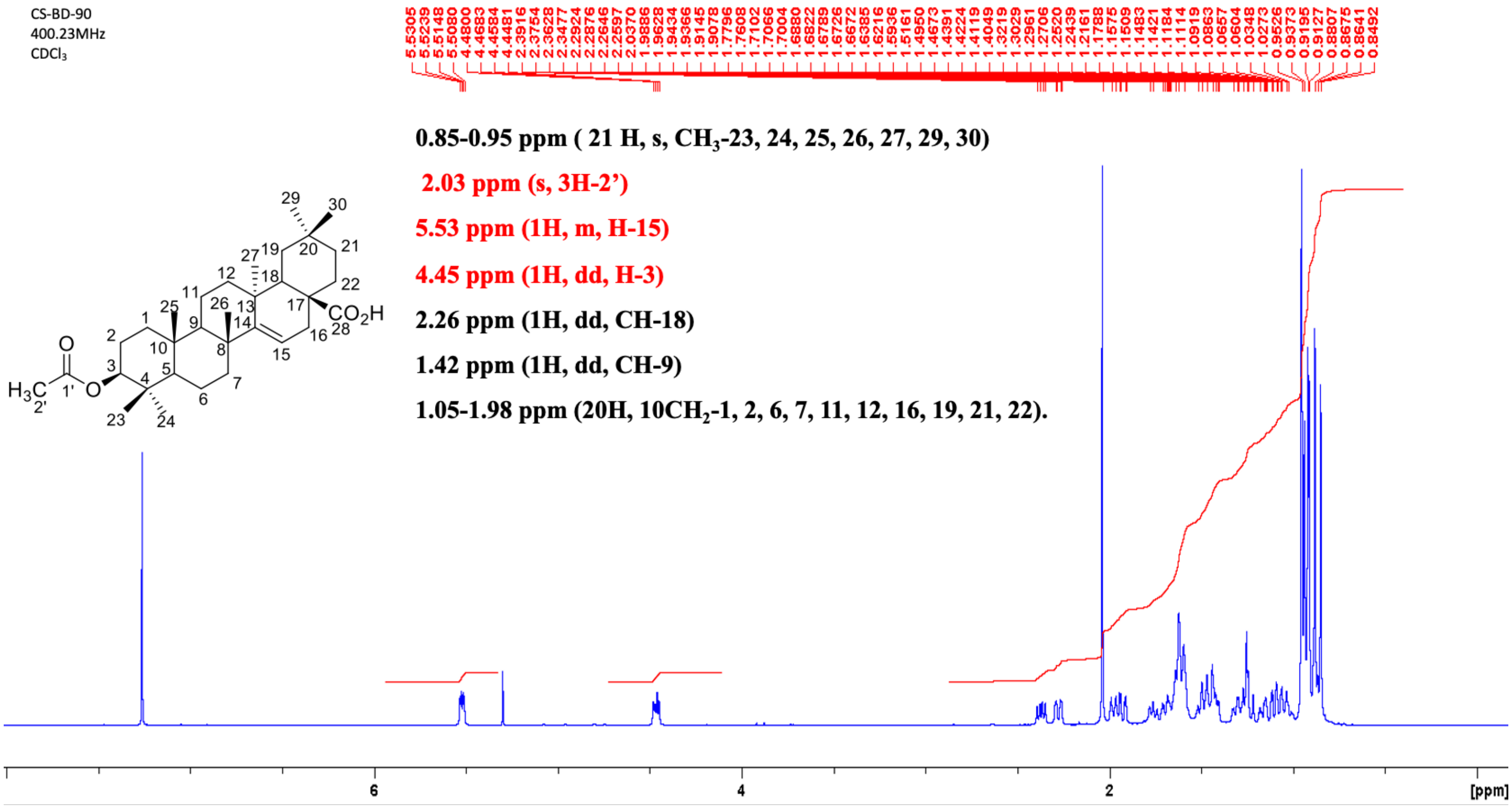

Figure S103. ^{1}H NMR for compound **6** (DCM/CS) in $CDCl_3$

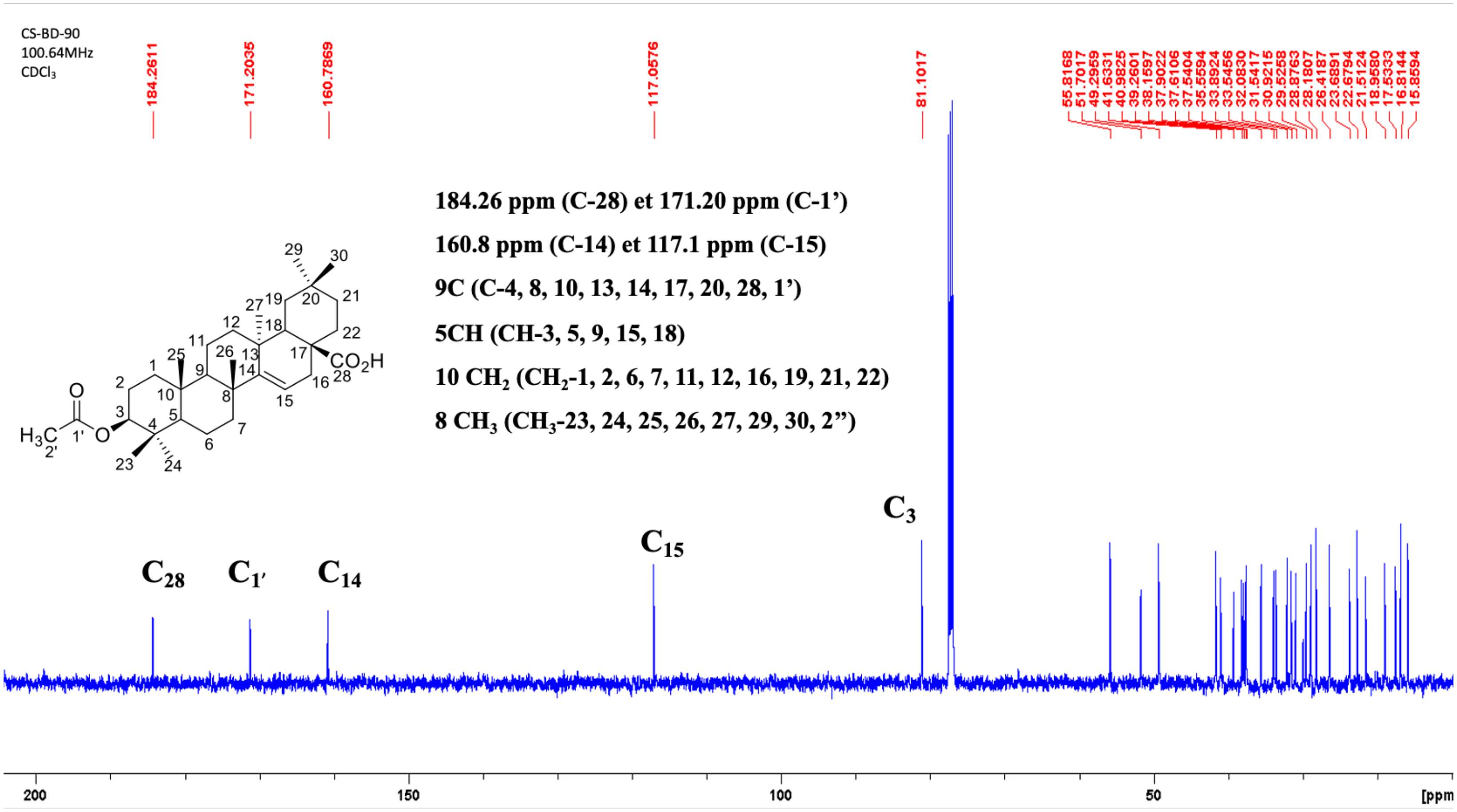

Figure S104. ^{13}C NMR for compound **6** (DCM/CS) in $CDCl_3$

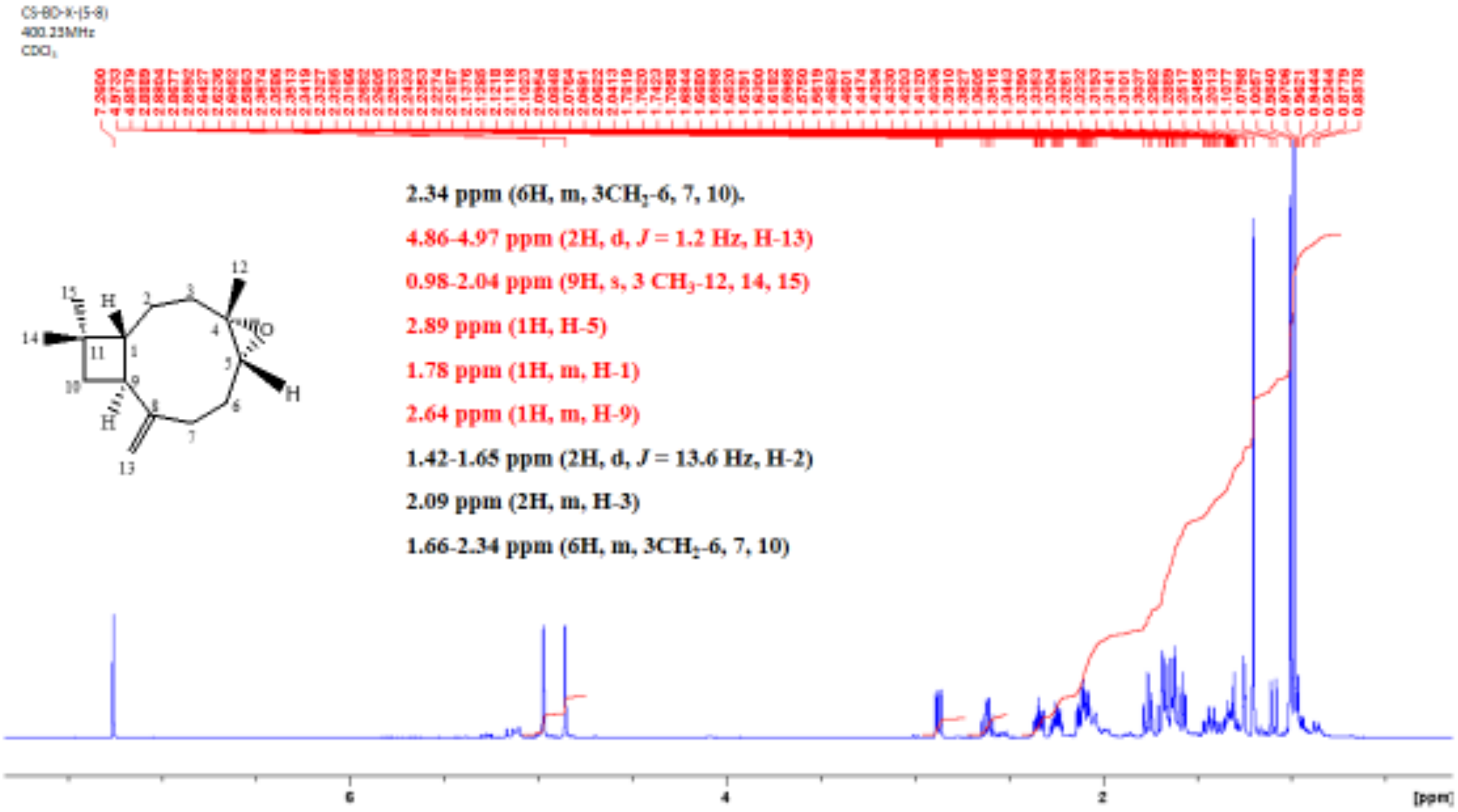

Figure S105. ^{1}H NMR for compound **7** (DCM/CS) in $CDCl_3$

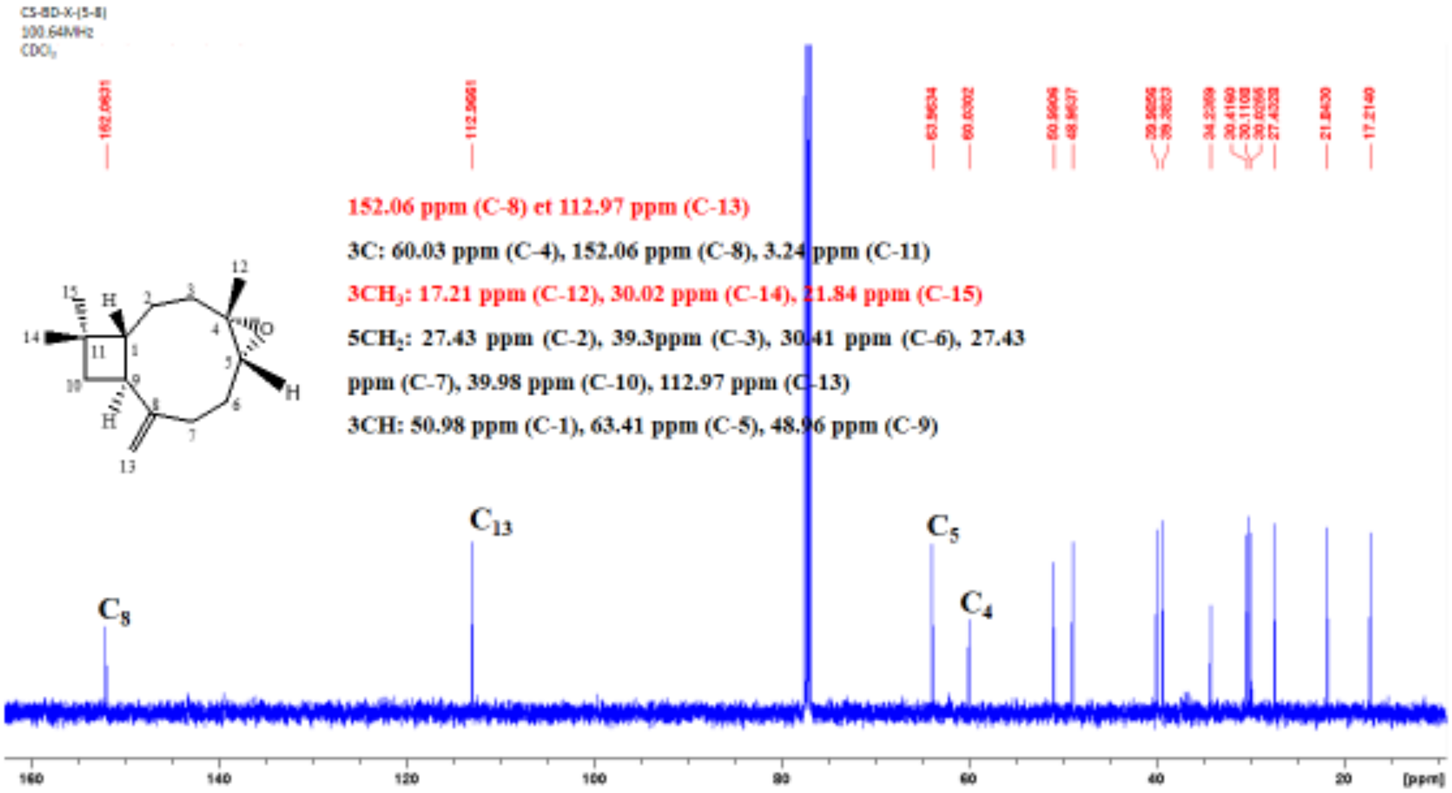

Figure S106. ^{13}C NMR for compound **7** (DCM/CS) in $CDCl_3$

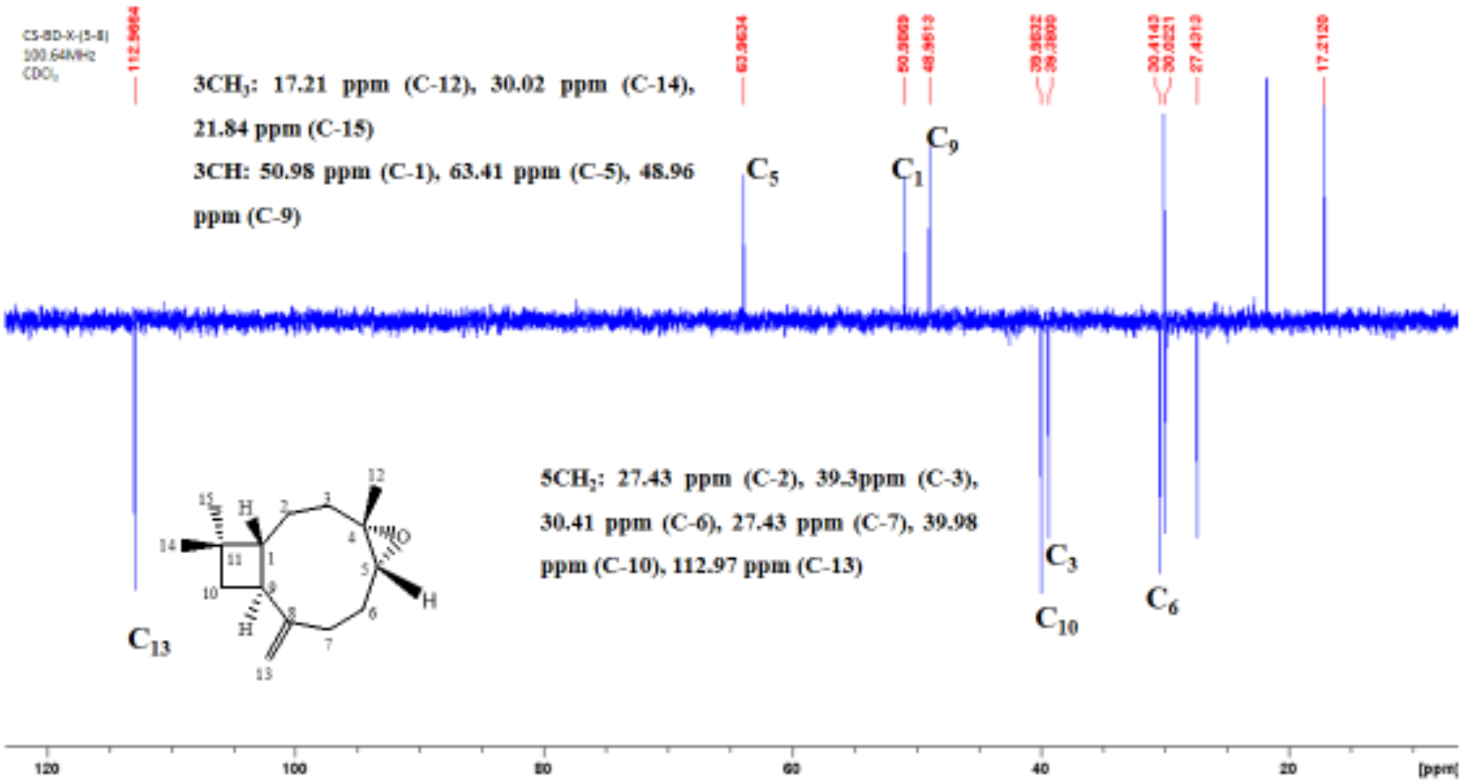

Figure S107. DEPT for compound **7** (DCM/CS) in $CDCl_3$

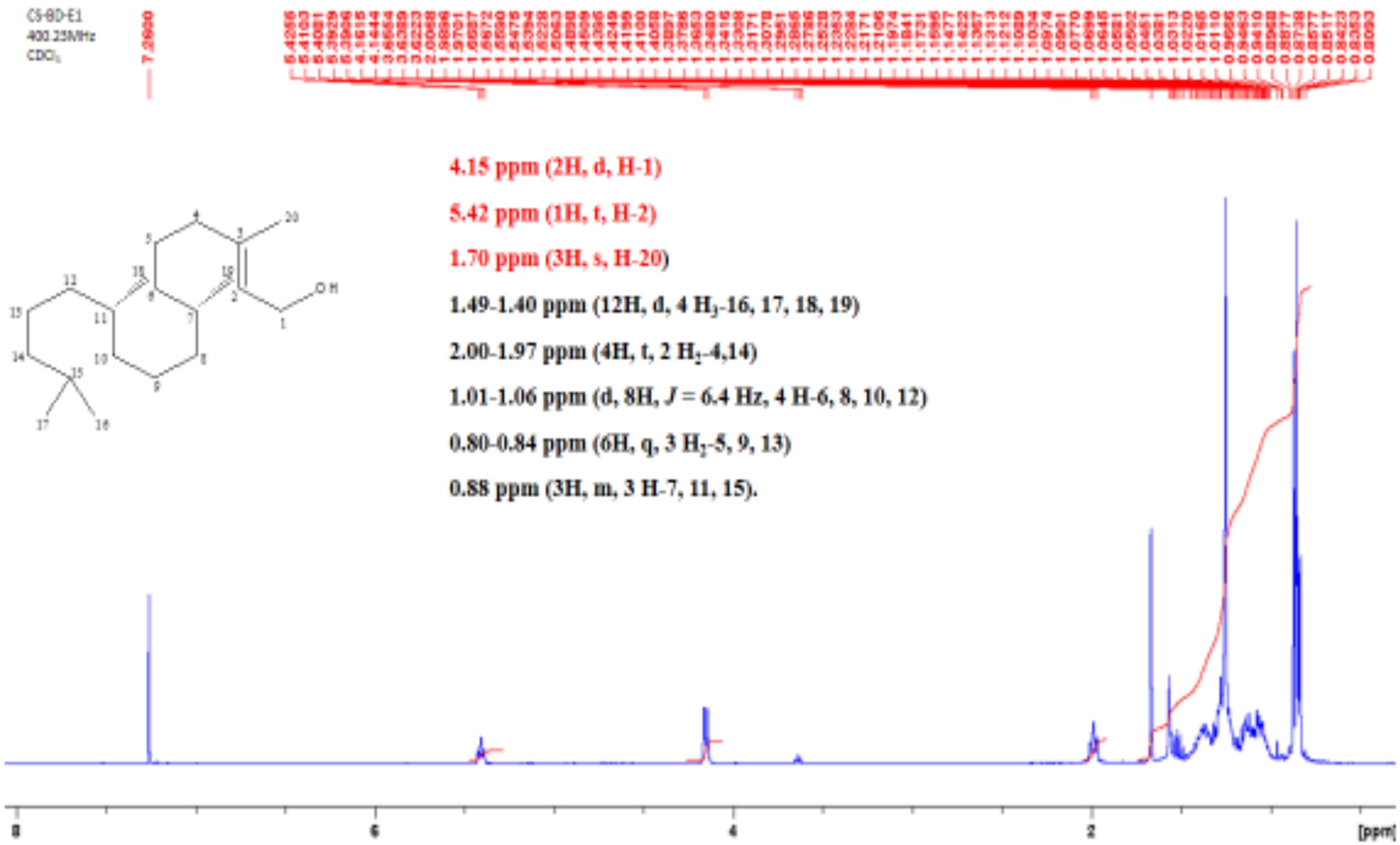

Figure S108. ^{1}H NMR for compound **8** (DCM/CS) in CDCl$_3$

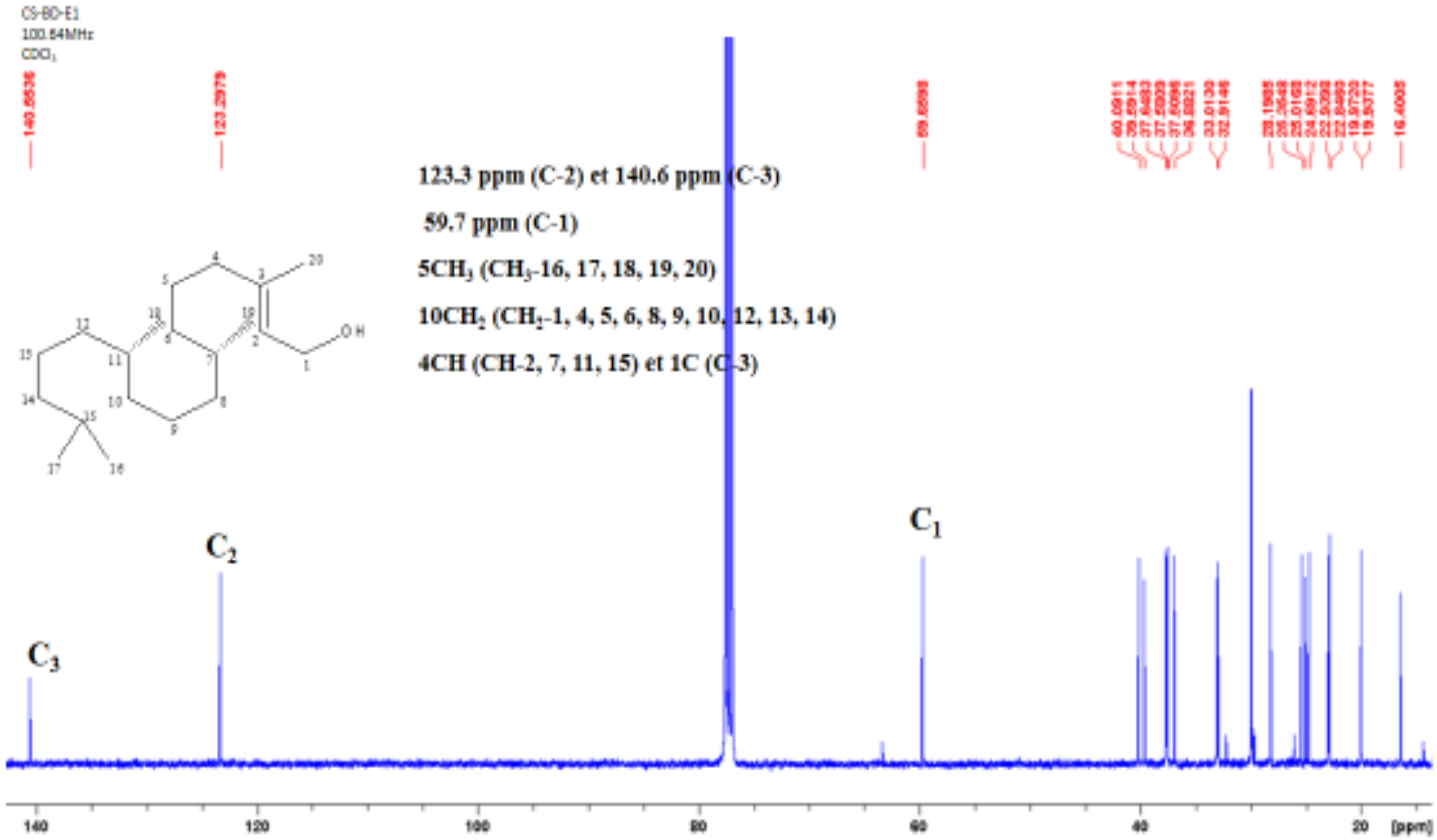

Figure S109. ^{13}C NMR for compound **8** (DCM/CS) in $CDCl_3$

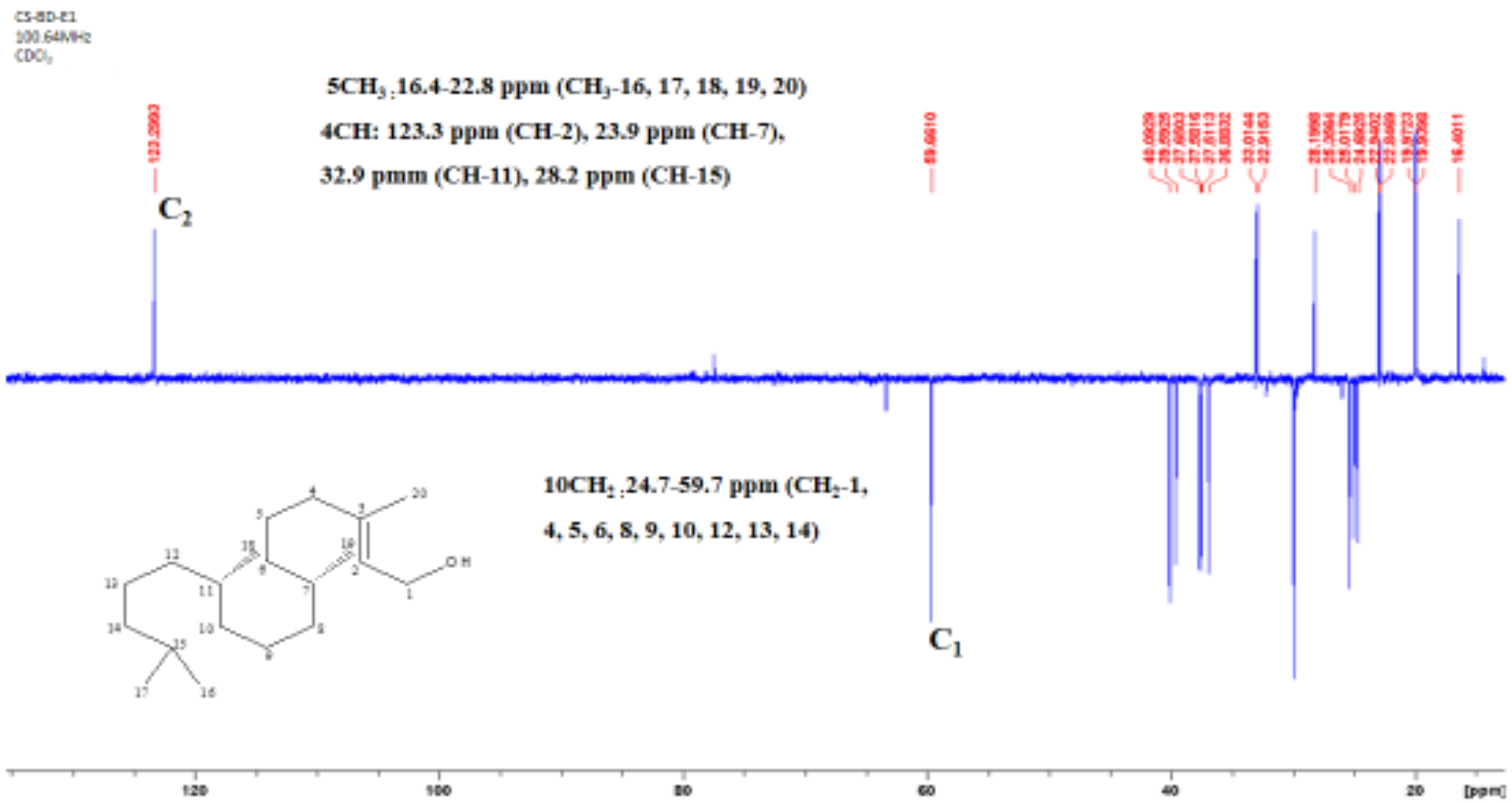

Figure S110. DEPT for compound **8** (DCM/CS) in $CDCl_3$

Table S5. ^{13}C-NMR values for compound acetyl aleuritolic acid **6** (CS-BD-90)

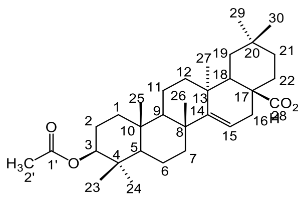

Carbon	δ_C	δ_C Ref.	Carbon	δ_C	δ_C Ref.
1	37,6 (CH_2)	37,3	16	31,5 (CH_2)	31,3
2	23, 6 (CH_2)	23,4	17	51,7 (C)	51,5
3	81,1 (CH)	80,9	18	41,6 (CH)	41,3
4	37,6 (C)	37,7	19	35,6 (CH_2)	35,3
5	55,8 (CH)	55,5	20	29,5 (C)	29,3
6	18,9 (CH_2)	18,7	21	33,9 (CH_2)	33,6
7	40,9 (CH_2)	40,7	22	30,9 (CH_2)	30,7
8	39,3 (C)	39,0	23	28,2 (CH_3)	27,9
9	49,2 (CH)	49,0	24	16,8 (CH_3)	16,6
10	37,9 (C)	37,9	25	15,8 (CH_3)	15,6
11	17,3 (CH_2)	17,5	26	26,4 (CH_3)	26,2
12	33,5 (CH_2)	33,3	27	22,7 (CH_3)	22,5
13	38,3 (C)	37,3	28	184,3 (C)	184,3
14	160,8 (C)	160,5	29	32,0 (CH_3)	31,8
15	117,0 (CH)	116,8	30	28,8 (CH_3)	28,6
1′	171,2 (C)	171,0	2′′	21,5 (CH_3)	21,3

^{13}C-RMN 125 MHz, $CDCl_3$, Lit. Rosmawati et *al*., 2016, ^{13}C-RMN 125 MHz, $CDCl_3$

Table S6. ^{13}C values for compound Caryophyllene oxide 7 (CS-BD-X-**5**–**8**)

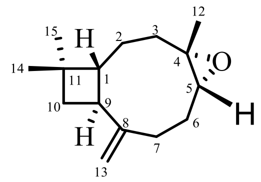

Carbon	δ_C	δ_C **Ref**.	Carbon	δ_C	δ_C **Ref**.
1	50.98 (CH)	50.80	9	48.96 (CH)	48.90
2	27.43 (CH_2)	27.40	10	39.98 (CH_2)	39.90
3	39.36 (CH_2)	39.30	11	34.24 (C)	34.20
4	60.03 (C)	60.00	12	17.21 (CH_3)	17.20
5	63.96 (CH)	63.90	13	112.97 (CH_2)	113.0
6	30.41 (CH_2)	30.40	14	30.02 (CH_3)	30.10
7	27.43 (CH_2)	29.90	15	21.84 (CH_3)	21.80
8	152.06 (C)	152.00			

^{13}C-RMN 125 MHz, $CDCl_3$, Lit. Christopher et *al*., 2007, ^{13}C-RMN 100 MHz, $CDCl_3$

Table S7. ^{13}C values for compound *Trans*-Phytol (3,7,11,15-tetramethylhexadec-2-en-1-ol) (CS-BD-E1)

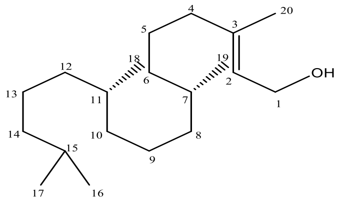

Carbon	δC	δC Ref.	Carbon	δC	δC Ref.
1	59,7 (CH_2)	59,6	11	33,0 (CH)	32,9
2	123,3 (CH)	123,2	12	36,9 (CH_2)	36,8
3	140, 6 (C)	140,4	13	24,7 (CH_2)	24,9
4	40,1 (CH_2)	40,7	14	39,6 (CH_2)	39,5
5	25,4 (CH_2)	25,3	15	28,2 (CH)	28,1
6	37,5 (CH_2)	37,4	16	22,8 (CH_3)	22,7
7	23,9 (CH)	32.7	17	22,9 (CH_3)	22,8
8	37,6 (CH_2)	38,2	18	19,9 (CH_3)	19,9
9	24,7 (CH_2)	24,6	19	19,9 (CH_3)	19,0
10	37,6 (CH_2)	37,6	20	16,4 (CH_3)	16,3

^{13}C-RMN 125 MHz, $CDCl_3$, Lit. Vladimir et *al*., 2019, ^{13}C-RMN 100 MHz, $CDCl_3$

Table S8. 15 compounds used in this study

N°	Especes	Structure	Name
1	*C. mubango*	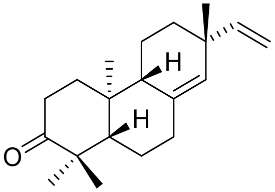	*Ent*-pimara-8(14),15-dien-3-one
2	*C. haumanianus*	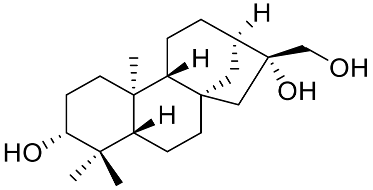	*Ent*-kaurane-3β,16β,17-triol
3	*C. haumanianus*	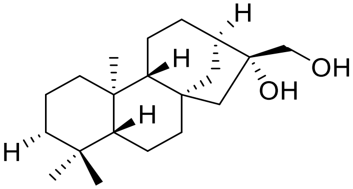	*Ent*-kaurane-16β,17-diol
4	*C. haumanianus*	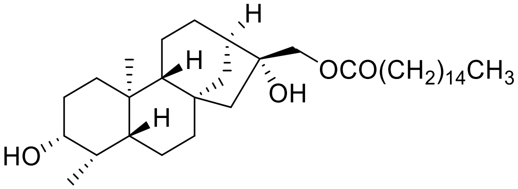	*Ent*-17-palmityloxykaurane-3β,16β-diol
5	*C. haumanianus*	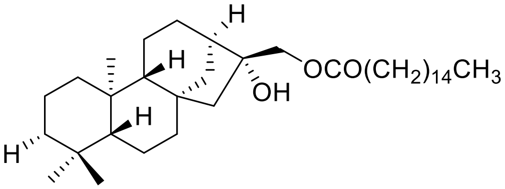	*Ent*-17-palmityloxykauran-16β-ol

6	*C. zehntneri, cajucara, Cascarilloides, urucurana*	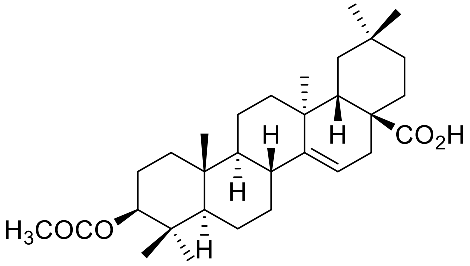	Acetyl aleuritolic acid
7	*C. zehntneri, sylvaticus*	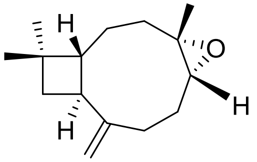	
8	*C. zambesicus, thurifer*	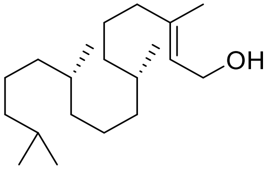	*Trans*-Phytol (3,7,11,15-tetraméthylhexadec-2-en-1-ol)
9	*C. sylvaticus*	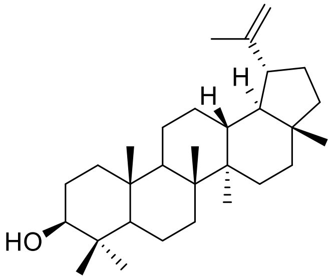	Lup-20(29)-en-3β-ol
10	*C. sylvaticus*	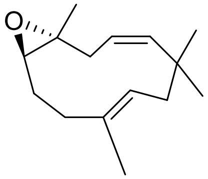	α-humulene-1,2-epoxide
11	*C. haumanianus*	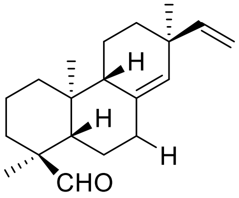	*Ent*-isopimara-8(14),15-dien-18-al

12	*C. haumanianus*	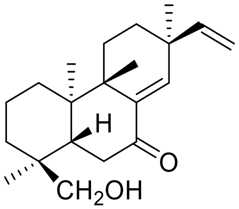	*Ent*-18-hydroxyisopimara-8(14),15-dien-7-one
13	*C. haumanianus*	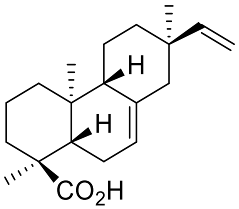	*Ent*-isopimara-7,15-dien-18-oic acid
14	*C. haumanianus*	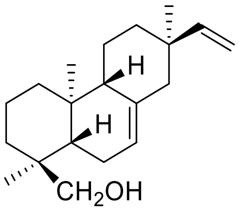	*Ent*-isopimara-7,15-dien-18-ol
15	*C. sylvaticus*	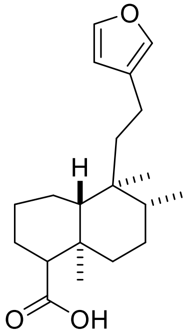	Hardwickiic acid

Table 9 Binding Affinity and Physicochemical Properties of Compounds for Protein Targets 1E3G and 3KCX

Protein	Compound	Ligand	Protein substate	H-Bound Distance	H-Bound angle
1E3G	1.	C=O	H-N(GLN711)	2.31	171
1E3G	12.	C=O	H-N(GLN711)	1.97	155
3KCX	1.	C=O	H-N(ARG238)	1.98	148
		C=O	H-N(ARG238)	2.57	98

Docking studies of terpenoids 6–8: Comparison with Ligand 6, Ligand 7 and Ligand 8

1E3G : Ligand 6 is stabilized predominantly by van der Waals interactions, which suggests a hydrophobic binding site. The presence of **Trp (tryptophan), Val (valine), Met (methionine), and Leu (leucine)** residues indicates a nonpolar environment in the pocket. The binding mode suggests that compound **6** fits well into the binding pocket, potentially making it a good inhibitor or modulator of the 1E3G protein.

The ligand (Ligand 7) interacted with **hydrophobic residues** primarily through **van der Waals and alkyl interactions**. Key interacting residues included **Leu-701, Leu-704, Leu-707, and Leu-873**, indicating a hydrophobic pocket. Additional contacts involved **Met-742, Met-745, Met-780, Met-787, and Met-895**, which further confirmed a nonpolar binding environment. One **conventional hydrogen bond** was formed, possibly increasing the binding affinity. The ball-and-stick model displayed **ligand 7** inside the binding pocket.

The **purple dashed lines represent the alkyl interactions**, reinforcing the **hydrophobic stabilization** mechanism. The ligand appeared to be **well-fitted within the pocket**, suggesting a strong interaction profile.

Ligand 8 interacted extensively with hydrophobic residues, forming van der Waals and alkyl interactions. Key interacting residues: Hydrophobic residues: Leu-701, Leu-704, Leu-707, Leu-873, Leu-880, Methionine residues: Met-742, Met-745, Met-749, Met-780, Met-787, Met-895

Aromatic residues (π-alkyl interactions): Phe-764, Phe-876, Trp-741 Polar contacts: Gln-711, Arg-752, Asn-705, and Thr-87. The presence of π-alkyl interactions suggests strong hydrophobic and π-stacking stabilization.

The **ball-and-stick model** showed that **ligand 8** was within the binding pocket. **The purple dashed lines** highlight **the π-alkyl and alkyl interactions**, confirming the **hydrophobic nature** of the pocket. The ligand appears **well-positioned and deeply buried**, suggesting strong binding stability.

3KCX: Compound **6** had a polycyclic aromatic structure, forming diverse interactions, including strong van der Waals forces and a notable π-sigma interaction with HIS A:199. Its complex and rigid structure likely result in the strongest binding affinity, making it highly stable in the hydrophobic and aromatic-rich pocket of 3KCX. Compound **7** is structurally simpler, relying primarily on van der Waals interactions with residues such as PHE A:177 and LEU A:160. While it fits into the hydrophobic binding pocket, the lack of significant polar or π-related interactions may reduce its binding strength compared to that of compound **6**. Compound **8** features a long hydrophobic chain and was optimized for fitting into an elongated hydrophobic pocket. It forms robust van der Waals and π-alkyl interactions with residues, such as TYR A:84 and TRP A:258, supported by a π-sigma interaction with HIS A:171. Its linear structure makes it less versatile than compound 6 but effective in hydrophobic environments.

In summary, all three compounds relied on hydrophobic interactions with the 3KCX receptor. Compound **6** exhibited the most diverse and potentially strongest binding due to its aromatic features, while compounds **7** and **8** showed simpler, hydrophobic-driven binding, with compound 8 being better suited for elongated hydrophobic regions.

Table S10: Comparison with Ligand 6, Ligand 7 and Ligand 8

Feature	Ligand 6	Ligand 7	Ligand 8
Hydrophobic Interactions	Moderate	Strong	Strongest
π-Interactions (π-alkyl, π-π stacking)	Few	Some	More frequent
Hydrogen Bonds	none	At least one	none
Binding Pocket Fit	Good	Better	Deepest and most buried
Overall Binding Stability	Moderate	High	Very High

A comparative analysis of Compounds 1, 2, 7, and 12 against both androgen receptor (HAR, PDB ID: 1E3G) and hypoxia-inducible factor-1 alpha (HIF-1α, PDB ID: 3KCX)

A comparative analysis of Compounds 1, 2, 7, and 12 against both androgen receptor (HAR, PDB ID: 1E3G) and hypoxia-inducible factor-1 alpha (HIF-1α, PDB ID: 3KCX) highlights their potential as promising multitarget anticancer agents. All four molecules demonstrated favorable binding affinities toward the two targets, with binding free energy differences (ΔG) equal to or below 1.0 kcal/mol, indicative of balanced interactions. Compound 1 exhibited the strongest binding within the group, with docking scores of –7.7 kcal/mol for 1E3G and –8.4 kcal/mol for 3KCX (ΔG = 0.7 kcal/mol). Compound 12 followed closely with –7.4 and –8.4 kcal/mol for 1E3G and 3KCX, respectively (ΔG = 1.0 kcal/mol). Compound 2 showed similar values (–7.3 and –8.3 kcal/mol), confirming its favorable affinity for both proteins. Notably, Compound 7 yielded identical binding energies for both targets (–7.2 kcal/mol), resulting in a ΔG of 0.0 kcal/mol, suggesting a particularly well-balanced and receptor-compatible binding mode.

Molecular interaction visualizations support these findings. Compound 1 forms multiple stabilizing interactions, including hydrogen bonds and hydrophobic contacts with key residues in the ligand-binding domains of both receptors. In 1E3G, π–π stacking and hydrogen bonds contribute to its high affinity, while in 3KCX, polar interactions with residues such as ASN, TYR, or GLU further stabilize the complex. Compound 2 displays similar interaction patterns, occupying the binding cavities effectively and forming polar and nonpolar contacts that reinforce its binding. Compound 12 also forms dual hydrogen bonds along with van der Waals interactions, contributing to its strong affinity, particularly in 3KCX. Compound 7, despite slightly lower absolute binding energies, is unique in its perfect energetic symmetry across both targets. Its binding mode is characterized by hydrophobic interactions complemented by hydrogen bonding, suggesting a versatile pharmacophore capable of adapting to both receptor environments without compromising affinity.

When benchmarked against reference ligands, all four test compounds outperformed Reference 2 (–6.5 and –6.4 kcal/mol) and matched or approached the performance of Reference 3 (–6.1 and –9.8 kcal/mol) and, in the case of Compound 1 and 12, partially approximated the binding affinity of the high-performing Reference 1 (–11.7 and –8.8 kcal/mol). The favorable and balanced interaction profiles observed for Compounds 1, 2, 7, and 12 across two distinct cancer-related targets suggest their potential as dual inhibitors. These findings justify further in vitro and in vivo investigation, as well as structural optimization to enhance specificity, bioavailability, and therapeutic efficacy in the context of multitarget cancer drug design.

Table S11: ADMET predictions

Index		1	2	3	4	5	6	7	8	9	10	11	12	13	14	15
Absorption	Water solubility	-6.13	-3.766	-4.713	-5.896	-5.588	-4.351	-4.357	-7.546	-6.011	-4.152	-6.148	-5.129	-4.303	-5.787	-4.193
	Caco2 permeability	1.532	1.353	1.684	0.322	1.204	0.955	1.598	1.52	1.271	1.516	1.239	1.538	1.19	1.215	1.469
	Intestinal absorption (human)	96.536	93.219	96.006	88.34	91.127	100	95.989	91.162	100	95.492	99.076	97.627	94.111	95.02	96.518
	Skin Permeability	-2.249	-3.325	-2.946	-2.708	-2.726	-2.729	-3.053	-2.569	-2.753	-2.986	-2.392	-2.977	-2.587	-2.594	-2.774
	P-glycoprotein substrate	No	Yes	Yes	Yes	Yes	No	No	No	No	Yes	No	No	No	No	Yes
	P-glycoprotein I inhibitor	Yes	No	Yes	Yes	No	No	No	No	Yes	No	Yes	Yes	No	No	No
	P-glycoprotein II inhibitor	No	No	No	Yes	Yes	Yes	No	Yes	Yes	No	No	No	No	No	No
Distribution	VDss (human)	0.672	-0.223	-0.178	-0.803	-0.785	-0.985	0.603	0.478	0.068	0.454	0.49	0.392	-0.543	0.315	-0.435
	Fraction unbound (human)	0.081	0.147	0.001	0	0	0	0.324	0	0	0.409	0	0.064	0.047	0.004	0.001
	BBB permeability	0.645	0.092	0.103	-0.542	-0.522	-0.133	0.651	0.811	0.762	0.667	0.704	-0.099	0.137	0.669	-0.217
	CNS permeability	-2.205	-2.05	-0.775	-2.07	-1	-1.004	-2.533	-1.566	-1.867	-2.914	-1.869	-2.305	-1.743	-1.653	-1.635
Metabolism	CYP2D6 substrate	No	No	No	No	No	No	No	No	No	No	No	No	No	No	No
	CYP3A4 substrate	Yes	Yes	Yes	Yes	Yes	Yes	No	Yes	Yes	No	Yes	Yes	Yes	Yes	Yes
	CYP1A2 inhibitior	No	No	No	No	No	No	No	Yes	No	No	No	No	No	No	No
	CYP2C19 inhibitior	Yes	No	No	No	No	No	Yes	No	No	No	No	No	No	No	No
	CYP2C9 inhibitior	No	No	No	No	No	No	Yes	No	No	No	No	No	Yes	No	No
	CYP2D6 inhibitior	No	No	No	No	No	No	No	No	No	No	No	No	No	No	No
	CYP3A4 inhibitior	No	No	No	No	No	No	No	No	No	No	No	No	No	No	No
Excretion	Total Clearance	0.757	0.738	0.544	0.862	0.668	-0.121	0.905	1.686	0.153	1.065	0.981	0.753	0.717	0.816	1.014
	Renal OCT2 substrate	No	No	No	No	No	No	No	No	No	No	No	No	No	No	No
Toxicity	AMES toxicity	No	No	No	No	No	No	No	No	No	No	No	No	No	No	No
	Max. tolerated dose (human)	-0.265	-0.442	-0.607	-0.603	-0.309	0.839	0.197	0.133	-0.067	0.393	-0.322	-0.462	-0.64	-1.084	-1.031
	hERG I inhibitor	No	No	No	No	No	No	No	No	No	No	No	No	No	No	No
	hERG II inhibitor	Yes	No	No	Yes	Yes	No	No	Yes	Yes	No	Yes	No	No	Yes	No
	Oral Rat Acute Toxicity (LD50)	1.531	2.538	3.132	3.458	3.41	2.896	1.551	1.603	2.616	1.583	1.777	1.978	2.179	1.972	2.797

	Oral Rat Chronic Toxicity (LOAEL)	1.078	1.627	2.034	2.467	2.915	1.796	1.206	1.043	0.967	1.169	1.098	2.086	2.301	1.69	2.161
	Hepatotoxicity	No	No	No	No	No	No	No	No	No	No	Yes	No	Yes	Yes	No
	Skin Sensitisation	No	No	No	No	No	No	Yes	Yes	No	Yes	Yes	No	No	Yes	No
	T.Pyriformis toxicity	1.711	0.529	0.613	0.296	0.288	0.286	1.073	1.903	0.321	1.075	1.63	1.479	0.331	1.567	0.785
	Minnow toxicity	-0.176	1.367	0.838	-1.88	-2.934	-2.482	1.049	-1.59	-2.355	1.143	-0.601	0.074	-0.375	-0.346	-0.892

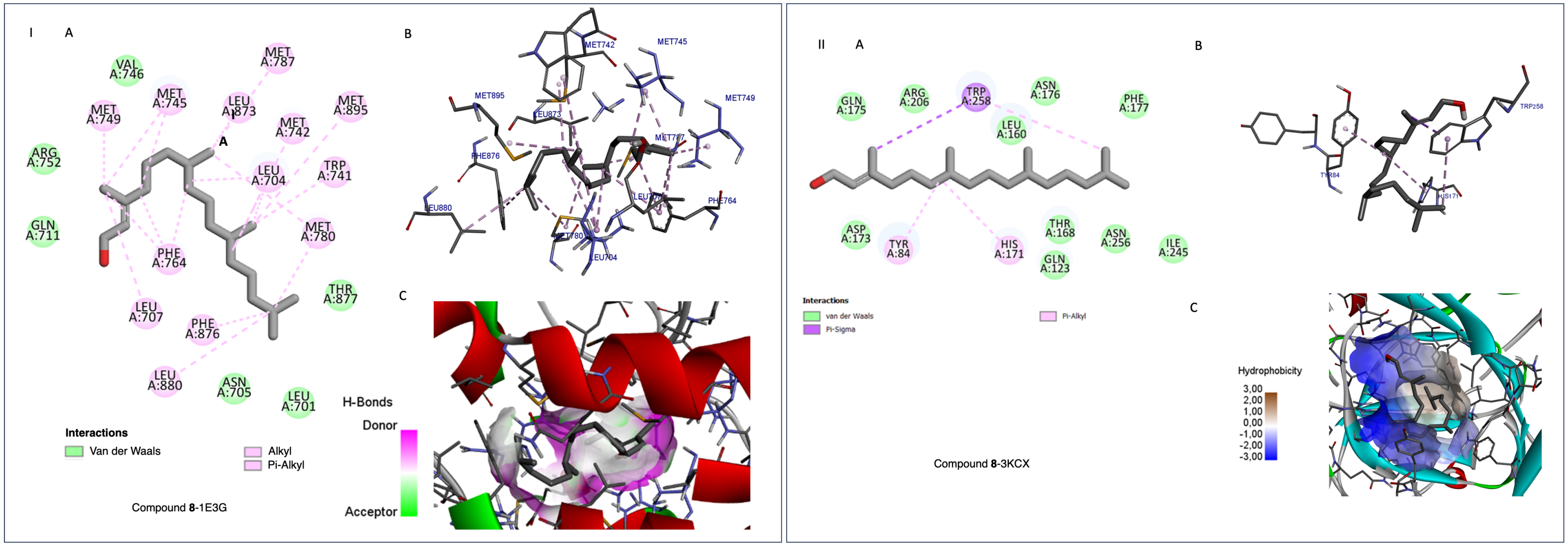

Figure S111. Compound **8**-1E3G and-3KCX: 2D and 3D image of the interaction between compound **8** with 1E3G and-3KCX receptor.

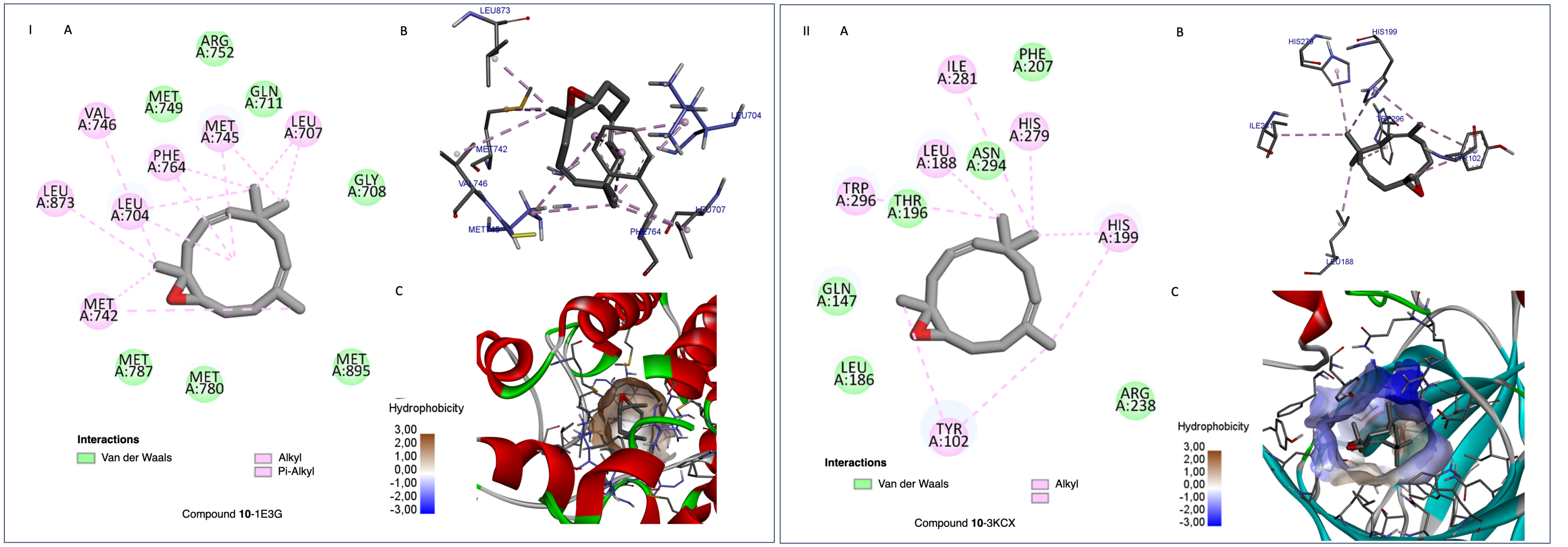

Figure S112. Compound **10**-1E3G and-3KCX: 2D and 3D image of the interaction between compound **10** with 1E3G and-3KCX receptor.

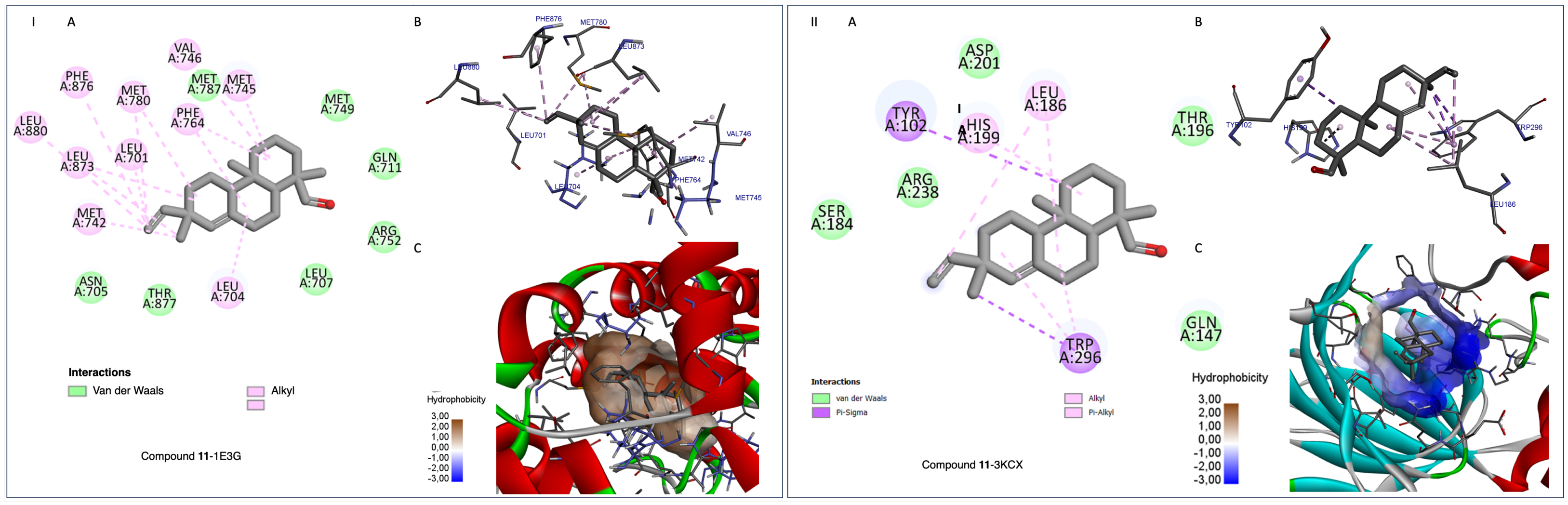

Figure S113. Compound **11**-1E3G and-3KCX: 2D and 3D image of the interaction between compound **11** with 1E3G and-3KCX receptor.

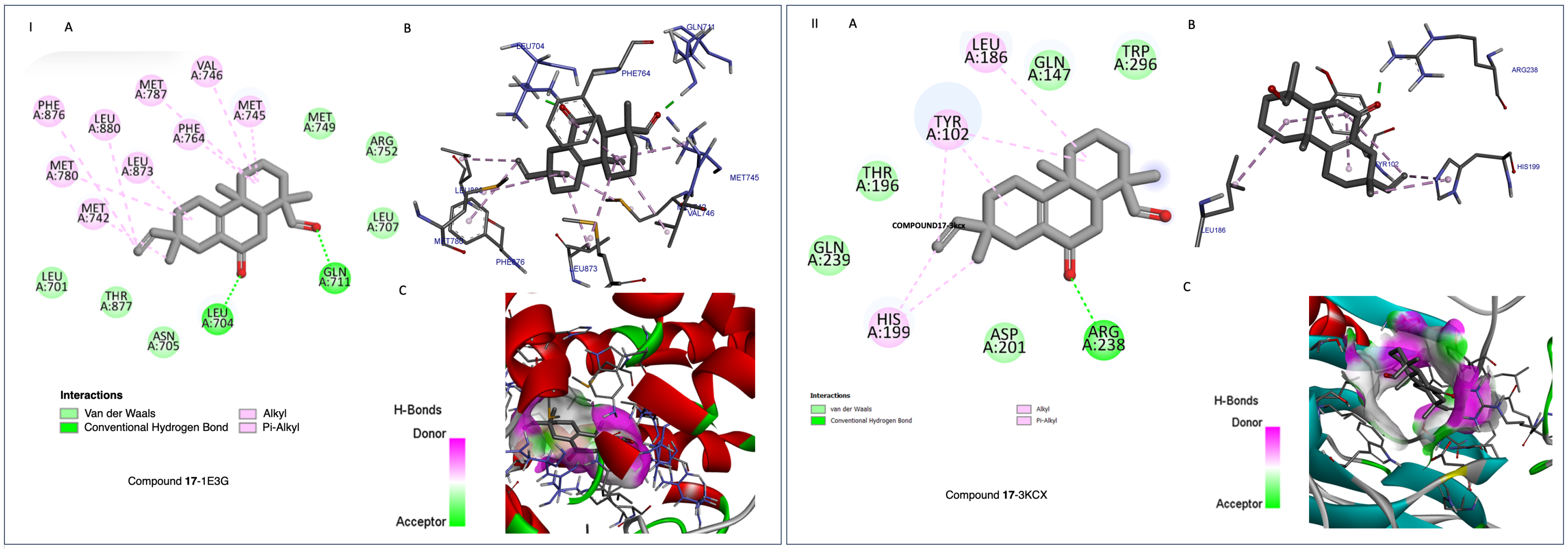

Figure S114. Compound **17**-1E3G and-3KCX: 2D and 3D image of the interaction between compound **17** with 1E3G and-3KCX receptor.
